# Supplementary material for: Mapping variants in thyroid hormone transporter MCT8 to disease severity by genomic, phenotypic, functional, structural and deep learning integration
Source: Nat Commun. 2025 Mar 12;16:2479. doi: 10.1038/s41467-025-56628-w (PMC11904026; doi:10.1038/s41467-025-56628-w)
Supplement: Supplementary file 1 — Supplementary Information [file 41467_2025_56628_MOESM1_ESM.pdf]

Supplementary Information to 'Mapping variants in thyroid hormone transporter MCT8 to disease severity by genomic, phenotypic, functional, structural and deep learning integration'.

Complete author list

Supplementary Note 1

Supplementary Tables

Supplementary Figures

References

Full (uncropped) immunoblots

Complete author list

Stefan Groeneweg*, Ferdy S. van Geest*, Mariano Martín[#], Mafalda Dias[#],, Jonathan Frazer[#], M. Carolina M. Medina-Gomez[#], Rosalie B.T.M. Sterenborg[#], Hao Wang[#], Anna Dolcetta-Capuzzo, Linda J. de Rooij, Alexander Teumer, Ayhan Abaci, Erica L.T van den Akker, Gautam P. Ambegaonkar, Christine M. Armour, Iiuliu Bacos, Priyanka Bakhtiani, Diana Barca, Andrew J. Bauer, Sjoerd A.A van den Berg, Amanda van den Berge, Enrico Bertini, Ingrid M. van Beynum, Nicola Bruntetti-Pierri, Doris Brunner, Marco Cappa, Gerarda Cappuccio, Barbara Castellotti, Claudia Castiglioni, Krishna Chatterjee, Alexander Chesover, Peter Christian, Jet Coenen - van der Spek, Irenaeus F.M. de Coo, Regis Coutant, Dana Craiu, Patricia Crock, Christian DeGoede, Korcan Demir, Cheyenne Dewey, Alice Dica, Paul Dimitri, Marjolein H.G Dremmen, Rachana Dubey, Anina Enderli, Jan Fairchild, Jonathan Gallichan, Luigi Garibaldi, Belinda George, Evelien F. Gevers, Erin Greenup, Annette Hackenberg, Zita Halász, Bianka Heinrich, Anna C. Hurst, Tony Huynh, Amber R. Isaza, Anna Klosowska, Marjo S. van der Knaap, Marieke M. van der Knoop, Daniel Konrad, David A. Koolen, Heiko Krude, Abhishek Kulkarni, Alexander Laemmle, Stephen H. LaFranchi, Amy Lawson-Yuen, Jan Lebl, Selmar Leeuwenburgh, Michaela Linder-Lucht, Anna López Martí, Cláudia F. Lorea, Charles M. Lourenço, Roelineke J. Lunsing, Greta Lyons, Jana Malikova, Edna E. Mancilla, Kenneth L. McCormick, Anne McGowan, Veronica Mericq, Felipe Monti Lora, Carla Moran, Katalin E. Muller, Lindsey E. Nicol, Isabelle Oliver-Petit, Laura Paone, Praveen G. Paul, Michel Polak, Francesco Porta, Fabiano O. Poswar, Christina Reinauer, Klara Rozenkova, Rowen Seckold, Tuba Seven Menevse, Peter Simm, Anna Simon, Yogen Singh, Marco Spada, Milou A.M Stals, Merel T. Stegenga, Athanasia Stoupa, Gopinath M. Subramanian, Lilla Szeifert, Davide Tonduti, Serap Turan, Joel Vanderniet, Adri van der Walt, Jean-Louis Wémeau, Anne-Marie van Wermeskerken, Jolanta Wierzba, Marie-Claire Y. de Wit, Nicole I. Wolf, Michael Wurm, Federica Zibordi, Amnon Zung, Nitash Zwaveling-Soonawala, Fernando Rivadeneira, Marcel E. Meima, Debora S. Marks, Juan P. Nicola, Chi-Hua Chen, Marco Medici, W. Edward Visser

*joint first authors, [#]joint second authors

Supplementary Note 1

Structural modeling

Unravelling the three-dimensional structure of integral membrane proteins has been proven to be a challenging procedure. Up to 2020, of the ~7100 human proteins with experimentally determined (partial) protein structure available, only 1260 were single-pass or multi-pass transmembrane proteins, covering ~18% of all integral membrane proteins [1]. Therefore, homology modeling of the membrane protein of interest has long been the most suitable alternative to gain structural insights.

MCT8 belongs to the Multi Facilitator Superfamily (MFS) of transporter proteins, and is thought to act according to the rocker-switch model. Rocker-switch proteins are made up of two helical bundles that contain a pseudo-2-fold symmetry axis that runs through the center of the transporter and perpendicular to the cell membrane [2]. During the transport cycle, the protein moves around the substrate molecule and thereby alternately exposing the substrate binding site to each side of the membrane.

Making advantage of the recently published cryo-EM structures of the human monocarboxylate transporters MCT1 and MCT2, we established novel homology models of MCT8 in outward-open and inward-open configuration, with overall z-scores of -0.590 and -0.771, respectively (Supplementary Fig. 16-18), which constitutes a significant improvement compared to previously available models [3]. Resulting models were compatible with observations from previously published *in vitro* studies, particularly those identifying residues accessible for chemical modification [3, 4, 5]. Although the exact orientation of residue sidechains should be inferred from homology models with some caution, key residues implied in substrate recognition (e.g. R445 and D498 – aligning to the critical residues R313 and F367 in human MCT1 [6, 7]) faced the substrate channel and formed hydrogen bonds with a T4 substrate molecule docked at the substrate binding center in outward-open conformation (Supplementary Fig. 18C). The bottom of the substrate binding center is predominantly composed by

aliphatic and aromatic residues (e.g. F189, F410, and Y409), that form a hydrophobic niche surrounding the inner and outer ring of T4. The aromatic ring of Y409 was moreover predicted to be alternately involved in a cation-pi interaction with the primary amine group of thyroid hormone, which also formed a hydrogen bond with D498. In addition to hydrophobic residues, the walls of the substrate pore are composed by several polar residues, including N193, S313, S314, and S317 that predominantly face the 5 and 5'iodine moieties. Following the current paradigm that MCTs translocate their substrates according to the rocker switch model, substrate interacting residues at the substrate binding center in outward-open and inward-open conformation were grossly the same (Supplementary Fig. 18D).

In order to explore if a common substrate interaction signature is present in thyroid hormone-interacting proteins, we compared the ligand-binding domains of human TRα, TRβ and the thyroid hormone binding protein mu-Crystallin, which have all been crystallized over the past decades, with the architecture of the predicted substrate binding center of MCT8 (Supplementary Fig. 19A-D). All three thyroid hormone interacting proteins contain an Arg residue at the substrate binding center that forms a hydrogen bond with the negatively charged carboxylic acid group of thyroid hormone, which corresponds to the role of R445 in MCT8. Like MCT8, a histidyl group interacts with the phenolic hydroxyl group and the outer ring of thyroid hormone in both TRα and TRβ. The importance of this interaction is illustrated by the functional impairment caused by variants affecting this His435 residue in TRβ, leading to a clinical phenotype of resistance to thyroid hormone (RTH)β [8]. In mu-Crystallin such a His residue is lacking, which could be related to the position of the phenolic hydroxyl group just outside the substrate binding center causing exposure to the cytosolic environment. Similar to the predicted substrate binding center of MCT8, the vast majority of the substrate binding center of TRα and β is composed of aliphatic residues that form hydrophobic interactions with the inner and outer ring of thyroid hormone. Distortion of these hydrophobic niches, as is for example the case in the TRα M256T variant [9] and TRβ L341V variant [10], has been previously shown to interfere with T3-signaling, suggesting that at least in TRs the exact composition of these hydrophobic niches is important for

proper substrate binding and/or recognition. Taken together, it appears that the presence of a positively charged Arg residue near the carboxylic acid group of thyroid hormone, as well as the presence of hydrophobic niches enclosing the inner and outer ring are mandatory features to allow thyroid hormone recognition. It is conceivable that the architecture of the pockets accommodating the iodine moieties is also a critical factor, although it is currently unknown if the iodine moieties are involved in specific interactions that are required for proper substrate recognition and binding.

As a complementary approach, a structural model of MCT8 in outward-open configuration was derived from the AlphaFold2 server [11]. Although the overall z-score of the AlphaFold model was -0.100 and therewith outperformed the outward-open model constructed with classical homology modeling in YASARA (Supplementary Fig. 20A and B), it was not fully compatible with the available *in vitro* data. In particular, the side-chain of H192 in the AlfaFold model does not flank the substrate pore and is therefore not accessible for chemical modification or interaction with the phenolic-hydroxyl group of T4 as has been suggested by previous functional studies (Supplementary Fig. 20C and D) [4]. This discrepancy may be inherent to the poor conservation of this His-residue across species and among the MCT-family. The higher Z-score of the AlphaFold model was mainly driven by a superior 3D packaging of the proposed intracellular N- and C-terminal domains, as well as the intra- and extracellular loop structures, whereas differences in the TMDs were less apparent, or even in favor of the YASARA model (Supplementary Fig. 20E). Indeed, trimming of the N- and C-terminus already improved the Z-score of the YASARA model up to -0.330, which approximates the model quality of an average high-resolution X-ray structure. These observations suggest that in particular the loop structures and large intracellular domains are intrinsically disordered or unstructured, which is reflected by the high RMSD of residues within these domains (11.06±8.33), and relatively low RMSD for residues within the predicted TMDs (3.76±1.94) when comparing the YASARA and AlphaFold2 structures (Supplementary Fig. 20F). A similar RMSD profile was observed after 2ns simulation of the YASARA-derived model (Supplementary Fig. 20G). The RMSD of individual residues indeed strongly correlates with the distance of the involved residue to the center of the substrate binding center

(Supplementary Fig. 20H), corroborating that residues located in the loop regions have a lower degree of structural organization. In line, such residues were found to be relatively less well conserved among species (Supplementary Fig. 20I) and within the MCT protein family (Supplementary Fig. 20J). Moreover, these intra- and extracellular domains had a relatively low predictive accuracy in both models.

As the YASARA-derived model was most compatible with the available experimental data, this model was used to inform on the impact of variants on MCT8 structure.

Identifying functional residues through systematic alanine scanning mutagenesis

Alanine scanning mutagenesis is a widely used technique employed to identify functionally important residues of proteins. We systematically investigated the effects of alanine substitutions in MCT8 with T3 and T4 transport studies to identify critical regions within the protein and gain insights into the mechanism by which MCT8 transports its substrate(s). Informed by our structural models and previous functional studies, Ala scanning was restricted to the TMDs and their connecting intracellular and extracellular loops, as the large intracellular N- and C-termini likely have a low degree of structural organisation and had no functional significance in our experimental system [12, 13]. Hence, we generated 375 alanine variants (including 21 alanine blocks and 1 Ala variant identified in a patient with MCT8 deficiency), covering 61% of the entire MCT8 protein (66% of non-alanine residues) and 100% of non-alanine residues in the selected region.

We defined the individual alanine variants as severe (<10% transport function compared to WT), moderate (10-40% transport function compared to WT), or mild (40-70% transport function compared to WT) loss of function (LoF) variants. Definition of these cutoffs was guided by the residual transport

capacity of known pathogenic variants, identified in individuals with clinical features of MCT8 deficiency. Specifically, severe LoF in the alanine scanning corresponds to severe and moderate LoF variants in the genotype-phenotype analyses. Moderate LoF in the alanine scanning corresponds to all other (mild) LoF variants in the genotype-phenotype analyses; with 36% residual uptake as the highest residual T4 uptake in patient variants expressed in JEG3 cells, a 40% as upper limit was used. Mild LoF in the alanine scanning was defined as 40% to 70%; an upper limit of 70% allows for a robust detection from WT function. These studies revealed that 3% (11/375) of Ala variants had severe impact on MCT8 function, whereas 5% (20/375) had moderate and 15% (55/375) had mild impact on MCT8 function (Fig. 3A; Supplementary Fig. 13A). Most of the inactivating Ala variants were located along the substrate channel or were (in)directly involved in the orientation of side-chains that flank the substrate channel (Fig. 3B; Supplementary Fig. 13B; 21A). Functionally important residues (i.e. residues of which the Ala substituent had <70% residual transport activity compared to WT) could be categorized into seven different functional groups (Fig. 3C, Supplementary Fig. 21B): 1) residues located at the substrate binding center, 2) residues flanking the substrate channel outside the substrate binding center with potential gating function, 3) residues that support the structure of (critical parts of) the substrate channel, 4) residues within cluster 1 composed of the first part of TMD5 and the second part of TMD8, 5) residues within cluster 2 composed of the second part of TMD2 and the first part of TMD11, 6) residues within a “linker region” connecting cluster 1 and cluster 2 with potential substrate-interacting residues, 7) a residual group that mostly contains residues involved in maintenance of a) helix structure (i.e. Gly and Pro), or b) provide structural support for functionally important residues in group 4-6. Ala substitutions of almost all residues within TMD3, 6, 9, and 12 were well-tolerated, which is in line with the general assumption that these TMDs form contacts with the lipid bilayer and that, as a consequence, the preservation of hydrophobicity might be more relevant than the exact side-chain composition. Indeed, of the 67 different missense variants identified in patients with MCT8 deficiency, only 11 (16.4%) affect residues within these TMDs. Moreover, 6 (55.0%) out of these 11 variants were classified as mild, 2 (18.0%) out of 11 as moderate, and 3 (27.0%) out of 11 as severe LoF variants, with

the latter three variants (i.e. G251E, L471R and G564R) causing major changes in amino acid properties and introducing side-chains that are poorly compatible with a hydrophobic membrane environment (Fig. 3I).

Group 1: Critical residues identified at the substrate binding center

The first group constitutes residues located at the proposed substrate binding center (i.e. <4 Å from the substrate molecule, docked at the deepest point of the substrate channel). In line with experimentally determined structures of other MFS-members (reviewed in e.g. [2, 14, 15]), the substrate binding center of MCT8 is mainly composed of TMD1, 4, 7, and 10. Of the 16 residues located at the substrate binding center, only the substitutions of F189 (TMD1), S314 (TMD5), F501 and I502 (TMD10) by an Ala were tolerated, whereas all other Ala substituents caused a functional reduction (Supplementary Fig. 21C-E). This was most prominent for those residues that are predicted to form electrostatic interactions with thyroid hormone. In particular the D498A and R445A variants resulted in (near-) complete inactivation of MCT8-mediated thyroid hormone transport, which is in line with previous studies [16, 17, 18]. The R445 residue, located within TMD8, is strongly conserved within the MCT family. Also in MCT1, used as a template for our structural model(s), the corresponding R313 residue is critical for the transport of its substrates [6, 7, 15], and mutation of which to a Gln has been associated with clinical features of MCT1 deficiency [19]. In MCT8, the η-amino groups of R445 may form a (transient) hydrogen-bond with the carboxyl group of T3 and T4, which was also suggested by previous homology models [3, 17, 18]. Despite D498 (TMD10) not being conserved among MCTs, its corresponding residue in MCT1, F367, is critical for MCT1 function and implied in substrate selection [15]. As in our previous model in outward-open conformation [3], during molecular dynamic simulations in absence of substrate molecules, a (transient) hydrogen bond between a η-NH2 group of R445 and the γ-COOH group of D498 was observed, whereas this interaction was absent in the presence of substrate. Although data from molecular simulations should be interpreted with caution, this may suggest that the presence of a substrate molecule disrupts this hydrogen bond and thereby allows local

conformational changes required for substrate translocation. In line with Groeneweg et al [4], loss of the imidazole-group of H192 and, therewith, the proposed hydrogen bond with the phenolic hydroxyl group of substrate [3, 17, 18], reduced thyroid hormone uptake by up to 40% (Supplementary Fig. 21E). Other members of the MFS, including GlpT (R45) and FucP (N45) contain residues at the corresponding position that are implied in substrate interaction [20, 21, 22], supporting an important role for residues at this location in TMD1 in at least several MFS-members. The solvent accessibility of the H192 was previously confirmed by chemical modification studies with the His-reactive compound diethylpyrocarbonate (DEPC) [4].

Substitution of G196 (TMD1) and G499 (TMD10) by an Ala resulted in a moderate LoF, potentially through local loss of backbone flexibility. This could interfere with the orientation of their neighbouring substrate-interacting residues (e.g. H192 and D498, respectively), or potential gating residues that prevent the substrate from exiting the substrate binding center once bound. Elongation of their side-chains may moreover pose structural interference with either the substrate molecule or other residues facing the substrate channel. Loss of the aromatic residues F229 (TMD2), Y409 (TMD7), F410 (TMD7) all resulted in a mild-moderate functional reduction, which was also observed upon the reduction of the side-chain length of the aliphatic residues V414 (TMD7), L434 (TMD8), and M225 (TMD2) in their corresponding Ala variants. These variants likely alter the shape of the hydrophobic niche accommodating the inner and outer ring as well as the iodine moieties.

Besides the charged residues R445 and D498, substitution of N193 in TMD1 by an Ala had a major impact on T4 transport, whereas the transport of T3 was only mildly affected (Supplementary Fig. 21F). These findings suggested a particular role for this residue in the recognition and/or translocation of T4 along the substrate channel, with a potential involvement of the C5'-iodine moiety. To further pinpoint the function of the N193 residue, we generated expression constructs in which N193 was substituted by an Ile (loss of amide and similar size) in addition to the Ala substituent (loss of the amide side-chain and smaller size). Both variants were effectively expressed at the cell membrane (Supplementary Fig.

21I), but severely reduced MCT8-mediated T4 uptake, whereas T3 uptake was clearly less affected (Supplementary Fig. 18F). Both N193 mutants reduced the apparent affinity for T3, indicated by 2-3-times higher Km values than WT (Supplementary Fig. 18G,H). In case of N193A, this was accompanied by a 2-times increase in Vmax over WT, suggesting that the reduced affinity of this variant has a favorable effect on the (maximum) transport rate of T3. By contrast, the increased apparent Km for T4 was accompanied by a decrease in apparent Vmax for both mutants. The lower affinity of both mutants for T4 may thus have unfavorable effects on T4 transport rate. To further understand the role of N193 in the transport cycle, we combined substrate docking and molecular morphing to construct the trajectory of T4 through the substrate pore. Just before entering the substrate binding center, the distance and angle between the side-chain oxygen of N193 and C5-iodine of T4 allows the formation of a transient halogen bond (Supplementary Fig. 21J) [23]. Simultaneously, the distance and orientation of the C5'-iodine allows the formation of second halogen bond to the side-chain nitrogen of N193, which appears particularly important in fine-tuning the orientation of the large outer ring of T4 (Supplementary Fig. 21J). To further support that loss of N193 has the greatest impact on the transport of substrates with a fully saturated outer ring, we also studied the direct uptake of 3,3',5'-triiodothyronine (rT3) and 3,3'-diodothyronine (T2) (Fig. 3F; Supplementary Fig. 21F). Indeed, the effect of the N193A variant on 3,3'-T2 uptake was smaller than that on T4 uptake, whereas an intermediate effect was observed for rT3. Notably, N193 aligns to K38 in hMCT1, which has been implied in the recognition and binding of lactate by MCT1 [15].

Together, these data support the concept that electrostatic interactions with the amine and carboxylic acid moieties of the alanine side-chain of thyroid hormone, as well as the phenolic hydroxyl group are key determinants in substrate recognition and translocation. Extending on previous studies on MCT8 (and other members of the MFS), these data also indicate that the exact composition of the hydrophobic niches accommodating the inner and outer ring with their iodine moieties importantly determine transport efficacy [3, 24]. In particular the N193 may exert a specific role in positioning the C5'- and, to a lesser extent, the C5-iodine moiety of T4, possibly due to the formation of halogen bond(s).

Group 2: residues flanking the substrate channel outside the substrate binding center

The second group of functional residues constitutes those residues that are not directly located at the substrate binding center, but do flank the substrate channel at other sites in the outward-open conformation (Supplementary Fig. 22A). The critical residues in this category are exposed to the extracellular environment and are located within the second part of TMD1 (I197, Y199, L203), TMD7 (Y413, L416) and the first part of TMD8 (L433, I437, S441), and TMD10 (L494) (Supplementary Fig. 22B-C). It is conceivable that these residues help improving the orientation of the substrate molecule before entering the substrate binding center through either direct or indirect interactions with the substrate molecule, or have an important gating function once the substrate is localized at the substrate binding center.

Group 3: critical residues (indirectly) supporting structure of substrate channel

The third group of residues may determine the orientation of substrate-interacting residues classified to group 1 and 2 and thereby (indirectly) determine the shape of the substrate channel (Supplementary Fig. 22D). This group of residues include residues that 1) potentially determine the helical structure of the TMDs composing the substrate binding center (e.g. G186, TMD1; G495, TMD10) (Supplementary Fig. 22E) or 2) their relative position to other TMDs (e.g. I188 and I191, L198 TMD1) (Supplementary Fig. 22F), or 3) residues located in other TMDs, providing structural support to the TMDs that form the substrate binding center, exemplified by L259 (TMD3), F316, F320, F324 (TMD5), L340 (TMD6), L487 (TMD10), M533 (TMD11), F554 (TMD12) and most prominently by a series of residues within the first part of TMD4 (Supplementary Fig 22G and H). Several residues within the first half of TMD4 interact with G196, I188 and I191, as well as with several substrate-interacting residues within TMD1 and TMD2. Ala substitution of these residues within TMD4 resulted in moderate (R271, F279, G280), or mild (L268, S269, Y275, G282, C283) LoF (Supplementary Fig 22I). These residues presumably provide direct support to the orientation of substrate-interacting residues and determine the relative position of TMD1 and TMD2 and thereby the shape of the substrate channel. Notably, Ala substituents of

residues within TMD4 that have side-chains pointing away from TMD1 and TMD2 are generally well-tolerated.

Group 4: residues within cluster 1 (TMD5 and TMD8)

The fourth group concerns a cluster of critical residues within the first part of TMD5 (R301-A311, with critical residues: L302, G303, L304, G307, V309) and second part of TMD8 (R445-D453, with critical residues: L446, G449, S452, D453), and their flanking intracellular loops (Supplementary Fig 23A-C). In MFS-transporters, TMD5 and TMD8 (as well as TMD2 and TMD11, see next section) are generally termed rocker-helices, to indicate the fact that they are directly involved in inter-domain conformational changes. Indeed in outward-open conformation, critical residues within this cluster form extensive hydrophobic and electrostatic interactions between TMD5 and TMD8, and therewith determine the relative position of the N-terminal and C-terminal half of the MCT8 protein (Supplementary Fig. 23B). Notably, Ala substituents of residues within this part of TMD5 and TMD8 not involved in the interactions between both TMDs, but rather face the lipid bilayer, are well-tolerated (Supplementary Fig. 23C).

Exemplary, critical residues D453 and R301 are predicted to form a salt bridge, that is perturbed once MCT8 is in inward-open conformation (Supplementary Fig. 23D-E). Whereas substitution of either one of these residues by an Ala results in (near-) complete inactivation of T4 transport, substitution of Arg301 by a positively charged Lys restored T4 transport to 80.5% of WT (Supplementary Fig. 23F). By contrast, all tested substitutions of D453, including the D453E and D453N, resulted in (near-)complete inactivation, suggesting that in addition to its negative charge, the exact side-chain length of D453 is also crucial (Supplementary Fig. 5 and 23F). As cluster 1 is in close structural proximity of, and in direct contact with the substrate-interacting residue R445, we hypothesize that interactions within this cluster may be influenced by the presence of substrate and could therefore be part of the mechanism involved in the establishment of substrate-induced conformational changes.

Group 5: residues within cluster 2 (TMD2 and TMD11)

A similar cluster appears to be present within the second part of TMD2 (M225-I234 with critical residues: t225, G226, F229, P233) and TMD11 (S519-A530 with critical residues: S519, I522, L526, G527, M529), that mirrors TMD5 and TMD8 at the other side of the protein (Supplementary Fig. 23G-I). Critical residues within this pair of rocker-helices also form extensive hydrophobic interactions that likely determine the position of TMD2 within the N-terminal half of the protein in relation to TMD11 within the C-terminal half of the protein. Indeed, in inward-open conformation interactions between the most intracellularly located residues in both TMDs are perturbed (Supplementary Fig. 23J-K). One of the residues that might be of particular importance in linking both TMDs is I522, substitution of which by an Ala resulted in moderate reduction of uptake function (Supplementary Fig. 23L). Preservation of the branched-chain aliphatic properties in the Ile522Leu variant resulted in a fully active transporter (Supplementary Fig. 23L). Again, Ala substituents of residues within TMD2 and TMD11 not involved in the interactions between both TMDs are well-tolerated. As for R445 in cluster 1, Met225 forms extensive interactions with the substrate molecule, and therefore interactions within this second pair of rocker-helices may also be influenced by the presence of substrate.

Group 6: linker region composed of residues within TMD4, ICL2, and TMD10

The sixth group of critical residues is located in the center of the protein and entails the second half of TMD4 (residues: F287-G295), intracellular loop 2 that connects TMD4 and 5 (residues H296–R301), as well as the second half of TMD10 (M505-F510). These residues are located in close structural proximity, below the level of substrate binding pocket, and are, as such, not accessible from the extracellular side of the transporter (Supplementary Fig. 24A and B). Almost all residues within this part of TMD4, ICL2, and TMD10 are important to MCT8 function as their Ala substituents cause severe (Y297, F298), moderate (R301, F287, F510), or mild (Q288, P289, L291, L294, M505, P507) LoF (Supplementary Fig. 24C). We postulate that this group of residues has at least two important functions. Firstly, in outward-open conformation, these residues may form a “linker region” between both pairs of rocker helices and therewith orchestrate a coordinated conformational change of the transporter in presence of

substrate, a process that might be further guided by the potential interactions of the first part of TMD4 with substrate-interacting residues (Supplementary Fig. 22H). Secondly, the linker region itself, as well as previously indicated residues within both rocker helices, are exposed to the proposed substrate channel in the inward-open conformation (Supplementary Fig. 24D-F), and may therefore play a direct role in propelling the substrate molecule from the substrate binding pocket to the intracellular end of the substrate channel.

A role of this linker region in substrate selection and transition through the substrate channel is supported by studies of Johannes *et al* [24], showing that Y184, corresponding to F287 in MCT8, is a key determinant of the substrate pocket size of MCT10 and is one of the residues that prevents MCT10 from transporting T4 efficiently. The authors suggest that alteration of the side-chain length at this position may change the size of the substrate pocket, and therewith exclude T4 as a prime substrate.

Particularly Y297 and F298 might be of particular importance to stabilize the structure of the linker region, as their Ala substitutions resulted in (near-)complete functional inactivation and strong reduction of (membrane) expression of the respective mutant MCT8 proteins. Particularly the aromatic moieties of both residues are likely to exert critical functions, potentially by forming a multitude of interactions, as maintenance of these aromatic properties in the Y297F and F298Y variants, respectively, preserved full MCT8 function (Supplementary Fig. 24G-L). It appears that this structural feature is strongly conserved among species and among members of the MCT family (Supplementary Fig. 15).

Group 7: residual group

The final group constitutes a residual group with residues that are dispersed throughout the MCT8 protein and may be involved in 1) maintenance of overall protein structure, such as Gly and Pro residues (Supplementary Fig. 25A), 2) interactions with other functional domains within MCT8 (i.e. group 4-6) (Supplementary Fig. 25B), or 3) determining loop conformation (Supplementary Fig. 25C).

Firstly, by increasing backbone flexibility, Gly residues allow dynamic changes within and between helical bundles. In addition to the Gly residues that are part of group 1-6, Ala substituents of G251 (TMD3) and G536 (TMD11) resulted in severe LoF, of G243 (ICL1) in moderate LoF, and of G171 (N-terminus) and G548 (ECL6) in mild LoF (Supplementary Fig. 25A and D). Except for G548, these Gly residues are highly conserved across species and within the MCT family, suggesting a common structural function (Supplementary Fig. 15). Another residue that greatly impacts helical structure is Pro, which typically functions as a helix-breaker. Loss of this unique characteristic in case of Ala substitution of P532 and P538, both located inTMD11, result in moderate LoF (Supplementary Fig. 25A and D).

Secondly, several residues that are located in close structural proximity of the “linker region” or cluster 1 showed mild-moderate LoF upon substitution by Ala (Supplementary Fig. 25B and D). Within TMD9, Q464 and K470 are interacting with residues within TMD10 that belong to the linker region. The same Lys470 residues also interacts with cluster 1 residues, as is the case for F500 (TMD10) and W175 (TMD1). Glu170, located at the border of the intracellular N-terminus and TMD1, also interacts with TMD4/ICL2 of the linker region. Based on its location, the E170 residue might also be important for the correct determination of TMD topology.

Thirdly, substitution of some residues located within the intracellular or extracellular loops to an Ala showed mild LoF, including F547 and D549 in ECL6 (Supplementary Fig. 25C and D). To exclude that any other potentially critical residues within intra- or extracellular loops were missed by focussing our mutational approach on the TMDs, up to 26 so-called Ala-blocks were generated in which several consecutive residues were substituted by an Ala in the same expression construct. However, only 6 out of 26 so-called Ala-blocks showed pronounced LoF (Supplemental Fig. 26). In case an Ala-block showed >50% functional reduction, individual Ala variants were tested. Importantly, only substitution of G243 and I478 resulted in significant LoF, whereas all other individual loop variants were well-

tolerated. This may suggest that the involved loop domains (i.e. ICL1, ECL4, ECL5, ICL5) may harbour functions critical to MCT8 – although the exact composition tolerates some variation.

From a structural perspective, it is unclear why Ala substitution of particularly Y354 and M476 (severe), but also S269 and Y462 (mild), result in LoF. Variants affecting Y354 (i.e. Y354C) have also been identified in patients with MCT8 deficiency, supporting that this residue might indeed be critical to MCT8 function. In depth mutational studies, also shown in Supplementary Fig. 25E, indicated that the aromatic features of Y354 are of importance, as substitution by a Phe restored T4 transport to WT levels. As Y354C greatly diminished MCT8 protein expression levels (Supplementary Fig. 5), as well as membrane expression levels (Supplementary Fig. 25F), we postulate that Y354 has a critical role in maintaining protein stability. This is supported by the extensive interactions Y354 forms with residues in surrounding TMDs (Supplementary Fig. 5).

Delineating pathogenic mechanisms of missense variants in MCT8 deficiency

Having identified the critical residues of the MCT8 protein, we next employed further functional studies to delineate the underlying pathogenic mechanism of missense variants identified in MCT8 deficiency. T3 and T4 transport function, as well as total and cell surface MCT8 protein expression levels were determined for all identified missense variants in transiently transfected cells. Next, the information obtained from the Ala scanning was employed to determine whether the loss of the original residue was the major pathogenic determinant of the variant, or the introduction of an unfavorable alternate residue. Third, in case the Ala variant was also pathogenic, the original residue was either changed into a residue with similar properties, and/or its equivalent in the highly homologues transporter MCT10, in order to pinpoint what property of the original residue was vital for MCT8 function. Finally, the findings were interpreted in the context of the novel MCT8 homology models, cross-species and cross-family conservation analyses, as well as previously reported functional

studies involving MCT family members. A summary of these results is provided in Supplementary Table 6, and Supplementary Fig. 5.

From the 67 different pathogenic missense variants, 5 variants affected an original Ala residue and 1 was a combined missense variant (L487R+V489D). Out of the remaining 61 pathogenic missense variants, 33 variants affected critical residues (i.e. residues of which the Ala substituent resulted in significant functional reduction) at 25 different positions. Eleven out of these 25 original residues concerned Gly or Pro residues, substitution of which presumably causing loss of the unique structural properties of both residues. Complementary mutational screening elucidated the key property/properties of 13 out of 14 of the remaining affected critical residues. For example, replacement of D498 by Asn (patient-derived mutation) or Ala both abolished thyroid hormone transport, while substitution by Glu (having similar charge as Asp) did not affect function (Fig. 3I, Supplementary Fig. 5). Similarly, substitution of R445 by an Ser, Leu, Asp, Cys or Ala resulted in substantial loss of function, whereas substitution by Lys (having similar charge) was relatively well tolerated (Supplementary Fig. 5).

As a corollary, 28 out of 61 pathogenic variants affected non-critical residues, and therefore the introduction of an unfavorable residue is likely to cause loss of function. Indeed, 26 out of 28 variants showed reduced total and/or membrane expression levels (<70% of WT levels), suggesting interference of the variant with proper protein stability and/or membrane trafficking. For example, substitution of C283 by a Tyr strongly reduced MCT8 expression levels. Structural modelling indeed suggests that this variant greatly distorts the helical structure of TMD4 (Fig. 3I, Supplementary Fig. 5). The two variants that retained normal (membrane) expression were S232F and L471R. Both variants increase side-chain size , which may cause steric clashes with residues predicted to flank the substrate channel and thereby (in)directly hamper substrate passage (Supplementary Fig. 5).

Supplementary Tables

Supplementary Table 1. Demographic characteristics

Characteristic	Reference cohort N=151	Literature and newly identified patients N=220	Meta-analysis N=371
**Age at last follow-up (years)**	9.1 (1.0 to 71.0)	7.3 (0.3-76.0)	8.7 (3.5-17.8)
**Age composition**			
<4 years	27 (18.6%)	74 (37.0%)	101 (29.3%)
4-10 years	57 (39.3%)	44 (22.0%)	101 (29.3%)
11-17 years	35 (24.1%)	24 (12.0%)	59 (17.1%)
Adults (≥18 years)	26 (17.9%)	58 (29.0%)	84 (24.3%)
**Sex**			
Female	0	0	0
Male	151 (100%)	220 (100%)	371 (100%)
**Alive**	119 (78.8%)	166 (75.5%)	284 (76.6%)
**Ethnic origin**			
Caucasian	100 (71.9%)	31 (50.0%)	131 (65.2%)
Middle-Eastern	13 (9.4%)	1 (1.6%)	14 (7.0%)
North-African	7 (5.0%)	0 (0.0%)	7 (3.5%)
South-American	9 (6.5%)	13 (21.0%)	22 (10.9%)
Asian	8 (5.8%)	7 (11.3%)	15 (7.5%)
Other	2 (1.4%)	10 (16.1%)	12 (6.0%)
**Race**			
White	120 (86.3)	34 (54.8%)	154 (76.6%)
Other	19 (13.7)	28 (45.2%)	47 (23.4%)
**Patients per country**			
Australia	5 (3.3%)		
Belgium	3 (2.0%)		
Brazil	7 (4.6%)		
Canada	5 (3.3%)		
Chile	2 (1.3%)		
Czech Republic	1 (0.7%)		
France	13 (8.6%)		
Germany	9 (6.0%)		
Hungary	3 (2.0%)		
India	8 (5.3%)		
Israel	4 (2.6%)		
Italy	21 (13.9%)		
Netherlands	23 (15.2%)		
Poland	5 (3.3%)		
Romania	6 (4.0%)		
South-Africa	1 (0.7 %)		
Spain	1 (0.7%)		
Sweden	2 (1.3%)		
Switzerland	3 (2.0 %)		
Turkey	6 (4.0%)		
UK	18 (11.9%)		
USA	5 (3.3%)		

Data are median (range), or n (%). Demographic information of patients at the time they were enrolled in the cohort, based on available data. As country of origin is infrequently reported and cannot be accurately inferred by the affiliations of the authors – this parameter has not been included for patients identified through the systematic literature search.

Supplementary Table 2. In depth phenotyping of neurodevelopmental features.

	Reference cohort [25]		Literature and newly identified patients		Meta-analysis	
	N=151		N=220		N=371	
		N		N		N
Age at assessment (years)	4.8 (0.44-66.8)	86	6.00 (0.04-76.0)	159	5.12 (0.04-76.0)	270
**Perinatal features**						
Pregnancy duration (weeks)	40.0 (32.0-42.3)	34	40.0 (35.0-42.0)	47	40.0 (32.0-42.3)	97
Apgar scores >8 after 5 min	15 (93.8%)	16	16 (94.1%)	17	35 (94.6%)	37
Term birth weight (grams)	3584 (±517)	22	3390 (±553)	36	3499 (±549)	69
Microcephaly (<3th centile) at birth	2 (18.2%)	11	3 (12.0%)	25	6 (15.8%)	38
**Neurological examination**						
Hypotonia	72 (100.0%)	72	139 (97.2%)	143	239 (98.0%)	244
Primitive reflexes (>1 present)	51 (91.1%)	56	10 (83.3%)	12	63 (90.0%)	70
Tonic neck reflex	17 (81.0%)	21	5 (83.3%)	6	25 (83.3%)	30
Glabellar sign	44 (80.0%)	55	2 (66.7%)	3	46 (79.3%)	58
Startle response	17 (68.0%)	25	6 (85.7%)	7	24 (72.7%)	33
Scoliosis (>8 years)	15 (88.2%)	17	24 (61.5%)	39	42 (68.9%)	61
Muscle hypoplasia	43 (84.3%)	51	71 (83.5%)	85	132 (84.6%)	156
Dystonia	57 (82.6%)	69	86 (85.1%)	101	167 (85.2%)	196
Spasticity	57 (80.3%)	71	141 (92.8%)	152	226 (90.0%)	251
Urinary / faecal incontinence (>4 years)	33 (80.5%)	41	0 (0.0%)	2	33 (75.0%)	44
Feeding problems	55 (71.4%)	77	50 (80.6%)	62	116 (75.8%)	153
Hip dislocation (>8 years)	10 (66.7%)	15	N/A	N/A	10 (66.7%)	15
Plantar extension response (Babinski sign)	38 (66.7%)	57	33 (78.6%)	42	81 (73.0%)	111
Delayed visual evoked potentials (<1 year)	3 (50.0%)	6	1 (25.0%)	4	5 (38.5%)	13
Delayed visual evoked potentials (<1 year)	0 (0.0%)	3	9 (56.3%)	16	10 (43.5%)	23
Sleep problems	20 (39.2%)	51	6 (54.5%)	11	33 (47.8%)	69
Tube feeding	27 (35.5%)	76	18 (47.4%)	38	48 (39.0%)	123
Strabismus	19 (35.2%)	54	18 (66.7%)	27	40 (44.9%)	89
Microcephaly (<3th centile)	19 (32.2%)	59	20 (24.1%)	83	44 (28.0%)	157
Nystagmus	13 (26.5%)	49	9 (12.2%)	74	23 (17.2%)	134
Extrapyramidal signs (other)	7 (25.0%)	28	47 (97.9%)	48	78 (85.7%)	91
Seizures (EEG proven)	15 (23.1%)	65	13 (12.4%)	105	35 (18.6%)	188
Apneusis	7 (21.9%)	32	2 (10.5%)	19	12 (21.8%)	55
Abnormal hearing	1 (2.3%)	44	3 (27.3%)	11	4 (6.9%)	58
**Development**						
Head control	19 (24.7%)	77	24 (20.1%)	117	53 (23.0%)	228
Speech (at least 1 word)	5 (6.6%)	76	33 (21.7%)	150	44 (17.0%)	259
Independent sitting	6 (7.7%)	78	17 (15.3%)	111	29 (12.7%)	224
Independent walking	4 (5.2%)	77	20 (13.4%)	149	30 (11.3%)	261
**MRI/MRS characteristics***						
Normal global anatomy	13 (100%)	13	9 (100.0%)	9	22 (100.0%)	22
Delayed myelination	13 (100%)	13	74 (91.4%)	81	92 (90.2%)	102
Reduced cerebral white matter volume	13 (100%)	13	21 (87.5%)	24	34 (91.9%)	37
Periventricular white matter lesions	10 (100%)	10	7 (70.0%)	10	17 (85.0%)	20

Prominent supratentorial ventricular system	13 (100%)	13	18 (48.6%)	37	33 (58.9%)	56
Prominent peripheral liquor spaces	13 (100%)	13	16 (47.1%)	34	31 (58.5%)	53
Low NAA peak	6 (85.7%)	7	3 (50.0%)	6	10 (71.4%)	14
High choline peak	6 (85.7%)	7	4 (66.7%)	6	11 (78.6%)	14

Data are median (range), n (%), or mean (±SD). Phenotyping of the neurological phenotype in indicated cohorts. Please note that for the International MCT8 Deficiency Consortium cohort, only 86 patients with extensive available data were analyzed. In the meta-analysis, all (151) patients of the International MCT8 Deficiency Consortium cohort were included. Data from the International MCT8 Deficiency Consortium cohort study (reference cohort; [25]) have been updated for the meta-analysis.

*** MRI scans in the International MCT8 Deficiency Consortium cohort were centrally assessed as described before [25].**

Supplementary Table 3. In depth phenotyping of metabolic features.

	Reference cohort [25] N=151	N	Literature and newly identified patients N=220	N	Meta-analysis N=371	N
**Serum thyroid function tests**						
Age at measurement (years)	5.3 (0.44-66.8)	106	2.5 (0.04-62.0)	80	4.4 (0.04-66.8)	190
Elevated T3 concentrations	96 (95.1%)	101	28 (87.5%)	32	126 (93.3%)	135
Reduced free T4 concentrations	94 (88.7%)	106	76 (85.4%)	89	174 (87.4%)	199
**Deep phenotyping**						
Age at assessment (years)	4.8 (0.44-66.8)	86	6.00 (0.04-76.0)	159	5.12 (0.04-76.0)	270
**Biochemical measurements**						
Elevated sex hormone binding globulin	69 (88.5%)	78	6 (66.7%)	9	79 (86.8%)	91
Elevated alanine aminotransferase	30 (46.2%)	65	0 (0.0%)	1	30 (43.5%)	69
Reduced creatinine	22 (27.8%)	79	0 (0.0%)	1	24 (28.9%)	83
Elevated lactate	3 (27.3%)	11	5 (100.0%)	5	8 (50.0%)	16
Reduced total cholesterol	12 (18.5%)	65	2 (100.0%)	2	15 (21.4%)	70
Elevated aspartate aminotransferase	11 (19.6%)	56	0 (0.0%)	1	11 (18.3%)	60
Elevated creatine kinase	3 (3.8%)	79	1 (25.0%)	4	4 (4.7%)	86
**Clinical features**						
Low bone mineral density (>8 years)	5 (100%)	5	0 (0.0%)	1	5 (83.3%)	6
Gastro-esophageal reflux disease	38 (79.2%)	48	13 (68.4%)	19	58 (78.4%)	74
Premature atrial complexes	34 (75.6%)	45	0 (0.0%)	5	36 (67.9%)	53
Recurrent (pulmonary) infections	29 (69.0%)	42	14 (73.7%)	19	50 (72.5%)	69
Underweight (<-2 SD)	59 (71.1%)	83	56 (65.9%)	85	140 (68.6%)	204
Constipation	37 (58.7%)	63	5 (55.6%)	9	44 (57.1%)	77
Cryptorchidism	9 (18.4%)	49	17 (31.5%)	54	29 (25.4%)	114
Elevated systolic blood pressure	25 (53.2%)	47	1 (10.0%)	10	9 (15.3%)	59
Elevated diasystolic blood pressure	17 (36.2%)	47	3 (30.0%)	10	5 (8.5%)	59
Increased perspiration	29 (48.3%)	60	5 (62.5%)	8	35 (48.6%)	72
Short stature (<-2 SD)	27 (40.3%)	67	22 (25.0%)	88	53 (30.7%)	174
Premature ventricular complexes	19 (42.2%)	45	0 (0.0%)	7	19 (36.5%)	52
Tachycardia in rest	20 (31.3%)	64	6 (27.3%)	22	31 (32.6%)	95
Aortic root dilatation	7 (26.9%)	26	1 (25.0%)	4	8 (26.7%)	30
Delayed sexual maturation (>8 years)	5 (26.3%)	19	N/A	N/A	5 (26.3%)	19
Cardiac conduction abnormalities	9 (18.0%)	50	0 (0.0%)	8	3 (5.2%)	58
Prolonged QTc interval	3 (7.7%)	39	N/A	N/A	3 (7.7%)	39
Supraventricular tachycardia	2 (4.2%)	48	N/A	N/A	2 (4.2%)	48
(Non-sustained) ventricular tachycardia	2 (4.2%)	48	N/A	N/A	2 (4.2%)	48
Atrial fibrillation	1 (2.1%)	48	N/A	N/A	1 (2.1%)	48

Data are median (range), or n (%). Phenotyping of the peripheral phenotype in indicated cohorts. Please note that for the International MCT8 Deficiency Consortium cohort, only 86 patients with extensive available data were analyzed [25]. In the meta-analysis, all (151) patients of the International MCT8 Deficiency Consortium cohort were included. Data from the International MCT8 Deficiency Consortium cohort study have been updated for the meta-analysis.

Supplementary Table 4. List of selected SNPs in SLC16A2.

		Allele Count	Allele frequency	Homo-zygous	Hemizygous males	Polyphen	SIFT	Rs-number
T>C	S107P	68114	49,8	12658	22733	Benign	Tolerated*	rs6647476
G>A	S82N	92	0.0502	0	28	Benign	Tolerated	rs746783950
C>T	R391C	91	0.0444	1	23	Possibly damaging	Deleterious	rs144755294
C>G	Q212E	80	0.0408	0	26	Benign	Tolerated	rs145061343
G>A	V254I	62	0.0302	1	20	Benign	Tolerated	rs759933264
G>A	A163T	34	0.0173	0	8	Benign	Tolerated	rs201661705
A>G	H575R	21	0.0102	0	8	Probably damaging	Tolerated	rs140303247
G>A	R482Q	19	0.00928	0	11	Benign	Tolerated	rs770854933
G>C	A163P	16	0.00816	0	4	Benign	Tolerated	rs201661705
G>A	A286T	15	0.00733	0	4	Benign	Deleterious	rs376266144
C>T	R482W	15	0.00732	0	6	Possibly damaging	Deleterious	rs760971939
C>T	R300C	13	0.0071	0	7	Probably damaging	Tolerated	rs201194222
C>T	P604L	11	0.006	0	2	Probably damaging	Deleterious	rs749396500
G>A	V447M	11	0.006	0	4	Benign	Deleterious	rs201039304

SNPs were retrieved from the gnomAD database [26], last accessed 2023-06-02. NM_006517.3 and NP_006508.1 were used as reference sequence. SNPs with an allele count >10 and at least two hemizygous male were extracted. *indicates low confidence.

Supplementary Table 5. Gene-based association of SLC16A2 with relevant traits

Trait	group	Chisq(Obs)	$P_{fastBAT}$	Top P_{GWAS}	TopSNP
BMI	all	4.71E+02	0.00013482	1.12E-07	rs5937846
	female	2.88E+02	0.0169823	3.16E-07	rs5937846
	male	357.778	0.00421502	0.000350593	rs67481382
ECG	all	139.5	0.125639	0.000342671	rs60360874
	female	163.516	0.04783	0.000538993	rs60360874
	male	96.2844	0.490546	0.0191054	rs182834831
FT4	all	213.745	9.87E-05	2.74E-05	rs150010878
	female	225.379	7.92E-05	5.51E-05	rs67736575
	male	103.581	0.0196441	0.000640652	rs4892386
SHBG	all	234.592	0.291762	0.0034254	rs5981613
	female	195.502	0.377007	0.000393413	rs112510481
	male	178.801	0.858424	0.0142882	rs113462626
TSH	all	54.4876	0.243617	0.0108529	rs4255295
	female	56.0216	0.225959	0.0106506	rs67736575
	male	41.2806	0.316881	0.00802387	rs143051802
Fluid Intelligence	all	234.592	0.291762	0.0034254	rs5981613
	female	205.22	0.236291	0.0106706	rs6647502
	male	318.861	0.0136554	0.000581449	rs6647506
anteromedial_temporal_area	all	76.9227	0.804863	0.0205235	rs146302399
	female	109.099	0.345575	0.00147346	rs60764888
	male	65.7676	0.870022	0.00921819	rs141167022
dorsolateral_prefrontal_area	all	161.542	0.0748153	0.00473037	rs5937291
	female	117.836	0.265617	0.00326217	rs112356501
	male	101.342	0.393448	0.00322024	rs141402453
dorsolateral_prefrontal_thickness	all	198.51	0.0226888	0.00173186	rs6647502
	female	96.2103	0.494324	0.0181592	rs5981640
	male	137.018	0.124135	0.00190093	X:73740097_AAG_A
dorsomedial_frontal_area	all	118.074	0.298941	0.0122411	rs192423676
	female	120.993	0.240852	0.00849193	rs147611522
	male	82.8742	0.64199	0.0111388	rs192423676
inferior_parietal_area	all	83.9847	0.713464	0.0346536	rs112931797
	female	88.5502	0.596691	0.0263457	rs113462626
	male	81.5113	0.661798	0.0515464	X:73787129_AT_A
inferior_parietal_thickness	all	177.307	0.0448611	0.00248923	rs768466155
	female	74.9944	0.781435	0.0600619	rs145996805
	male	133.543	0.13947	0.00152617	rs111853373
medial_prefrontal_thickness	all	72.718	0.853327	0.00208956	rs5937286
	female	70.2054	0.839626	0.011156	rs62611941
	male	93.2663	0.495075	0.00819922	rs5937286
medial_temporal_thickness	all	130.212	0.205512	0.000978874	rs73216270
	female	100.181	0.444834	1.84E-05	rs149056740
	male	108.273	0.318571	0.00151221	rs73216270
middle_temporal_thickness	all	73.6598	0.842976	0.0425905	rs113462626
	female	83.7017	0.663938	0.0480361	rs142852129
	male	58.0788	0.939256	0.0457526	rs147818495
motor_premotor_area	all	100.874	0.485964	0.0448475	rs5937848
	female	184.333	0.0309357	0.00201267	rs6647506
	male	62.9282	0.898934	0.038892	rs146302399
motor_premotor_SMA_thickness	all	213.797	0.0139541	0.000386466	rs4319247
	female	102.42	0.418289	0.00505675	rs60764888
	male	237.9	0.00464848	0.000567365	rs1926864
occipital_area	all	85.253	0.696237	0.0187725	rs184953713
	female	92.1738	0.547357	0.019607	rs60360874
	male	90.5313	0.532459	0.00826458	rs182024341
occipital_thickness	all	153.77	0.0963246	0.00320667	rs111853373

	female	78.9882	0.728745	0.0112366	rs112931797
	male	148.852	0.0834621	0.00223899	X:73740097_AAG_A
orbitofrontal_area	all	91.5544	0.60962	0.0158559	rs58009583
	female	81.9624	0.688041	0.0474203	rs73216271
	male	90.9574	0.526553	0.0303415	rs140165355
pars_opercularis_area	all	113.758	0.339794	0.00133594	rs5937827
	female	166.156	0.0558293	0.00164152	rs112866421
	male	76.0501	0.740038	0.00455725	rs5937827
posterolateral_temporal_area	all	252.956	0.00409539	0.000336817	rs4255295
	female	175.011	0.0418458	0.00233496	rs5937291
	male	147.789	0.0864893	0.00123739	rs146302399
precuneus_area	all	84.3993	0.70785	0.050557	rs150205528
	female	113.283	0.305146	0.00174938	rs112818030
	male	83.8922	0.627201	0.00168528	rs182024341
superior_parietal_area	all	73.4758	0.845023	0.0094984	rs7056656
	female	91.5363	0.555938	0.00924649	rs111853373
	male	74.3792	0.76317	0.00997637	rs5981637
superior_parietal_thickness	all	212.06	0.0147429	0.000191264	rs58321140
	female	107.234	0.364874	0.00188382	rs149056740
	male	118.795	0.227479	0.00337302	rs58321140
superior_temporal_area	all	176.397	0.0462024	0.00181479	rs5937813
	female	224.448	0.00859523	2.04E-05	rs2094331
	male	109.046	0.310958	0.0083326	rs149343149
temporal_pole_thickness	all	94.447	0.570209	0.0229271	rs73216270
	female	84.0544	0.659038	0.0245128	rs73216248
	male	86.778	0.585518	0.0411264	rs145996805
ventral_frontal_thickness	all	196.362	0.0243011	0.000345265	rs192423676
	female	86.5191	0.624785	0.00254614	rs73216271
	male	230.685	0.00583523	0.000397021	rs140165355
ventromedial_occipital_thickness	all	92.0527	0.602791	0.01057	rs147818495
	female	122.211	0.23185	0.015426	rs112818030
	male	68.1325	0.843258	0.0176302	rs141402453
3rd_ventricle	all	96.0417	0.548798	0.0400728	rs72630714
	female	76.0289	0.768078	0.0272325	rs73216259
	male	83.7958	0.628601	0.0171078	rs17312542
4th_ventricle	all	126.834	0.228487	0.000846581	rs5981640
	female	84.432	0.65379	0.0718297	rs149008214
	male	104.313	0.359881	0.00574284	rs149056740
amygdala	all	152.499	0.100385	0.00201316	rs112931797
	female	85.0713	0.644901	0.0240521	rs60502356
	male	158.723	0.0600135	0.000917463	rs4348648
brainstem	all	93.5797	0.581959	0.0528564	rs6647476
	female	132.799	0.165558	0.00366111	rs12835261
	male	67.1136	0.855069	0.0108333	rs12557933
caudate_nucleus	all	86.96	0.672859	0.0159017	rs12557933
	female	134.205	0.158225	0.00641071	rs12557933
	male	60.1665	0.923349	0.0024073	rs139620417
cerebellum_cortex	all	85.9403	0.686846	0.00273607	rs191421189
	female	87.6272	0.609431	0.0138913	rs5937844
	male	66.6366	0.860454	0.0311104	rs191421189
cerebellum_white_matter	all	97.6488	0.527526	0.00241024	rs191421189
	female	118.365	0.261322	0.00297733	rs62611941
	male	55.7376	0.954456	0.0482366	rs191421189
corpus_callosum_anterior	all	107.111	0.410856	0.0043249	rs112931797
	female	128.523	0.189879	0.00159301	rs149680282
	male	63.6201	0.892233	0.0346695	rs12557933
corpus_callosum_central	all	147.977	0.116252	0.00966149	rs150010878

	female	102.62	0.415972	0.0130329	rs146302399
	male	143.264	0.100661	0.00474538	rs182834831
corpus_callosum_mid_anterior	all	178.447	0.0432375	0.00247299	rs113979039
	female	123.868	0.220085	0.0098929	rs112578685
	male	150.01	0.0802871	0.00905106	rs150010878
corpus_callosum_mid_posterior	all	197.303	0.0235807	0.001151	rs1926864
	female	62.0359	0.919758	0.0771133	rs145391909
	male	206.759	0.012491	0.000438378	rs150010878
corpus_callosum_posterior	all	114.05	0.336903	0.00114965	rs112931797
	female	89.956	0.577402	0.00765716	rs112931797
	male	84.7265	0.615103	0.000241661	rs148492965
CSF	all	93.4813	0.583296	0.0465898	rs140165355
	female	76.854	0.757266	0.0318604	rs184398171
	male	121.865	0.205685	0.0122033	rs767630232
hippocampus	all	78.5912	0.784196	0.0366635	rs112931797
	female	109.777	0.338745	0.018871	rs6647506
	male	70.711	0.811652	0.0141248	rs187456990
inferior_lateral_ventricle	all	162.379	0.0728076	0.000436079	X:73598155_GA_G
	female	104.166	0.398322	0.0163566	rs5981637
	male	106.695	0.334564	0.00131057	X:73598155_GA_G
intracranial_volume	all	89.1514	0.642684	0.0146644	rs4255295
	female	100.506	0.440921	0.0171996	rs73216270
	male	96.3873	0.454113	0.012154	rs7057972
lateral_ventricle	all	117.926	0.30027	0.0139628	rs149008214
	female	62.4653	0.916265	0.0266191	rs60360874
	male	91.4716	0.519466	0.0226407	rs150046039
pallidum	all	183.494	0.0367372	0.00145957	rs141402453
	female	121.937	0.233846	0.00951454	rs374584740
	male	141.054	0.108412	0.00156817	rs141402453
thalamus	all	101.01	0.484245	0.0202101	rs376674720
	female	130.964	0.175614	0.00277647	rs12559757
	male	92.0706	0.511263	0.0111331	rs73216248
ventral_diencephalon	all	169.257	0.0582299	0.000722274	rs141402453
	female	149.743	0.095452	0.0021342	rs191435310
	male	120.981	0.211751	0.0018776	rs12557933

SLC16A2 gene body was defined as extending from 73641327-73753767 in chromosome X. Significance of the gene-based test is compared with the regional tophit in the univariate analysis.

Supplementary Table 6. Characterization of missense variants identified in MCT8 deficiency

Affected residue	Variant	Loss of transport capacity	Protein expression	Cell surface expression	Critical group	Critical property	Conservation score
**Ile188**	I188N				3	Branched AA	-1,002
**His192**	H192R				1	H-bond/pi-pi to T4	-0,427
	*H192P**				1		
**Ser194**	S194F				-	NA	-0,947
**Gly196**	G196V				1	Small size/flexibility	-0,656
	G196E				1		
**Leu203**	L203P					Branched AA	1,023
**Gly221**	G221R				-	NA	-0,648
**Ala224**	A224T				NA	Size	-0,384
	A224V				NA		
	*A224E**				NA		
**Met227**	M227R				-	NA	-0,882
**Ser232**	S232F				-	NA	-0,951
**Val235**	V235M				-	NA	-0,763
**Thr239**	T239P				-	NA	-0,860
**Gly251**	G251E				7	Size, flexibility	-0,799
**Ala252**	A252P				NA	NA	-0,786
**Arg271**	R271H				3	Size/charge?	-0,644
	R271S				3		
**Gly276**	G276R				-	NA	-0,800
**Gly282**	G282C				3	Small, flexibility	-0,794
	G282D				3		
**Cys283**	C283Y				3	Small, flexibility	-0,465
**Pro289**	P289L				6	NA	-0,843
**Ser290**	S290F				-	NA	-0,773
**Leu291**	L291R				**6**	Branched AA	-0,874

Table continues on next page

Supplementary Table 6. Characterization of missense variants identified in MCT8 deficiency (continued)

Affected residue	Variant	Loss of transport capacity	Protein expression	Cell surface expression	Critical group	Critical property	Conservation score
**Phe298**	F298L				**6**	Aromatic group	-0,871
**Leu304**	L304P				**4**	Branched AA	-0,656
**Gly307**	G307C				4	Small, flexibility	-0,667
**Gly312**	G312R				-	NA	0,718
**Ser313**	S313R				-	NA	-0,821
**Pro321**	P321L				-	NA	-0,596
**Tyr354**	Y354C				7	Aromatic	-0,545
**Trp398**	W398R				-	NA	-0,948
**Gly401**	G401R				-	NA	-0,638
	G401E				-		
**Leu433**	L433H				2	Aliphatic, branched AA	-0,665
**Leu434**	L434W				1	Aliphatic, branched AA	-0,768
**Ser441**	S441L				2	Hydroxyl-group	-1,008
	S441P				2		
**Arg445**	R445C				1	Positive charge	-0,994
	R445S				1		
	R445L				1		
**Gly449**	G449D				4	Flexibility	-0,953
**Asp453**	D453N				4	Size and + charge	-0,994
	D453V				4		
**Leu469**	L469P				-	NA	0,593
**Leu471**	L471P				2	NA	-0,459
	L471R				2		
**Cys491**	C491Y				-	NA	-0,519
**Leu492**	L492P				-	NA	-0,123
**Leu494**	L494P				2	NA	-0,706

Table continues on next page

Supplementary Table 6. Characterization of missense variants identified in MCT8 deficiency (continued)

Affected residue	Variant	Loss of transport capacity	Protein expression	Cell surface expression	Critical group	Critical property	Conservation score
**Gly495**	G495A				3	Size, flexibility	-0,953
**Asp498**	D498N				1	- charge	-0,838
**Gly499**	G499D				1	Size, flexibility	-0,808
**Leu512**	L512P				-	NA	-0,764
**Ser519**	S519L				5	Hydroxyl-group	-1,008
**Gly527**	G527S				5	Flexibility	-0,953
**Gly536**	G536R				7	Flexibility	-0,953
**Pro537**	P537L				-	NA	-0,848
**Leu534**	L543P				-	NA	-0,725
**Ala553**	A553D				NA	NA	-0,769
**Gly558**	G558D				-	NA	-0,952
**Gly564**	G564E				-	NA	-0,952
	G564R				-		
**Leu568**	L568P				-	NA	-0,882

Comprehensive overview of pathogenic mechanisms of studied missense variants and their conservation scores (hsMCT8 vs all MCT8). Color codes (see scale below) indicate the loss of transport capacity, the protein expression and the cell surface expression of individual variants in vitro, compared to mock-transfected cells (set to 0%) and cells transfected with WT MCT8 (set to 100%). Abbreviations: AA, amino acid; NA, not applicable. * denotes variant reported in ClinVar.

0% 50% 100%

Supplementary Table 7. Sequences for conservation analyses.

Sequence	Protein	Organism
<u>**hsMCT8 *versus* all MCT8**</u>		
A0A0P7W0K4	Monocarboxylate transporter 8-like	Scleropages formosus (Asian bonytongue)
A0A4W5P5E4	Uncharacterized protein	Hucho hucho (huchen)
A0A4W4F7A7	MFS domain-containing protein	Electrophorus electricus (Electric eel)
A0A3P8UY50	Monocarboxylate transporter 8	Cynoglossus semilaevis (Tongue sole)
A0A2I4B229	monocarboxylate transporter 8	Austrofundulus limnaeus
A0A3Q3B655	Solute carrier family 16 member 2	Kryptolebias marmoratus (Mangrove killifish)
A0A3Q2Y2S7	Monocarboxylate transporter 8-like	Hippocampus comes (Tiger tail seahorse)
A0A3B3ZTW0	MFS domain-containing protein	Periophthalmus magnuspinnatus
A0A672Z3Q4	Uncharacterized protein	Sphaeramia orbicularis (orbiculate cardinalfish)
A0A3B3HBM5	Monocarboxylate transporter 8	Oryzias latipes (Japanese rice fish)
H2LCL0	Monocarboxylate transporter 8	Oryzias latipes (Japanese rice fish)
A0A3Q1IHC1	Solute carrier family 16 member 2	Anabas testudineus (Climbing perch)
A0A6P7NLU8	monocarboxylate transporter 8	Betta splendens (Siamese fighting fish)
A0A1A7WM54	Solute carrier family 16 (Monocarboxylic acid transporters), member 2	Iconisemion striatum
A0A3Q3KV54	Solute carrier family 16 member 2	Mastacembelus armatus (zig-zag eel)
A0A3Q2CVZ6	Monocarboxylate transporter 8-like	Cyprinodon variegatus (Sheepshead minnow)
A0A3P9PQK7	Solute carrier family 16 member 2	Poecilia reticulata (Guppy)
A0A3Q2TZE8	Solute carrier family 16 member 2	Fundulus heteroclitus (Killifish)
A0A3P8SS10	Solute carrier family 16 member 2	Amphiprion percula (Orange clownfish)
A0A3P8SSH6	Solute carrier family 16 member 2	Amphiprion percula (Orange clownfish)
A0A3Q0T7V0	Solute carrier family 16 member 2	Amphilophus citrinellus (Midas cichlid)
A0A668TT83	Solute carrier family 16 member 2	Oreochromis aureus (Israeli tilapia)
A0A668TQJ4	Solute carrier family 16 member 2	Oreochromis aureus (Israeli tilapia)
A0A667X4F9	Monocarboxylate transporter 8-like	Myripristis murdjan (pinecone soldierfish)
A0A3Q3NM92	Monocarboxylate transporter 8-like	Labrus bergylta (ballan wrasse)
G3Q0F3	Solute carrier family 16 member 2	Gasterosteus aculeatus (Three-spined stickleback)
A0A671WG38	Monocarboxylate transporter 8-like	Sparus aurata (Gilthead sea bream)
A0A6P8TU20	LOW QUALITY PROTEIN: monocarboxylate transporter 8	Gymnodraco acuticeps (Antarctic dragonfish)
A0A5C6MLW9	Monocarboxylate transporter 8	Takifugu flavidus (sansaifugu)
A0A6P7J563	monocarboxylate transporter 8-like	Parambassis ranga (Indian glassy fish)
A0A6G1PUE8	Monocarboxylate transporter 8	Channa argus (northern snakehead)
A0A6A4T138	Uncharacterized protein	Scophthalmus maximus (Turbot)
A0A3Q3JGK9	Monocarboxylate transporter 8-like	Monopterus albus (Swamp eel)
A0A3B4UVY0	Solute carrier family 16 member 2	Seriola dumerili (Greater amberjack)
A0A665XG26	Monocarboxylate transporter 8-like	Echeneis naucrates (Live sharksucker)
A0A5N5MGB2	MFS domain-containing protein	Pangasianodon hypophthalmus (Striped catfish)
A0A6P3W4V1	monocarboxylate transporter 8	Clupea harengus (Atlantic herring)
A0A3P8XJ90	Solute carrier family 16 member 2	Esox lucius (Northern pike)
A0A673WGL6	Monocarboxylate transporter 8-like	Salmo trutta (Brown trout)
A0A5A9NJ52	Monocarboxylate transporter 8	Triplophysa tibetana
A0A553QVJ6	Uncharacterized protein	Danionella translucida
A0A673K5T1	Monocarboxylate transporter 8-like	Sinocyclocheilus rhinocerous
A0A0G2L3Z8	MFS domain-containing protein	Danio rerio (Zebrafish)
A0A0G2KDP5	MFS domain-containing protein	Danio rerio (Zebrafish)
A0A6J2US61	monocarboxylate transporter 8-like	Chanos chanos (Milkfish)
A0A4W4EBJ0	MFS domain-containing protein	Electrophorus electricus (Electric eel)
A0A4W4EBH4	MFS domain-containing protein	Electrophorus electricus (Electric eel)
A0A3B4CWB3	Solute carrier family 16 member 2	Pygocentrus nattereri (Red-bellied piranha)
W5K910	MFS domain-containing protein	Astyanax mexicanus (Blind cave fish)

A0A3B1K9K8	MFS domain-containing protein	Astyanax mexicanus (Blind cave fish)
W5N858	Solute carrier family 16 member 2	Lepisosteus oculatus (Spotted gar)
A0A3B3TCK7	Monocarboxylate transporter 8-like	Paramormyrops kingsleyae
A0A0P7WV38	Monocarboxylate transporter 8-like	Scleropages formosus (Asian bonytongue)
A0A4W3JKU4	MFS domain-containing protein	Callorhinchus milii (Ghost shark)
A0A401P3C6	Uncharacterized protein	Scyliorhinus torazame (Cloudy catshark)
F7BHA6	Solute carrier family 16 member 2	Ornithorhynchus anatinus (Duckbill platypus)
A0A7N4NRH6	Solute carrier family 16 member 2	Sarcophilus harrisii (Tasmanian devil)
A0A5F4VZX7	Solute carrier family 16 member 2	Callithrix jacchus (White-tufted-ear marmoset)
M3YA43	Solute carrier family 16 member 2	Mustela putorius furo (European domestic ferret)
A0A5E4CB28	Monocarboxylate transporter 8	Marmota monax (Woodchuck)
G3SUL4	Solute carrier family 16 member 2	Loxodonta africana (African elephant)
F7D7T1	Solute carrier family 16, member 2 (thyroid hormone transporter)	Xenopus tropicalis (Western clawed frog)
A0A6P8RDI2	monocarboxylate transporter 8	Geotrypetes seraphini (Gaboon caecilian)
A0A6P7YKZ6	monocarboxylate transporter 8	Microcaecilia unicolor
A0A099Z5H0	Monocarboxylate transporter 8	Tinamus guttatus (White-throated tinamou)
U3K8E3	Solute carrier family 16 member 2	Ficedula albicollis (Collared flycatcher)
A0A2P4T773	Uncharacterized protein	Bambusicola thoracicus (Chinese bamboo-partridge)
K7G832	Uncharacterized protein	Pelodiscus sinensis (Chinese softshell turtle)
A0A7M4EIQ3	Solute carrier family 16 member 2	Crocodylus porosus (Saltwater crocodile)
A0A6I9Z381	monocarboxylate transporter 8	Thamnophis sirtalis
A0A670YSN4	Solute carrier family 16 member 2	Pseudonaja textilis (Eastern brown snake)
A0A670K2C8	Uncharacterized protein	Podarcis muralis (Wall lizard)
A0A6J0T1R0	monocarboxylate transporter 8	Pogona vitticeps (central bearded dragon)
**hsMCT8 *versus* mammal MCT8**		
F7BHA6	Solute carrier family 16 member 2	Ornithorhynchus anatinus (Duckbill platypus)
A0A7N4NRH6	Solute carrier family 16 member 2	Sarcophilus harrisii (Tasmanian devil)
M3YA43	Solute carrier family 16 member 2	Mustela putorius furo (European domestic ferret)
A0A5E4CB28	Monocarboxylate transporter 8	Marmota monax (Woodchuck)
A0A2K5L6Y7	Solute carrier family 16 member 2	Cercocebus atys (Sooty mangabey)
A0A6J3QTC0	monocarboxylate transporter 8 isoform X2	Tursiops truncatus (Atlantic bottle-nosed dolphin)
F6Z232	Solute carrier family 16 member 2	Macaca mulatta (Rhesus macaque)
A0A2K6GCU4	Solute carrier family 16 member 2	Propithecus coquereli (Coquerel's sifaka)
A0A2Y9GS85	monocarboxylate transporter 8	Neomonachus schauinslandi (Hawaiian monk seal)
A0A7J8EM28	Solute carrier family 16 member 2	Rousettus aegyptiacus (Egyptian rousette)
F1MM56	Solute carrier family 16 member 2	Bos taurus (Bovine)
A0A384D2Q5	monocarboxylate transporter 8	Ursus maritimus (Polar bear)
A0A452QMZ6	Solute carrier family 16 member 2	Ursus americanus (American black bear)
G1PA04	Solute carrier family 16 member 2	Myotis lucifugus (Little brown bat)
A0A3Q7V1Q5	monocarboxylate transporter 8	Ursus arctos horribilis
A0A5N4C2S6	Monocarboxylate transporter 8	Camelus dromedarius (Dromedary)
A0A341D559	monocarboxylate transporter 8	Neophocaena asiaeorientalis asiaeorientalis (Yangtze finless porpoise)
G1SEY4	Solute carrier family 16 member 2	Oryctolagus cuniculus (Rabbit)
A0A2K5TN74	Solute carrier family 16 member 2	Macaca fascicularis (Crab-eating macaque)
A0A6J0XQP9	monocarboxylate transporter 8	Odocoileus virginianus texanus
I3LJG4	Solute carrier family 16 member 2	Sus scrofa (Pig)
A0A6P6IS12	monocarboxylate transporter 8	Puma concolor (Mountain lion)
A0A4W2IJN1	Solute carrier family 16 member 2	Bos indicus x Bos taurus (Hybrid cattle)

F1PS05	Solute carrier family 16 member 2	Canis lupus familiaris (Dog)
L5KBV3	Monocarboxylate transporter 8	Pteropus alecto (Black flying fox)
A0A671DPB9	Solute carrier family 16 member 2	Rhinolophus ferrumequinum (Greater horseshoe bat)
A0A7J7S6Q5	Solute carrier family 16 member 2	Pipistrellus kuhlii (Kuhl's pipistrelle)
S9X8J6	Solute carrier family 16, member 2-like protein	Camelus ferus (Wild bactrian camel)
A0A6J2MIS3	Solute carrier family 16 member 2	Phyllostomus discolor (pale spear-nosed bat)
A0A6J1YYD8	monocarboxylate transporter 8	Acinonyx jubatus (Cheetah)
A0A5N3XEM3	MFS domain-containing protein	Muntiacus reevesi (Reeves' muntjac)
A0A2Y9T6Q4	monocarboxylate transporter 8	Physeter macrocephalus (Sperm whale)
G3TWP7	Solute carrier family 16 member 2	Loxodonta africana (African elephant)
A0A6J3FPH5	monocarboxylate transporter 8	Sapajus apella (Brown-capped capuchin)
A0A6G1AXP3	MOT8 protein	Crocuta crocuta (Spotted hyena)
A0A6P6DVG8	monocarboxylate transporter 8	Octodon degus (Degu)
A0A1S3G690	monocarboxylate transporter 8	Dipodomys ordii (Ord's kangaroo rat)
A0A6D2VU78	SLC16A2 isoform 1	Pan troglodytes (Chimpanzee)
M3W8X5	Solute carrier family 16 member 2	Felis catus (Cat)
G1QJX7	MFS domain-containing protein	Nomascus leucogenys (Northern white-cheeked gibbon)
L8IYB4	Monocarboxylate transporter 8	Bos mutus (wild yak)
A0A3Q7SNF5	monocarboxylate transporter 8	Vulpes vulpes (Red fox)
I3MXZ7	Solute carrier family 16 member 2	Ictidomys tridecemlineatus (Thirteen-lined ground squirrel)
A0A340WRZ0	monocarboxylate transporter 8	Lipotes vexillifer (Yangtze river dolphin)
A0A667IAV3	Solute carrier family 16 member 2	Lynx canadensis (Canada lynx)
A0A673VJH2	Solute carrier family 16 member 2	Suricata suricatta (Meerkat)
A0A384AMW9	monocarboxylate transporter 8	Balaenoptera acutorostrata scammoni (North Pacific minke whale)
A0A7J8J7Z7	Solute carrier family 16 member 2	Molossus molossus (Pallas' mastiff bat)
G1L7X1	Solute carrier family 16 member 2	Ailuropoda melanoleuca (Giant panda)
A0A485P6Y7	Solute carrier family member 2	Lynx pardinus (Iberian lynx)
G3R214	Solute carrier family 16 member 2	Gorilla gorilla gorilla (Western lowland gorilla)
L8Y6I2	Monocarboxylate transporter 8	Tupaia chinensis (Chinese tree shrew)
A0A3L7H2J0	SLC16A2	Cricetulus griseus (Chinese hamster)
A0A6P3RA03	monocarboxylate transporter 8	Pteropus vampyrus (Large flying fox)
A0A2Y9L287	monocarboxylate transporter 8	Enhydra lutris kenyoni
A0A0D9RF61	MFS domain-containing protein	Chlorocebus sabaeus (Green monkey)
A0A2Y9NZT6	monocarboxylate transporter 8 isoform X1	Delphinapterus leucas (Beluga whale)
A0A4U1ES36	MFS domain-containing protein	Monodon monoceros (Narwhal)
A0A0P6J2C4	Monocarboxylate transporter 8	Heterocephalus glaber (Naked mole rat)
U3EU28	Monocarboxylate transporter 8	Callithrix jacchus (White-tufted-ear marmoset)
A0A6P7DIT7	monocarboxylate transporter 8	Ovis aries (Sheep)
A0A2Y9DS84	monocarboxylate transporter 8	Trichechus manatus latirostris (Florida manatee)
A0A6P5B4X0	monocarboxylate transporter 8	Bos indicus (Zebu)
H0WPV6	Solute carrier family 16 member 2	Otolemur garnettii (Small-eared galago)
A0A2K6LPV7	MFS domain-containing protein	Rhinopithecus bieti (Black snub-nosed monkey)
A0A2K5Y5Z0	Solute carrier family 16 member 2	Mandrillus leucophaeus (Drill)
A0A7J7YFA7	Solute carrier family 16 member 2	Myotis myotis (Greater mouse-eared bat)
A0A2U3WNQ8	monocarboxylate transporter 8	Odobenus rosmarus divergens (Pacific walrus)
A0A2K6C860	Solute carrier family 16 member 2	Macaca nemestrina (Pig-tailed macaque)
A0A1S2ZXF6	monocarboxylate transporter 8	Erinaceus europaeus (Western European hedgehog)
A0A452ET44	Solute carrier family 16 member 2	Capra hircus (Goat)
A0A3Q7Q4V7	monocarboxylate transporter 8	Callorhinus ursinus (Northern fur seal)
A0A6I9IRS5	monocarboxylate transporter 8	Vicugna pacos (Alpaca)

A0A6J2E758	monocarboxylate transporter 8 isoform X1	Zalophus californianus (California sealion)
A0A096MQQ4	Solute carrier family 16 member 2	Papio anubis (Olive baboon)
A0A6P4V9E0	monocarboxylate transporter 8	Panthera pardus (Leopard)
A0A2K5RM24	Solute carrier family 16 member 2	Cebus imitator (Panamanian white-faced capuchin)
A0A643BKS0	MFS domain-containing protein	Balaenoptera physalus (Fin whale)
L5MG83	Monocarboxylate transporter 8	Myotis davidii (David's myotis)
A0A2U3XQR2	monocarboxylate transporter 8	Leptonychotes weddellii (Weddell seal)
U6CY06	Monocarboxylate transporter 8	Neovison vison (American mink)
A0A5N3V609	MFS domain-containing protein	Muntiacus muntjak (Barking deer)
A0A2K5KFT7	Solute carrier family 16 member 2	Colobus angolensis palliatus (Peters' Angolan colobus)
A0A091EQ24	Monocarboxylate transporter 8	Fukomys damarensis (Damaraland mole rat)
S7MFP5	Monocarboxylate transporter 8	Myotis brandtii (Brandt's bat)
A0A0A1ECF2	Solute carrier family 16 member 2	Fukomys anselli (Ansell's mole-rat)
A0A2K5E4I2	Solute carrier family 16 member 2	Aotus nancymaae (Ma's night monkey)
A0A3Q2GVV8	Solute carrier family 16 member 2	Equus caballus (Horse)
A0A1U7SMK5	monocarboxylate transporter 8	Carlito syrichta (Philippine tarsier)
A0A2K6SUL6	Solute carrier family 16 member 2	Saimiri boliviensis boliviensis (Bolivian squirrel monkey)
A0A6I9KHK3	LOW QUALITY PROTEIN: monocarboxylate transporter 8-like	Chrysochloris asiatica (Cape golden mole)
G3V9C2	Solute carrier family 16 (Monocarboxylic acid transporters), member 2, isoform CRA_b	Rattus norvegicus (Rat)
Q05BA2	Slc16a2 protein	Mus musculus (Mouse)
A0A1U7R399	monocarboxylate transporter 8	Mesocricetus auratus (Golden hamster)
K9ITE4	Putative monocarboxylate transporter 8	Desmodus rotundus (Vampire bat)
A0A6I9MCG8	Solute carrier family 16 member 2	Peromyscus maniculatus bairdii (Prairie deer mouse)
H0UU03	Solute carrier family 16 member 2	Cavia porcellus (Guinea pig)
A0A6P5P250	monocarboxylate transporter 8	Mus caroli (Ryukyu mouse)
H2PW21	Solute carrier family 16 member 2	Pongo abelii (Sumatran orangutan)
A0A2R9BXU3	Solute carrier family 16 member 2	Pan paniscus (Pygmy chimpanzee)
Q3ZLS0	Solute carrier 16 A 2	Macropus eugenii (Tammar wallaby)
H9H6J2	MFS domain-containing protein	Monodelphis domestica (Gray short-tailed opossum)
A0A6P5IDU6	monocarboxylate transporter 8	Phascolarctos cinereus (Koala)
A0A4X2KC40	Solute carrier family 16 member 2	Vombatus ursinus (Common wombat)
**hsMCT8 *versus* functionally evaluated MCT8**		
NP_033223.2	Monocarboxylate transporter 8	Mus musculus (Mouse)
NP_671749.2	Monocarboxylate transporter 8	Rattus norvegicus (Rat)
NP_001245159.1	Monocarboxylate transporter 8	Danio rerio (Zebrafish)
AMQ48645.1	Monocarboxylate transporter 8	Gallus gallus (Chicken)
A0A1L8F2I6	Monocarboxylate transporter 8	Xenopus laevis (African clawed frog)
**hsMCT8 *versus* hsMCTs**		
NM_003051.4	Monocarboxylate transporter 1	Homo sapiens (Human)
NM_001270623.2	Monocarboxylate transporter 2	Homo sapiens (Human)
NM_013356.3	Monocarboxylate transporter 3	Homo sapiens (Human)
NM_004207.4	Monocarboxylate transporter 4	Homo sapiens (Human)
NM_004696.3	Monocarboxylate transporter 5	Homo sapiens (Human)
NM_004695.4	Monocarboxylate transporter 6	Homo sapiens (Human)
NM_004694.5	Monocarboxylate transporter 7	Homo sapiens (Human)

NM_194298.3	Monocarboxylate transporter 9	Homo sapiens (Human)
NM_018593.5	Monocarboxylate transporter 10	Homo sapiens (Human)
NM_001370549.1	Monocarboxylate transporter 11	Homo sapiens (Human)
NM_213606.4	Monocarboxylate transporter 12	Homo sapiens (Human)
NM_201566.3	Monocarboxylate transporter 13	Homo sapiens (Human)
NM_152527.5	Monocarboxylate transporter 14	Homo sapiens (Human)

Sequences used for conservation analyses.

Supplementary Table 8. Antibody Table.

Target protein/ antigen	Antigen sequence (if known)	Name of AB	Species raised (P or M)	Manufacturer (and catalogue number)	Dilution used for WB	Dilution used for ICH	RRID	Ref
hsMCT8	AA 52-155	MCT8	Rabbit (P)	ATLAS (HPA003353)	1:2,000	1:1,000	AB_1079343	[27]
GAPDH		GAPDH	Mouse (M)	Millipore (Mab 374)	1:20,000		AB_2107445	[28]
ZO1		ZO1	Mouse (M)	Thermo Fisher (33-9100)		1:500	AB_2533147	[29]
Rabbit IgG		IRDye800	Goat	LI-COR (926-32211)	1:20,000		AB_621843	[30]
Mouse IgG		IRDye680	Goat	LI-COR (926-68020)	1:20,000		AB_10706161	[31]
Rabbit IgG		Alexa 488	Goat	Thermo Fisher (A11008)		1:1,000	AB_143165	[32]
Mouse IgG		Alexa 633	Goat	Thermo Fisher (A21050)		1:1,000	AB_2535718	[33]

AB: antibody; GAPDH: glyceraldehyde-3-phosphate dehydrogenase; ZO-1: zona occludens 1;P: polyclonal antibody; M: monoclonal antibody; WB: Western Blot ; ICH: immunohistochemistry

Supplementary Figures

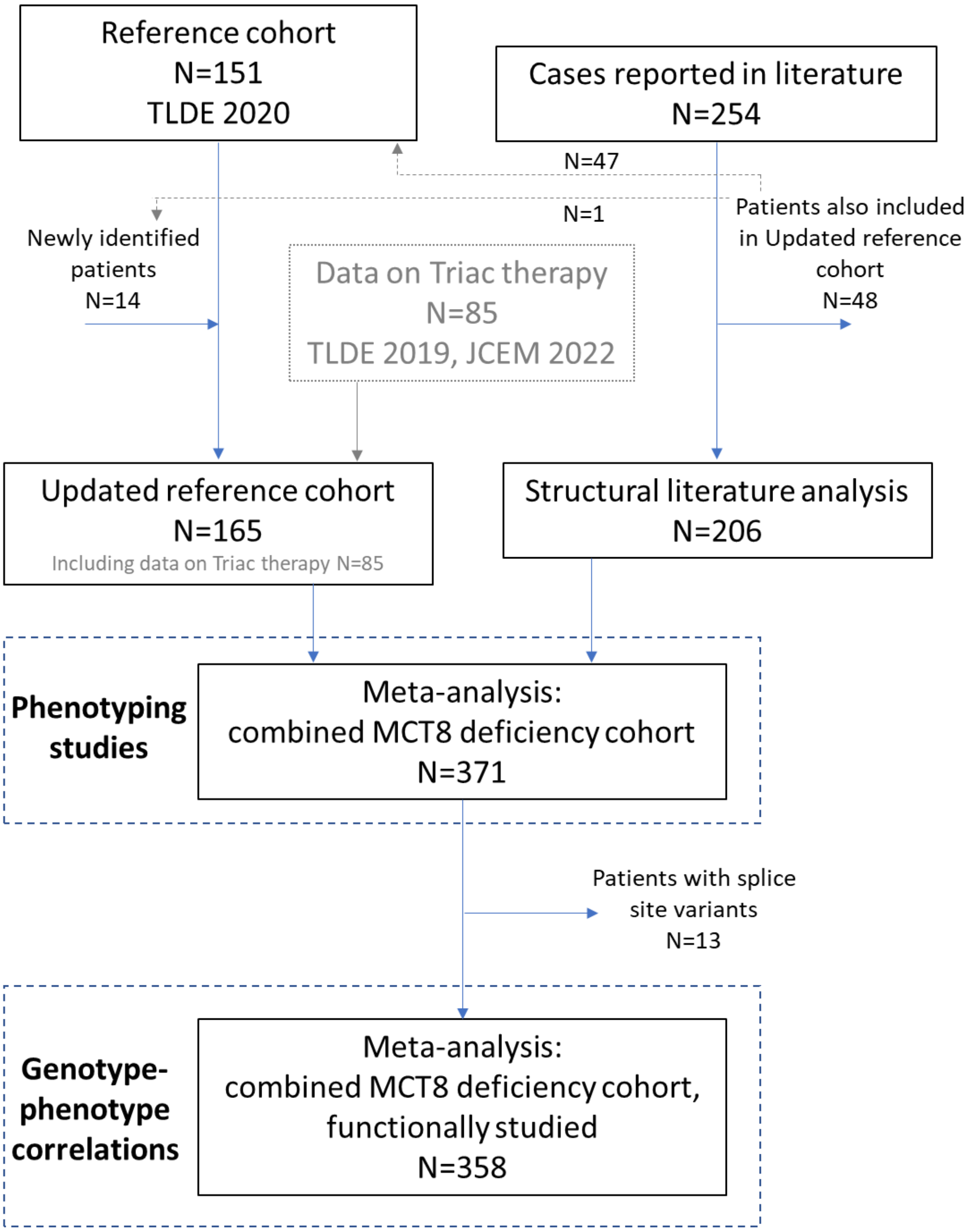

Supplementary Fig. 1. Flow diagram of the study. TLDE 2019 refers to [34] TLDE 2020 refers to [25] and JCEM 2022 refers to [35].

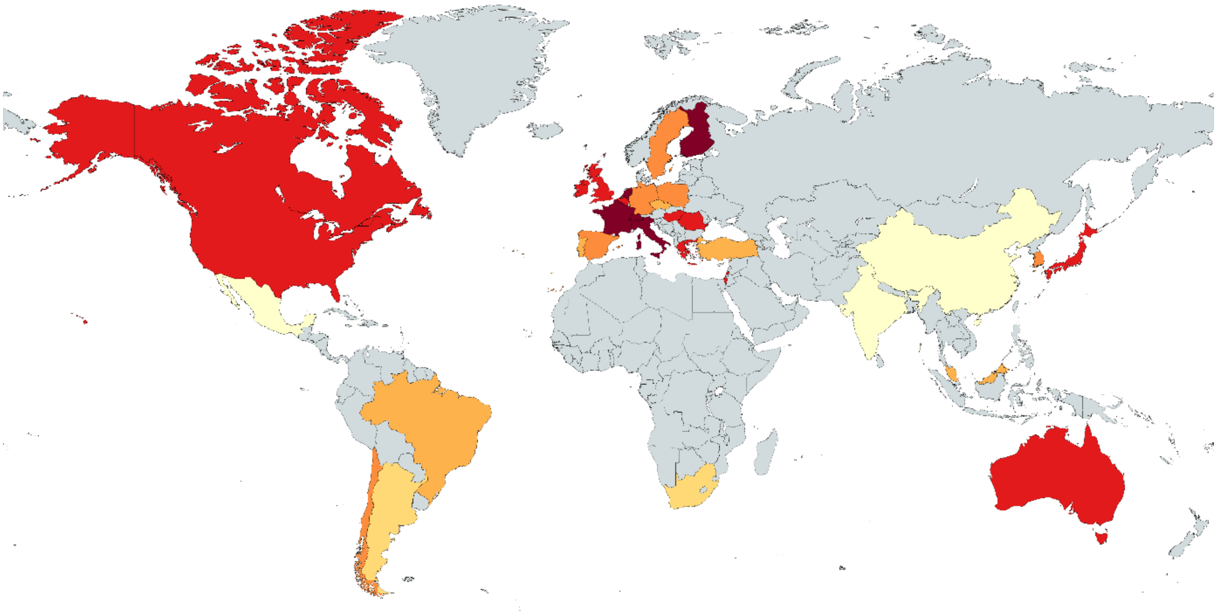

Supplementary Fig. 2. Distribution of patients included in the meta-analysis. Light yellow indicates <0.1 patients per 10 million residents; yellow indicates 0.1 – 0.5 patients per 10 million residents; light orange indicates 0.5 – 1 patients per 10 million residents; orange indicates 1 – 2 patients per 10 million residents; red indicates 2 -5 patients per 10 million residents; dark red indicates >5 patients per 10 million residents [36, 37].

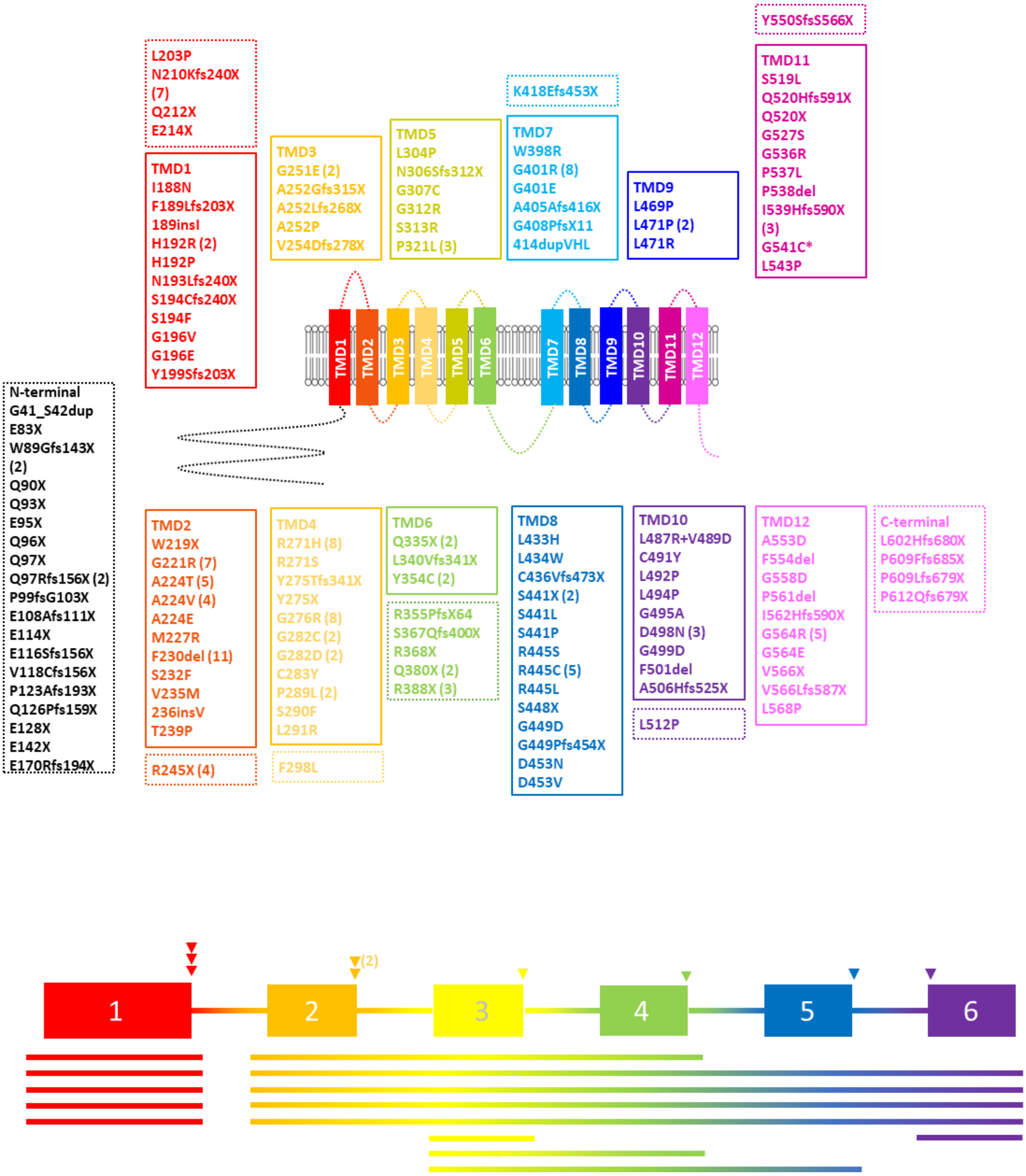

Supplementary Fig. 3. Overview of unique genetic variants identified in the *SLC16A2* gene encoding MCT8 and investigated in this study. (A) Missense, nonsense and frameshift variants and (B) deletions (lines) and splicing variants (arrow heads). Mutations that occurred >1 in independent families are indicated with a frequency between brackets. Dashed boxes represent variants in the intracellular or extracellular domains. * also results in a splicing variant.

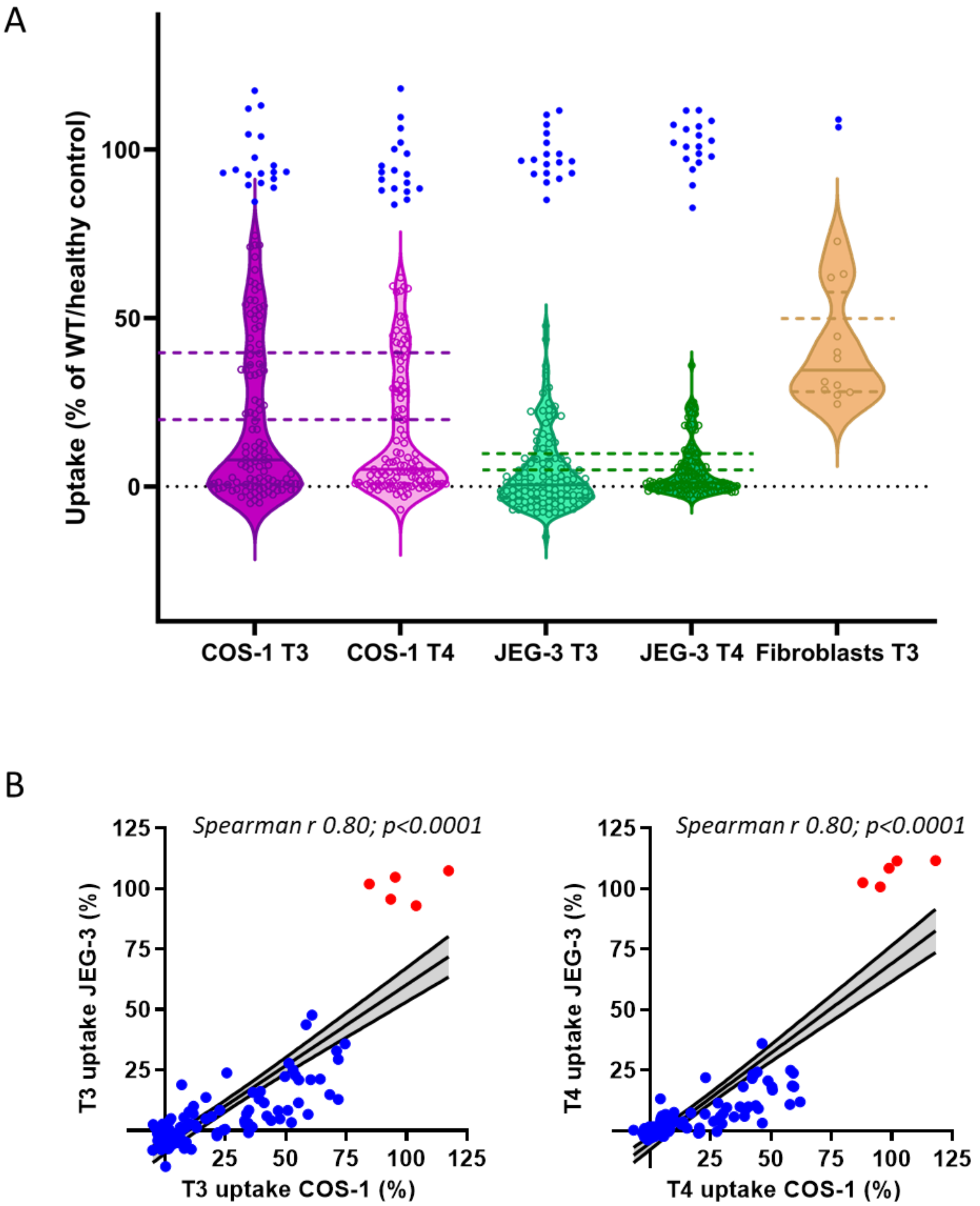

Supplementary Fig. 4. (A) Distribution of T3 and T4 transport capacity of MCT8 variants shown as residual transport function (100% = WT MCT8, or healthy control fibroblasts) in different cells. All variants were introduced in an MCT8 expression vector and functionally evaluated, except for variants that lead to a premature truncation before transmembrane domain 12 as such truncations have been

previously shown to be fully inactive (1, 2). WT and mutant MCT8 were expressed in COS-1 and JEG-3 (lacking endogenous MCT8 expression) cells. After incubation with 1 nM T3 or 1 nM T4 for 30 minutes, cellular uptake of radio-labelled hormone was measured. Uptake values were corrected for mock-transfected cells, and for protein content in the lysates (for fibroblast studies). Variation in T3 uptake across variants was largest in COS-1 cells, leaving this model suitable to perform genotype-phenotype analyses. Variation in T4 uptake across variants was smallest in JEG-3 cells, leaving this model suitable as a first screen to distinguish benign from pathogenic variants. Dashed lines represent cut-off values for different LoF classes. For COS-1 cells: <20% (severe LoF), 20-40% (moderate LoF), >40% (mild LoF). For JEG-3 cells: <5% (severe LoF), 5-10% (moderate LoF), >10% (mild LoF). For fibroblasts: <50% (severe LoF), >50% (mild LoF). Blue dots represent data from benign non-synonymous missense variants. (B) Correlation plots of transport capacity of T3 (left panel) and T4 (right panel) between COS-1 and JEG-3 cells expressing WT or mutant MCT8. Blue dots represent data from patient-derived mutations; red dots represent data from benign non-synonymous missense variants. Exact P values are provided in Supplementary Data 1. Source data are provided as a Source Data file.

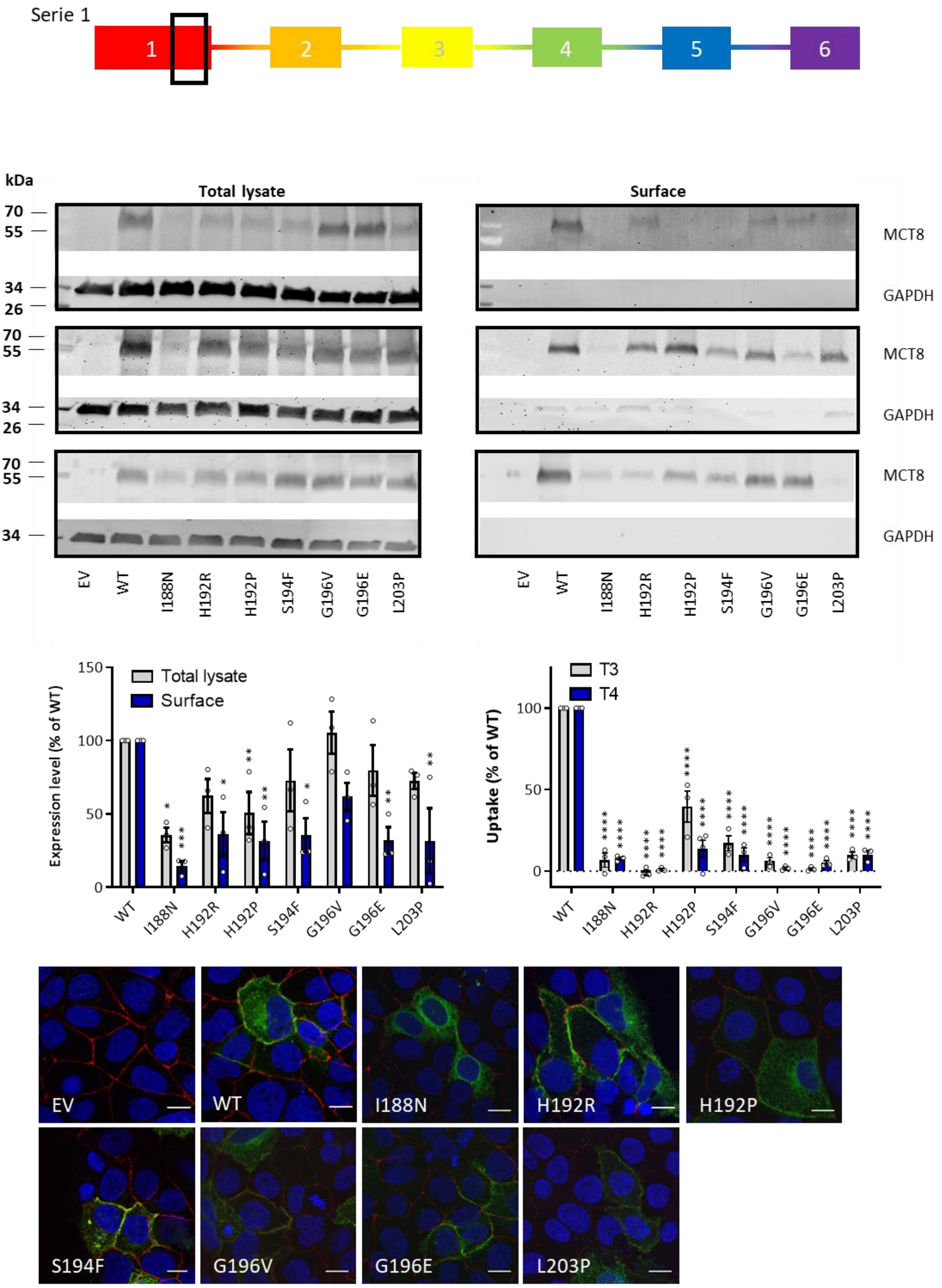
Serie 1
1
2
3
4
5
6
kDa
Total lysate
Surface
70
55
34
26
MCT8
GAPDH
EV
WT
I188N
H192R
H192P
S194F
G196V
G196E
L203P
Expression level (% of WT)
Total lysate
Surface
150
100
50
0
Uptake (% of WT)
T3
T4

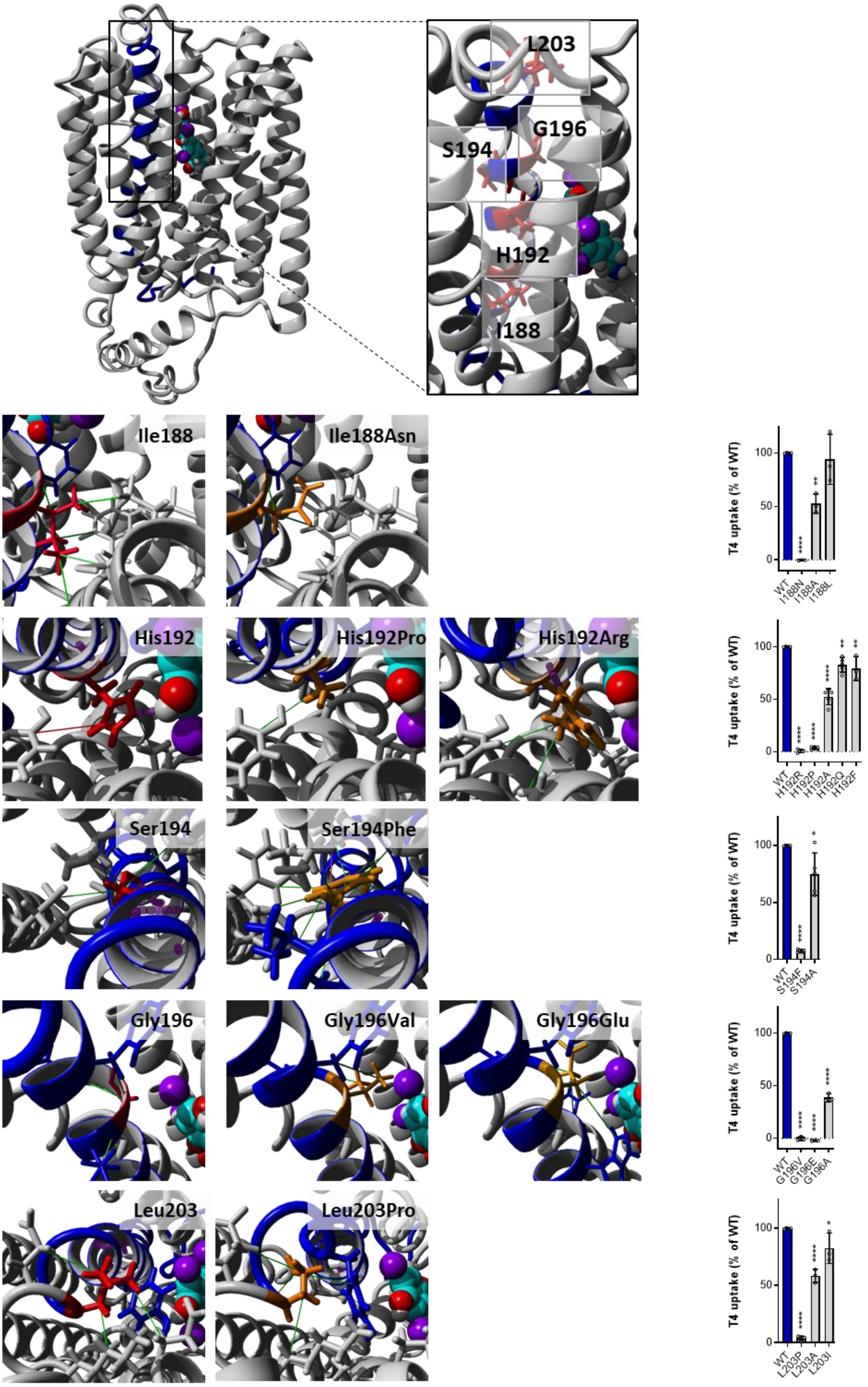

L203
G196
S194
H192
I188
Ile188
Ile188Asn
His192
His192Pro
His192Arg
Ser194
Ser194Phe
Gly196
Gly196Val
Gly196Glu
Leu203
Leu203Pro
T4 uptake (% of WT)
WT
I188N
I188A
I188L
H192R
H192P
H192A
H192Q
H192F
S194F
S194A
G196V
G196E
G196A
L203P
L203A
L203I

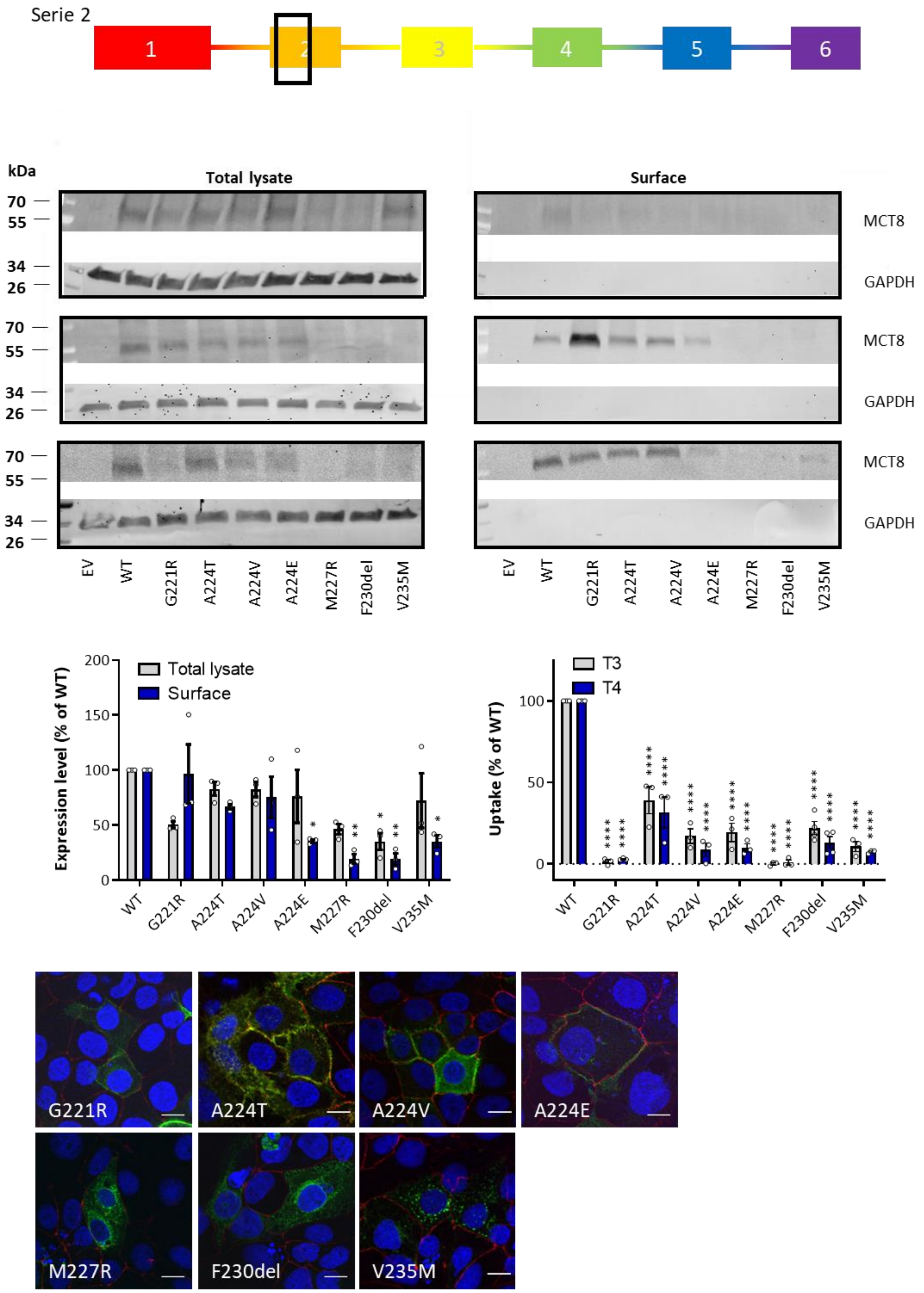
Serie 2
1
2
3
4
5
6
kDa
Total lysate
Surface
70
55
34
26
MCT8
GAPDH
EV
WT
G221R
A224T
A224V
A224E
M227R
F230del
V235M
Expression level (% of WT)
Total lysate
Surface
200
150
100
50
0
Uptake (% of WT)
T3
T4
G221R
A224T
A224V
A224E
M227R
F230del
V235M

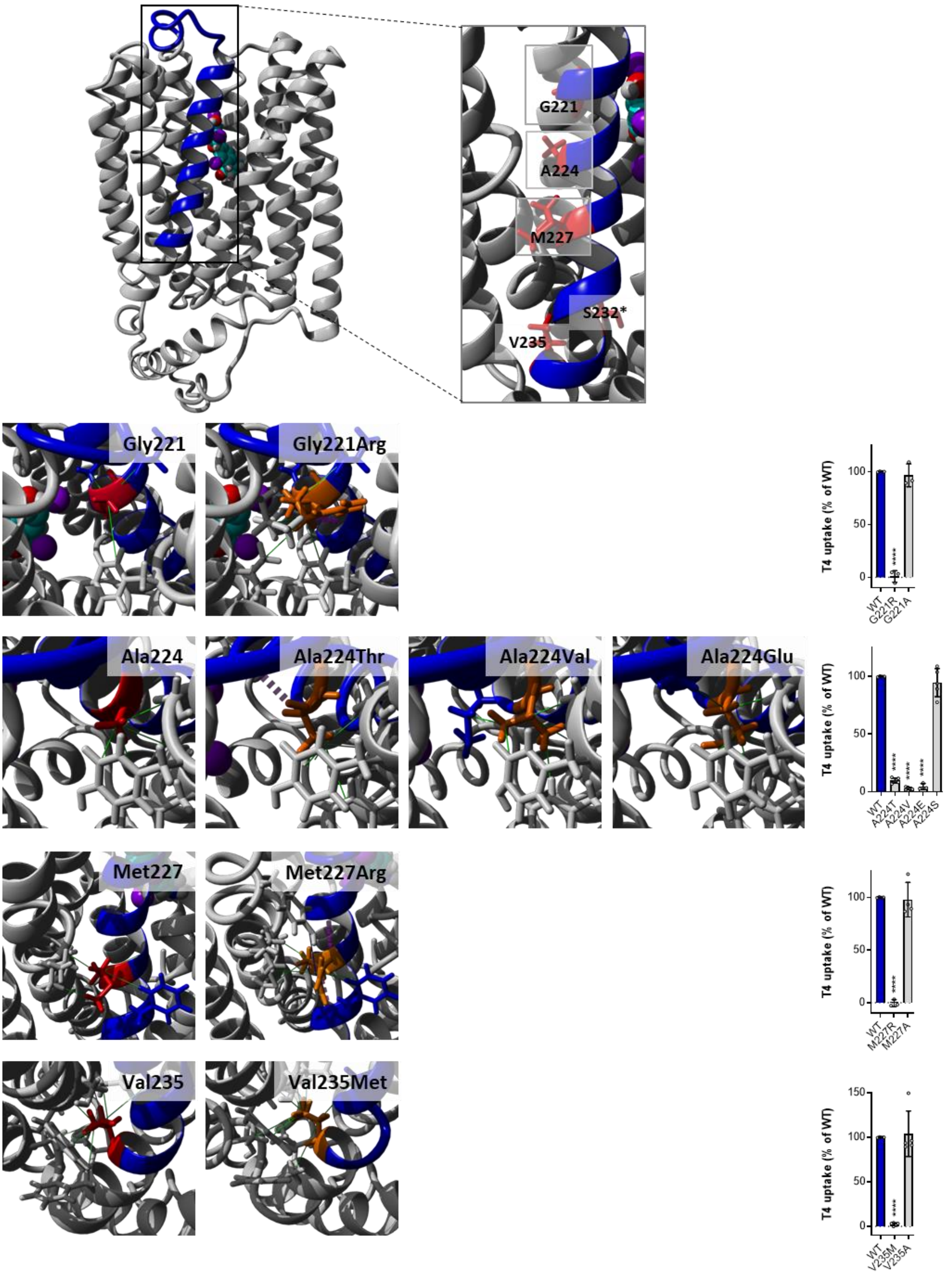

G221
A224
M227
S232*
V235
Gly221
Gly221Arg
Ala224
Ala224Thr
Ala224Val
Ala224Glu
Met227
Met227Arg
Val235
Val235Met
T4 uptake (% of WT)
100
50
0
150
****
WT
G221R
G221A
A224T
A224V
A224E
A224S
M227R
M227A
V235M
V235A

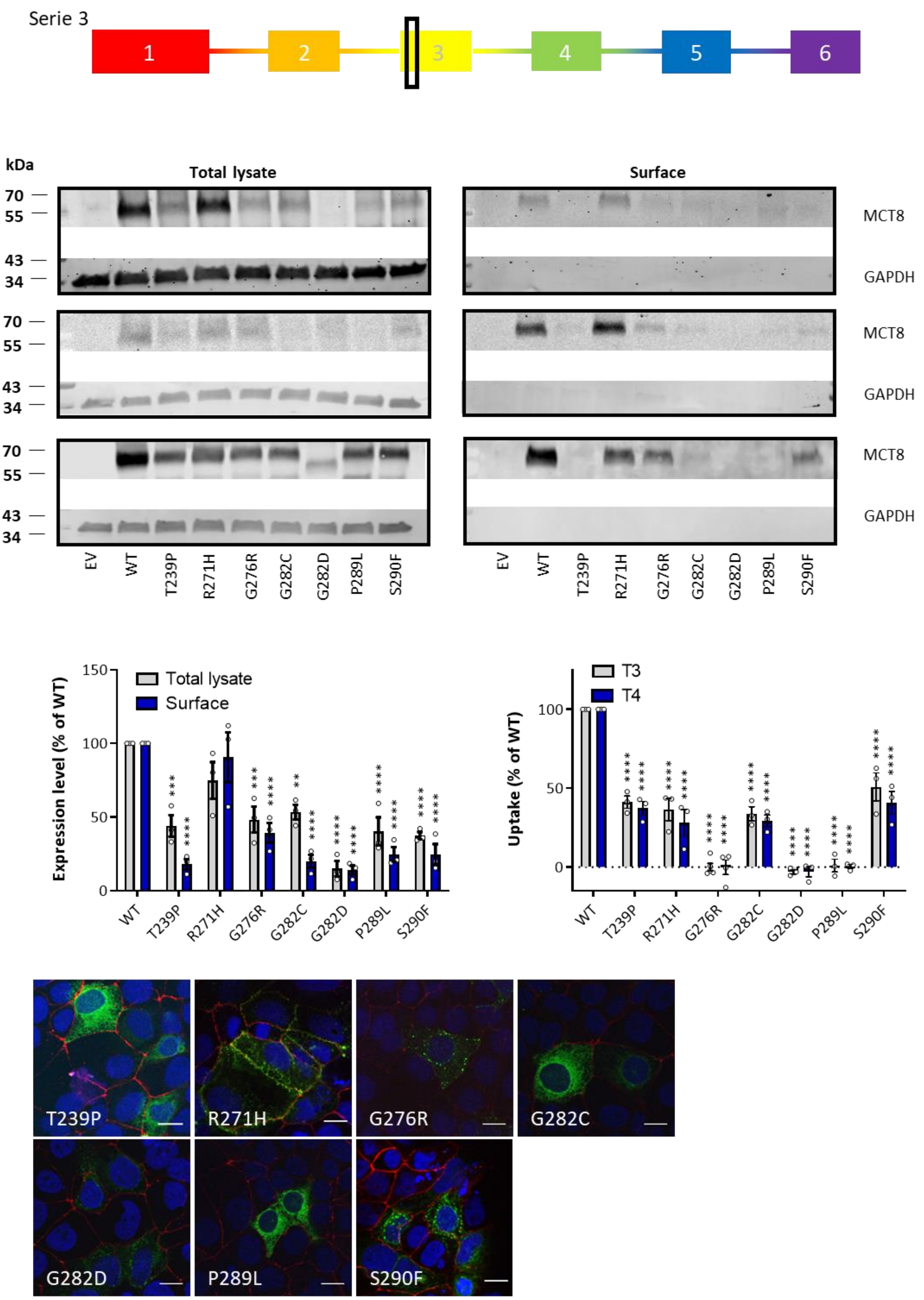
Serie 3
1
2
3
4
5
6
kDa
Total lysate
Surface
70
55
43
34
MCT8
GAPDH
EV
WT
T239P
R271H
G276R
G282C
G282D
P289L
S290F
Expression level (% of WT)
Total lysate
Surface
150
100
50
0
Uptake (% of WT)
T3
T4
T239P
R271H
G276R
G282C
G282D
P289L
S290F

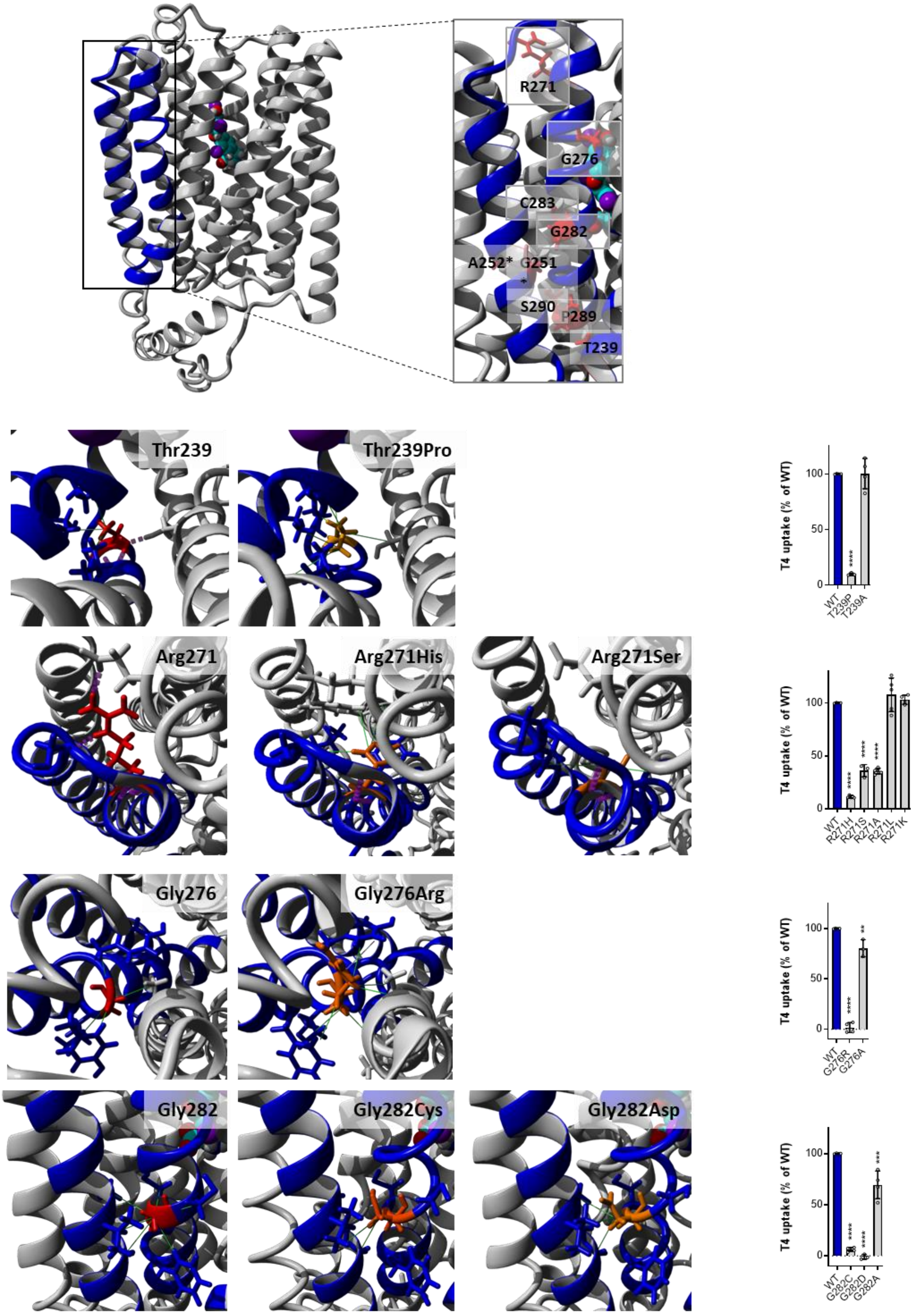
R271
G276
C283
G282
A252*
G251
S290
P289
T239
Thr239
Thr239Pro
Arg271
Arg271His
Arg271Ser
Gly276
Gly276Arg
Gly282
Gly282Cys
Gly282Asp
T4 uptake (% of WT)
WT
T239P
T239A
R271H
R271S
R271A
R271L
R271K
G276R
G276A
G282C
G282D
G282A

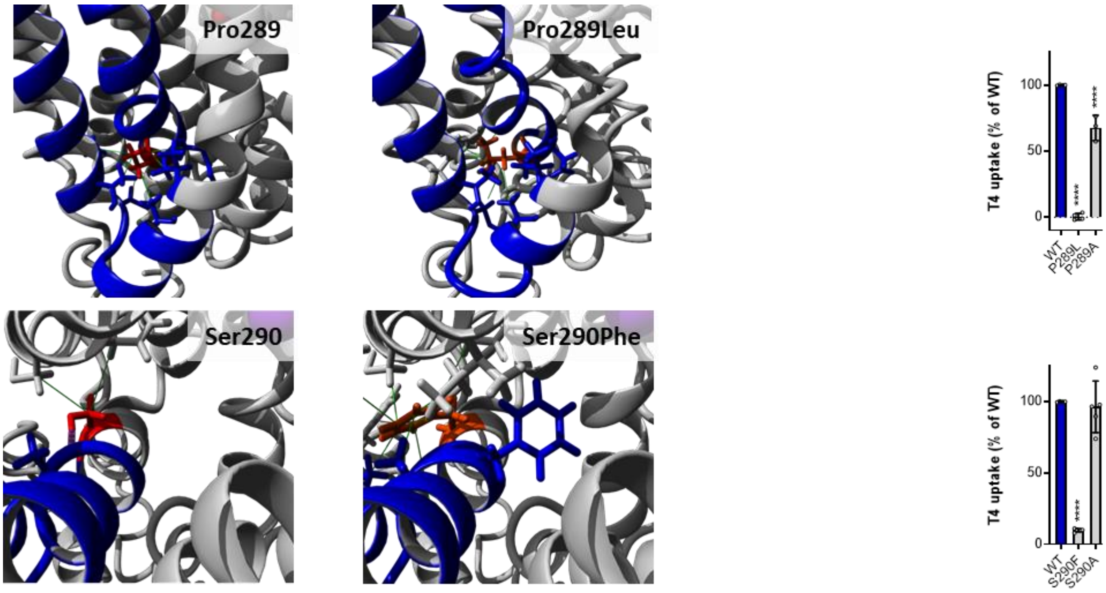
Pro289
Pro289Leu
Ser290
Ser290Phe
T4 uptake (% of WT)
100
50
0
WT
P289L
P289A
****
****
T4 uptake (% of WT)
100
50
0
WT
S290F
S290A
****

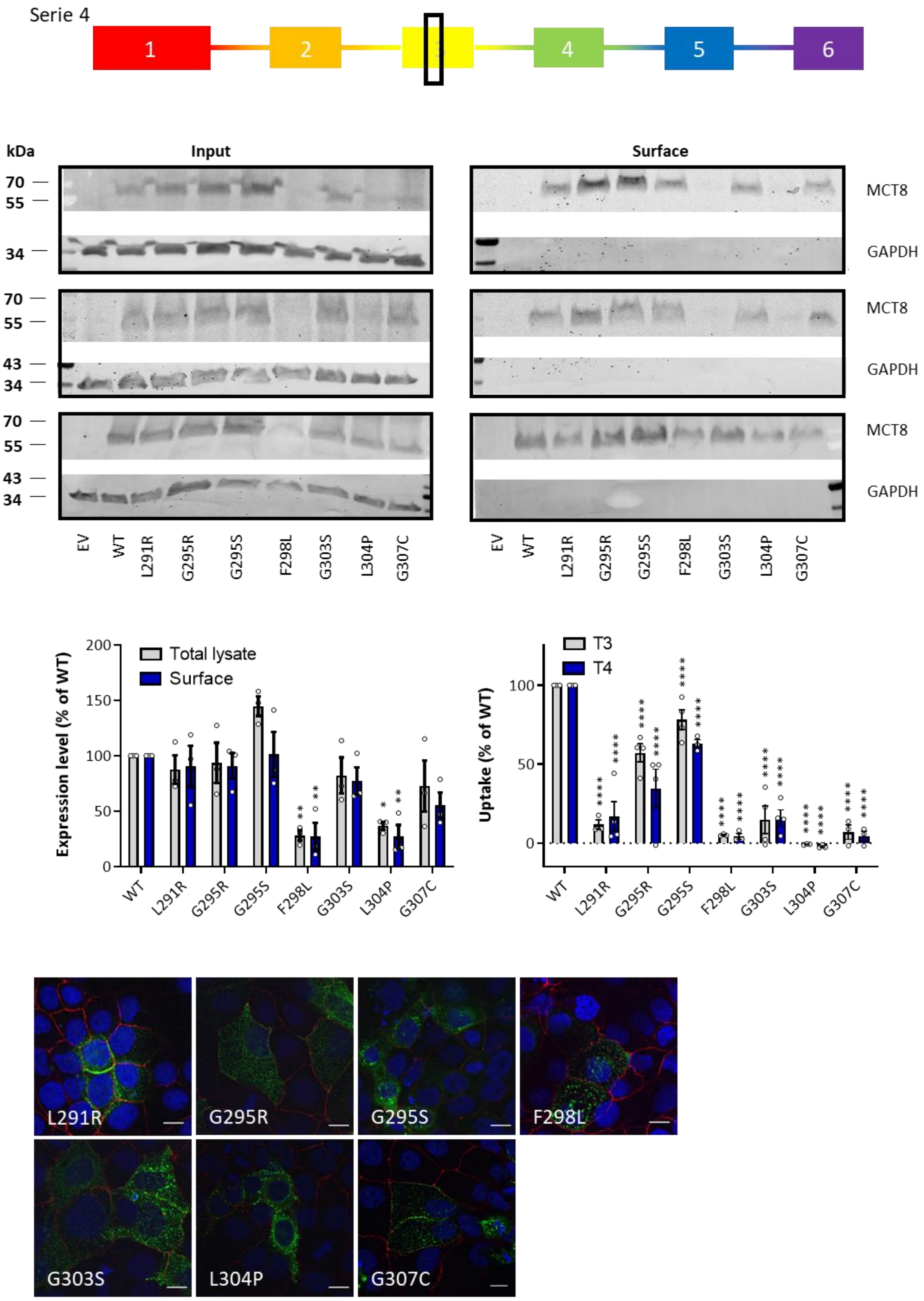
Serie 4
1
2
3
4
5
6
kDa
Input
Surface
70
55
34
43
MCT8
GAPDH
EV
WT
L291R
G295R
G295S
F298L
G303S
L304P
G307C
Expression level (% of WT)
Total lysate
Surface
200
150
100
50
0
Uptake (% of WT)
T3
T4
100
50
0
L291R
G295R
G295S
F298L
G303S
L304P
G307C

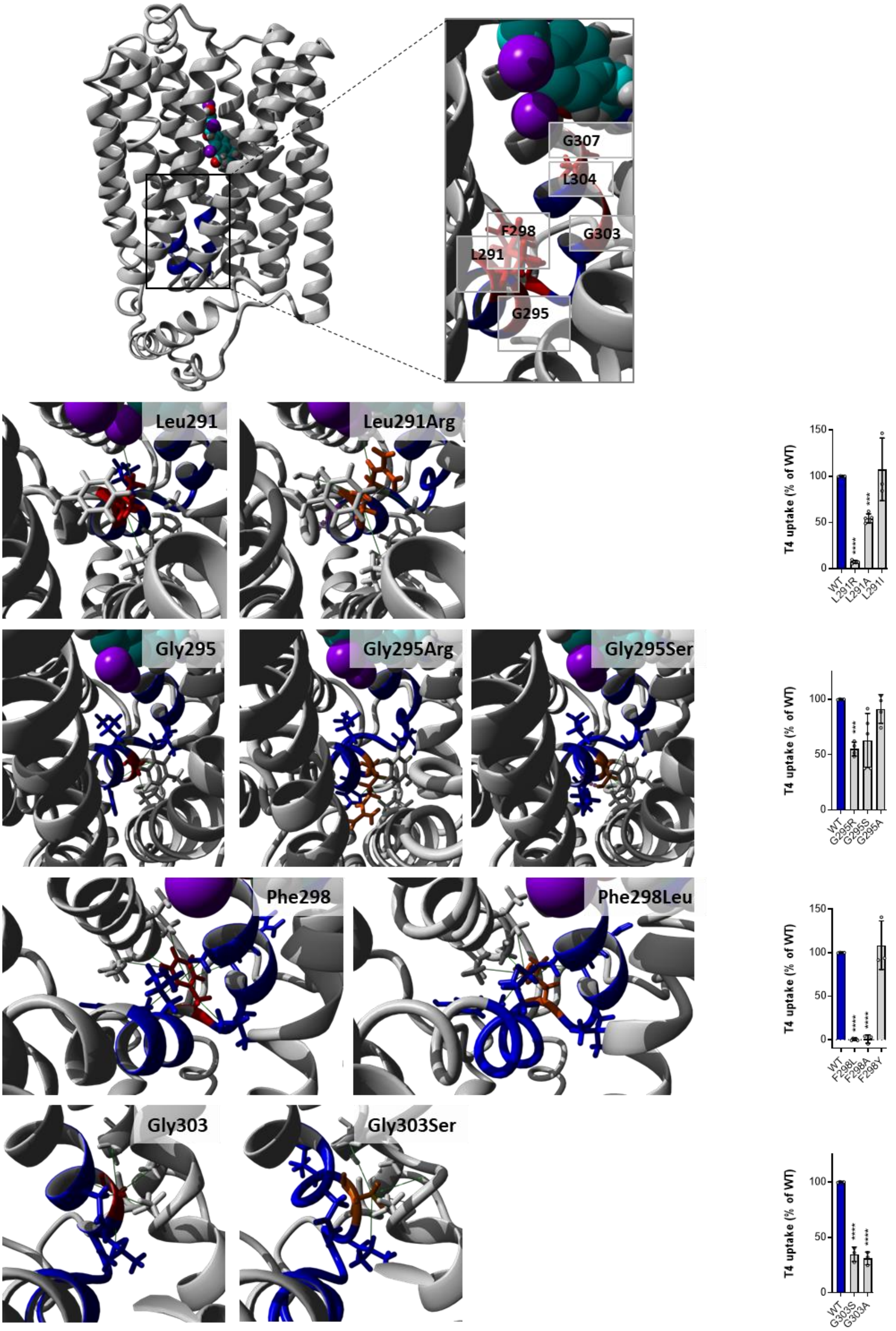
G307
L304
F298
G303
L291
G295
Leu291
Leu291Arg
Gly295
Gly295Arg
Gly295Ser
Phe298
Phe298Leu
Gly303
Gly303Ser
T4 uptake (% of WT)
WT
L291R
L291A
L291I
G295R
G295S
G295A
F298L
F298A
F298Y
G303S
G303A

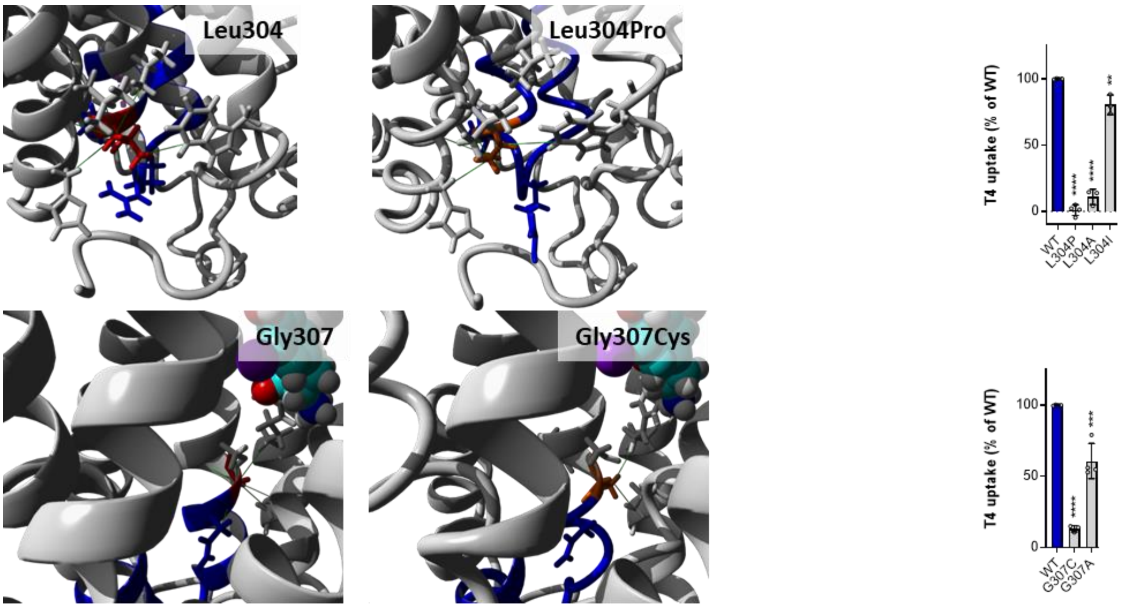
Leu304
Leu304Pro
Gly307
Gly307Cys
T4 uptake (% of WT)
100
50
0
WT
L304P
L304A
L304I
****
****
**
T4 uptake (% of WT)
100
50
0
WT
G307C
G307A
****
***

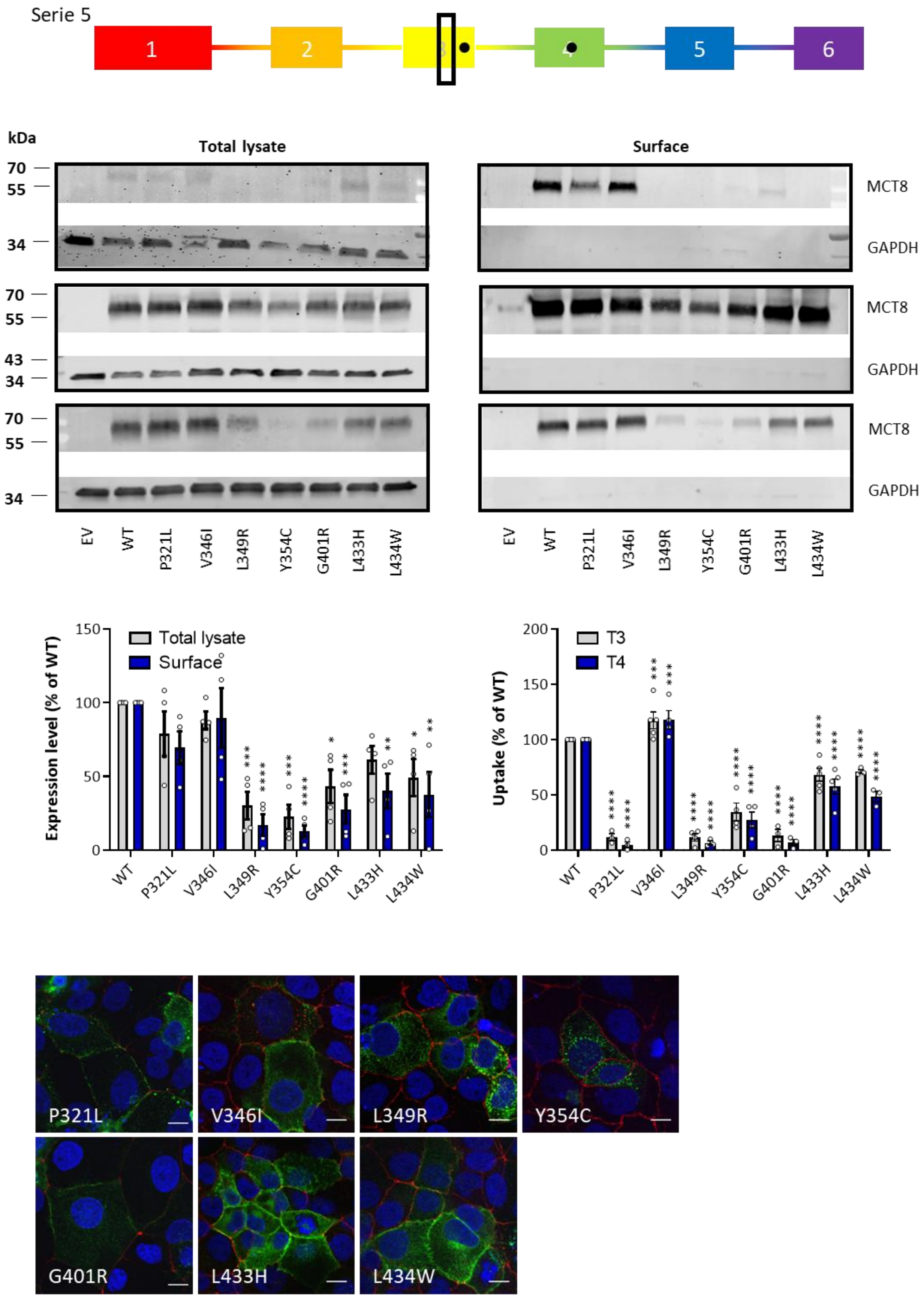

Serie 5
1
2
3
4
5
6
kDa
Total lysate
Surface
70
55
34
43
MCT8
GAPDH
EV
WT
P321L
V346I
L349R
Y354C
G401R
L433H
L434W
Expression level (% of WT)
Total lysate
Surface
Uptake (% of WT)
T3
T4

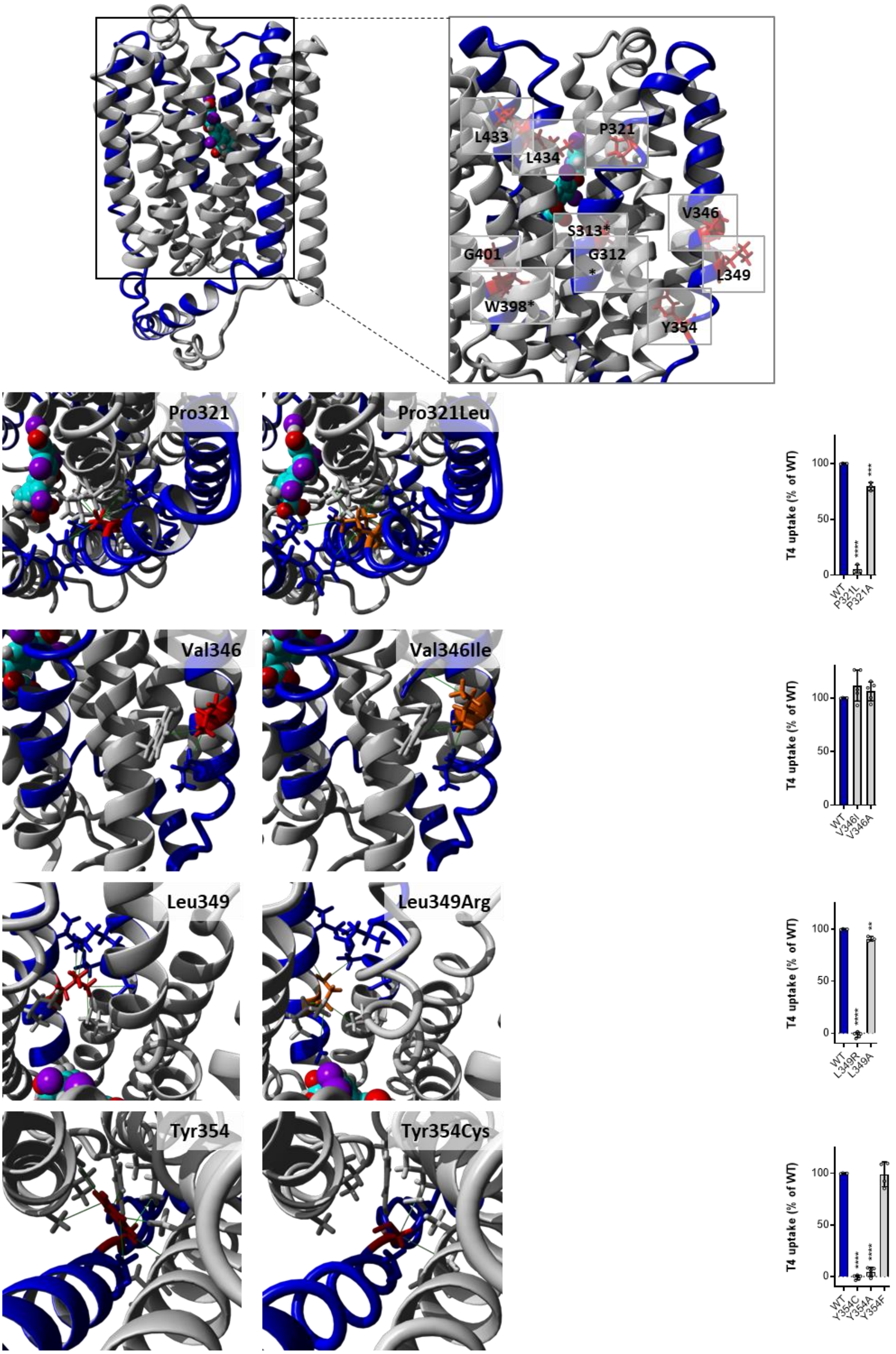
L433
L434
P321
V346
S313*
G401
G312
*
L349
W398*
Y354
Pro321
Pro321Leu
T4 uptake (% of WT)
100
50
0
WT
P321L
P321A
****
***
Val346
Val346Ile
T4 uptake (% of WT)
100
50
0
WT
V346I
V346A
Leu349
Leu349Arg
T4 uptake (% of WT)
100
50
0
WT
L349R
L349A
****
**
Tyr354
Tyr354Cys
T4 uptake (% of WT)
100
50
0
WT
Y354C
Y354A
Y354F
****
****

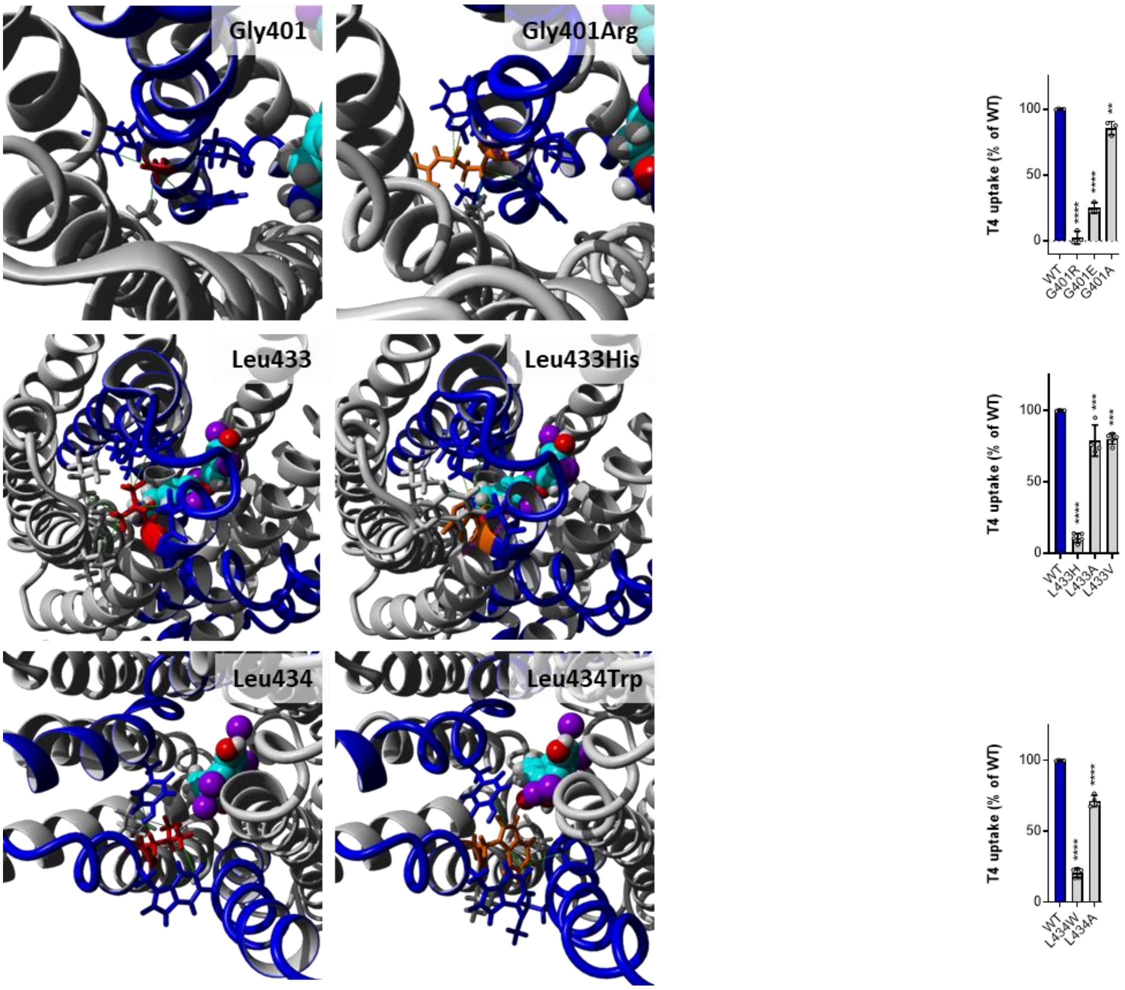
Gly401
Gly401Arg
Leu433
Leu433His
Leu434
Leu434Trp
T4 uptake (% of WT)
100
50
0
WT
G401R
G401E
G401A
T4 uptake (% of WT)
100
50
0
WT
L433H
L433A
L433V
T4 uptake (% of WT)
100
50
0
WT
L434W
L434A

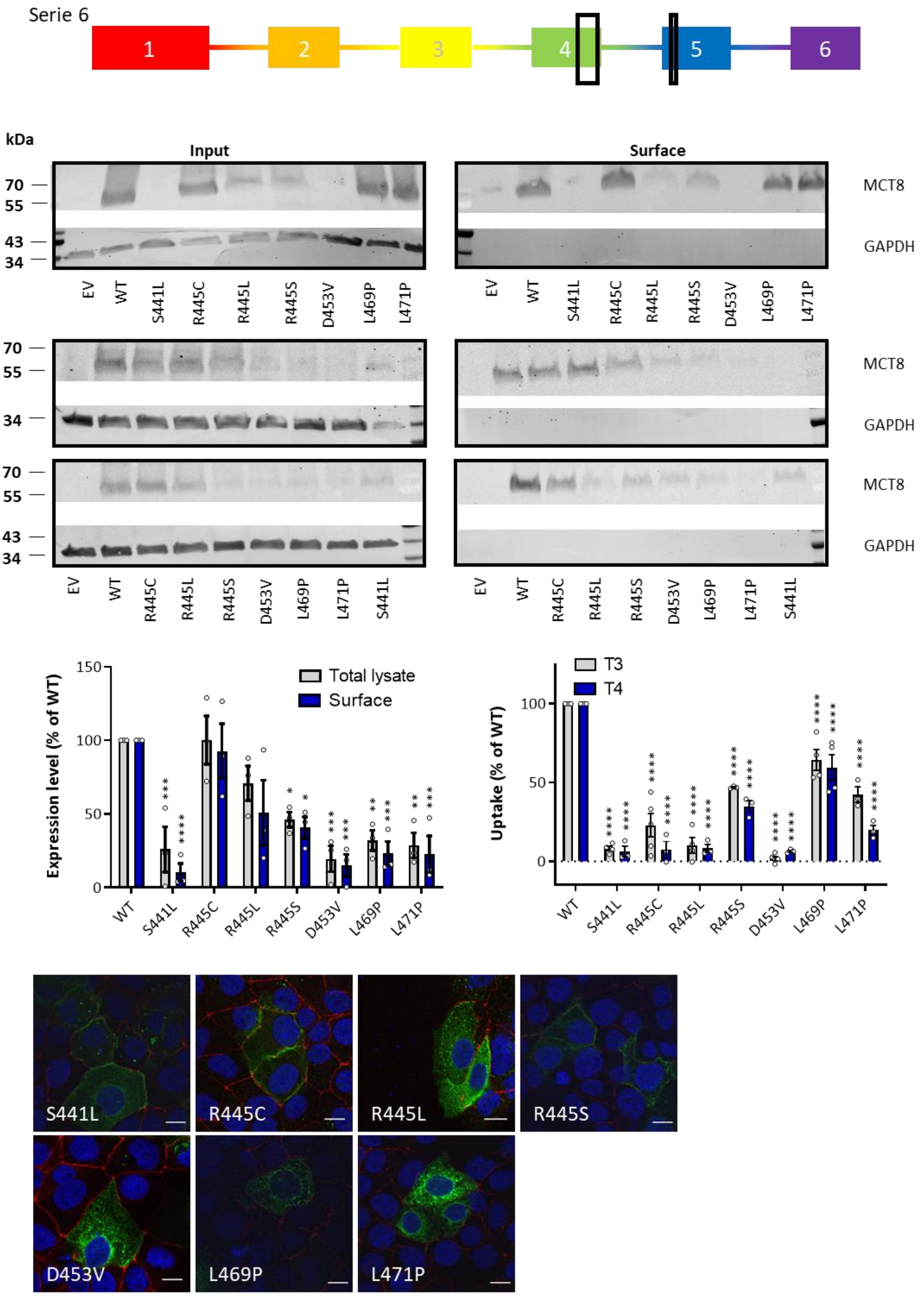
Serie 6
1
2
3
4
5
6
kDa
Input
Surface
70
55
43
34
MCT8
GAPDH
EV
WT
S441L
R445C
R445L
R445S
D453V
L469P
L471P
Expression level (% of WT)
Total lysate
Surface
150
100
50
0
Uptake (% of WT)
T3
T4
S441L
R445C
R445L
R445S
D453V
L469P
L471P

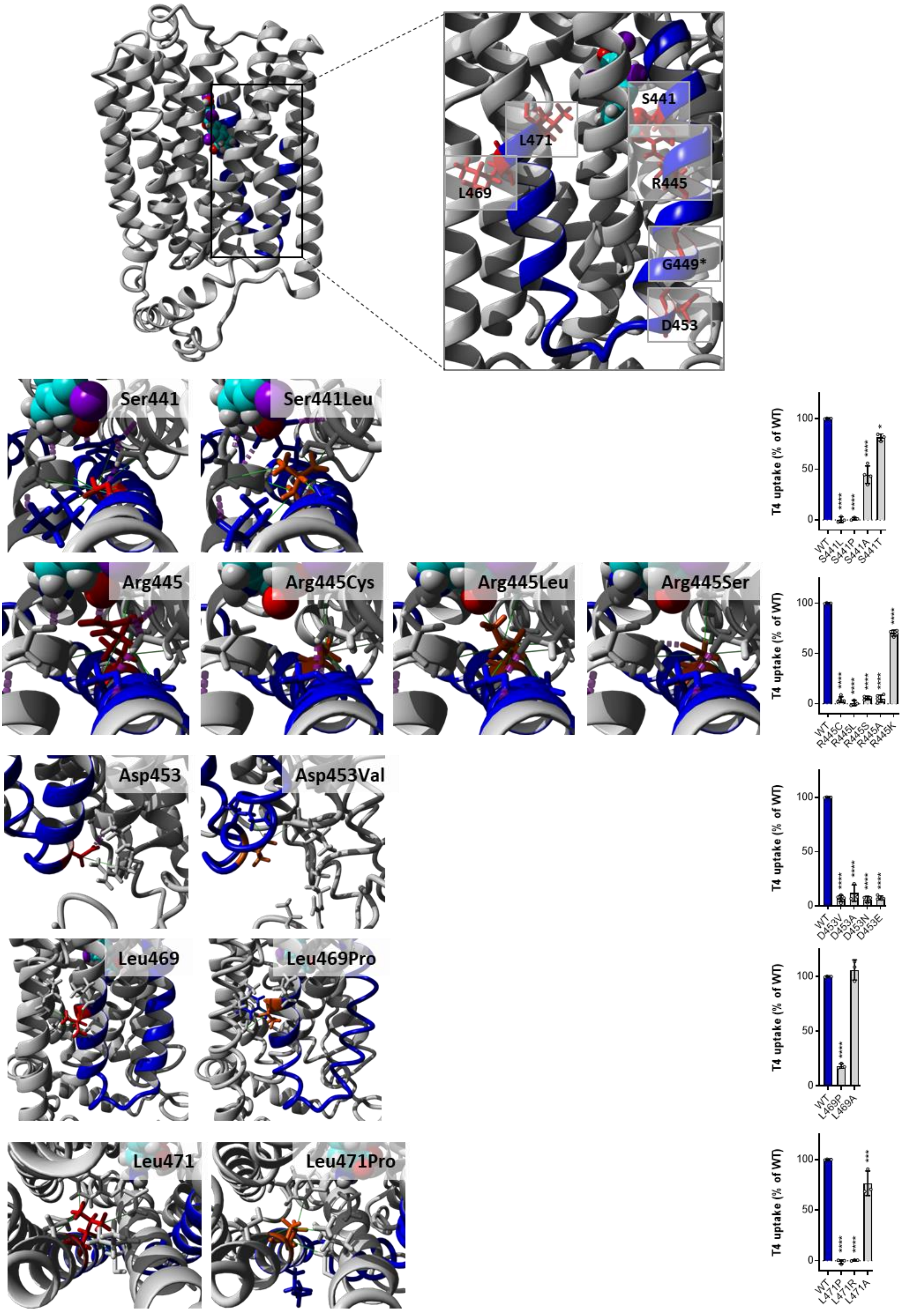

S441
L471
R445
L469
G449*
D453
Ser441
Ser441Leu
Arg445
Arg445Cys
Arg445Leu
Arg445Ser
Asp453
Asp453Val
Leu469
Leu469Pro
Leu471
Leu471Pro
T4 uptake (% of WT)
0
50
100
WT S441L S441P S441A S441T
WT R445C R445L R445S R445A R445K
WT D453V D453A D453N D453E
WT L469P L469A
WT L471P L471R L471A

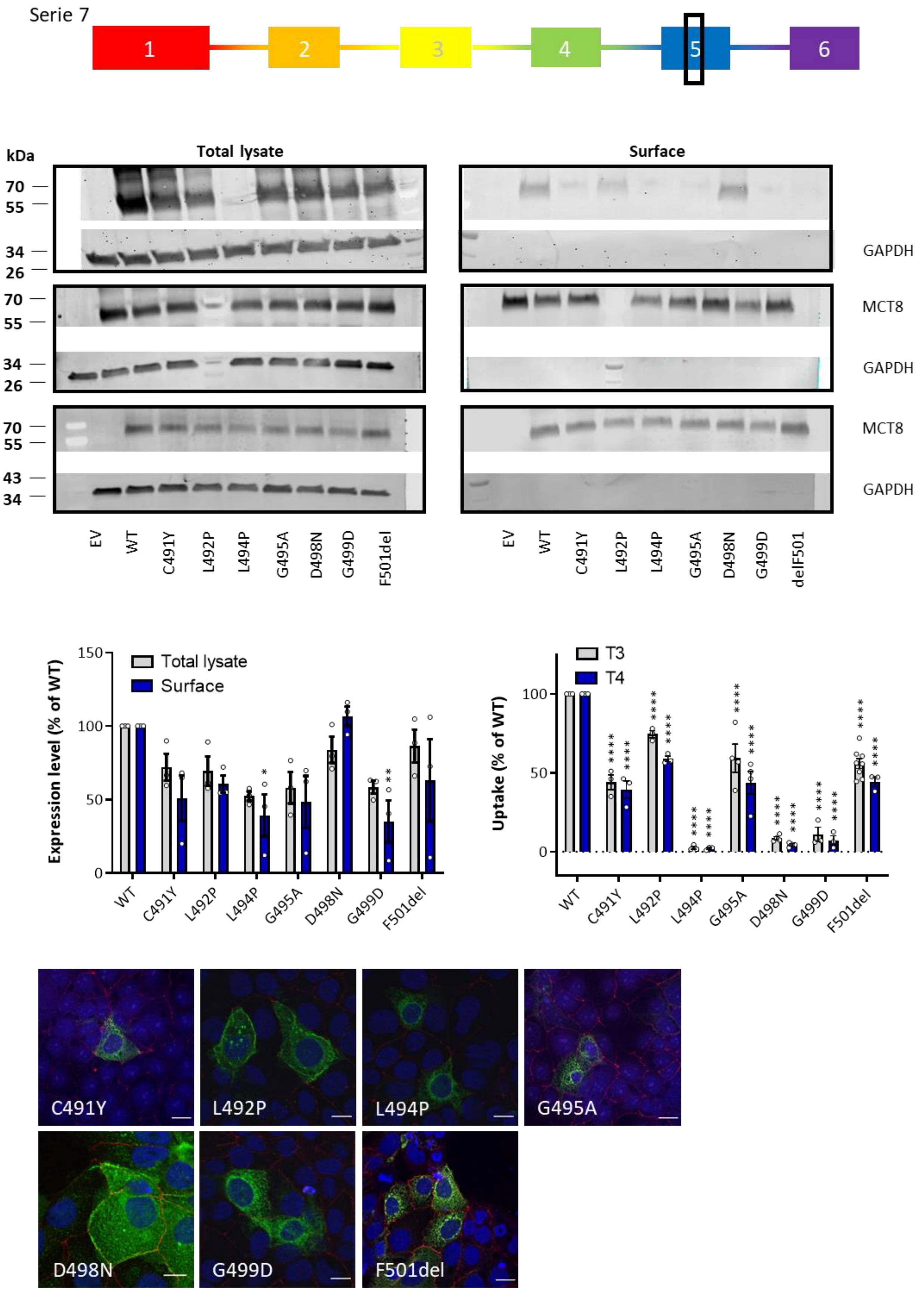
Serie 7
1
2
3
4
5
6
kDa
Total lysate
Surface
70
55
34
26
43
GAPDH
MCT8
EV
WT
C491Y
L492P
L494P
G495A
D498N
G499D
F501del
delF501
Expression level (% of WT)
Total lysate
Surface
150
100
50
0
Uptake (% of WT)
T3
T4
C491Y
L492P
L494P
G495A
D498N
G499D
F501del

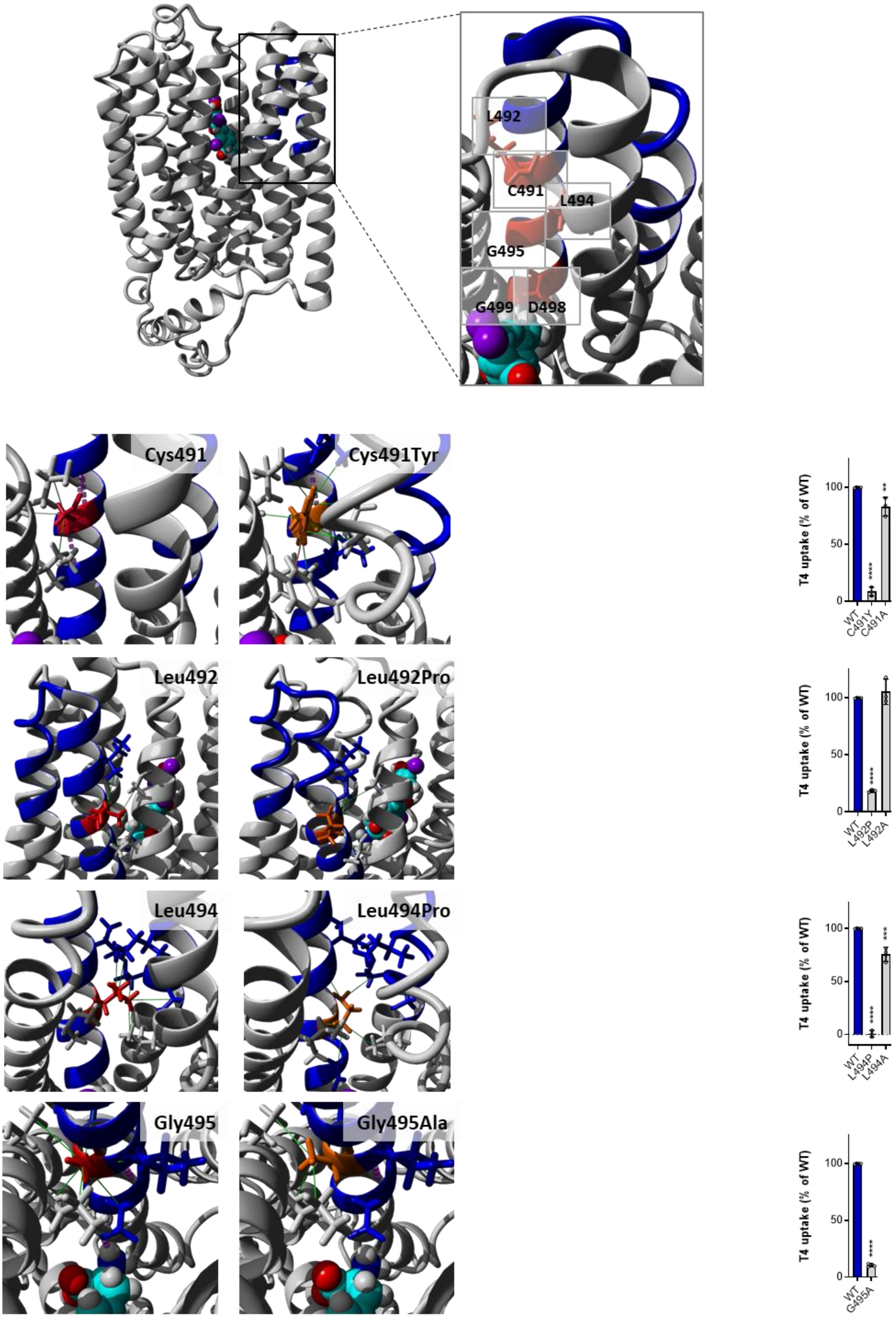
L492
C491
L494
G495
G499
D498
Cys491
Cys491Tyr
Leu492
Leu492Pro
Leu494
Leu494Pro
Gly495
Gly495Ala
T4 uptake (% of WT)
100
50
0
WT
C491Y
C491A
L492P
L492A
L494P
L494A
G495A
****
**
***

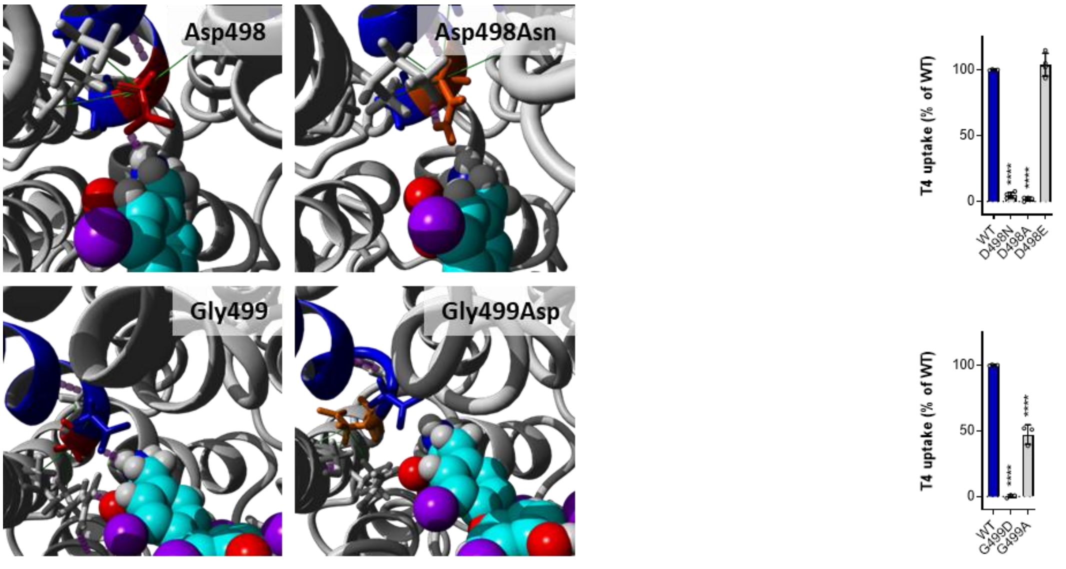
Asp498
Asp498Asn
Gly499
Gly499Asp
T4 uptake (% of WT)
100
50
0
WT
D498N
D498A
D498E
****
T4 uptake (% of WT)
100
50
0
WT
G499D
G499A
****

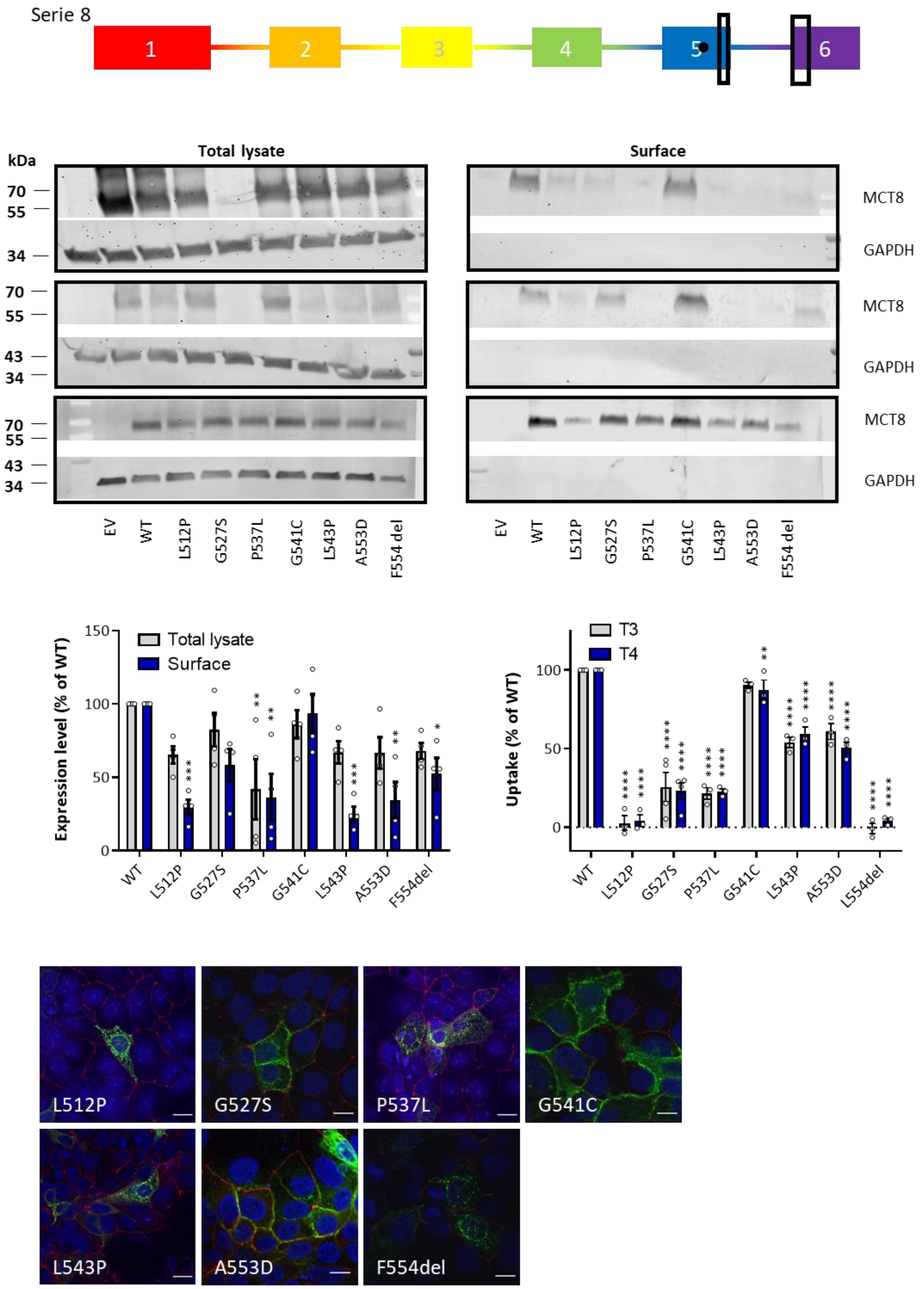
Serie 8
1
2
3
4
5
6
Total lysate
Surface
kDa
70
55
34
43
MCT8
GAPDH
EV
WT
L512P
G527S
P537L
G541C
L543P
A553D
F554 del
Expression level (% of WT)
Total lysate
Surface
150
100
50
0
F554del
Uptake (% of WT)
T3
T4
L554del
L512P
G527S
P537L
G541C
L543P
A553D
F554del

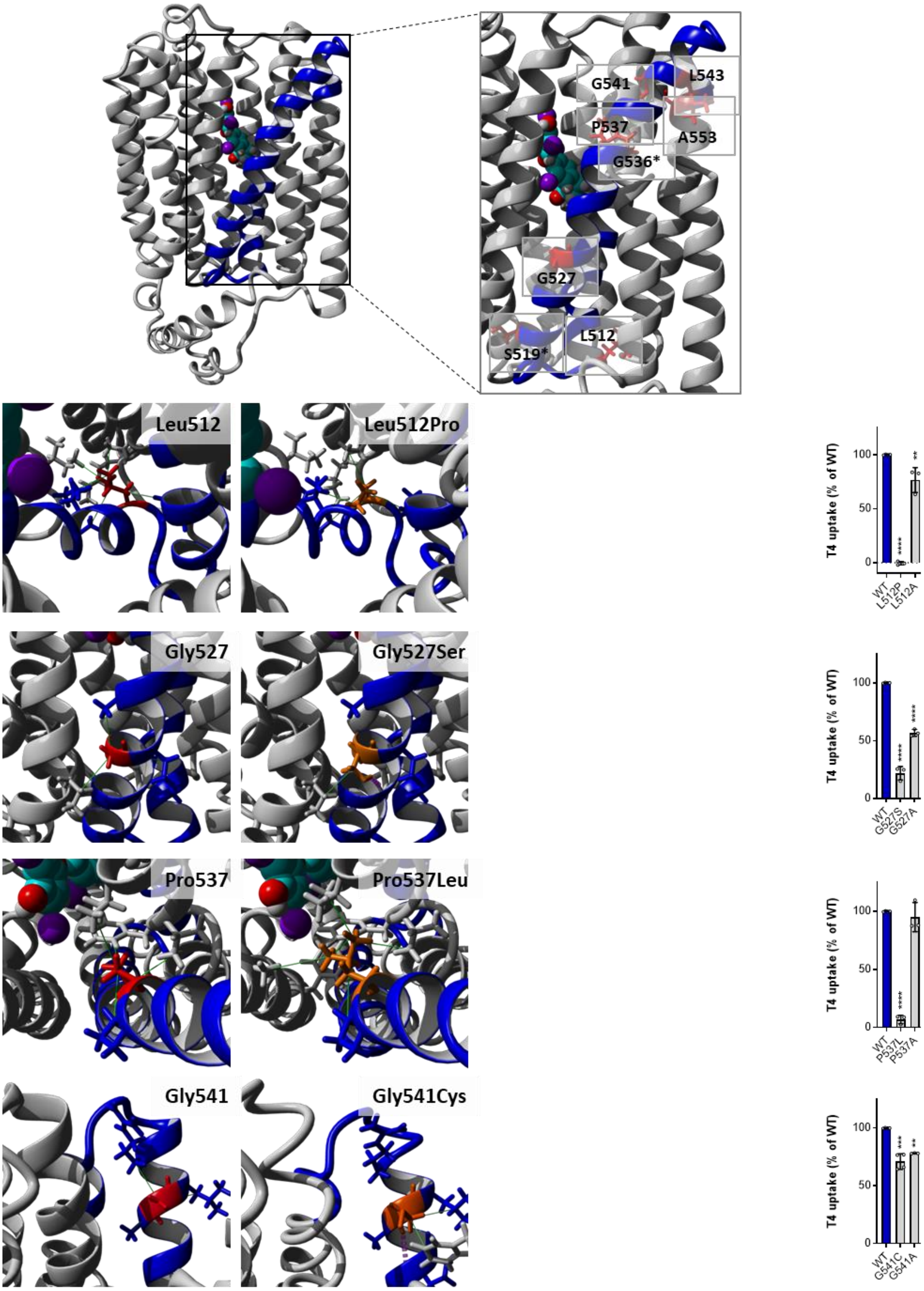
G541
L543
P537
A553
G536*
G527
L512
S519*
Leu512
Leu512Pro
Gly527
Gly527Ser
Pro537
Pro537Leu
Gly541
Gly541Cys
T4 uptake (% of WT)
100
50
0
WT
L512P
L512A
G527S
G527A
P537L
P537A
G541C
G541A
****
**
***

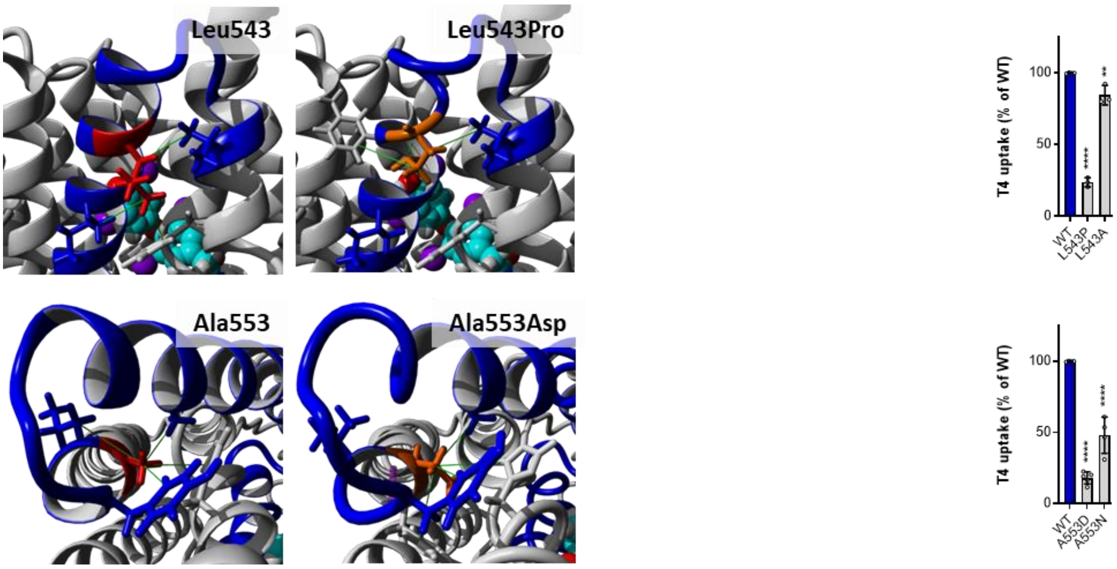
Leu543
Leu543Pro
Ala553
Ala553Asp
T4 uptake (% of WT)
100
50
0
WT
L543P
L543A
****
**
T4 uptake (% of WT)
100
50
0
WT
A553D
A553N
****
****

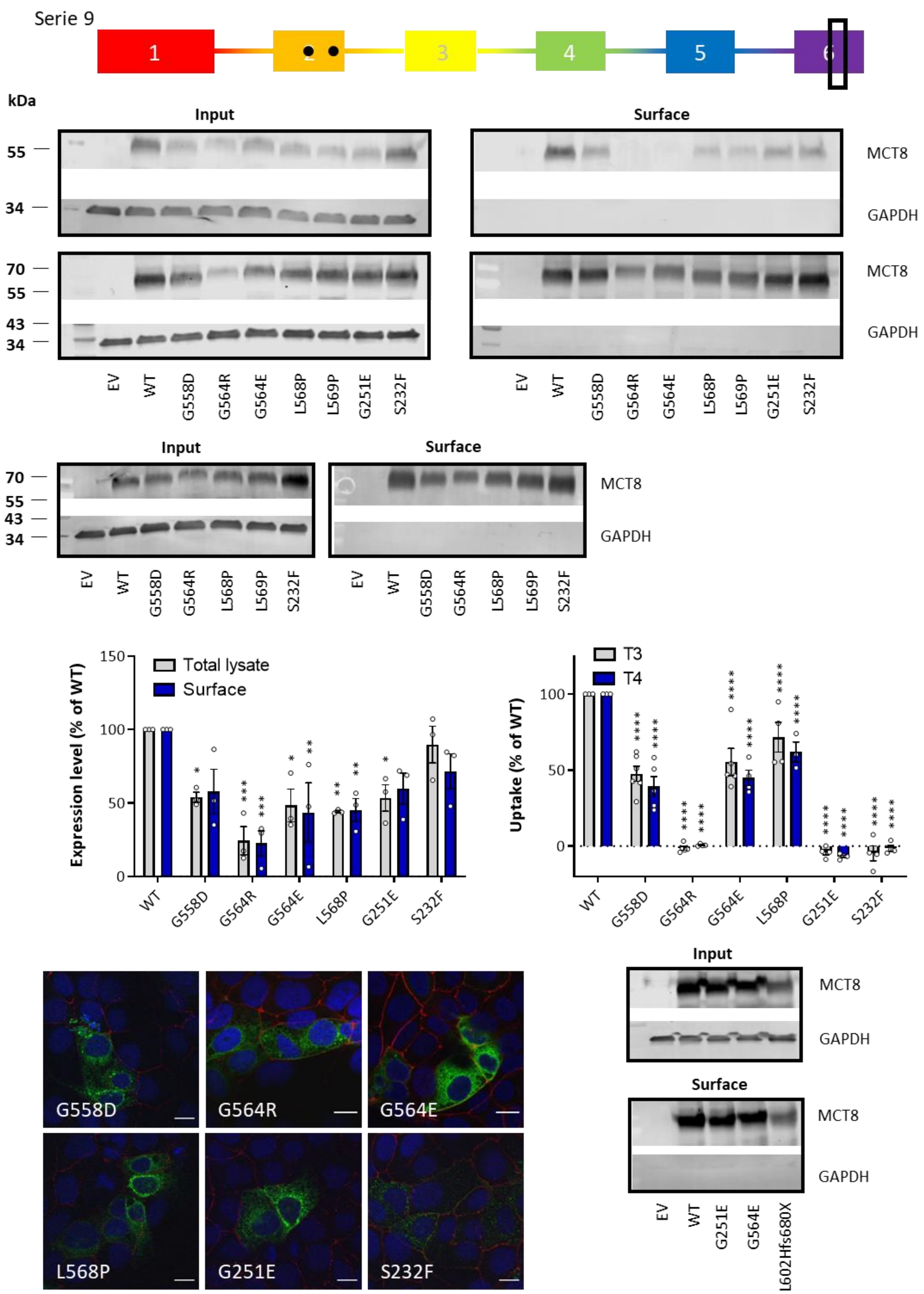

Serie 9
1
2
3
4
5
6
kDa
Input
Surface
55
34
70
55
43
34
MCT8
GAPDH
EV
WT
G558D
G564R
G564E
L568P
L569P
G251E
S232F
Total lysate
Surface
Expression level (% of WT)
T3
T4
Uptake (% of WT)
G558D
G564R
G564E
L568P
G251E
S232F
L602Hfs680X

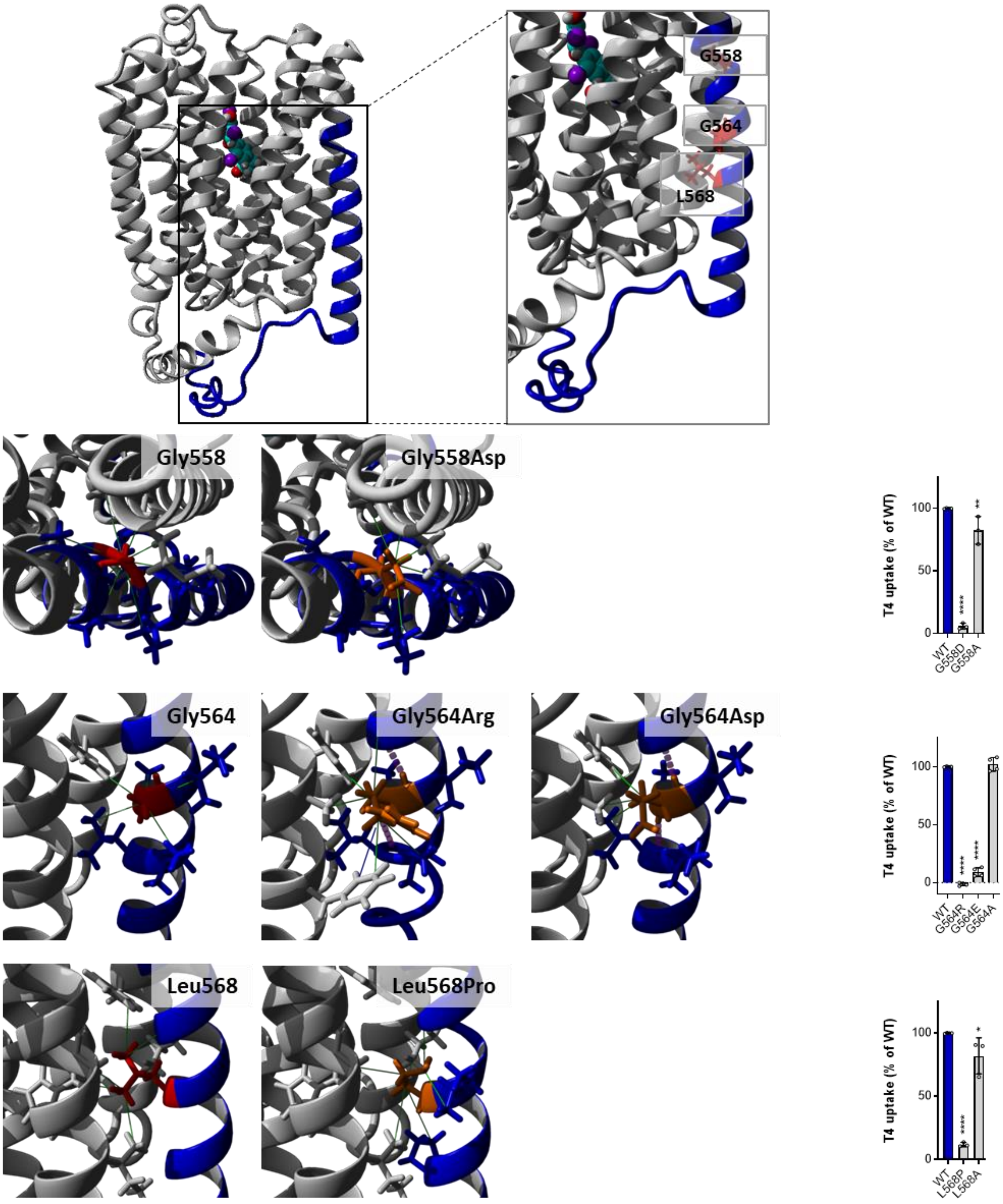
G558
G564
L568
Gly558
Gly558Asp
T4 uptake (% of WT)
100
50
0
WT
G558D
G558A
****
**
Gly564
Gly564Arg
Gly564Asp
T4 uptake (% of WT)
100
50
0
WT
G564R
G564E
G564A
****
****
Leu568
Leu568Pro
T4 uptake (% of WT)
100
50
0
WT
L568P
L568A
****
*

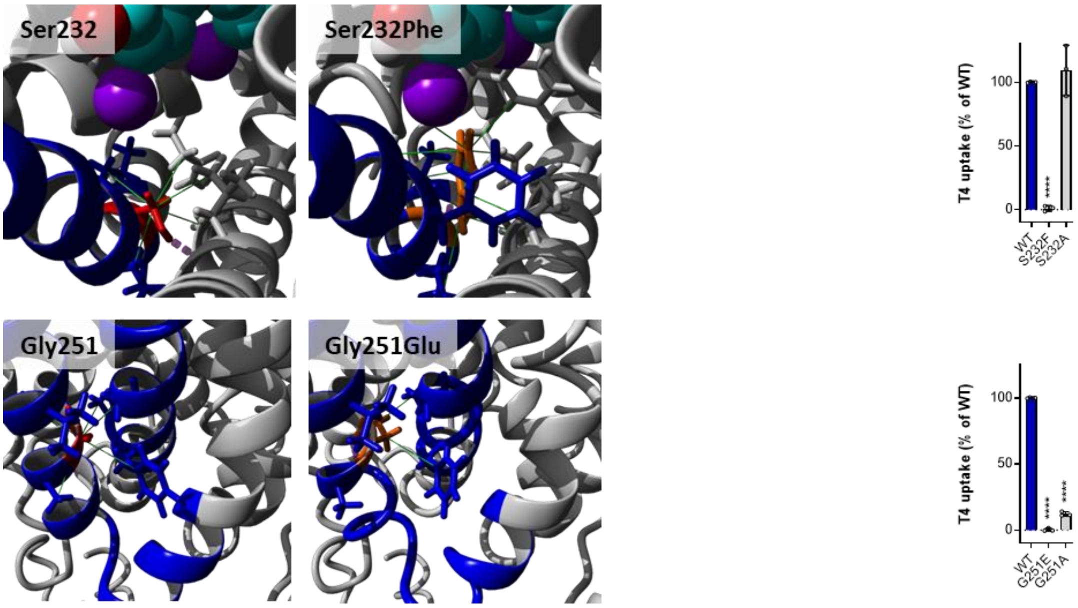
Ser232
Ser232Phe
Gly251
Gly251Glu
T4 uptake (% of WT)
100
50
0
WT
S232F
S232A
****
T4 uptake (% of WT)
100
50
0
WT
G251E
G251A
****
****

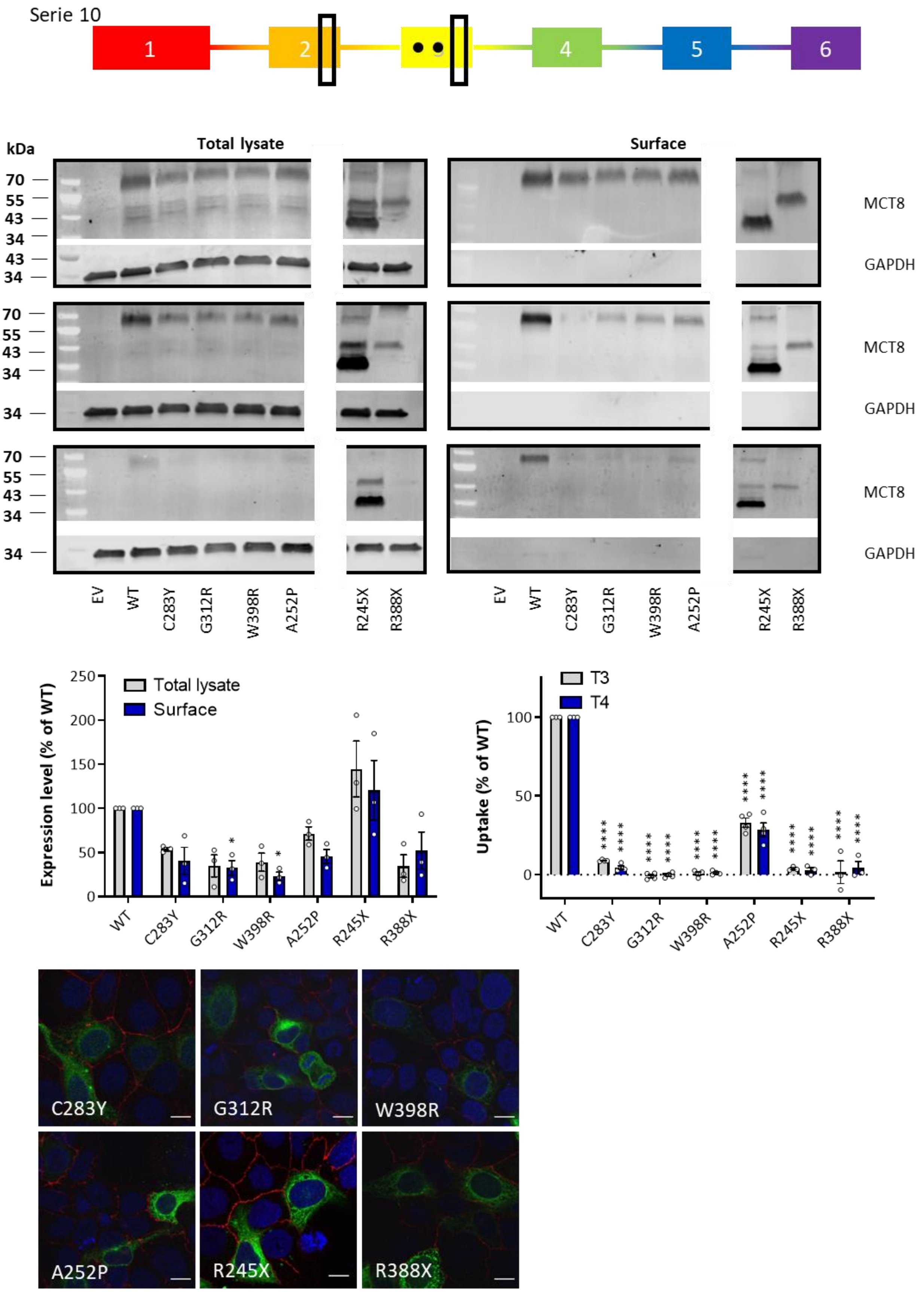
Serie 10
1
2
4
5
6
kDa
Total lysate
Surface
70
55
43
34
MCT8
GAPDH
EV
WT
C283Y
G312R
W398R
A252P
R245X
R388X
Expression level (% of WT)
Total lysate
Surface
Uptake (% of WT)
T3
T4

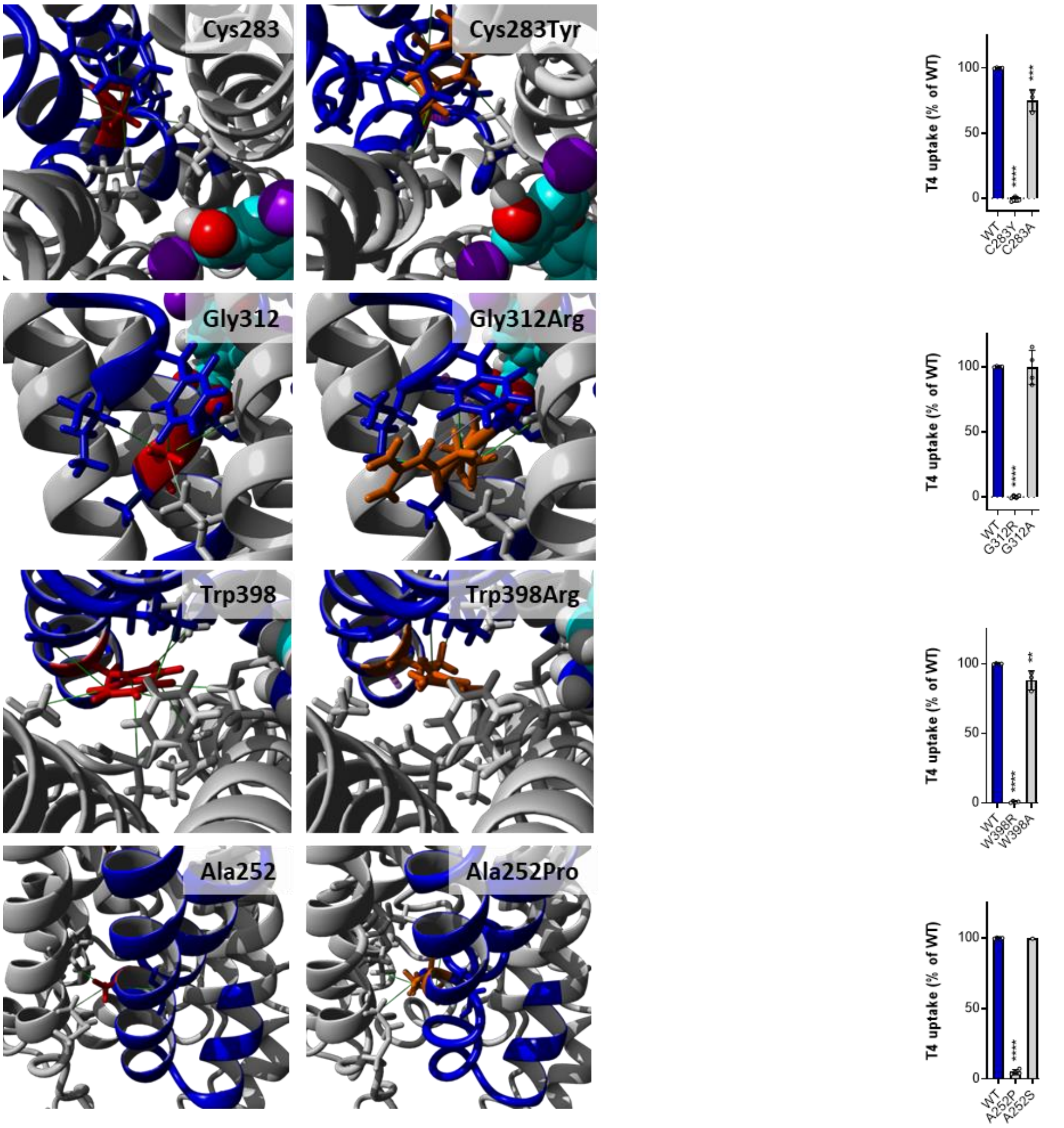
Cys283
Cys283Tyr
Gly312
Gly312Arg
Trp398
Trp398Arg
Ala252
Ala252Pro
T4 uptake (% of WT)
100
50
0
WT
C283Y
C283A
G312R
G312A
W398R
W398A
A252P
A252S
****
***
**

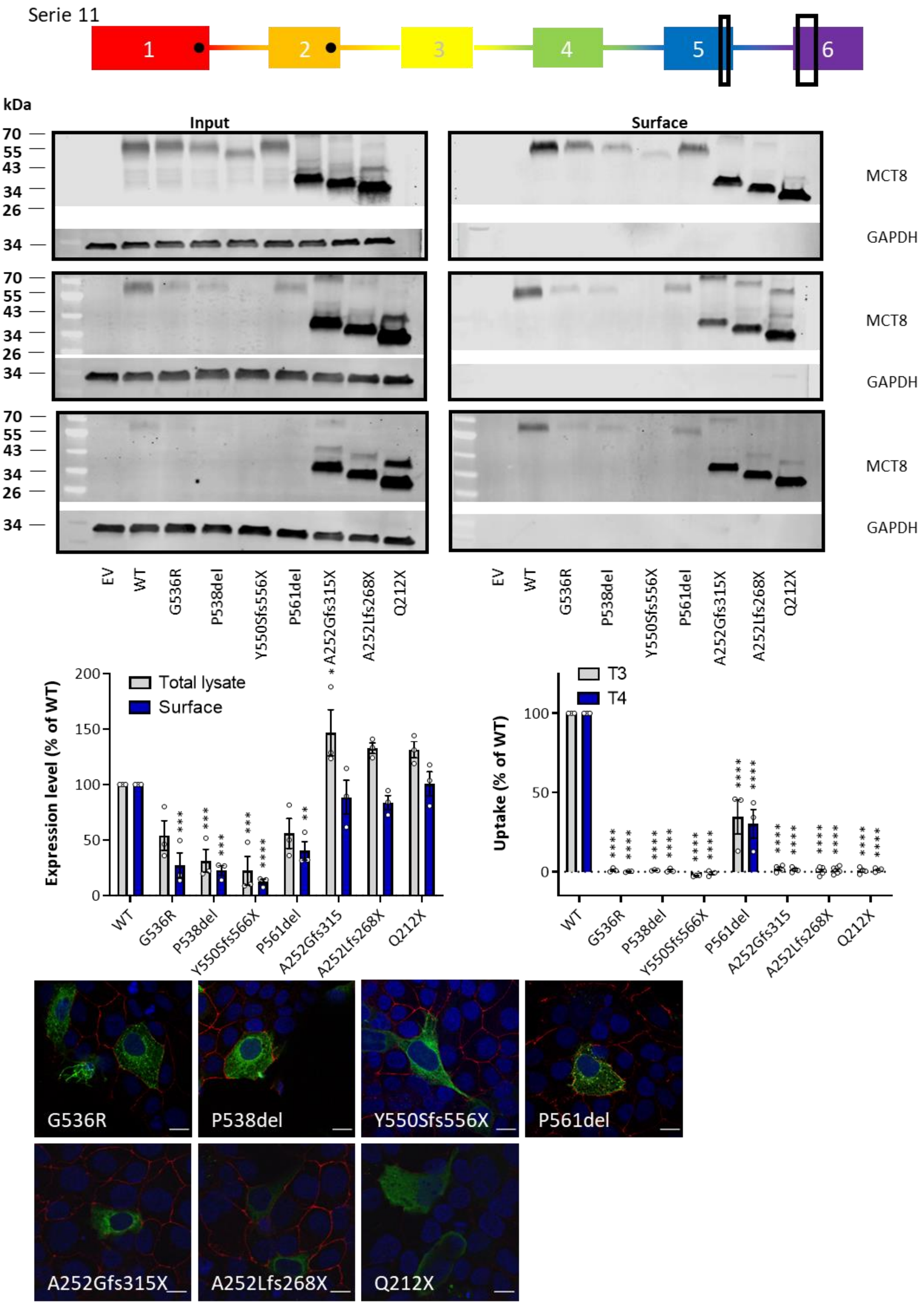

Serie 11
1
2
3
4
5
6
kDa
Input
Surface
70
55
43
34
26
34
MCT8
GAPDH
EV
WT
G536R
P538del
Y550Sfs556X
P561del
A252Gfs315X
A252Lfs268X
Q212X
Expression level (% of WT)
Total lysate
Surface
Uptake (% of WT)
T3
T4
A252Gfs315

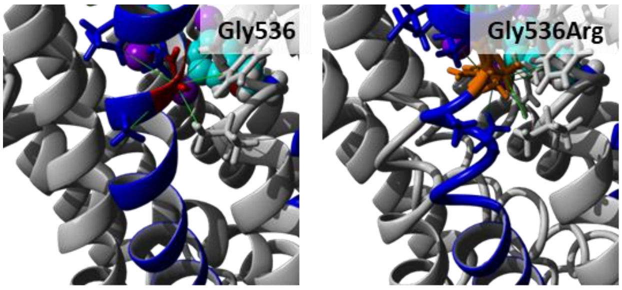
Gly536
Gly536Arg

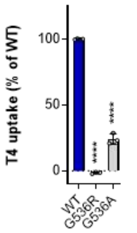
T4 uptake (% of WT)
100
50
0
****
****
WT
G536R
G536A

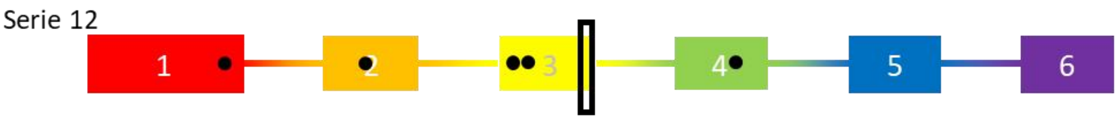
Serie 12
1
2
3
4
5
6

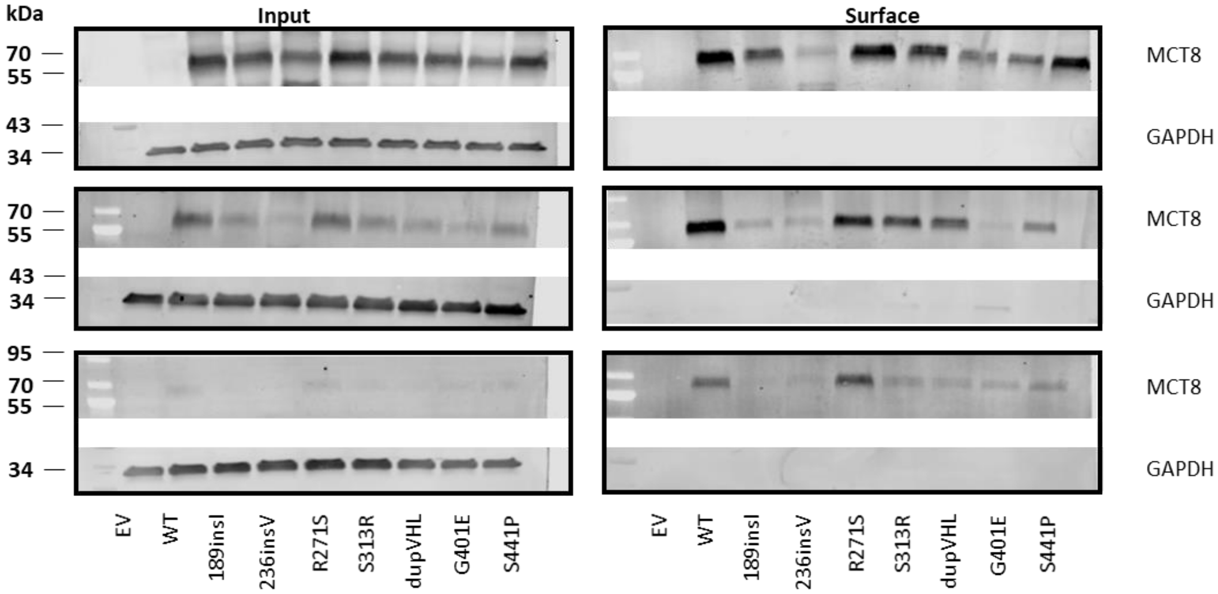
kDa
Input
Surface
70
55
43
34
95
MCT8
GAPDH
EV
WT
189insI
236insV
R271S
S313R
dupVHL
G401E
S441P

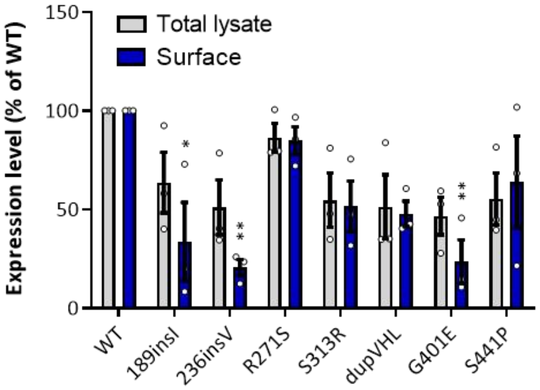
Expression level (% of WT)
Total lysate
Surface
150
100
50
0
WT
189insI
236insV
R271S
S313R
dupVHL
G401E
S441P

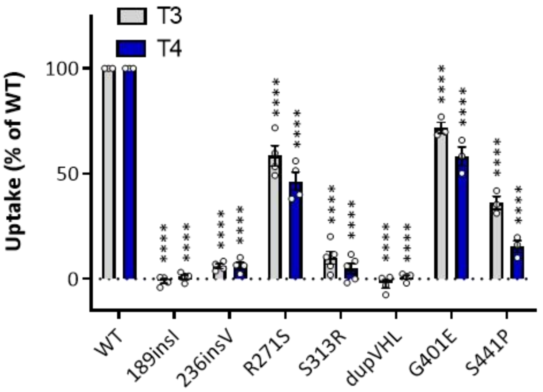
Uptake (% of WT)
T3
T4
100
50
0
WT
189insI
236insV
R271S
S313R
dupVHL
G401E
S441P

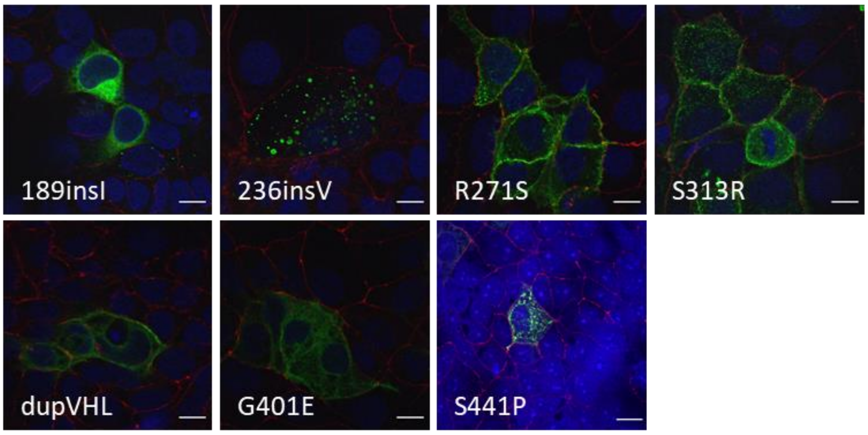
189insI
236insV
R271S
S313R
dupVHL
G401E
S441P

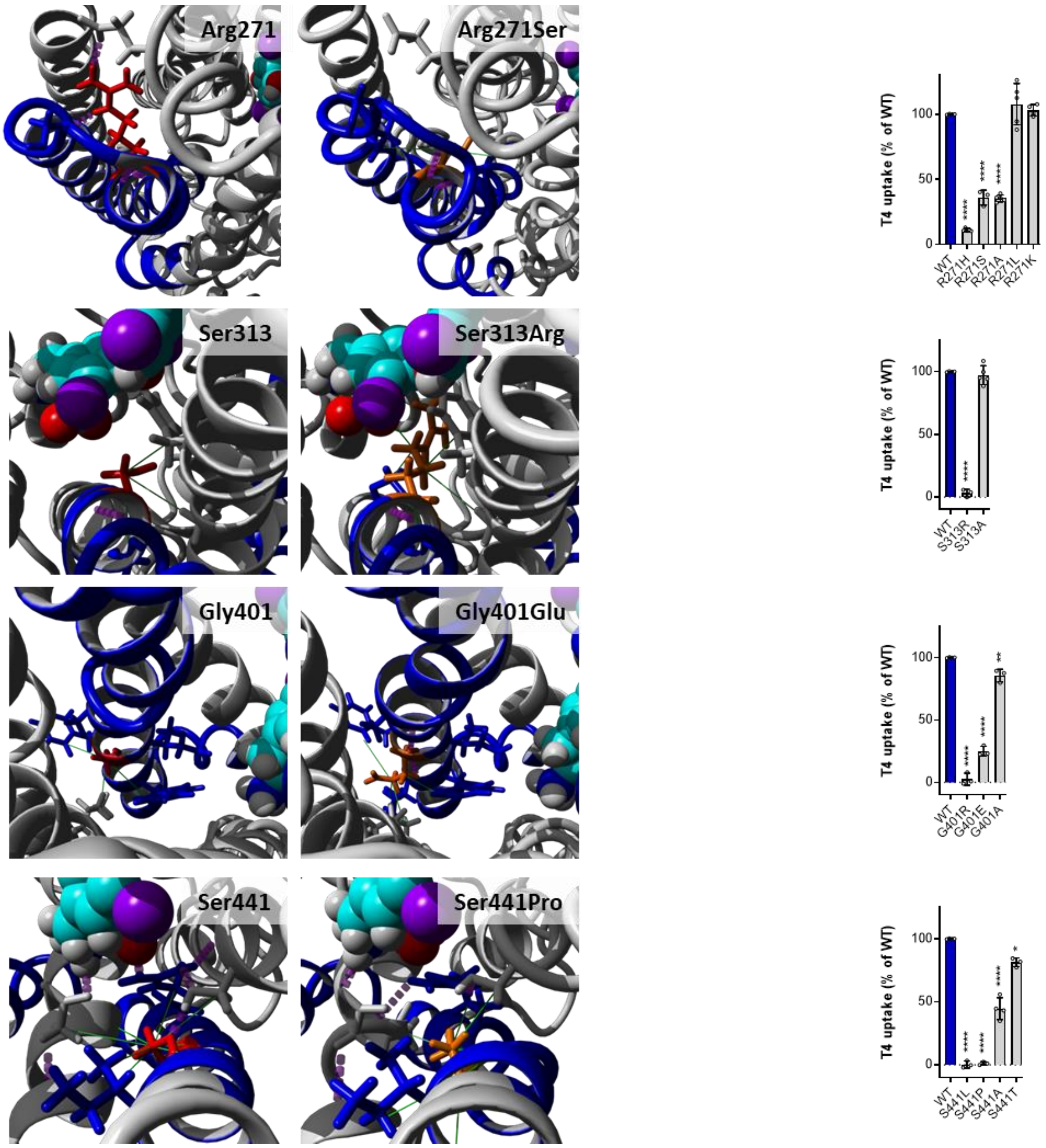
Arg271
Arg271Ser
Ser313
Ser313Arg
Gly401
Gly401Glu
Ser441
Ser441Pro
T4 uptake (% of WT)
WT
R271H
R271S
R271A
R271L
R271K
S313R
S313A
G401R
G401E
G401A
S441L
S441P
S441A
S441T

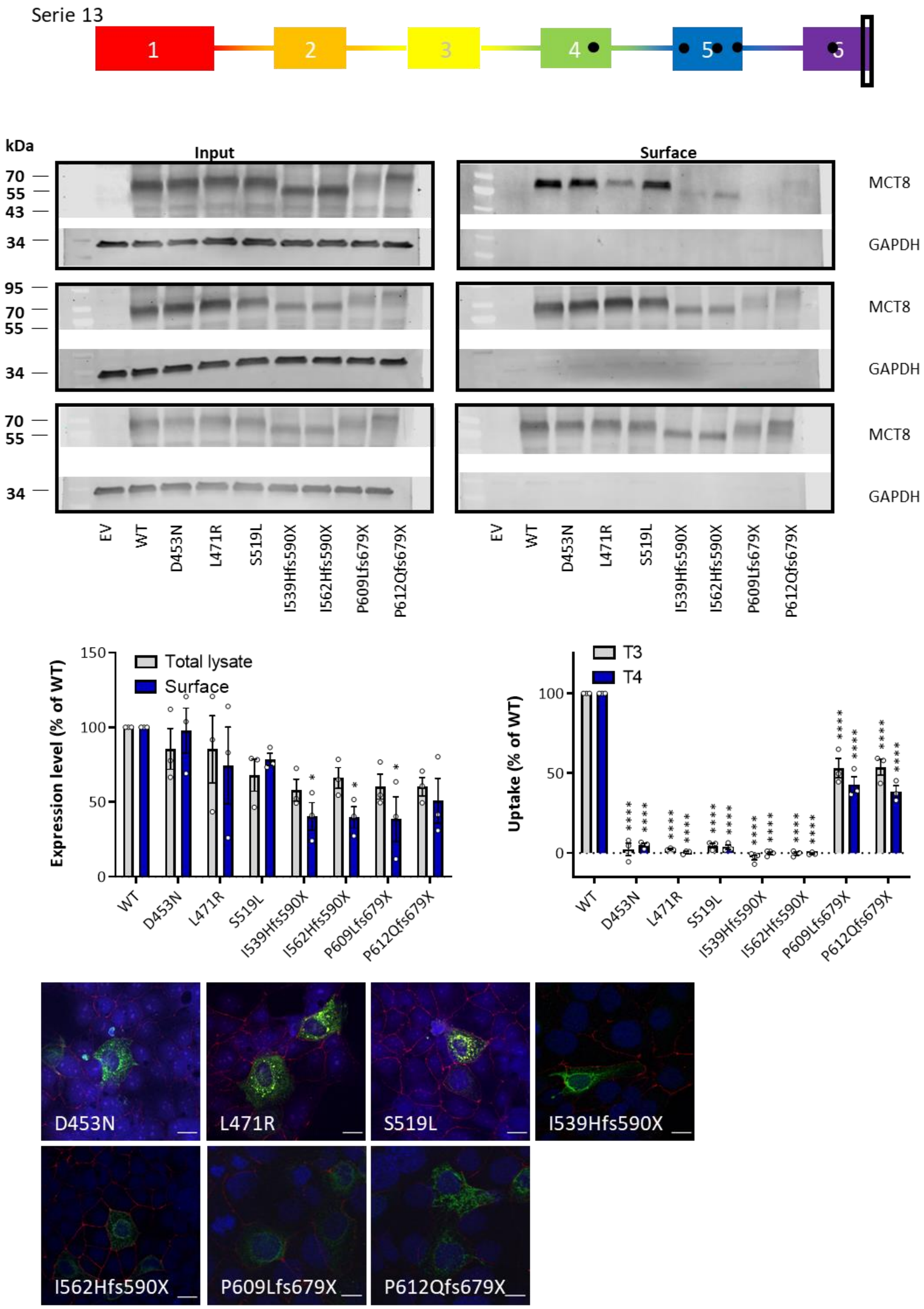
Serie 13
1
2
3
4
5
6
kDa
Input
Surface
70
55
43
34
95
MCT8
GAPDH
EV
WT
D453N
L471R
S519L
I539Hfs590X
I562Hfs590X
P609Lfs679X
P612Qfs679X
Expression level (% of WT)
Total lysate
Surface
Uptake (% of WT)
T3
T4
D453N
L471R
S519L
I539Hfs590X
I562Hfs590X
P609Lfs679X
P612Qfs679X

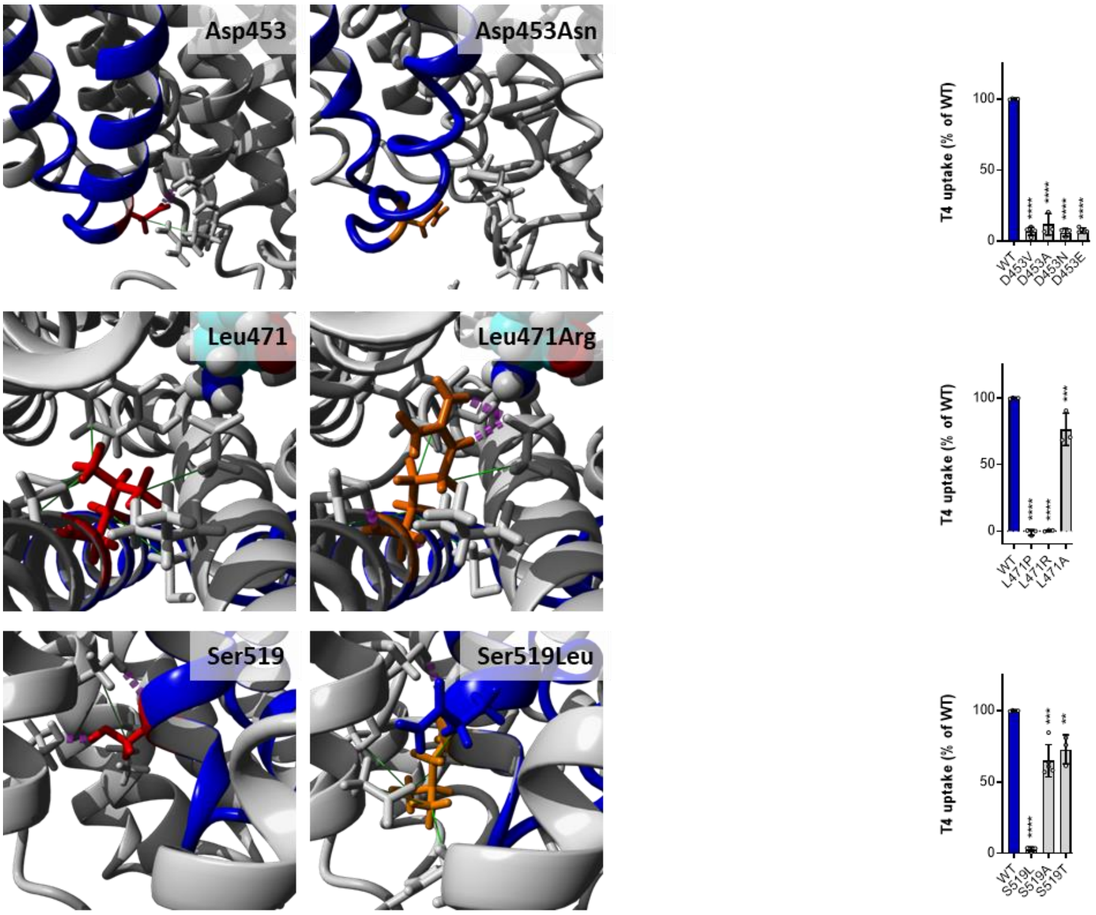
Asp453
Asp453Asn
Leu471
Leu471Arg
Ser519
Ser519Leu
T4 uptake (% of WT)
100
50
0
WT
D453V
D453A
D453N
D453E
L471P
L471R
L471A
S519L
S519A
S519T

Serie 14

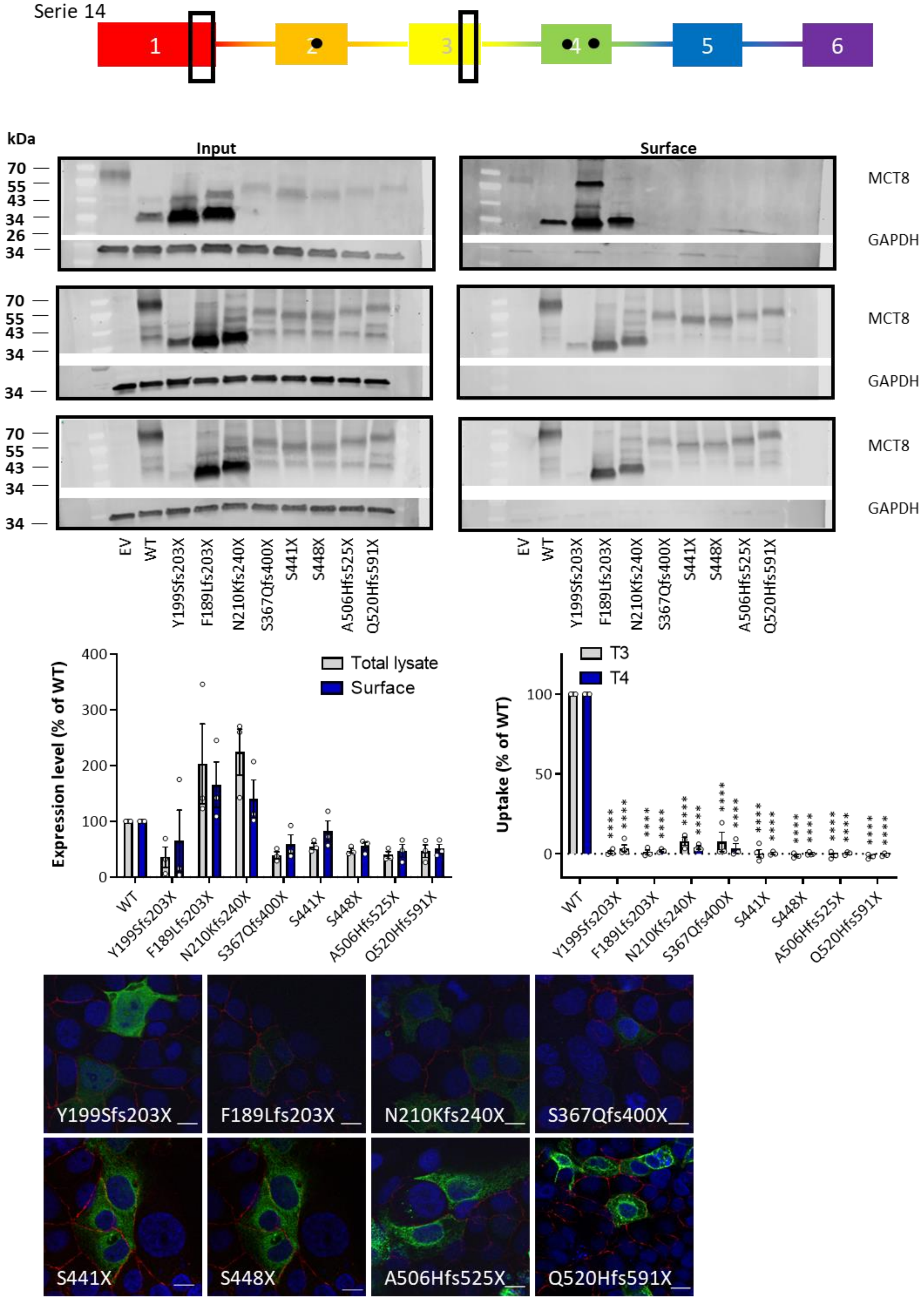

1
2
3
4
5
6
kDa
Input
Surface
70
55
43
34
26
34
MCT8
GAPDH
EV
WT
Y199Sfs203X
F189Lfs203X
N210Kfs240X
S367Qfs400X
S441X
S448X
A506Hfs525X
Q520Hfs591X
Expression level (% of WT)
400
300
200
100
0
Total lysate
Surface
Uptake (% of WT)
T3
T4
100
50
0
****
Y199Sfs203X
F189Lfs203X
N210Kfs240X
S367Qfs400X
S441X
S448X
A506Hfs525X
Q520Hfs591X

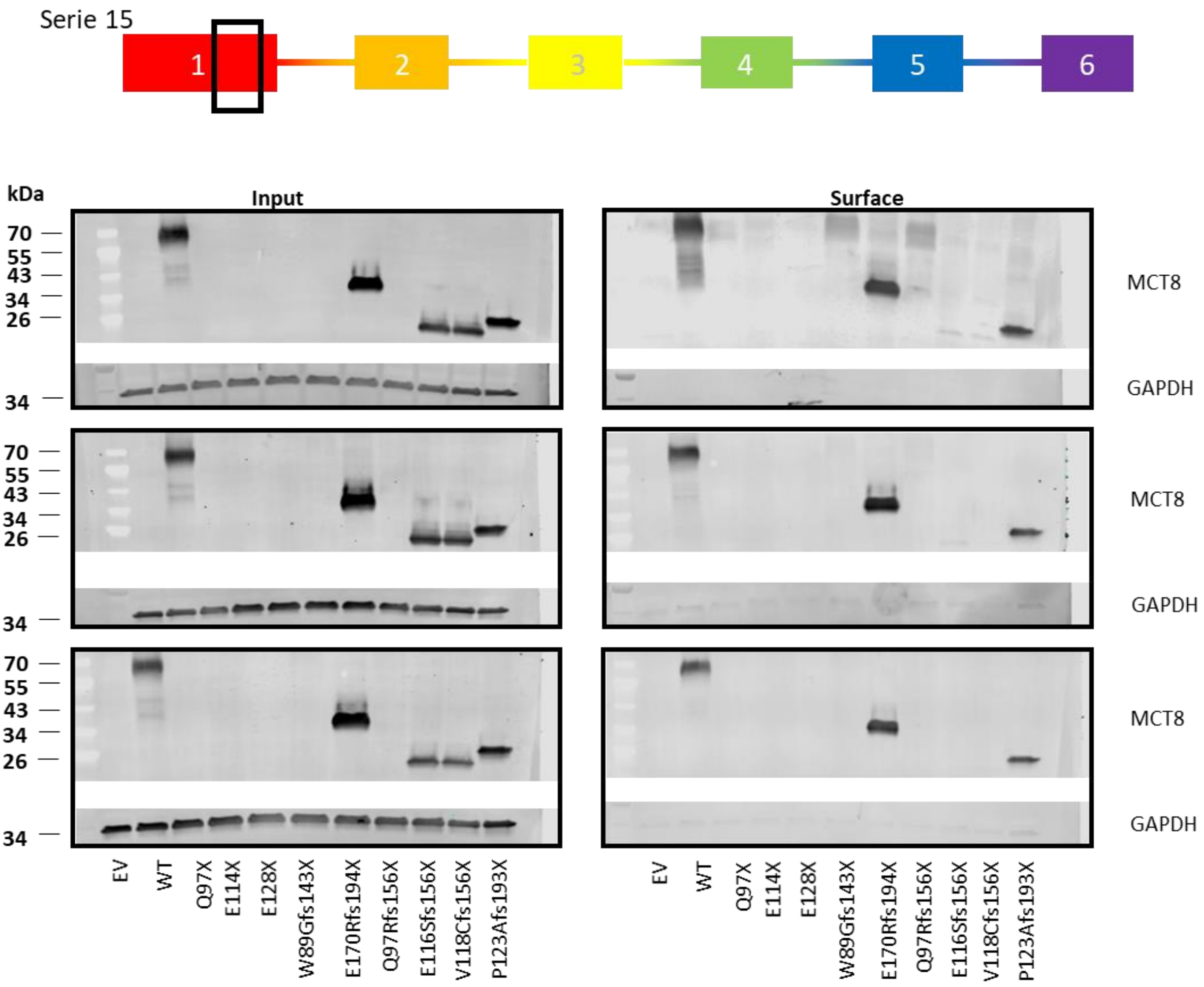

(Continues on next page)

Serie 15 (continued)

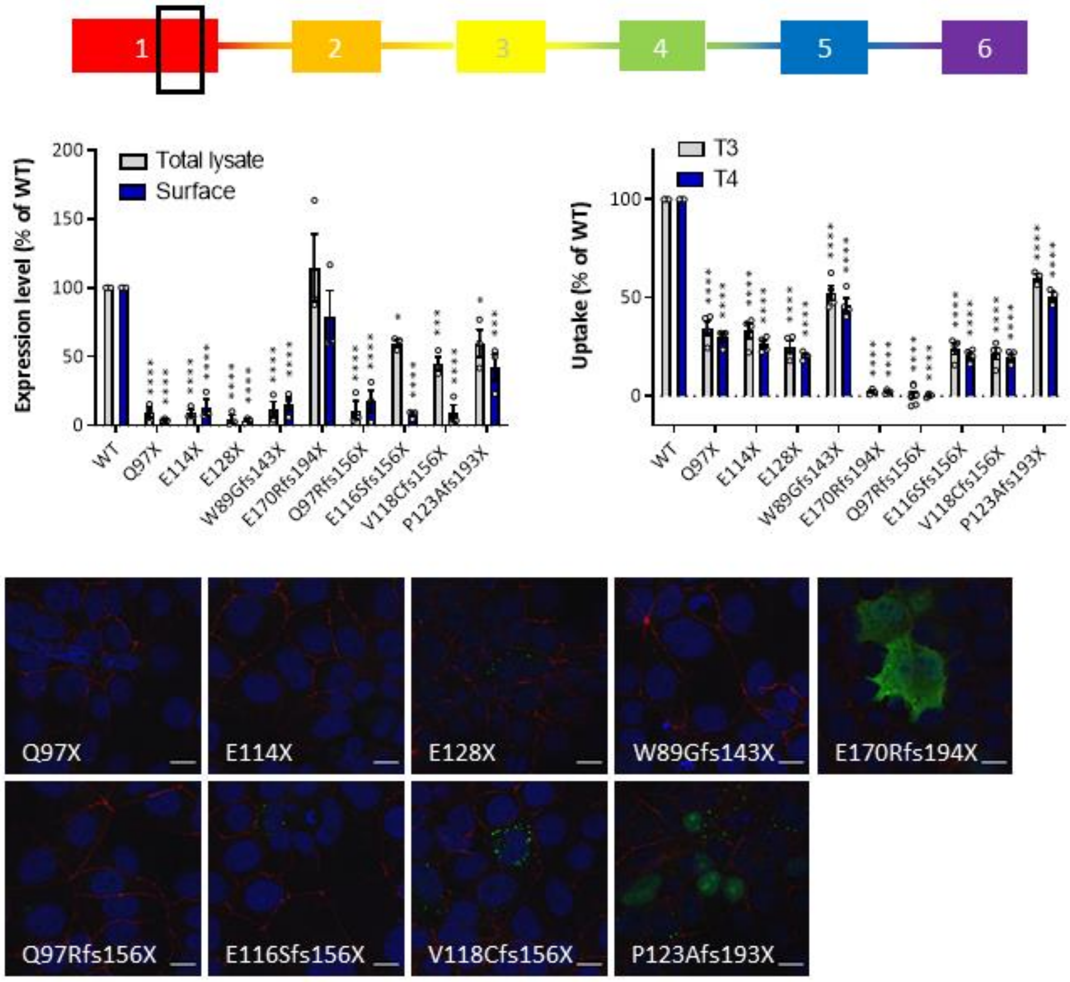

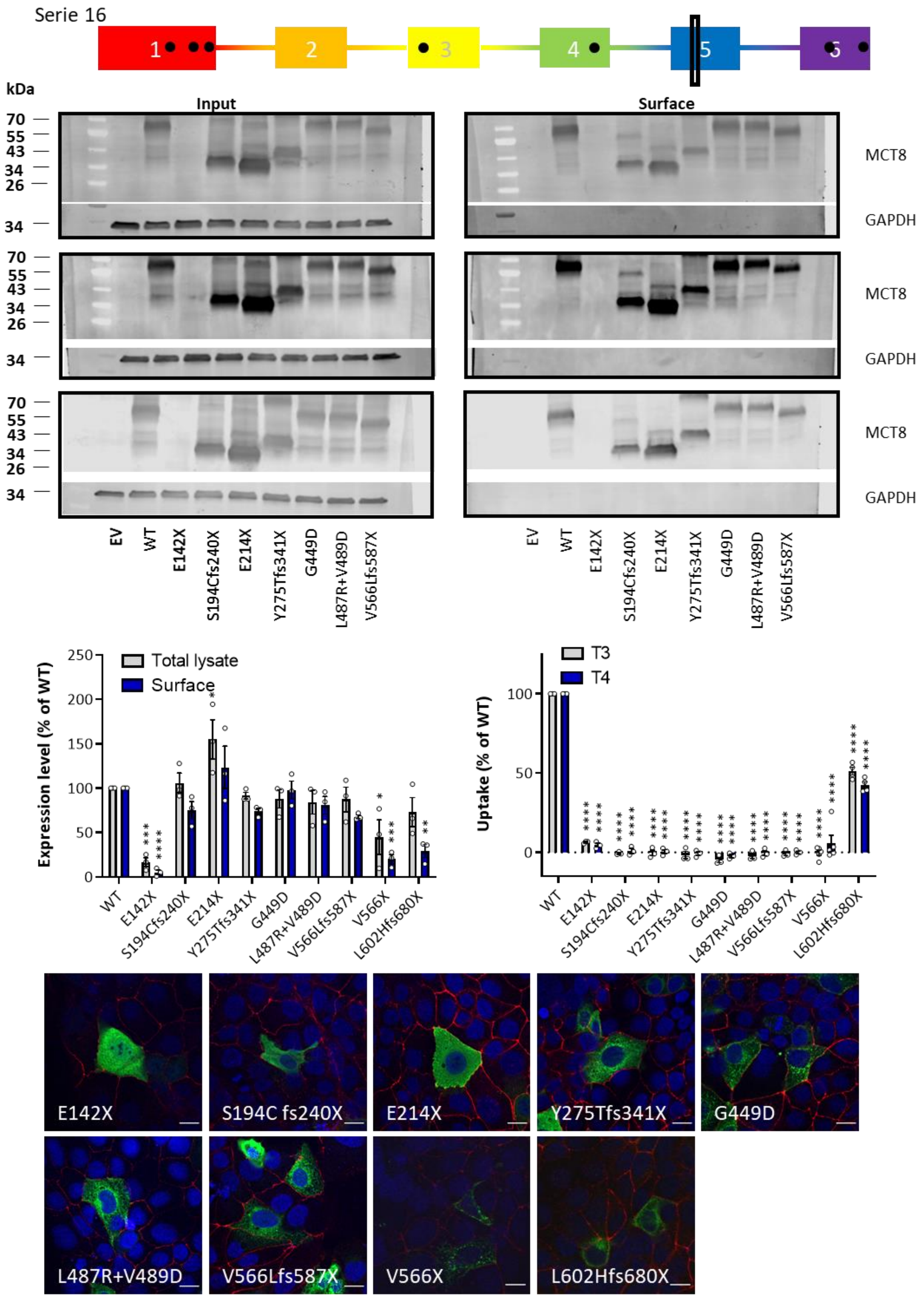
Serie 16
1
2
3
4
5
6
kDa
Input
Surface
70
55
43
34
26
34
MCT8
GAPDH
EV
WT
E142X
S194Cfs240X
E214X
Y275Tfs341X
G449D
L487R+V489D
V566Lfs587X
Total lysate
Surface
Expression level (% of WT)
250
200
150
100
50
0
V566X
L602Hfs680X
T3
T4
Uptake (% of WT)
E142X
S194C fs240X
E214X
Y275Tfs341X
G449D
L487R+V489D
V566Lfs587X
V566X
L602Hfs680X

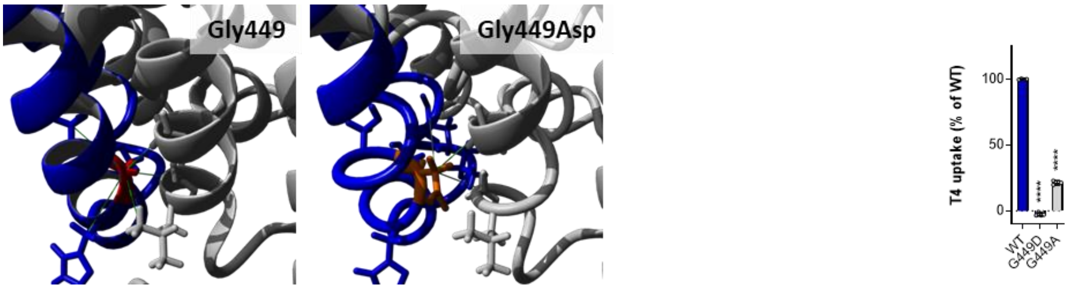

Supplementary Fig. 5. Overview of functional and structural evaluations of studied variants, including their exonic location, their expression in total lysates (n=3) and on the cell surface (including quantification, WT MCT8, set to 100% mean ±s.e.m)(n=3), residual T3 and T4 transport capacity (expressed relatively to mock-transfected cells, set to 0%, and WT MCT8, set to 100%, mean ±s.e.m.; n=3), immunocytochemistry studies(Scale bar corresponds to 15 uM), structural predictions and residual transport capacity of individual missense variants, their alanine substituents and related variants. Surface expression of V566X and L602HfsX680 (including original Western blots) was previously reported [12]. For series 1-8, the close-up panel that displays the location of the affected residues in the predictive MCT8 protein structure also highlights affected residues for which experimental data is provided in later series (9-16); such residues are indicated with an * in the close-up panels. Dashed lines in some graphs of uptake represent 0% uptake. Uptake function and (cell surface) expression levels of indicated variants were compared to WT MCT8 levels, using One-way ANOVA with Dunnett's post-hoc tests. Significance: * $p<0.05$, ** $p<0.005$, *** $p<0.0005$, **** $p<0.0001$. Exact P values are provided in Supplementary Data 1. Source data are provided as a Source Data file.

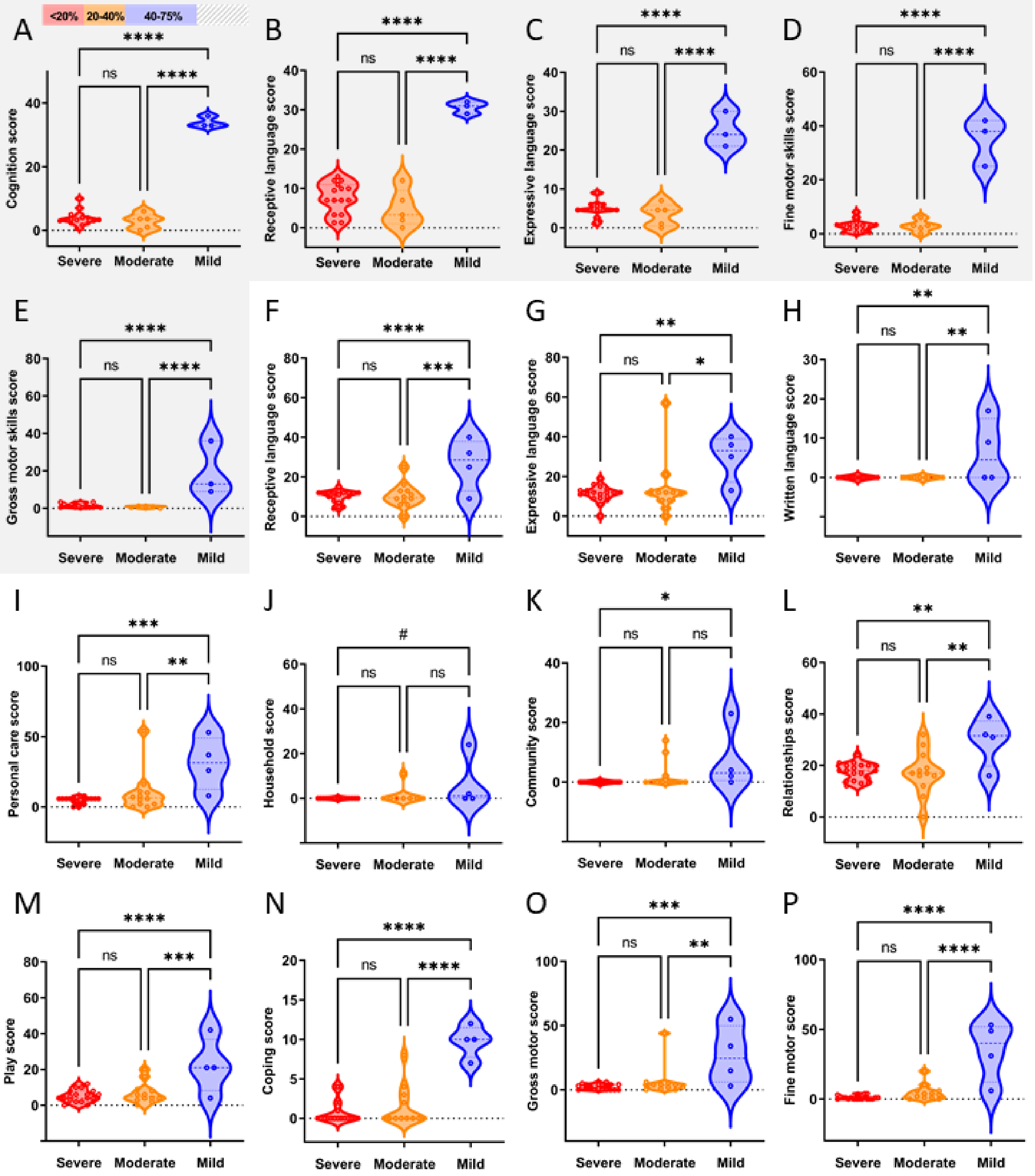

Supplementary Fig. 6. Developmental outcomes in MCT8 deficiency linked to mutant T3 transport capacity in COS-1 cells. Patients with MCT8 deficiency are stratified across different LoF classes of functional impact. Scores for sub-domains of the Bayley Scales of Infant Development (BSID) III (grey): (A) cognition, (B) receptive language, (C) expressive language, (D) fine motor skills and (E) gross motor skills and the Vineland Adaptive Behavior Scales: (F) receptive language, (G) expressive language, (H) written language, (I) personal care, (J) household, (K) community, (L) relationships, (M) play, (N) coping,

(O) gross motor and (P) fine motor. Patients younger than 2 years were excluded from analyses of all BSID-III subscales (A-E). Patients younger than 4 yours were excluded from analyses of the VABS-II Written language (H) and Household (J) subscales and patients younger than 1 year were excluded from analyses of the VABS-II Community (K) subscale. Dashed lines represent 0% score. Differences across groups were tested using One-way ANOVA with Tukey's post-hoc tests. # indicates borderline significance (p<0.1), * p<0.05, **p<0.005, ***p<0.0005, ****p<0.0001. Exact P values are provided in Supplementary Data 1. Source data are provided as a Source Data file.

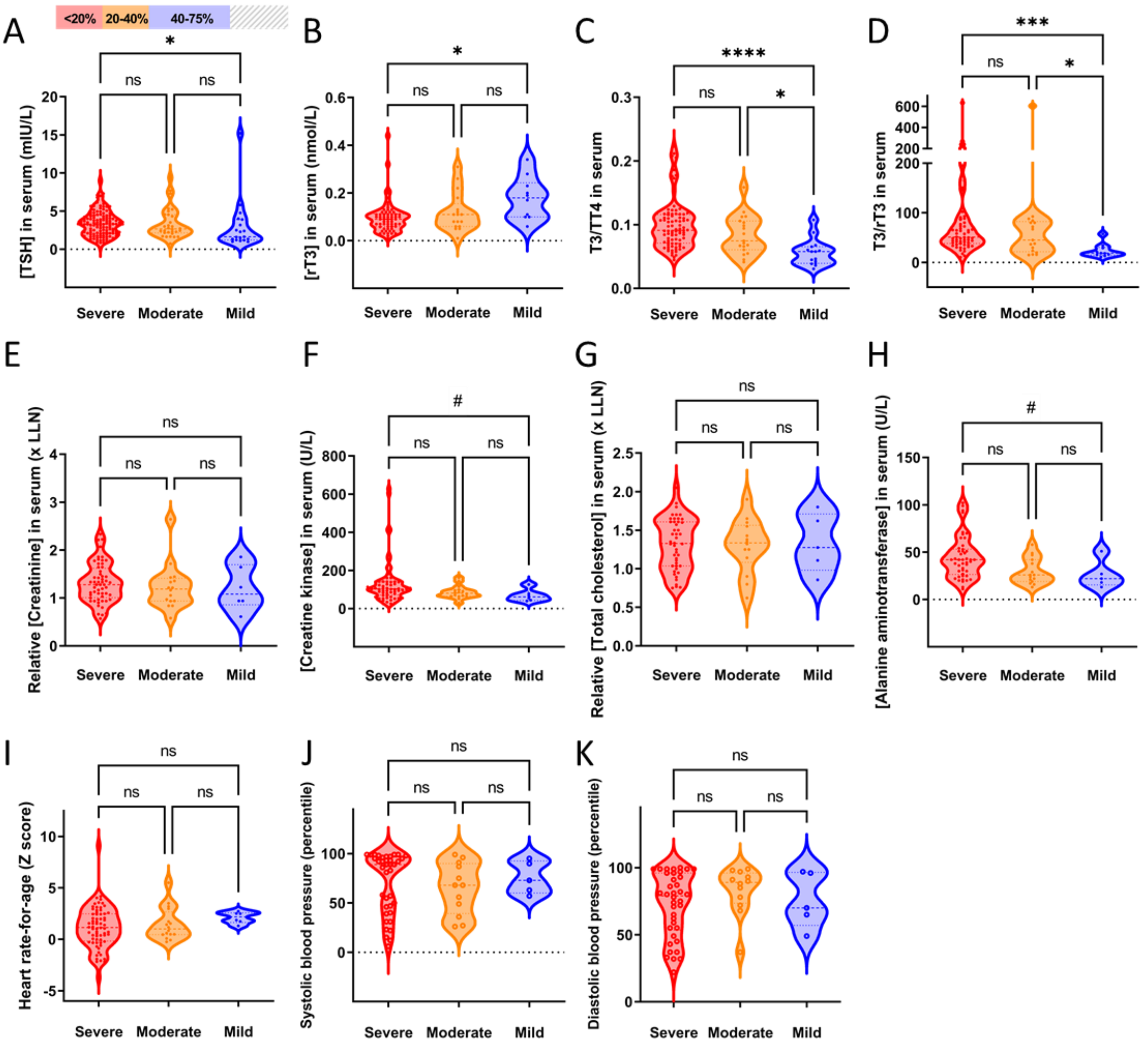

Supplementary Fig. 7. Metabolic outcomes in MCT8 deficiency linked to mutant T3 transport capacity in COS-1 cells. Patients with MCT8 deficiency are stratified across different LoF classes of functional impact. (A) Serum TSH concentration; (B) Serum rT3 concentration; (C) Serum T3:TT4 ratio; (D) Serum T3/rT3 ratio; (E) Serum creatinine concentration relative to the lower limit of normal; (F) Serum creatinine kinase concentrations; (G) Serum total cholesterol relative to the lower limit of normal; (H) Serum Alanine aminotransferase concentration; (I) Heart rate-for-age; (J) Systolic blood pressure percentile; (K) Diastolic blood pressure percentile. Dashed lines represent 0% score. Differences across groups were tested using One-way ANOVA with Tukey's post-hoc tests or Kruskal-Wallis with Dunn's multiple comparisons test. # indicates borderline significance ($p<0.1$), * $p<0.05$, ** $p<0.005$,

***p<0.0005, ****p<0.0001. Exact P values are provided in Supplementary Data 1. Source data are provided as a Source Data file.

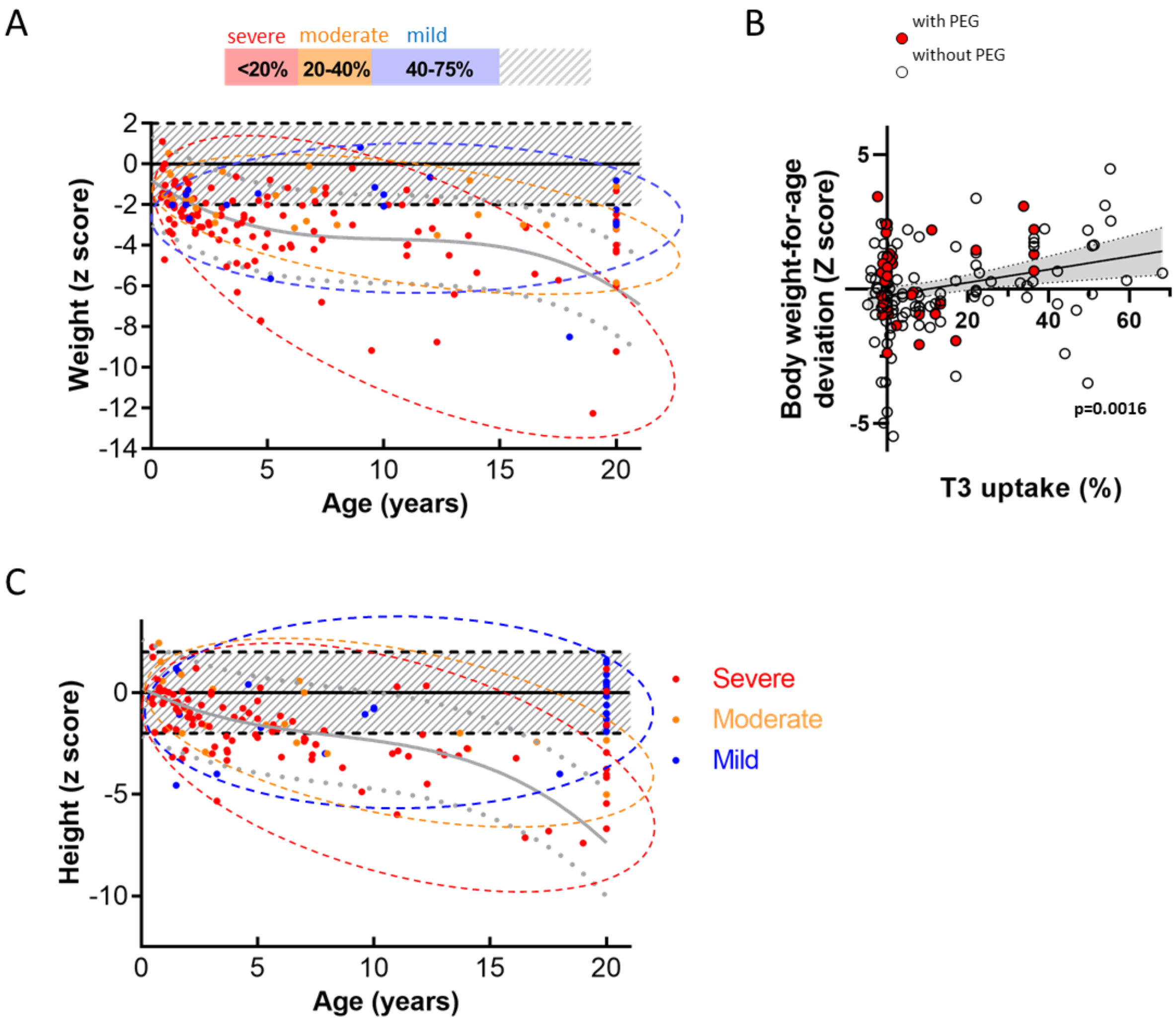

Supplementary Fig. 8. (A) Body weight-for-age with dots representing individual body weight measurements. (B) Deviation of bodyweight-for-age z-score from the expected bodyweight-for-age z-score as calculated based on available natural history data (1), with dots representing individual bodyweight measurements (red: patients with PEG-tube, white: patients without PEG-tube). (C) Body height-for-age with dots representing individual body height measurements. For A and C, non-linear (third order) polynomial regression was used to plot the trend (grey solid line) with its 95% error band (grey dashed lines); normal range indicated by shaded area. Patients aged >20 years were plotted at 20 years. Dashed ovals include >90% of data points for each functional LoF class, based on T3 uptake function in COS-1 cells. Source data are provided as a Source Data file. Exact P values are provided in Supplementary Data 1.

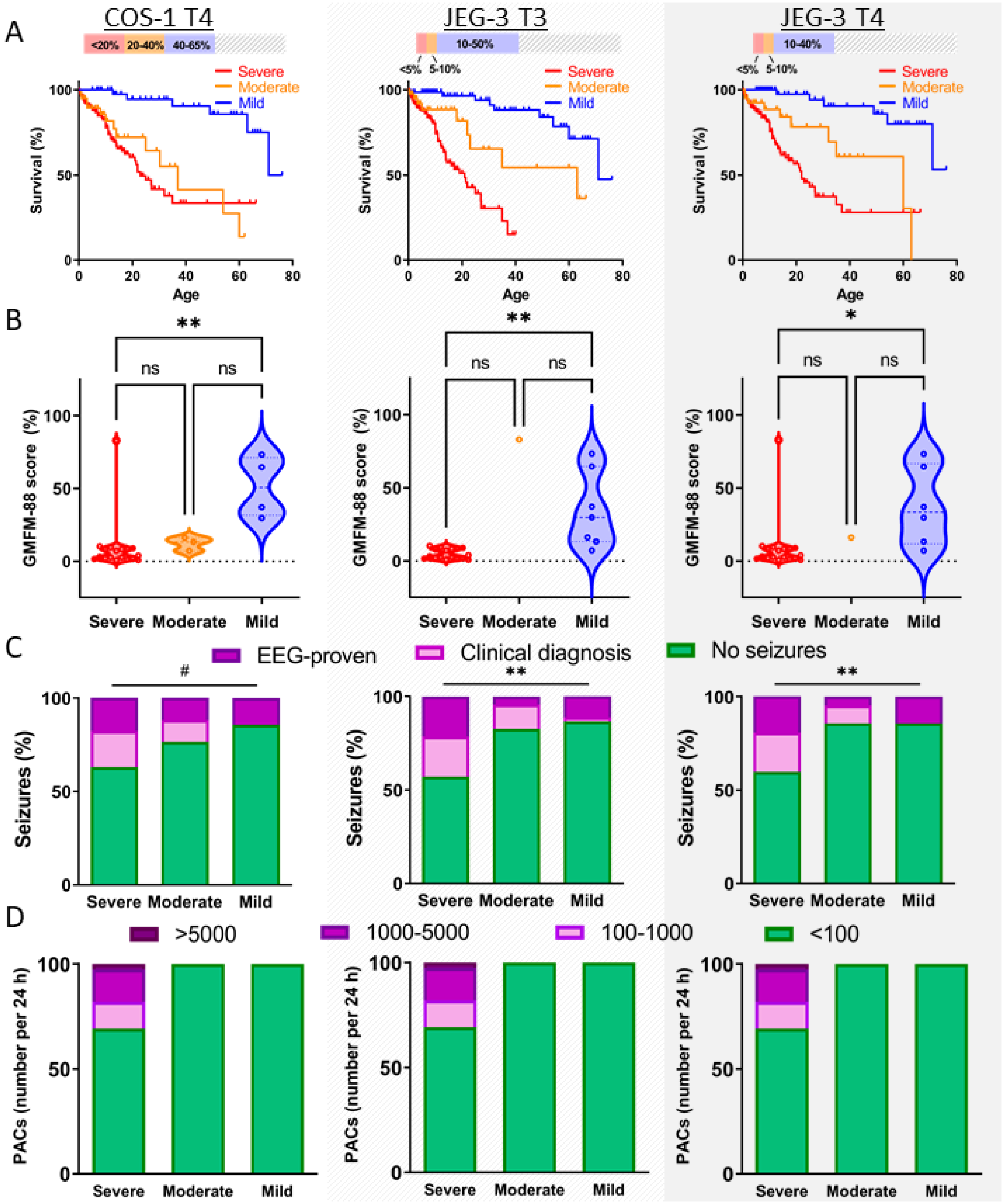

Supplementary Fig. 9. Phenotypic outcomes in MCT8 deficiency linked to mutant T3 transport capacity in different cell lines and different substrates. Patients with MCT8 deficiency are stratified across different LoF classes of functional impact. The left panel indicates T4 uptake in COS-1 cells; the middle panel indicates T3 uptake in JEG-3 cells; the right panel indicates T4 uptake in JEG-3 cells. (A) Survival; (B) GMFM-88; (C) Seizures; (D) PACs. Dashed line in (B) represents 0% score. # indicates borderline significance ($p<0.1$), * $p<0.05$, ** $p<0.005$ with Kruskal-Wallis test with Dunn's multiple comparisons

test. Source data are provided as a Source Data file. Exact P values are provided in Supplementary Data 1.

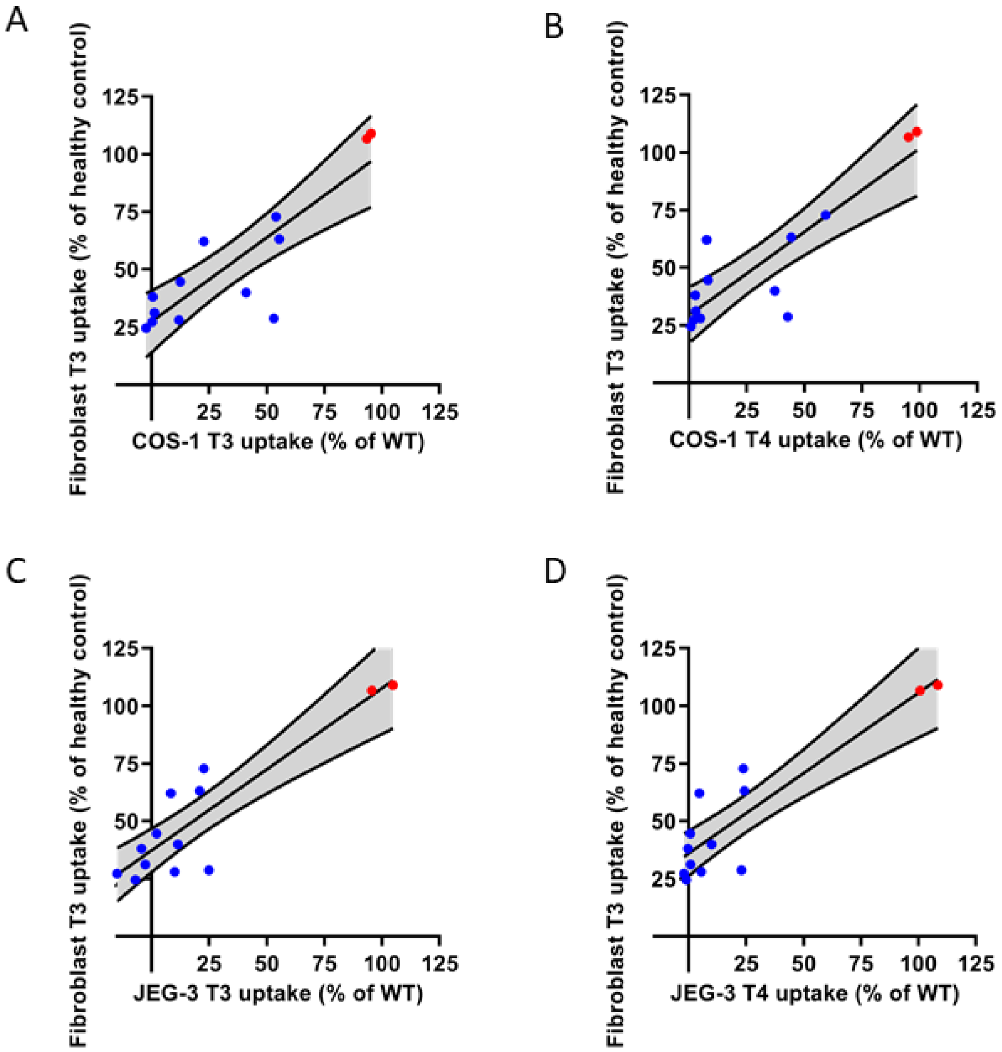

Supplementary Fig. 10. Correlation plot of accumulation of T3 (A) and T4 (B) in COS-1 cells and T3 (C) and T4 (D) in JEG-3 cells with accumulation of T3 in patient-derived fibroblasts. Blue dots represent uptake values of pathogenic variants associated with a clinical phenotype typical of MCT8 deficiency, that segregated within families as an X-linked disorder. Red dots represent values of known benign variants in MCT8, judged on the absence of a clinical phenotype, or incompatible mode of segregation. The lines represent the mean with the 95% confidence intervals. Source data are provided as a Source Data file.

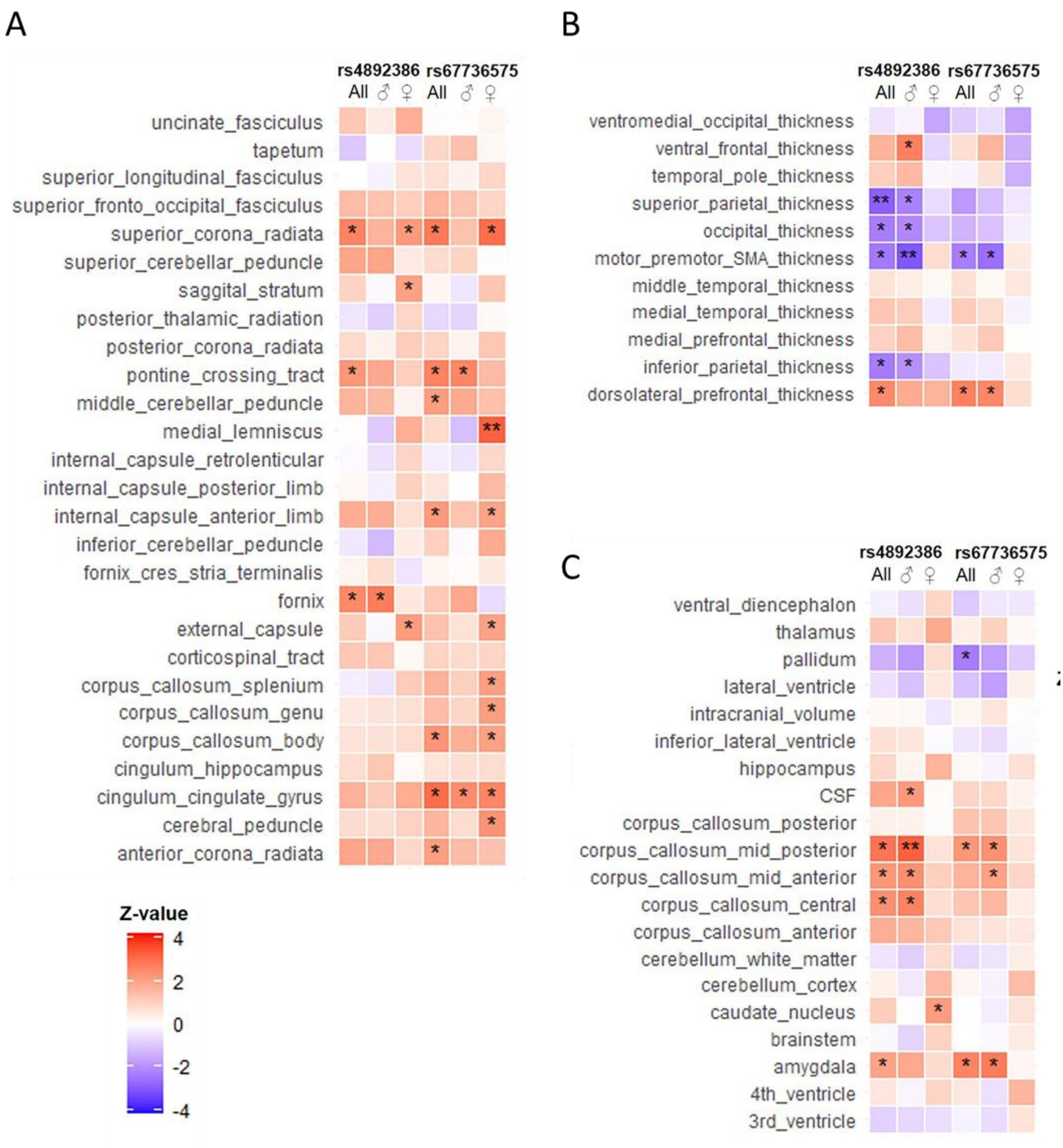
A
rs4892386
rs67736575
All ♂ ♀ All ♂ ♀
uncinate_fasciculus
tapetum
superior_longitudinal_fasciculus
superior_fronto_occipital_fasciculus
superior_corona_radiata
superior_cerebellar_peduncle
saggital_stratum
posterior_thalamic_radiation
posterior_corona_radiata
pontine_crossing_tract
middle_cerebellar_peduncle
medial_lemniscus
internal_capsule_retrolenticular
internal_capsule_posterior_limb
internal_capsule_anterior_limb
inferior_cerebellar_peduncle
fornix_cres_stria_terminalis
fornix
external_capsule
corticospinal_tract
corpus_callosum_splenium
corpus_callosum_genu
corpus_callosum_body
cingulum_hippocampus
cingulum_cingulate_gyrus
cerebral_peduncle
anterior_corona_radiata
Z-value
4
2
0
-2
-4
B
rs4892386
rs67736575
All ♂ ♀ All ♂ ♀
ventromedial_occipital_thickness
ventral_frontal_thickness
temporal_pole_thickness
superior_parietal_thickness
occipital_thickness
motor_premotor_SMA_thickness
middle_temporal_thickness
medial_temporal_thickness
medial_prefrontal_thickness
inferior_parietal_thickness
dorsolateral_prefrontal_thickness
C
rs4892386
rs67736575
All ♂ ♀ All ♂ ♀
ventral_diencephalon
thalamus
pallidum
lateral_ventricle
intracranial_volume
inferior_lateral_ventricle
hippocampus
CSF
corpus_callosum_posterior
corpus_callosum_mid_posterior
corpus_callosum_mid_anterior
corpus_callosum_central
corpus_callosum_anterior
cerebellum_white_matter
cerebellum_cortex
caudate_nucleus
brainstem
amygdala
4th_ventricle
3rd_ventricle

Supplementary Fig. 11. Lookup of SNPS mapping to SLC16A2 in brain outcomes. Association of rs4892386 and rs67736575 and specific brain MRI phenotypes: fiber tract fractional anisotropy (A), cortical thickness (B), subcortical volumes (C) in participants of the UK Biobank were assessed using an additive model, in sex-specific and joint analyses adjusting for age, Euler number, 10 genomic principal components, BrainDx , and global brain measures (mean cortical thickness and total surface area for thickness and area phenotypes, respectively). Multiple-testing adjustment was applied at 3 levels, nominal P<0.05, denoted by *; categorical, denoted by ** (i.e., Specific brain thickness 11 traits*2 SNPs P<0.0023. Subcortical volumes 20 traits* 2 SNPs P<0.00125. Fiber tract anisotropy, 58 traits* 2 SNPs P<0.0009); and study-wise, denoted by ***, 18 traits *2 SNPs; P<0.0004. Exact P values are provided in Supplementary Table 5.

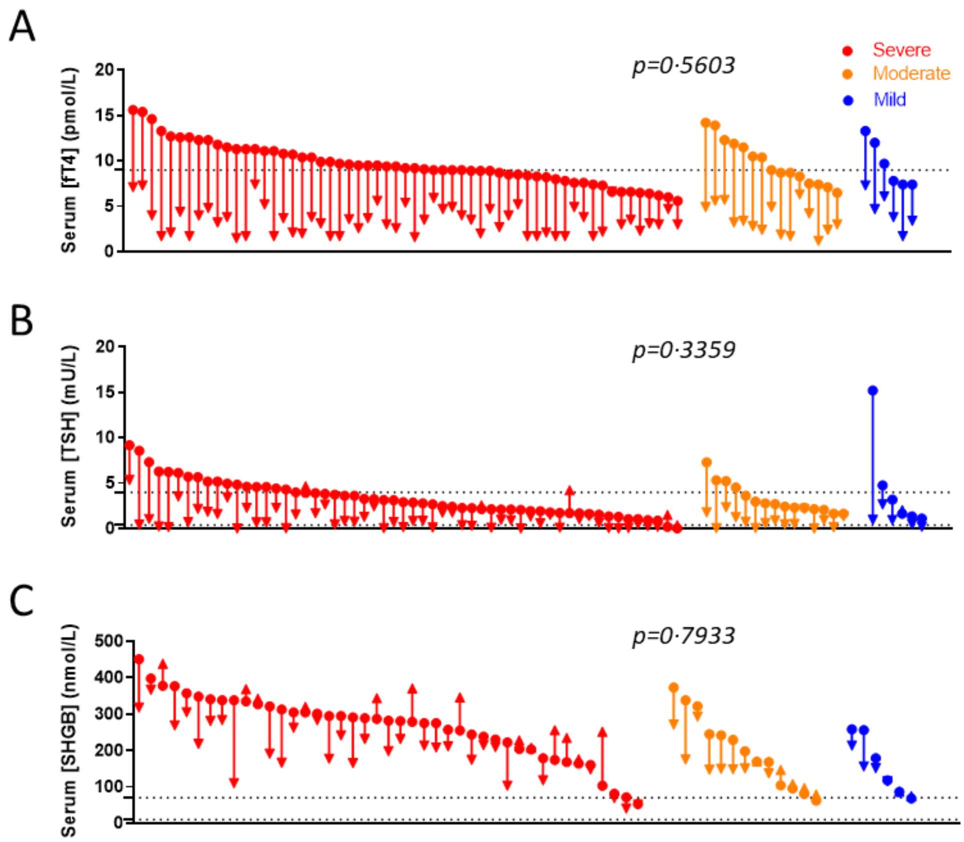

Supplementary Fig. 12. Disease outcomes in patients treated with Triac. Changes from baseline to last available follow-up visit in serum concentrations of FT4 (A), TSH (B) and SHBG (C). Dashed lines represent reference intervals (and lower limit of normal in (A)). Source data are provided as a Source Data file.

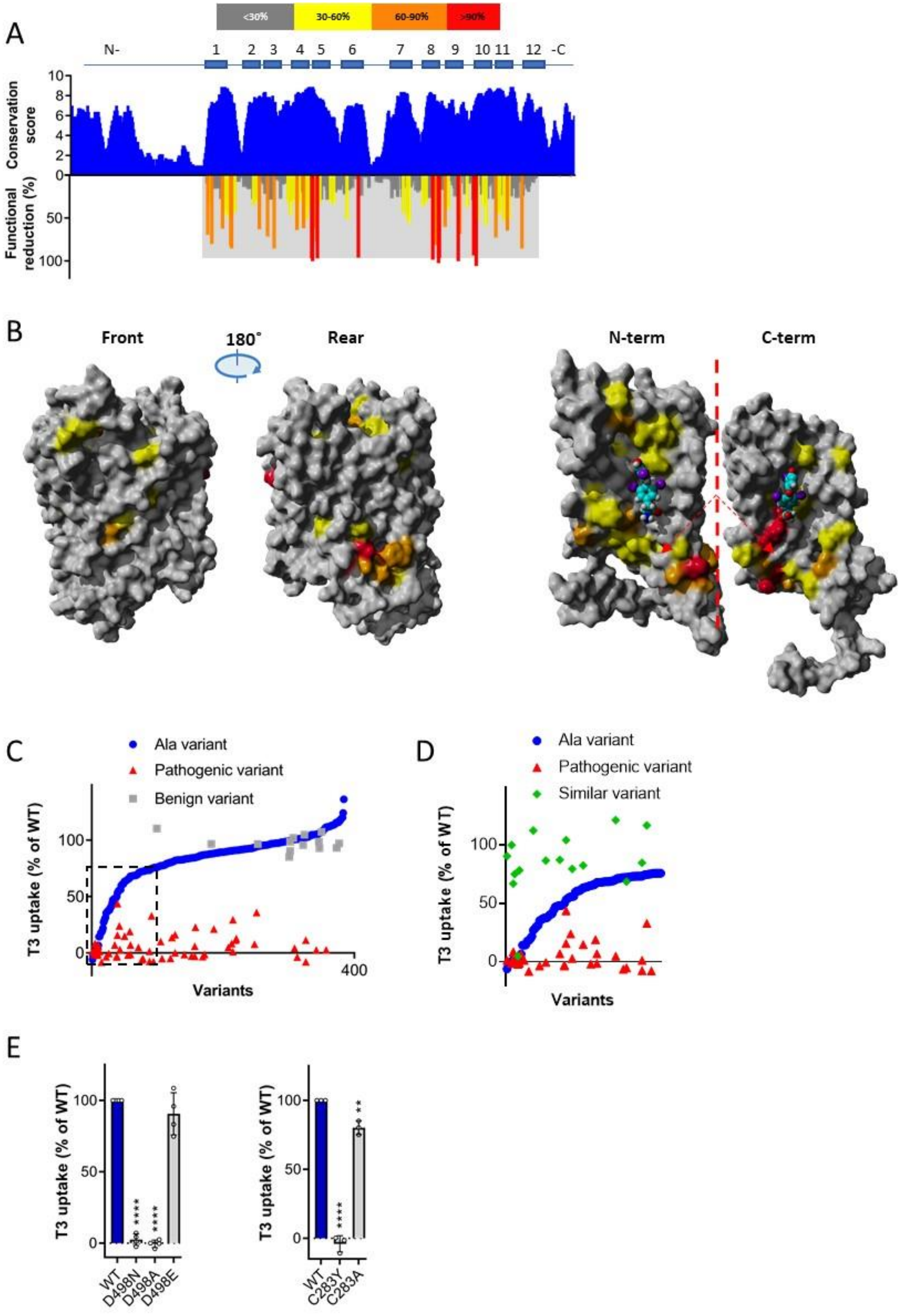
A
<30%
30-60%
60-90%
>90%
N-
1 2 3 4 5 6 7 8 9 10 11 12 -C
Conservation score
Functional reduction (%)
B
Front
180°
Rear
N-term
C-term
C
Ala variant
Pathogenic variant
Benign variant
T3 uptake (% of WT)
Variants
D
Ala variant
Pathogenic variant
Similar variant
T3 uptake (% of WT)
Variants
E
T3 uptake (% of WT)
WT
D498N
D498A
D498E
T3 uptake (% of WT)
WT
C283Y
C283A

Supplementary Fig. 13. (A) (upper part) Conservation analysis of human MCT8 with 73 species. (lower part) T3 transport capacity of all MCT8 Ala variants shown as functional impact in JEG-3 cells. (red: >90-100%, orange: 90-60%, yellow: 30-60%, grey: 0-30% functional reduction with WT MCT8 set as 0% impact). Grey box indicates boundaries for Ala scanning (Pro169 – His575).

(B) Color-coded mapping (see A) of functional impact identified through alanine scanning onto the MCT8 homology structure in outward-open conformation. (left panel) frontal view and rear view of MCT8 and (right panel) inside views (vertical section of frontal view) of the N-terminal (left) and C-terminal (right) halves of the MCT8 protein.

(C) T3 uptake capacity of all tested Ala variants (blue; ranked from 0% to 100% transport capacity), LoF patient variants (red), and benign non-synonymous missense variants (grey). Patients variants affecting residues within the dashed box are likely pathogenic due to the loss of a critical native residue, whereas patient variants affecting other residue are likely pathogenic due to the introduction of an unfavourable residue.

(D) T3 uptake capacity in selected panel (dashed box in Fig. **3c**) of Ala variants (blue), LoF patient variants (red), and artificial variants, where the native residue at the position of patient variant was replaced by a residue with similar properties (dark grey).

(E) T3 transport capacity (mean ± s.e.m.) in JEG-3 cells expressing WT (set as 100%) or mutant MCT8. P values were calculated using One-way ANOVA with Dunnet's multiple comparisons post-test; **p<0.005, **** p<0.0001. Exact P values are provided in Supplementary Data 1. Source data are provided as a Source Data file.

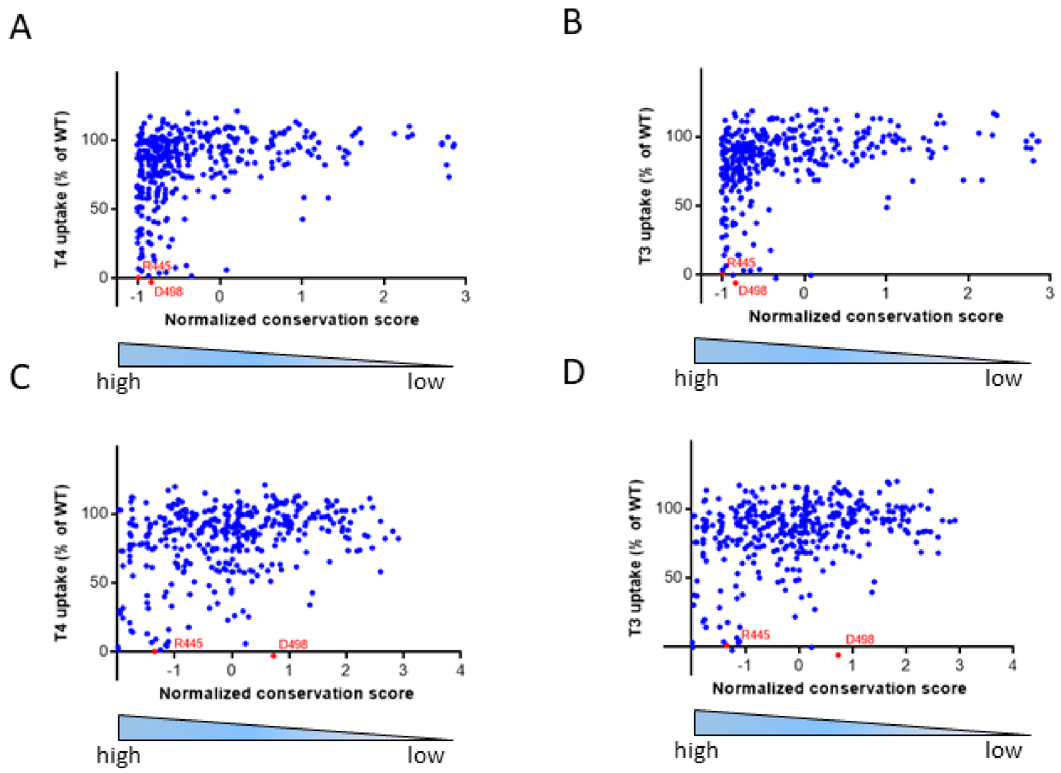

Supplementary Fig. 14. Thyroid hormone transport capacity and conservations scores. (A) T4 uptake vs conservation score across MCT8 in other species (all); (B) T3 uptake vs conservation score across MCT8 in other species (all); (C) T4 uptake vs conservation score across human MCT-family members; (D) T3 uptake vs conservation score across human MCT-family members. Arg445 and Asp498 are plotted as exemplary critical residues. Source data are provided as a Source Data file.

```
hsMCT8     MALQSQASEEAKGPWQEADQEQQEPVGSPEPESEPEPEPEPEPVPVPPPEPQPEPQPLPD   60
hsMCT10    MVLSQEEPDSARGT------SEAQPLGP------------------APT--GAAPPPGPG   34
hsMCT9     ------------------------------------------------------------   0
hsMCT14    ------------------------------------------------------MYTSH-   5
hsMCT5     ------------------------------------------------------------   0
hsMCT12    -----------------------------------------------------MPSGSHW   7
hsMCT6     ------------------------------------------------------------   0
hsMCT1     ------------------------------------------------------------   0
hsMCT2     ------------------------------------------------------------   0
hsMCT3     ------------------------------------------------------------   0
hsMCT4     ------------------------------------------------------------   0
hsMCT7     -----------------------------------------------------MTQN---   4
hsMCT11    ---------------------------------------------------MPAPQRKHR   9
hsMCT13    ------------------------------------------------------------   0


hsMCT8     PAPL-PELEFESERVHEPEPTPTVETRGTARGFQPPEGGFGWVVVFAATWCNGSIFGIHN   119
hsMCT10    PSDS-PEAAVEKVEVELAGP----ATAEPHEPPEPPEGGWGWLVMLAAMWCNGSVFGIQN   89
hsMCT9     -----------------------------MELKKSPDGGWGWVIVFVSFLTQFLCYGSPL   31
hsMCT14    -----EDIGYD--------FEDGPKDKKTLKPHPNIDGGWAWMMVLSSFFVHILIMGSQM   52
hsMCT5     ---------------------MLKREGKVQPYTKTLDGGWGWMIVIHFFLVNVFVMGMTK   39
hsMCT12    TANSSKIITWLLEQPGKEEKRKTMAKVNRARSTSPPDGGWGWMIVAGCFLVTICTRAVTR   67
hsMCT6     ----------------------------MPQALERADGSWAWVVLLATMVTQGLTLGFPT   32
hsMCT1     ----------------------MPPAVGGPVGYTPPDGGWGWAVVIGAFISIGFSYAFPK   38
hsMCT2     ----------------------MPPMPSAPPVHPPPDGGWGWIVVGAAFISIGFSYAFPK   38
hsMCT3     --------------------MGA---GGPRRGEGPPDGGWGWVVLGACFVVTGFAYGFPK   37
hsMCT4     --------------------MGGAVVDEGPTGVKAPDGGWGWAVLFGCFVITGFSYAFPK   40
hsMCT7     ---------------------KLKLCSKANVYTEVPDGGWGWAVAVSFFFVEVFTYGIIK   43
hsMCT11    RGGF------------SHRCFPTPQTAMTPQPAGPPDGGWGWVVAAAAFAINGLSYGLLR   57
hsMCT13    ----------------------------MARRTEPPDGGWGWVVVLSAFFQSALVFGVLR   32
                                               :*.:.* :            .

hsMCT8     SVGILYSMLLEEEK-EKNRQVEFQAAWVGALAMGMIFFCSPIVSIFTDRLGCRITATAGA   178
hsMCT10    ACGVLFVSMLETFGSKDDDKMVFKTAWVGSLSMGMIFFCCPIVSVFTDLFGCRKTAVVGA   149
hsMCT9     AVGVLYIEWLDAFGEGK-----GKTAWVGSLASGVGLLASPVCSLCVSSFGARPVTIFSG   86
hsMCT14    ALGVLNVEWLEEFHQSR-----GLTAWVSSLSMGITLIVGPFIGLFINTCGCRQTAIIGG   107
hsMCT5     TFAIFFVVFQEEFEGTS-----EQIGWIGSIMSSLRFCAGPLVAIICDILGEKTTSILGA   94
hsMCT12    CISIFFVEFQTYFTQDY-----AQTAWIHSIVDCVTMLCAPLGSVVSNHLSCQVGIMLGG   122
hsMCT6     CIGIFFTELQWEFQASN-----SETSWFPSILTAVLHMAGPLCSILVGRFGCRVTVMLGG   87
hsMCT1     SITVFFKEIEGIFHATT-----SEVSWISSIMLAVMYGGGPISSILVNKYGSRIVMIVGG   93
hsMCT2     AVTVFFKEIQQIFHTTY-----SEIAWISSIMLAVMYAGGPVSSVLVNKYGSRPVVIAGG   93
hsMCT3     AVSVFFRALMRDFDAGY-----SDTAWVSSIMLAMLYGTGPVSSILVTRFGCRPVMLAGG   92
hsMCT4     AVSVFFKELIQEFGIGY-----SDTAWISSILLAMLYGTGPLCSVCVNRFGCRPVMLVGG   95
hsMCT7     TFGVFFNDLMDSFNESN-----SRISWIISICVFVLTFSAPLATVLSNRFGHRLVVMLGG   98
hsMCT11    SLGLAFPDLAEHFDRSA-----QDTAWISALALAVQQAASPVGSALSTRWGARPVVMVGG   112
hsMCT13    SFGVFFVEFVAAFEEQA-----ARVSWIASIGIAVQQFGSPVGSALSTKFGPRPVVMTGG   87
              :                     .*. ::   :     *.        . :     ..

hsMCT8     AVAFIGLHTSSFTSSLSLRYFTYGILFGCGCSFAFQPSLVILGHYFQRRLGLANGVVSAG   238
hsMCT10    AVGFVGLMSSSFVSSIEPLYLTYGIIFACGCSFAYQPSLVILGHYFKKRLGLVNGIVTAG   209
hsMCT9     FMVAGGLMLSSFAPNIYFLFFSYGIVVGLGCGLLYTATVTITCQYFDDRRGLALGLISTG   146
hsMCT14    LVNSLGWVLSAYAANVHYLFITFGVAAGLGSGMAYLPAVVMVGRYFQKRRALAQGLSTTG   167
hsMCT5     FVVTGGYLISSWATSIPFLCVTMGLLPGLGSAFLYQVAAVVTTKYFKKRLALSTAIARSG   154
hsMCT12    LLASTGLILSSFATSLKHLYLTLGVLTGLGFALCYSPAIAMVGKYFSRRKALAYGIAMSG   182
hsMCT6     VLASLGMVASSFSHNLSQLYFTAGFITGLGMCFSFQSSITVLGFYFVRRRVLANALASMG   147
hsMCT1     CLSGCGLIAASFCNTVQQLYVCIGVIGGLGLAFNLNPALTMIGKYFYKRRPLANGLAMAG   153
hsMCT2     LLCCLGMVLASFSSSVVQLYLTMGFITGLGLAFNLQPALTIIGKYFYRKRPMANGLAMAG   153
hsMCT3     LLASAGMILASFATRLLELYLTAGVLTGLGLALNFQPSLIMLGLYFERRRPLANGLAAAG   152
hsMCT4     LFASLGMVAASFCRSIIQVYLTTGVITGLGLALNFQPSLIMLNRYFSKRRPMANGLAAAG   155
hsMCT7     LLVSTGMVAASFSQEVSHMYVAIGIISGLGYCFSFLPTVTILSQYFGKRRSIVTAVASTG   158
hsMCT11    VLASLGFVFSAFASDLLHLYLGLGLLAGFGWALVFAPALGTLSRYFSRRRVLAVGLALTG   172
hsMCT13    ILAALGMLLASFATSLTHLYLSIGLLSGSGWALTFAPTLACLSCYFSRRRSLATGLALTG   147
            .   *   :::   :    .  *.  . *  :    :      **  :  :  .:   *
```

```
hsMCT8          SSIFSMSFPFLIRMLGDKIKLAQTFQVLSTFMFVLMLLSLTYRPLLPSSQDTPSKR-GVR   297
hsMCT10         SSVFTILLPLLLRVLIDSVGLFYTLRVLCIFMFVLFLAGFTYRPLATSTKDKESGGSGSS   269
hsMCT9          SSVGLFIYAALQRMLVEFYGLDGCLLIVGALALNILACGSLMRPLQSSDCPLPKKI-APE   205
hsMCT14         TGFGTFLMTVLLKYLCAEYGWRNAMLIQGAVSLNLCVCGALMRPLSPGKNPNDPGEKDVR   227
hsMCT5          MGL-TFLLAPFTKFLIDLYDWTGALILFGAIALNLVPSSMLLRPIHIKSENNSGIKDKGS   213
hsMCT12         SGIGTFILAPVVQLLIEQFSWRGALLILGGFVLNLCVCGALMRPITLKEDHTTPEQNHVC   242
hsMCT6          VSLGITLWPLLSRYLLENLGWRGTFLVFGGIFLHCCICGAIIRPVATSVAPETKECPPPP   207
hsMCT1          SPVFLCTLAPLNQVFFGIFGWRGSFLILGGLLLNCCVAGALMRPIGPKPTKAGKDKSKAS   213
hsMCT2          SPVFLSSLAPFNQYLFNTFGWKGSFLILGSLLLNACVAGSLMRPLGPNQTTSKSKNKTGK   213
hsMCT3          SPVFLSALSPLGQQLLERFGWRGGFLLLGGLLLHCCACGAVMRPPPGPGPRPRRDSAG--   210
hsMCT4          SPVFLCALSPLGQLLQDRYGWRGGFLILGGLLLNCCVCAALMRPLVVTAQ----------   205
hsMCT7          ECFAVFAFAPAIMALKERIGWRYSLLFVGLLQLNIVIFGALLRPIFIRGPASPKIVIQEN   218
hsMCT11         NGASSLLLAPALQLLLDTFGWRGALLLLGAITLHLTPCGALLLPLVLPGDPPAP------   226
hsMCT13         VGLSSFTFAPFFQWLLSHYAWRGSLLLVSALSLHLVACGALLRPPSLAEDPAVG------   201
                              :         : .    . :      .    *

hsMCT8          TLH---------------------------------------------------------   300
hsMCT10         LFS---------------------------------------------------------   272
hsMCT9          DLPDKYS----IYNEKGKNLEENINILDKSYSSEEKCRITLANGDWKQDSLLHKNPTVTH   261
hsMCT14         GLPAHSTESVKSTGQQGRTEEKDGGLG----NEETLCDLQA-----------QECPDQAG   272
hsMCT5          SLSAHGPEAH----ATETHCHETEESTI---KDSTTQKAGLPSKNLTVSQNQSEEFYNGP   266
hsMCT12         RTQ--------------------------KED----------------IKRVSPYSSL   258
hsMCT6          PET---------------------------------------------------------   210
hsMCT1          LEKAGK------------------SGV---KKD----------------LHDAN--TDL   233
hsMCT2          TE--------------------------------------------------------DD   217
hsMCT3          -DRAGD------------------APG---EAE----------------------ADG   224
hsMCT4          ------------------------------------------------------------   205
hsMCT7          RKEAQYMLEN----EKTRTSIDSIDSGV---ELTTS------PKNVPTHTNLELEPKADM   265
hsMCT11         ------------------------------------------------------------   226
hsMCT13         ------------------------------------------------------------   201


hsMCT8          -Q--------------RFLAQLRK---------YFNMRVF-RQRTYRIWAFG-IAAAALG   334
hsMCT10         -R--------------KKFSPPKK---------IFNFAIF-KVTAYAVWAVG-IPLALFG   306
hsMCT9          TKEPETYKKKVAEQTYFCKQLAKRKWQLYKNYCGETVALF-KNKVFSALFIAILLFDIGG   320
hsMCT14         HRKNMCALRILKTVSWLTMRVRKGFEDWYSGYFGT-ASLF-TNRMFVAFIFWA-LFAYSS   329
hsMCT5          NR-NRLLLKSDEESDKVISWSCKQ---------LFDISLF-RNPFFYIFTWS-FLLSQLA   314
hsMCT12         TK--EWAQT----CLCCCLQQEYS---------FL------LMSDFVVLAVS-VLFMAYG   296
hsMCT6          -----PALG----CLAACGRTIQR---------HLAFDILRHNTGYCVYILG-VMWSVLG   251
hsMCT1          IG--RHPKQ----EKRSVFQTINQ---------FLDLTLF-THRGFLLYLSG-NVIMFFG   276
hsMCT2          SS--PKKIK----TKKSTWEKVNK---------YLDFSLF-KHRGFLIYLSG-NVIMFLG   260
hsMCT3          A---GLQLR----EASPWVRPRRR---------LLDLAVC-TDRAFAVYAVT-KFLMALG   266
hsMCT4          ------------PGSGPPRPSRR---------LLDLSVF-RDRGFVLYAVA-ASVMVLG   241
hsMCT7          QQ---VLVK----TSPRPSEKKAP---------LLDFSIL-KEKSFICYALF-GLFATLG   307
hsMCT11         -----------------PR-SPLA---------ALGLSLF-TRRAFSIFALG-TALVGGG   257
hsMCT13         ----------------GPR-AQ-------------LTSLL-HHGPFLRYTVA-LTLINTG   229
                                                           :              .

hsMCT8          YFVPYVHLMKYVEEEFSEIKETWV-LLVCIGATSGLGRLVSGHISDSIPGLKK-IYLQVL   392
hsMCT10         YFVPYVHLMKHVNERFQDEKNKEV-VLMCIGVTSGVGRLLFGRIADYVPGVKK-VYLQVL   364
hsMCT9          -FPPSLLMEDVARSSNVKEEEFIMPLISIIGIMTAVGKLLLGILADFKWINTLYLYVAT-   378
hsMCT14         FVIPFIHLPEIVNLYNLSEQNDVFPLTSIIAIVHIFGKVILGVIADLPCISVWNVFLLA-   388
hsMCT5          YFIPTFHLVARAKTLGIDIMDASY-LVSVAGILETVSQIISGWVADQNWIKKYHYHKSY-   372
hsMCT12         CSPLFVYLVPYALSVGVSHQQAAF-LMSILGVIDIIGNITFGWLTDRRCLKNYQYVCYLF   355
hsMCT6          FPLPQVFLVPYAMWHSVDEQQAAL-LISIIGFSNIFLRPLAGLMAGRPAFASHRKYLFSL   310
hsMCT1          LFAPLVFLSSYGKSQHYSSEKSAF-LLSILAFVDMVARPSMGLVANTKPIRPRIQYFFAA   335
hsMCT2          FFAPIIFLAPYAKDQGIDEYSAAF-LLSVMAFVDMFARPSVGLIANSKYIRPRIQYFFSF   319
hsMCT3          LFVPAILLVNYAKDAGVPDTDAAF-LLSIVGFVDIVARPACGALAGLARLRPHVPYLFSL   325
hsMCT4          LFVPPVFVVSYAKDLGVPDTKAAF-LLTILGFIDIFARPAAGFVAGLGKVRPYSVYLFSF   300
hsMCT7          FFAPSLYIIPLGISLGIDQDRAAF-LLSTMAIAEVFGRIGAGFVLNREPIRKIYIELIC-   365
hsMCT11         YFVPYVHLAPHALDRGLGGYGAAL-VVAVAAMGDAGARLVCGWLADQGWVPLPRLLAVF-   315
hsMCT13         YFIPYLHLVAHLQDLDWDPLPAAF-LLSVVAISDLVGRVVSGWLGDAVPGPVTRLLMLW-   287
                     . :                :     .       .   * : .
```

```
hsMCT8          SFLLLGLMSMM-----IPLCRDFGG--LIVVCLFLGLCDGFFITIMAP--IAFELVGPMQ    443
hsMCT10         SFFFIGLMSMM-----IPLCSIFGA--LIAVCLIMGLFDGCFISIMAP--IAFELVGAQD    415
hsMCT9          -LIIMGLALCA-----IPFAKSYVT--LALLSGILGFLTGNWS-IFPY--VTTKTVGIEK    427
hsMCT14         -NFTLVLSIFI-----LPLMHTYAG--LAVICALIGFSSGYFS-LMPV--VTEDLVGIEH    437
hsMCT5          -LILCGITNLL-----APLATTFPL--LMTYTICFAIFAGGYLALILP--VLVDLCRNST    422
hsMCT12         AVGMDGLCYLC-----LPMLQSLPL--LVPFSCTFGYFDGAYVTLIPV--VTTEIVGTTS    406
hsMCT6          ALLLNGLTNLV-----CAASGDFWV--LVGYCLAYSVSMSGIGALIFQ--VLMDIVPMDQ    361
hsMCT1          SVVANGVCHML-----APLSTTYVG--FCVYAGFFGFAFGWLSSVLFE--TLMDLVGPQR    386
hsMCT2          AIMFNGVCHLL-----CPLAQDYTS--LVLYAVFFGLGFGSVSSVLFE--TLMDLVGAPR    370
hsMCT3          ALLANGLTDLS-----SARARSYGA--LVAFCVAFGLSYGMVGALQFE--VLMAAVGAPR    376
hsMCT4          SMFFNGLADLA-----GSTAGDYGG--LVVFCIFFGISYGMVGALQFE--VLMAIVGTHK    351
hsMCT7          -VILLTVSLFAFT-----FATEFWG--LMSCSIFFGFMVGTIGGTHIPLLAEDDVVGIEK    417
hsMCT11         -GALTGLGLWVVGLVPVVGGEESWGGPLLAAAVAYGLSAGSYAPLVFG--VLPGLVGVGG    372
hsMCT13         -TTLTGVSLALFP---VAQ----APTALVALAVAYGFTSGALAPLAFS--VLPELIGTRR    337
                      :                    :       .   .

hsMCT8          ASQAIGYLLGMMALPMIAGPPIAGLLRNCFGDYHVAFYFAGVPPIIGAVILFFVPLMHQ-    502
hsMCT10         VSQAIGFLLGFMSIPMTVGPPIAGLLRDKLGSYDVAFYLAGVPPLIGGAVLCFIPWIHS-    474
hsMCT9          LAHAYGILMFFAGLGNSLGPPIVGWFYDWTQTYDIAFYFSGFCVLLGGFILLLAALPSW-    486
hsMCT14         LANAYGIIICANGISALLGPPFAGWIYDITQKYDFSFYICGLLYMIGILFLLIQPCIRII    497
hsMCT5          VNRFLGLASFFAGMAVLSGPPIAGWLYDYTQTYNGSFYFSGICYLLSSVSFFFVPLAE--    480
hsMCT12         LSSALGVVYFLHAVPYLVSPPIAGRLVDTTGSYTAAFLLCGFSMIFSSVLLGFARLIK--    464
hsMCT6          FPRALGLFTVLDGLAFLISPPLAGLLLDATNNFSYVFYMSSFFLISAALFMG-GSF--Y-    417
hsMCT1          FSSAVGLVTIVECCPVLLGPPLLGRLNDMYGDYKYTYWACGVVLIISGIYLFIGMGINY-    445
hsMCT2          FSSAVGLVTIVECGPVLLGPPLAGKLVDLTGEYKYMYMSCGAIVVAASVWLLIGNAINY-    429
hsMCT3          FPSALGLVLLVEAAAVLIGPPSAGRLVDALKNYEIIFYLAGSEVALAGVFMAVATNCCL-    435
hsMCT4          FSSAIGLVLLMEAVAVLVGPPSGGKLLDATHVYMYVFILAGAEVLTSSLILLLGNFFCI-    410
hsMCT7          MSSAAGVYIFIQSIAGLAGPPLAGLLVDQSKIYSRAFYSCAAGMALAAVCLALVRPCKM-    476
hsMCT11         VVQATGLVMMLMSLGGLLGPPLSGFLRDETGDFTASFLLSGSL-ILSGSFIYIGLPRAL-    430
hsMCT13         IYCGLGLLQMIESIGGLLGPPLSGYLRDVTGNYTASFVVAGAF-LLSGSGILLTLPHFF-    395
                     *            .**  * : :    :   :  ..     .   :

hsMCT8          RMFKKEQRDSS-----KDK----------------------------MLAPDPDPNGELL    529
hsMCT10         KKQREISKTTG-----KEK----------------------------MEKMLENQNS-LL    500
hsMCT9          DTCNKQLPKPAPT----------------TFLYKVASNV---------------------    509
hsMCT14         EQSRRKYMDGA--------------------------HV---------------------    510
hsMCT5          ----RW--KNSLT-----------------------------------------------    487
hsMCT12         ----RM-RKTQLQFIAKESDPKLQLWTNGSVAYSVARELDQ--------KHGEPVATAV-    510
hsMCT6          ALQKKEQGKQAVAADALERDLFLEAK----------DGPGKQRSPEIMCQSSRQPRPAGV    467
hsMCT1          RLLAKEQKANEQKKESKEEETSIDVAGKPNEVTKAAESPDQKDT------EGGPKEEES-    498
hsMCT2          RLLAKERKEENARQKTRESEPLSKSK-HSEDVNVKVSNA-----------QSVTSERET-    476
hsMCT3          RCAKAAPSGPGTEGGASDTEDAE-AEGDSEPLPVVAEEPGNLEALEVLSARGEPTEPEIE    494
hsMCT4          RKKPKE---PQPEVAAAEEEKLH-KPPAD--------SGVDLREVEHFLKAEPEKNGEVV    458
hsMCT7          GLCQHHHS-GETKVVSHRGKTLQDIP-------------------EDFLEMDLAKNEHRV    516
hsMCT11         PSCGPASP-PA-TPPPE-TGELLPAP-------------------QAVLLSPGGPGSTLD    468
hsMCT13         CFSTTTSG-PQ-DLVTEALDTKVPLP-------------------KEGLEED--------    426


hsMCT8          ----PGSPN----PEEPI--------------------         539
hsMCT10         ----SSSSGMFKKESDSII-------------------         515
hsMCT9          --------------------------------------         509
hsMCT14         --------------------------------------         510
hsMCT5          --------------------------------------         487
hsMCT12         -------PGYSLT-------------------------         516
hsMCT6          NKHLWGCPASSRTSHEWLLWPKAVLQAKQTALGWNSPT         505
hsMCT1          -------P---------V--------------------         500
hsMCT2          -------N---------I--------------------         478
hsMCT3          -----ARP---RLAAESV--------------------         504
hsMCT4          -----HTP---ETSV-----------------------         465
hsMCT7          --HVQMEP---------V--------------------         523
hsMCT11         --T-TC--------------------------------         471
hsMCT13         --------------------------------------         426
```

Supplementary Fig. 15. Multiple sequence alignment of the human MCT-family by Clustal omega. Highlighted are the residues at which an Ala substituent results in a functional reduction of >70% (red), 40-70% (magenta), 10-40% (yellow) compared to WT (T4 uptake in COS-1 cells). In red font are the

residues at which pathogenic variants have been identified, substituting the original residue in residue(s) other than an Ala. MCT8 represents the long isoform. Indicated variants in MCTs other than MCT8 are derived from an exhaustive literature search [15, 38, 39, 40, 41, 42, 43, 44].

```
SecStr   : CCCCCCCCCCCCCCCCCCCCCCCCCCCCCCCCCCCCCCCCCCCCCCCCCCCCCCCCCCCCCCCCCCCCCCCCCCCCCCCCCCCCCC
Target   : MALQSQASEEAKGPWQEADQEQQEPVGSPEPESEPEPEPEPEPVPVPPPEPQPEPQPLPDPAPLPELEFESERVHEPEPTPTVETR
Match    :
Template: ......................................................................................
SecStr   : ......................................................................................

SecStr   : CCCCCCCCCCCHHHHHHHHHHHHHHHHHHHHHHHHHHHHHHHHHHHHCCCHHHHHHHHHHHHHHHHHHHHHHHHHHHHHHHCCCHH
Target   : GTARGFQPPEGGFGWVVVFAATWCNGSIFGIHNSVGILYSMLLEEEKEKNRQVEFQAAWVGALAMGMIFFCSPIVSIFTDRLGCRI
Match    :           GG:GW:VV::A    G  |:: :S| |:|: |          :  |::W|::| |:::|  :PI SI:::| G RI
Template: ..........GGWGWAVVIGAFISIGFSYAFPKSITVFFKEIEGIFH.....TTSEVSWISSIMLAVMYGGGPISSILVNKYGSRI
SecStr   : ..........CCCHHHHHHHHHHHHHHHCCGGGGGGCHHHHHHHHHH.....HHHHHHHHHHHHHHHHHHHHHHHHHHHHHHCHHH

SecStr   : HHHHHHHHHHHHHHHHHHHHHHHHHHHHHHHHHHHHHHHHHHHHHHHHHHHHHHHHHHHHHHHHHHHHHHCCHHHHHHHHHCCCCC
Target   : TATAGAAVAFIGLHTSSFTSSLSLRYFTYGILFGCGCSFAFQPSLVILGHYFQRRLGLANGVVSAGSSIFSMSFPFLIRMLGDKIK
Match    : :  :G::::  GL ::SF ::::  Y   G|| G G :F ::P:L::|G YF |R  LANG:: AGS |F  ::  L :::    :
Template: VMIVGGCLSGCGLIAASFCNTVQQLYVCIGVIGGLGLAFNLNPALTMIGKYFYKRRPLANGLAMAGSPVFLCTLAPLNQVFFGIFG
SecStr   : HHHHHHHHHHHHHHHHHHHCCHHHHHHHHHHHHHHHHHHHHHHHHHHHHHCCCCCHHHHHHHHHHHHHHHHHHHHHHHHHHHHHHC

SecStr   : HHHHHHHHHHHHHHHHHHHHHCCCCCCCCCCCCCCCCCCHHHHHHHHHHHHHHHHHHHCCHHHHHHHHHHHHHHHHHHHHHHHHHH
Target   : LAQTFQVLSTFMFVLMLLSLTYRPLLPSSQDTPSKRGVRTLHQRFLAQLRKYFNMRVFRQRTYRIWAFGIAAAALGYFVPYVHLMK
Match    :    :F |L: :|:   : :   RP| P                             :F :R | ||  G :  :G F:P V  L :
Template: WRGSFLILGGLLLNCCVAGALMRPIGP.............................LFTHRGFLLYLSGNVIMFFGLFAPLVFLSS
SecStr   : HHHHHCCHHHHHHHHHHHHHHHHHHHHHHHHHHHH.....HHHHHHHHHHHHHHHHHHHHCCCCHHHHHHHHHHHHHHHHHHHHHH

SecStr   : HHHHHCCCHHHHHHHHHHHHHHHHCCCCCCCCCCCCCCCHHHHHHHHHHHHHHHHHHHHCCCHHHHHHHHHHHHHHHHHHHHHHHH
Target   : YVEEEFSEIKETWVLLVCIGATSGLGRLVSGHISDSIPGLKKIYLQVLSFLLLGLMSMMIPLCRDFGGLIVVCLFLGLCDGFFITI
Match    : Y ::|  : :::  LL  |: :: ::R   G |::     |  Y: : S :  G:  M| PL   | G: V : F:G:: G:: :|
Template: YGKSQHYSSEKSAFLLSILAFVDMVARPSMGLVAN.....RIQYFFAASVVANGVCHMLAPLSTTYVGFCVYAGFFGFAFGWLSSV
SecStr   : HHHHHCCHHHHHHHHHHHHHHHHHHHHHHHHHHHH.....HHHHHHHHHHHHHHHHHHHHCCCCHHHHHHHHHHHHHHHHHHHHHH

SecStr   : HHHHHHCCCCCHHHHHHHHHHHHHHHHHHHHHHHHHHHHHHHHHCCCCHHHHHHHHHHHHHHHHHHHHHHHHHHHHHHHHHCCCCC
Target   : MAPIAFELVGPMQASQAIGYLLGMMALPMIAGPPIAGLLRNCFGDYHVAFYFAGVPPIIGAVILFFVPLMHQRMFKKEQRDSSKDK
Match    : |    :|LVGP:: S:A|G :  : : P:| GPP| G L:: |GDY  :|| :GV  II::| LF:   :: R|:
Template: LFETLMDLVGPQRFSSAVGLVTIVECCPVLLGPPLLGRLNDMYGDYKYTYWACGVVLIISGIYLFIGMGINYRLL...........
SecStr   : HHHHHHHHCCCHHHHHHHHHHHHHHHHCCCHHHHHHHHHHHHCCHHHHHHHHHHHHHHHHHHHHHHHHHHHHHHH...........

SecStr   : CCCCCCCCCCCCCCCCCCCCCCC [Secondary structure predicted by PsiPred]
Target   : MLAPDPDPNGELLPGSPNPEEPI
Match    :
Template: .......................
SecStr   : .......................
```

Supplementary Fig. 16. Amino acid sequence alignment between human MCT8 (target) and the prime template MCT1 (template) used for the MCT8 homology model in outward-open conformation. Highlighted (in magenta: MFS1 (PDB#6HCL); blue: FucP (PDB #3O7Q); red: MCT2 (PDB# 7BP3)) are the elements derived from templates other than MCT1.

```
SecStr   : CCCCCCCCCCCCCCCCCCCCCCCCCCCCCCCCCCCCCCCCCCCCCCCCCCCCCCCCCCCCCCCCCCCCCCCCCCCCCCCCCCCCCC
Target   : MALQSQASEEAKGPWQEADQEQQEPVGSPEPESEPEPEPEPEPVPVPPPEPQPEPQPLPDPAPLPELEFESERVHEPEPTPTVETR
Template: ......................................................................................
SecStr   : ......................................................................................

SecStr   : CCCCCCCCCCCCHHHHHHHHHHHHHHHHHHHHHHHHHHHHHHHHHHCCCHHHHHHHHHHHHHHHHHHHHHHHHHHHHHHHHCCCHH
Target   : GTARGFQPPEGGFGWVVVFAATWCNGSIFGIHNSVGILYSMLLEEEKEKNRQVEFQAAWVGALAMGMIFFCSPIVSIFTDRLGCRI
Match    :           GG:GW:VV::A    G  |:: :S| |:|: |          :  |::W|::| |:::|  :PI SI:::| G RI
Template: ..........GGWGWAVVIGAFISIGFSYAFPKSITVFFKEIEGIFH.....TTSEVSWISSIMLAVMYGGGPISSILVNKYGSRI
SecStr   : ..........CCHHHHHHHHHHHHHHHHHTTTTCCCCHHHHHHHHHH.....HHHHHHHHHHHHHHHHHHHHHHHHHHHHHHCHHH

SecStr   : HHHHHHHHHHHHHHHHHHHHHHHHHHHHHHHHHHHHHHHHHHHHHHHHHHHHHHHHHHHHHHHHHHHHHHHCCHHHHHHHHCCCCC
Target   : TATAGAAVAFIGLHTSSFTSSLSLRYFTYGILFGCGCSFAFQPSLVILGHYFQRRLGLANGVVSAGSSIFSMSFPFLIRMLGDKIK
Match    : :  :G:::   GL ::SF ::::  Y   G|| G G :F ::P:L::|G YF |R  LANG:: AGS |F  ::  L :::   :
Template: VMIVGGCLSGCGLIAASFCNTVQQLYVCIGVIGGLGLAFNLNPALTMIGKYFYKRRPLANGLAMAGSPVFLCTLAPLNQVFFGIFG
SecStr   : HHHHHHHHHHHHHHHHHHCCCCHHHHHHHHHHHHHHHHHHHHHHHHHHHHCCCHHHHHHHHHHHCHHHHHHHHHHHHHHHHHHHHC

SecStr   : HHHHHHHHHHHHHHHHHCCCCCCCCCCCCCCCCHHHHHHHHHHHHHHHHHHHHHHHHHCCHHHHHHHHHHHHHHHHHHHHHHHHHH
Target   : LAQTFQVLSTFMFVLMLLSLTYRPLLPSSQDTPSKRGVRTLHQRFLAQLRKYFNMRVFRQRTYRIWAFGIAAAALGYFVPYVHLMK
Match    :    :F |L: :|:   : :   RP| P                                :R | ||  G :   :G F:P V L :
Template: WRGSFLILGGLLLNCCVAGALMRPIGP................................HRGFLLYLSGNVIMFFGLFAPLVFLSS
SecStr   : HHHHHHHHHHHHHHHHHHHCCCCCCCC................................CCHHHHHHHHHHHHHCCCCTTTTCHHH

SecStr   : HHHHHCCCHHHHHHHHHHHHCCCCCCCCCCCCCCCCCHHHHHHHHHHHHHHHHHHHHHCCCHHHHHHHHHHHHHHHHHHHHHHHHH
Target   : YVEEEFSEIKETWVLLVCIGATSGLGRLVSGHISDSIPGLKKIYLQVLSFLLLGLMSMMIPLCRDFGGLIVVCLFLGLCDGFFITI
Match    : Y ::|  : :::  LL  |: :: ::R   G |:         Y: : S :  G:  M| PL   | G: V : F:G:: G:: :|
Template: YGKSQHYSSEKSAFLLSILAFVDMVARPSMGLVA........QYFFAASVVANGVCHMLAPLSTTYVGFCVYAGFFGFAFGWLSSV
SecStr   : HHHCCCCCHHHHHHHHHHHHHHHHHHHHHHHHHH........CCCHHHHHHHHHHHHHHCCCCCCHHHHHHHHHHHHHHHHHHHHH

SecStr   : HHHHHHCCCCCHHHHHHHHHHHHHHHHHHHHHHHHHHHHHHCCCCHHHHHHHHHHHHHHHHHHHHHHHHHHHHHHHHHHHHCCCCC
Target   : MAPIAFELVGPMQASQAIGYLLGMMALPMIAGPPIAGLLRNCFGDYHVAFYFAGVPPIIGAVILFFVPLMHQRMFKKEQRDSSKDK
Match    : |    :|LVGP:: S:A|G :  : : P:| GPP| G L:: |GDY  :|| :GV  II::| LF:   :: R|:
Template: LFETLMDLVGPQRFSSAVGLVTIVECCPVLLGPPLLGRLNDMYGDYKYTYWACGVVLIISGIYLFIGMGINYRLLA..........
SecStr   : HHHHHHHHHCCCHHHHHHHHHHHCHHHHHHHTTTTHHHHHHHCCCTTTTCCCHHHHHHHHHHHHHHHHHHHHHHHH..........

SecStr   : CCCCCCCCCCCCCCCCCCCCCCC
Target   : MLAPDPDPNGELLPGSPNPEEPI
Template:........................
SecStr   : .......................
```

Supplementary Fig. 17. Amino acid sequence alignment between human MCT8 (target) and the prime template MCT1 (template) used for the MCT8 homology model in inward-open conformation. Highlighted (in magenta: MFS1 (PDB#6HCL); blue: FucP (PDB #3O7Q); red: MCT2 (PDB# 7BP3); yellow: MCT1 (PDB# 7DA5)) are the elements derived from templates other than MCT1 (PDB# 7CKO).

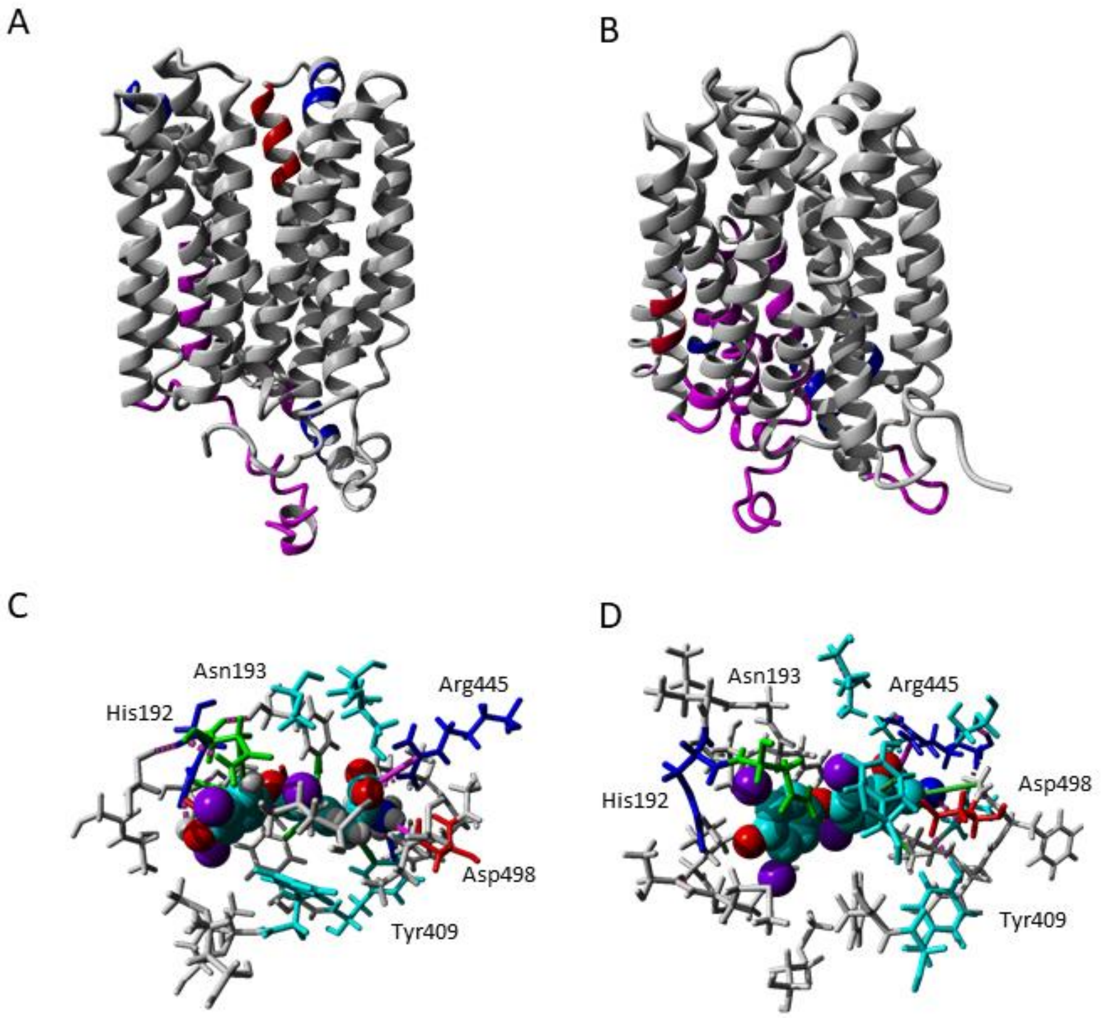

Supplementary Fig. 18. Homology model of MCT8 in outward-open (A) and inward-open (B) configuration. Both models were predominantly based on the EM-structures of MCT1 (PDB# 7CKO and 7DA5, grey residues), and further improved into a hybrid model with elements of MFS (6HCL, magenta residues), FucP (3O7Q, blue residues), and MCT2 (7BP3, red residues). Residues composing the substrate binding pocket in outward-open (C) and inward-open (D) are shown in sticks (view: outside → inside). A T4 molecule docked at the bottom of the substrate binding center is shown as a ball-structure. Blue: basic residues, red: acidic residues, turquoise: hydroxylic residues, green: amines, grey: hydrophobic residues.

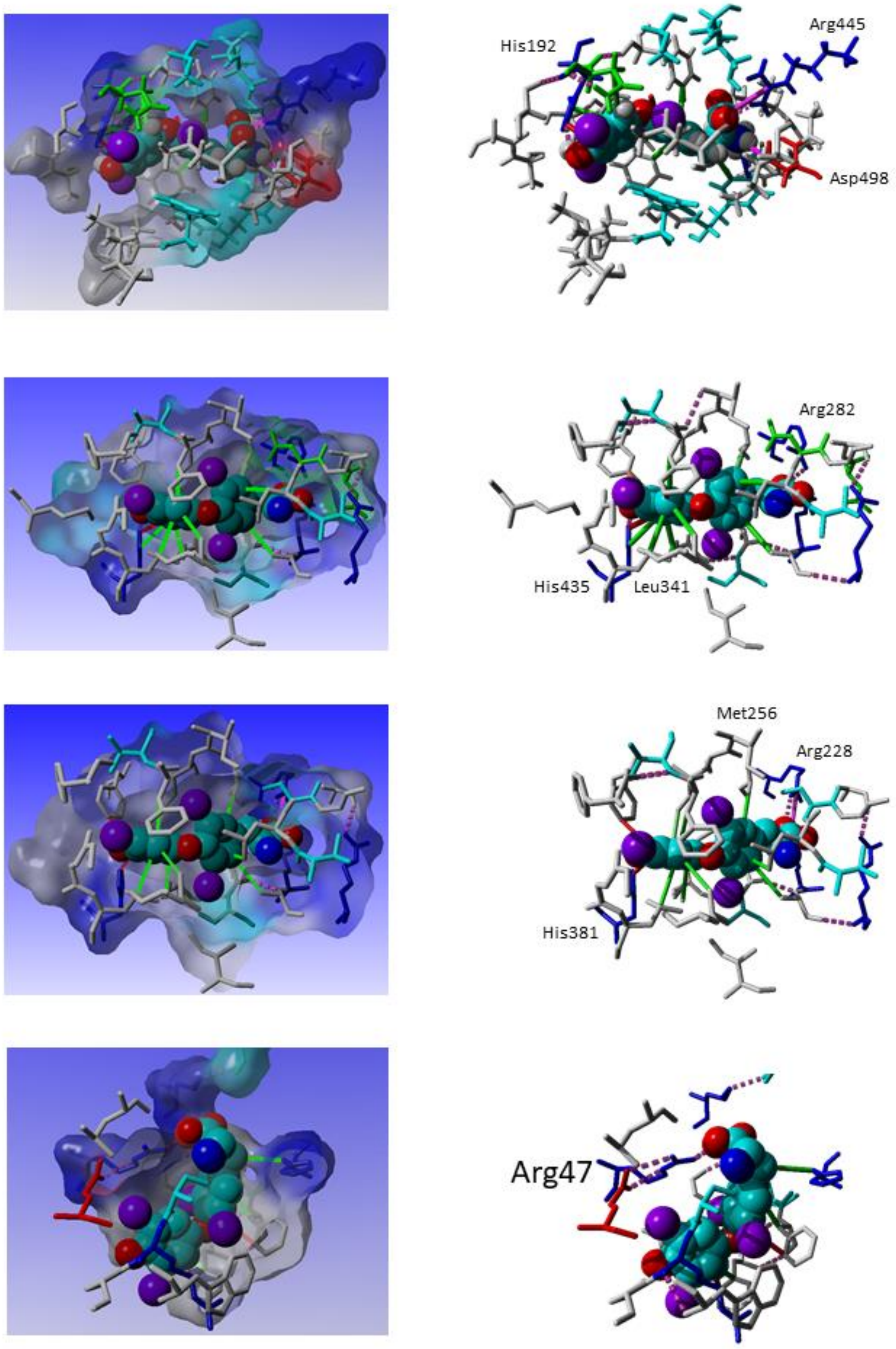

Supplementary Fig. 19. Comparison of substrate binding pockets in major thyroid hormone interacting proteins. Residues composing the substrate binding center in the homology model of MCT8 in outward-open conformation (see Supplementary Fig. 18C), and the crystal structures of TRβ

(PDB#3gws), TRα (PDB#2h77), or the thyroid hormone-interacting protein mu-crystallin (PDB#4bva). Indicated are the Arg and His residues as well as some of the hydrophobic residues known to exert a critical function for the involved protein. Blue: basic residues, red: acidic residues, turquoise: hydroxylic residues, green: amines, grey: hydrophobic residues.

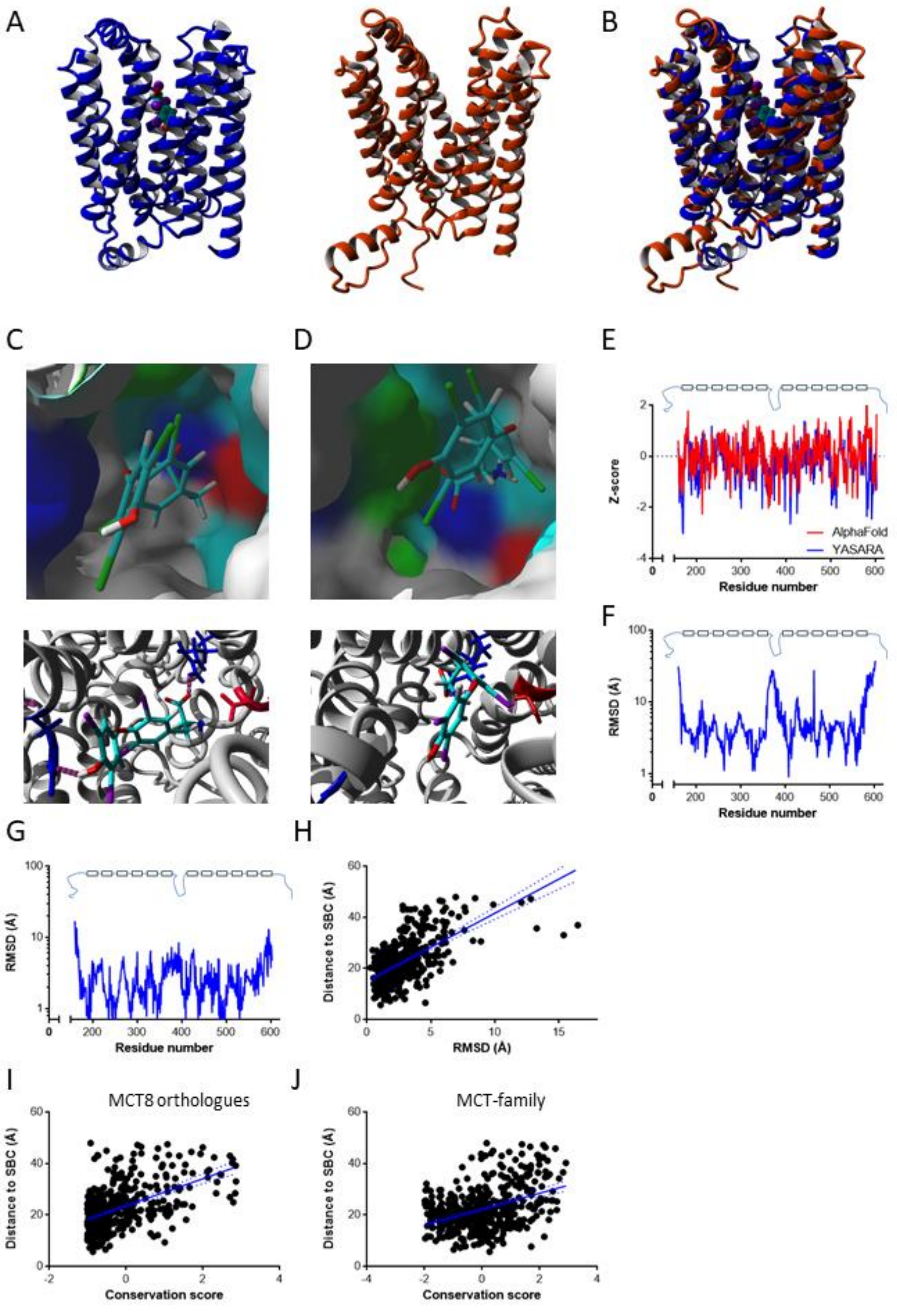

Supplementary Fig. 20. Comparison of MCT8 homology model to AlphaFold-2 derived structural prediction. (A) MCT8 homology model using YASARA Structure (left) or AlphaFold2 (right) and their overlay (B). Zoom-in of the substrate binding center of the YASARA-based model (C) and AlphaFold2

model (D) in which a T4 molecule has been docked. Note the different orientation of the His192 residue, which is, in line with available experimental data [4], fully exposed via the substrate channel in (C), but not in (D). (E) Comparison of the model reliability at residue level based on Z-scores [45]. (F) RMSD per residue comparing the YASARA and AlphaFold2 model, with highest RMSD in the intracellular N- and C-terminus as well as the intracellular and extracellular loops. (G) RMSD per residue after 2 ns molecular dynamic simulation of the YASARA model, showing a similar pattern as in (F). Correlation analysis between the RMSD shown in (G) (H), conservation score based on all MCT8 orthologues (I) or all human MCT-family members (J), and the distance of each residue to the substrate binding center. The lines in (H), (I) and (J) represent the mean with the 95% confidence intervals. Source data are provided as a Source Data file.

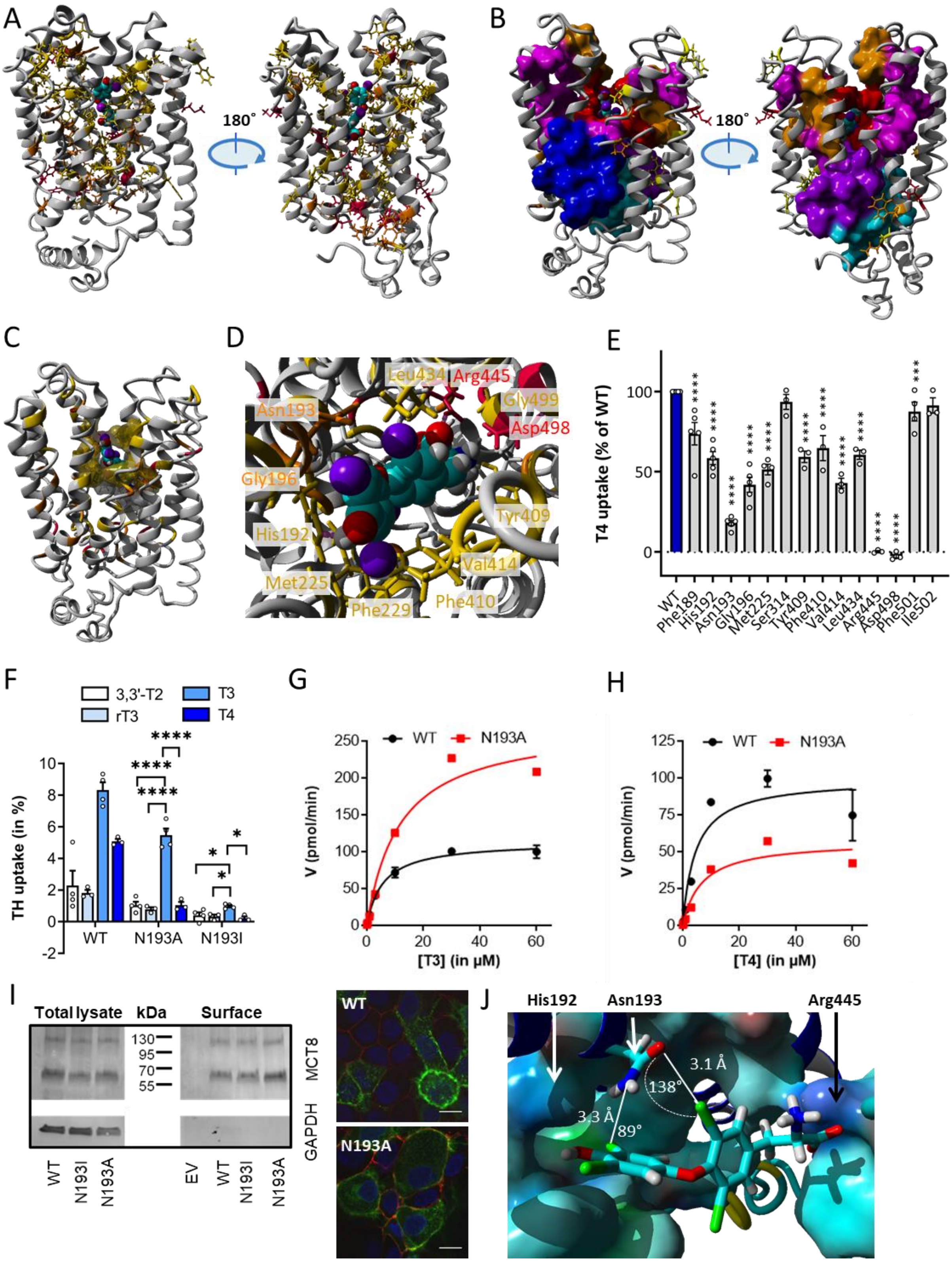

Supplementary Fig. 21. Mapping of Ala-screen onto MCT8 homology model – identification of 7 critical regions, including substrate binding pocket. (A) Color-coded mapping of functional impact on T4 transport by MCT8 Ala variants in JEG-3 cells (red: >90-100%, orange: 60-90%, yellow: 30-60%, grey: 0-

30% impact with WT MCT8 set as 0% impact) onto the MCT8 homology structure: frontal view (left panel) and rear view (right panel). (B) Critical functional domains in MCT8: 1) residues at substrate binding center (red), 2) channel-facing residues out substrate binding center (orange), 3) residues supporting substrate-interacting residues in group 1) and 2) (magenta), 4) cluster 1 composed of TMD5 and TMD 8 (purple), 5) cluster 2 composed of TMD2 and TMD11 (dark blue), and 6) a linker region connecting clusters 1 and 2 (light blue), as well as 7) residual residues (side-chains indicated as sticks). (C) Group 1, critical residues identified at the substrate binding center, with a close-up in (D), using the same color-coding as in (A). (E) T4 transport of WT and indicated mutant MCT8 in transiently transfected JEG-3 cells, expressed relative to WT (set as 100%, mean ±s.e.m). Dashed lines represent 0% uptake. P values were calculated using One-way ANOVA with Dunnet post-test, *** $p < 0.0005$, **** $p < 0.0001$. (F) Similarly, the transport of indicated iodothyronine by WT or N193A mutant MCT8 in JEG-3 cells. Thyroid hormone uptake (mean ±s.e.m) is expressed as a percentage of radio-labelled substrate added to the cells at the start of the incubation. P values were calculated using Two-way ANOVA with Bonferroni post-test, ** $p < 0.01$, *** $p < 0.005$. T3 (G) and T4 (H) transport kinetics (mean ±s.e.m) in COS-1 cells in absence of CRYM. Vmax (in pmol/min) and Km (in µM) values were calculated using Michaelis-Menten equation. (I) surface biotinylation in COS-1 and immunocytochemistry in JEG-3 cells, using MCT8-antibody (green), ZO-1-antibody as a membrane marker (red) and DAPI as a nuclear marker (blue). Scale bar corresponds to 15 uM. (J) Close-up of the substrate binding center highlighting the N193 in relation to a T4 substrate molecule. The C5-iodine of T4 and the side-chain oxygen of N193 are in close structural proximity (~3.1 Å) with overlapping Van der Waals radii and an σ-hole angle of 130-150°, which is optimal for the formation of a halogen bond between an iodobenzene and the side-chain oxygen of Asn [23]. Simultaneously, the σ-hole of the C5'-iodine is perpendicular to the side-chain nitrogen of N193 at a distance of 3.3Å, allowing the formation of a second halogen-bond that directing the large outer ring of T4. Exact P values are provided in Supplementary Data 1. Source data are provided as a Source Data file.

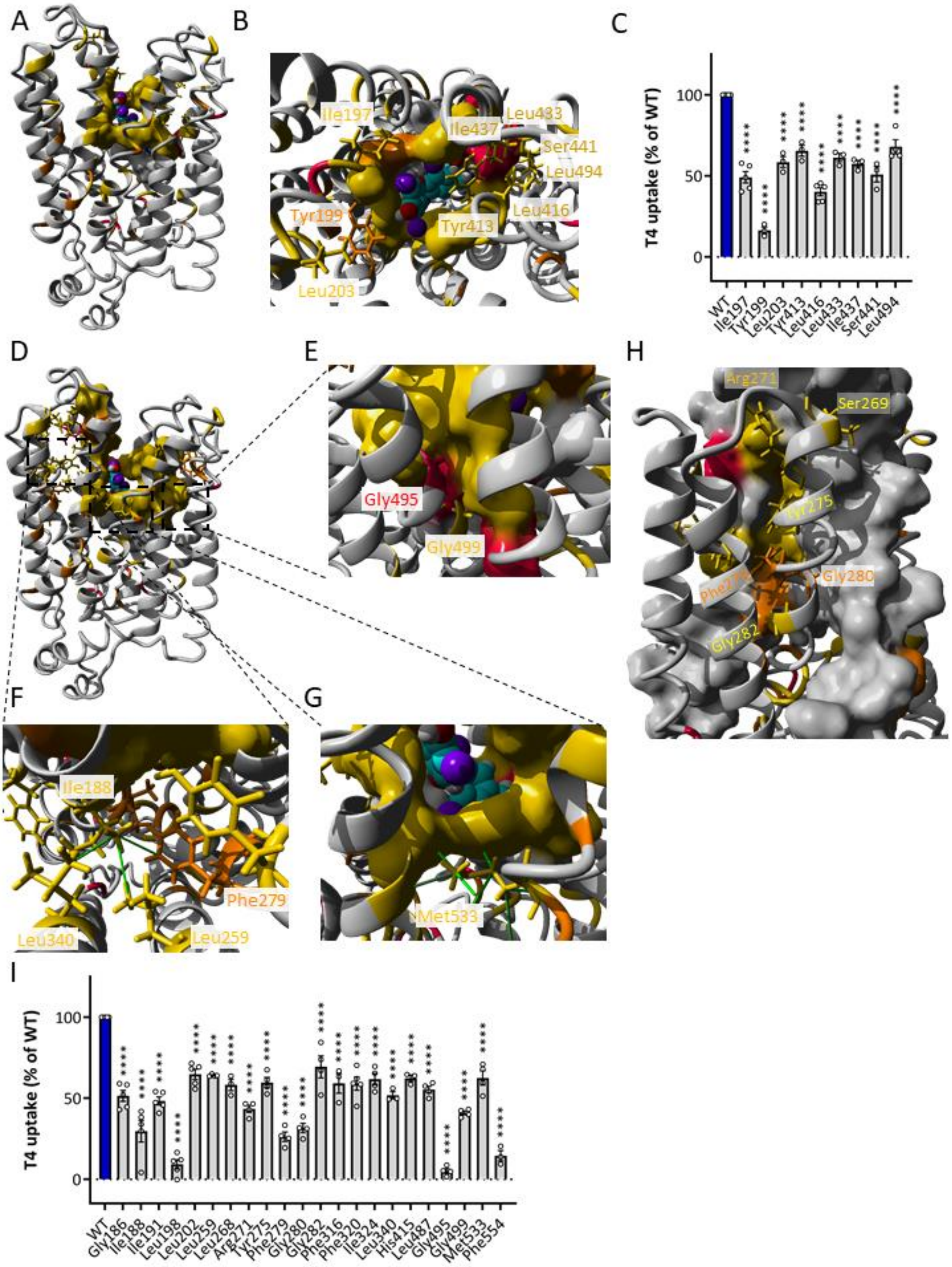

Supplementary Fig. 22. Mapping of Ala-screen onto MCT8 homology model – identification of 7 critical regions, including channel-flanking residues (group 2), and supporting residues (group 3). (A) Group 2, critical residues identified along the substrate channel outside the substrate binding center, with a

close-up in (B), color-coded as in Supplementary Fig. 18A. (C) T4 transport of WT and indicated mutant MCT8 in transiently transfected JEG-3 cells, expressed relative to WT MCT8 (set as 100%, mean ±s.e.m). P values were calculated using One-way ANOVA with Dunnet post-test, **** $p < 0.0001$. (D) Group 3: residues supporting substrate-interacting residues in group 1 and 2, by 1) potentially determining the helical structure of the TMDs composing the substrate binding center (e.g. G186, TMD1; G495, TMD10) or 2) their relative position to other TMDs (e.g. I188 and I191, L198 TMD1) (F), or 3) residues located in other TMDs, providing structural support to the TMDs that form the substrate binding center (G), with a particular hotspot in TMD4 (H).(I) T4 transport of WT and indicated mutant MCT8 in transiently transfected JEG-3 cells, expressed relative to WT (set as 100%, mean ±s.e.m). P values were calculated using One-way ANOVA with Dunnet post-test, **** $p < 0.0001$. Dashed lines represent 0% uptake. Source data are provided as a Source Data file.

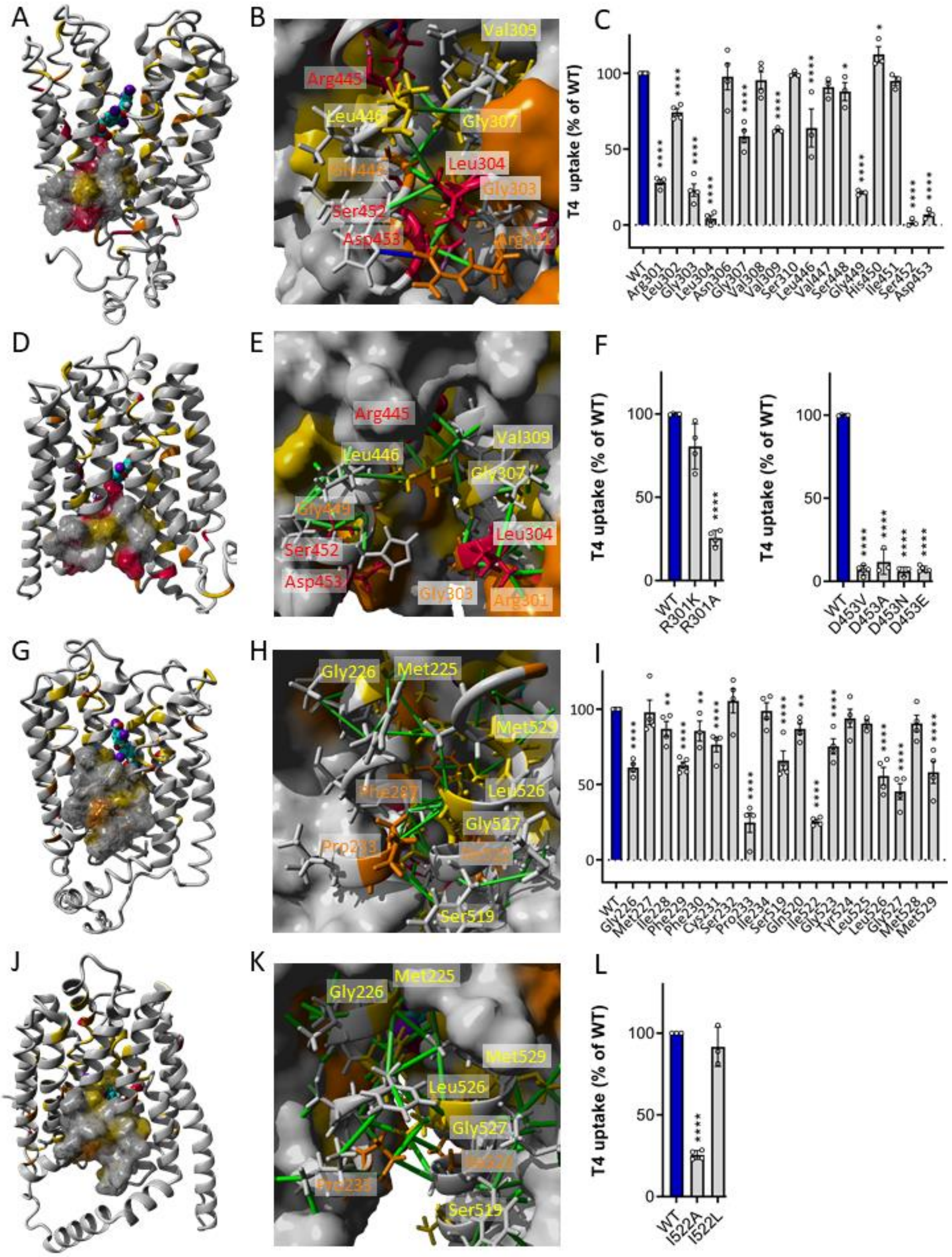

Supplementary Fig. 23. Mapping of Ala-screen onto MCT8 homology model – identification of 7 critical regions, including both pairs of rocker-helices (group 4 and 5). (A) Critical residues in group 4: cluster 1 composed of TMD5 and TMD8, connecting the N-terminal and C-terminal half of the MCT8 protein,

depicted in the MCT8 homology model in outward-open conformation, with a close-up in (B). (C) T4 transport of WT and indicated mutant MCT8 in transiently transfected JEG-3 cells, expressed relative to WT (set as 100%, mean ±s.e.m). P values were calculated using One-way ANOVA with Dunnet post-test, * $p < 0.05$, **** $p < 0.0001$. (D) The same cluster 1 highlighted in the MCT8 homology model in inward-open conformation, with a close-up in (E). Note the loss of interactions between both TMDs in the inward-open conformation, particularly the loss of the interaction between Asp453 and Arg301. (F) The effect of different substitutions of R301 and D453 on T4 transport (WT MCT8 set as 100%, mean ±s.e.m), illustrating the importance of local charge and side-chain length. P values were calculated using One-way ANOVA with Dunnet post-test, **** $p < 0.0001$. (G) Critical residues in group 5: cluster 2 composed of TMD2 and TMD11, connecting the N-terminal and C-terminal half of the MCT8 protein, depicted in the MCT8 homology model in outward-open conformation, with a close-up in (H). (I) T4 transport of WT and indicated mutant MCT8 in transiently transfected JEG-3 cells, expressed relative to WT (set as 100%, mean ±s.e.m). P values were calculated using One-way ANOVA with Dunnet post-test, ** $p < 0.005$, **** $p < 0.0001$. (J) The same cluster 1 highlighted in the MCT8 homology model in inward-open conformation, with a close-up in (K). Note the loss of interactions between both TMDs in the inward-open conformation. (L) The effect of different substitutions of I522 on T4 transport (WET MCT8 set as 100%, mean ±s.e.m), illustrating the importance of the branched side-chain. P values were calculated using One-way ANOVA with Dunnet post-test, **** $p < 0.0001$. In (B), (E), (H), (K) hydrophobic interactions are depicted as green sticks, electrostatic interactions as purple sticks and cation-pi interactions as blue sticks. Dashed lines represent 0% uptake. Source data are provided as a Source Data file.

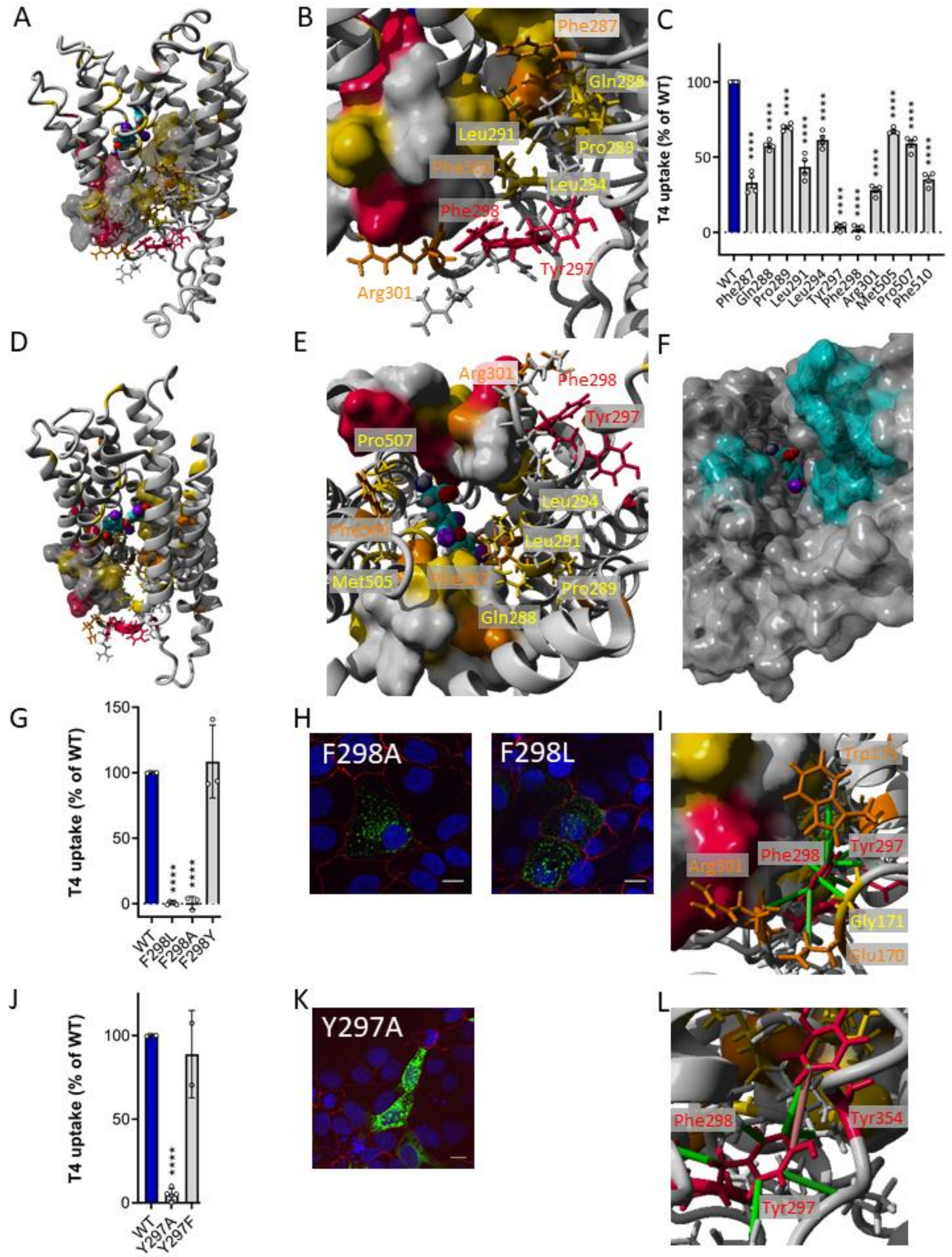

Supplementary Fig. 24. Mapping of Ala-screen onto MCT8 homology model – identification of 7 critical regions, including a linker region (group 6). (A) Critical residues in group 6: a linker region composed by the second part of TMD4 and ICL2, connecting cluster 1 and cluster 2, depicted in the MCT8

homology model in outward-open conformation, with a close-up in (B), using the same color-coding as in Supplementary Fig. 18A. (C) T4 transport of WT and indicated mutant MCT8 in transiently transfected JEG-3 cells, expressed relative to WT (set as 100%, mean ±s.e.m). P values were calculated using One-way ANOVA with Dunnet post-test, **** $p < 0.0001$. (D) The same linker region highlighted in the MCT8 homology model in inward-open conformation, with a close-up from an intracellular view in (E). (F) Substrate channel from an intracellular view with the linker region highlighted in light blue. (G) T4 transport of WT MCT8 and indicated Phe298 variants in transiently transfected JEG-3 cells, expressed relative to WT (set as 100%, mean ±s.e.m). P values were calculated using One-way ANOVA with Dunnet post-test, **** $p < 0.0001$. (H) Immunocytochemistry of JEG-3 cells transiently expressing indicated variants: DAPI (blue), MCT8 (green), the membrane marker ZO-1 (red). Scale bar indicates 15 µm. (I) MCT8 homology model indicating the position of F298 and its interactions with surrounding residues (green sticks represent hydrophobic interactions). (J) T4 transport of WT MCT8 and indicated Y297 variants in transiently transfected JEG-3 cells, expressed relative to WT (set as 100%, mean ±s.e.m). P values were calculated using One-way ANOVA with Dunnet post-test, **** $p < 0.0001$. (K) Immunocytochemistry of JEG-3 cells transiently expressing Y297A: DAPI (blue), MCT8 (green), the membrane marker ZO-1 (red). Scale bare indicates 15 µm. (L) MCT8 homology model indicating the position of Tyr297 and its interactions with surrounding residues (green sticks represent hydrophobic interactions, pink stick represents pi-pi interactions). Dashed lines represent 0% uptake. Source data are provided as a Source Data file.

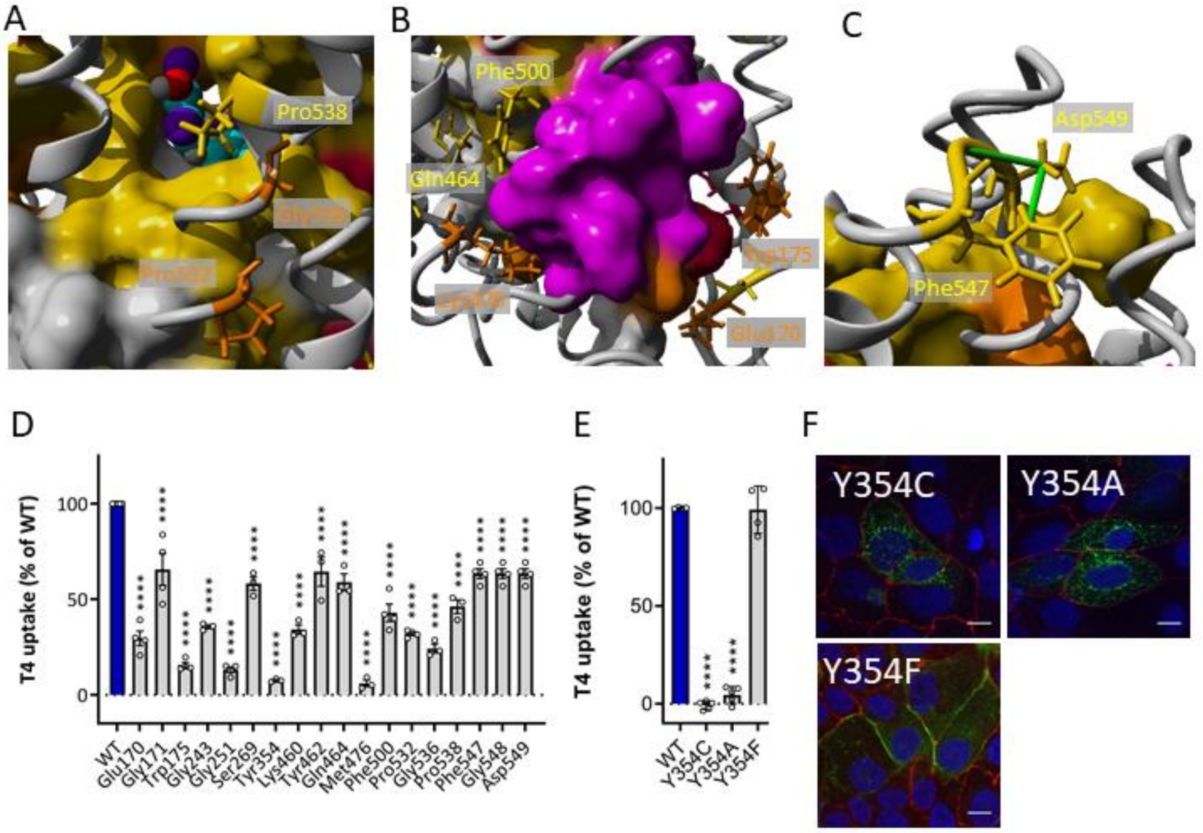

Supplementary Fig. 25. Mapping of Ala-screen onto MCT8 homology model – identification of 7 critical regions, including a residual group (group 7). Critical residues in group 7 could not be classified within groups 1-6, but were either structurally important Gly or Pro residues (A), residues that provided structural support to groups 4-6 (B), or putatively involved in stabilization of loop structures (C). Implied residues are shown as sticks with the same color-coding as in Supplementary Fig. 18A. Group 2 residues are shown in yellow (molecular surface) and group 4 residues in magenta. (D) T4 transport of WT and indicated mutant MCT8 in transiently transfected JEG-3 cells, expressed relative to WT (set as 100%, mean ±s.e.m). P values were calculated using One-way ANOVA with Dunnet post-test, **** $p < 0.0001$. (E) T4 transport of WT MCT8 and indicated Tyr354 variants in transiently transfected JEG-3 cells, expressed relative to WT (set as 100%, mean ±s.e.m). P values were calculated using One-way ANOVA with Dunnet post-test, **** $p < 0.0001$. (F) Immunocytochemistry of JEG-3 cells transiently expressing Y354 variants: DAPI (blue), MCT8 (green), the membrane marker ZO-1 (red). Scale bar indicates 15 µm. Dashed lines represent 0% uptake. Source data are provided as a Source Data file.

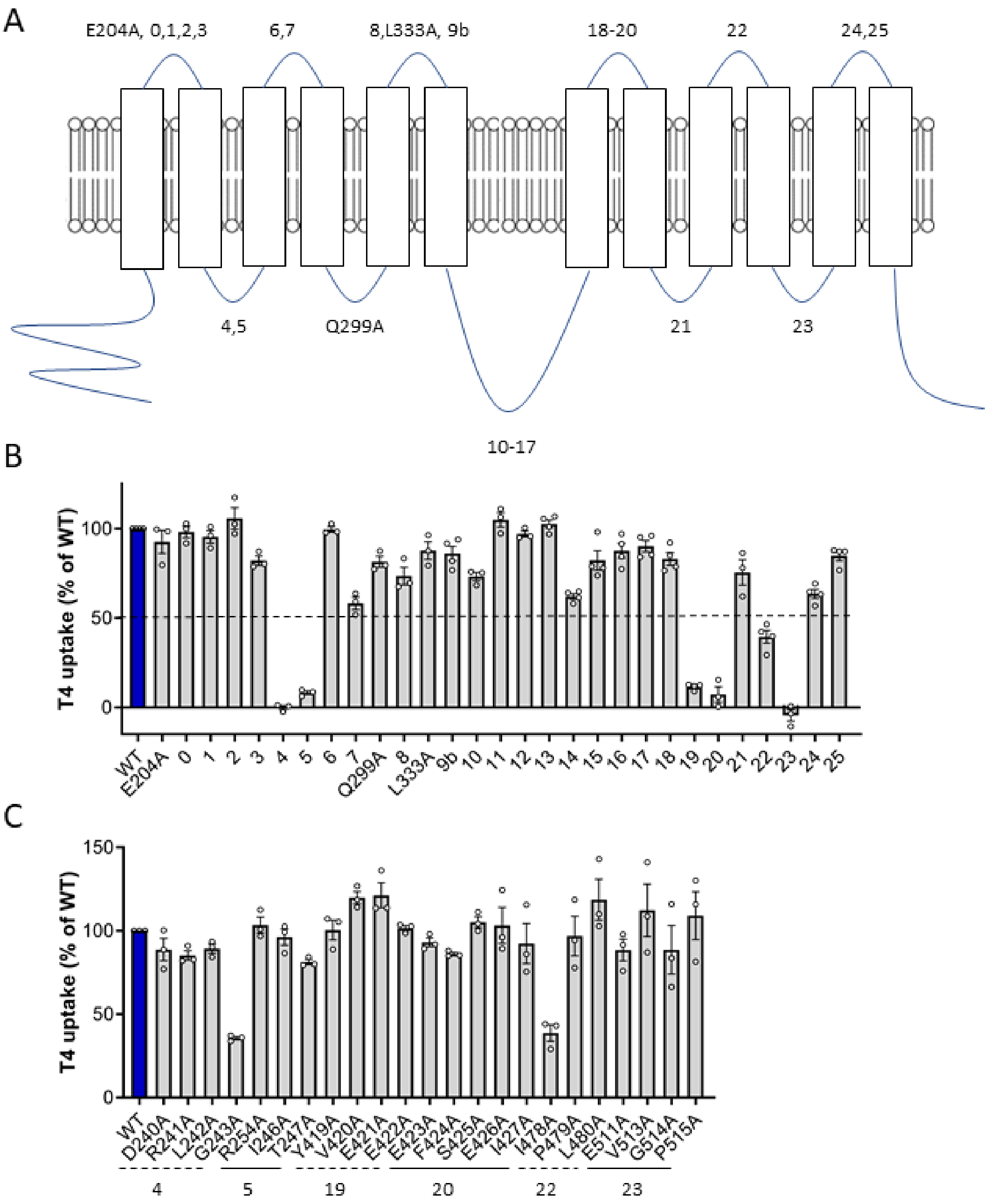

Supplementary Fig. 26. Screening of extracellular and intracellular loops. Visualization of functionally evaluated Ala blocks (A), residual uptake capacity of Ala blocks, expressed relatively to WT (100%) (B), and residual uptake capacity of individual Ala variants in Ala blocks with <50% residual uptake capacity (as determined in B), expressed relatively to WT (set as 100%, mean ±s.e.m) (C). Source data are provided as a Source Data file.

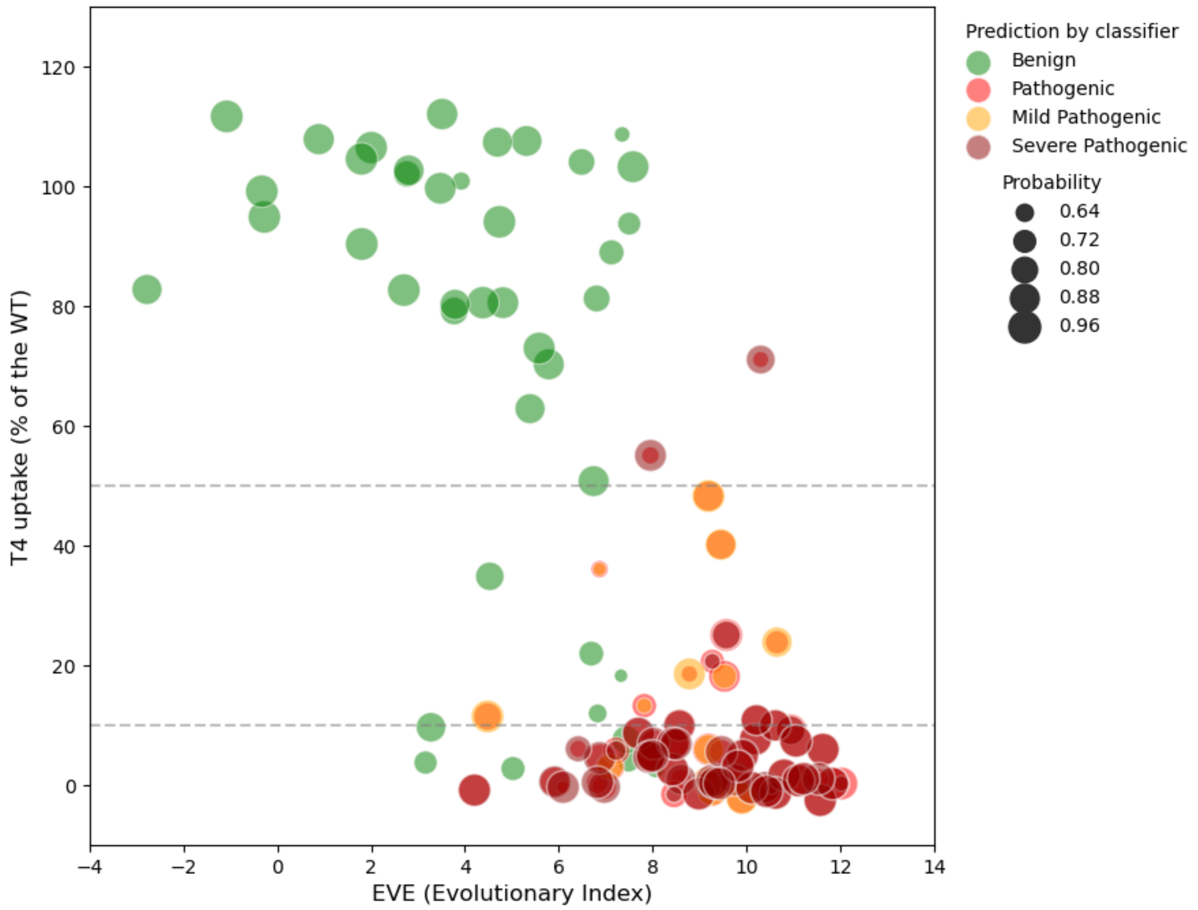

Supplementary Fig. 27. Pathogenicity and severity prediction of all functionally evaluated variants identified in patients with MCT8 deficiency as well as known benign variants by the unsupervised approached based on EVE; higher number denotes a stronger evolutionary constraint. Color and size of dots correspond to prediction by the dual pathogenicity-severity classifier. Dashed lines represent cut-off values for different LoF classes. Source data are provided as a Source Data file.

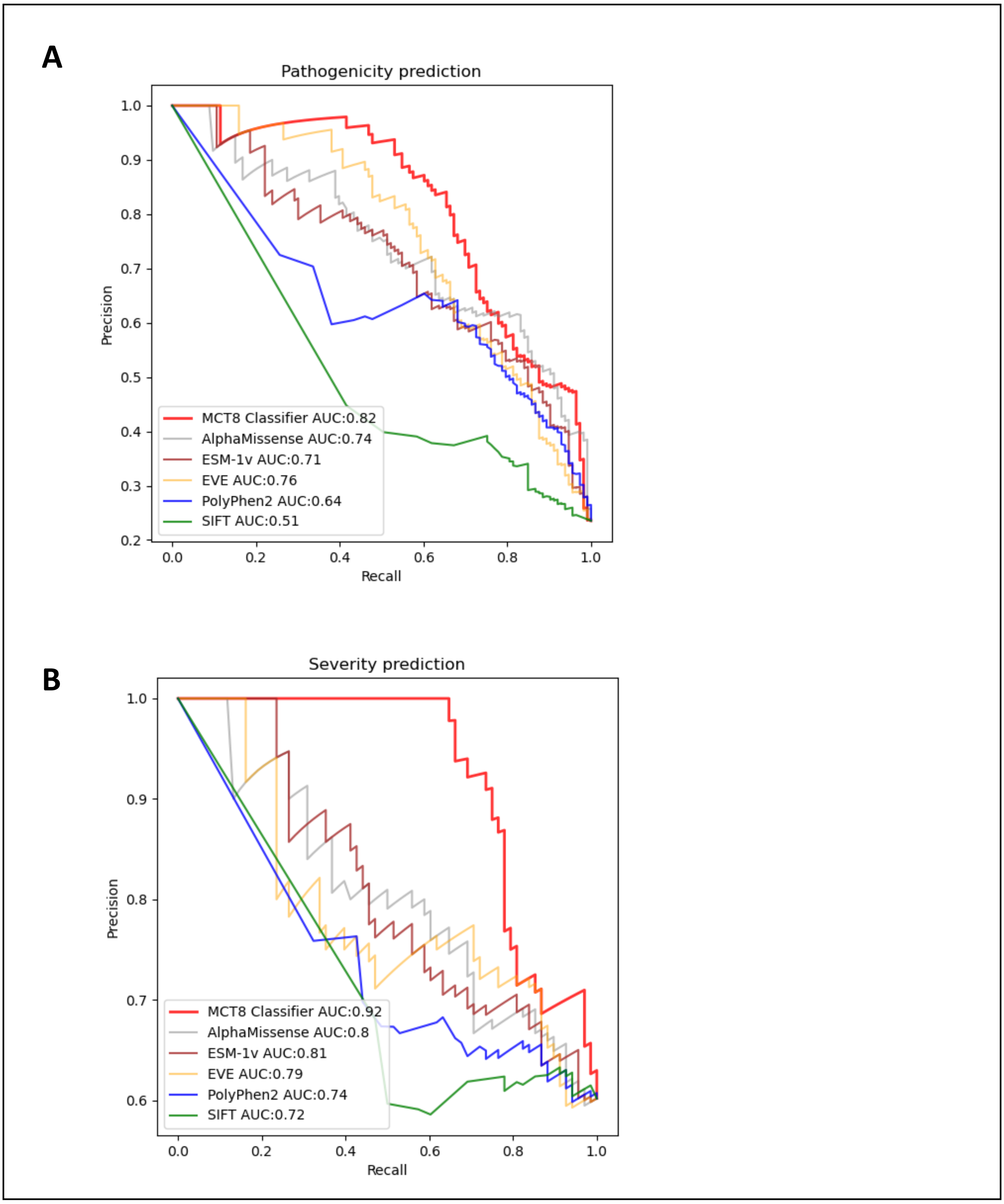

Supplementary Fig. 28. Performance of the MCT8 pathogenicity (A) and severity (B) classifier for all functionally evaluated variants as shown by AUPRC curve, with direct comparison to the unsupervised machine learning tool (EVE) and commonly used pathogenicity prediction tools. Source data are provided as a Source Data file.

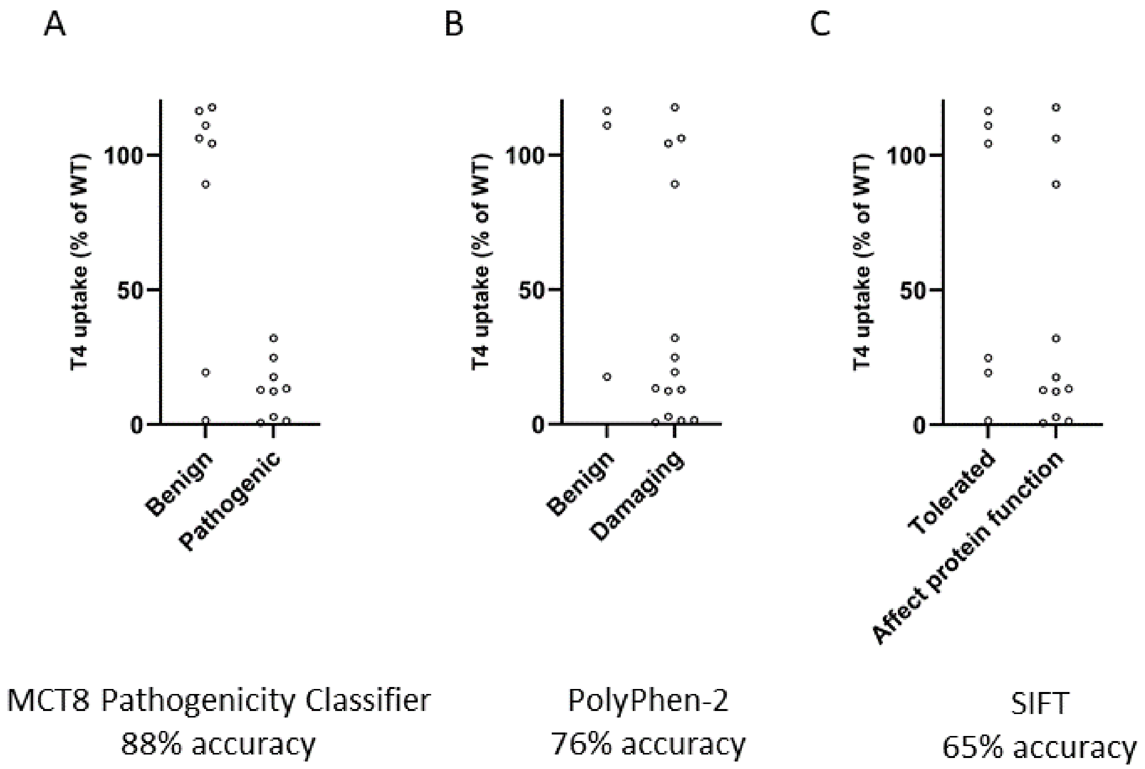

Supplementary Fig. 29. Classifier validation experiments compared to currently available prediction tools. Residual uptake capacity of newly identified patient variants and random artificial variants, expressed relatively to WT (100%), categorized by prediction of their pathogenicity by the MCT8 Pathogenicity Classifier (A), PolyPhen-2 (B) and SIFT (C). Source data are provided as a Source Data file.

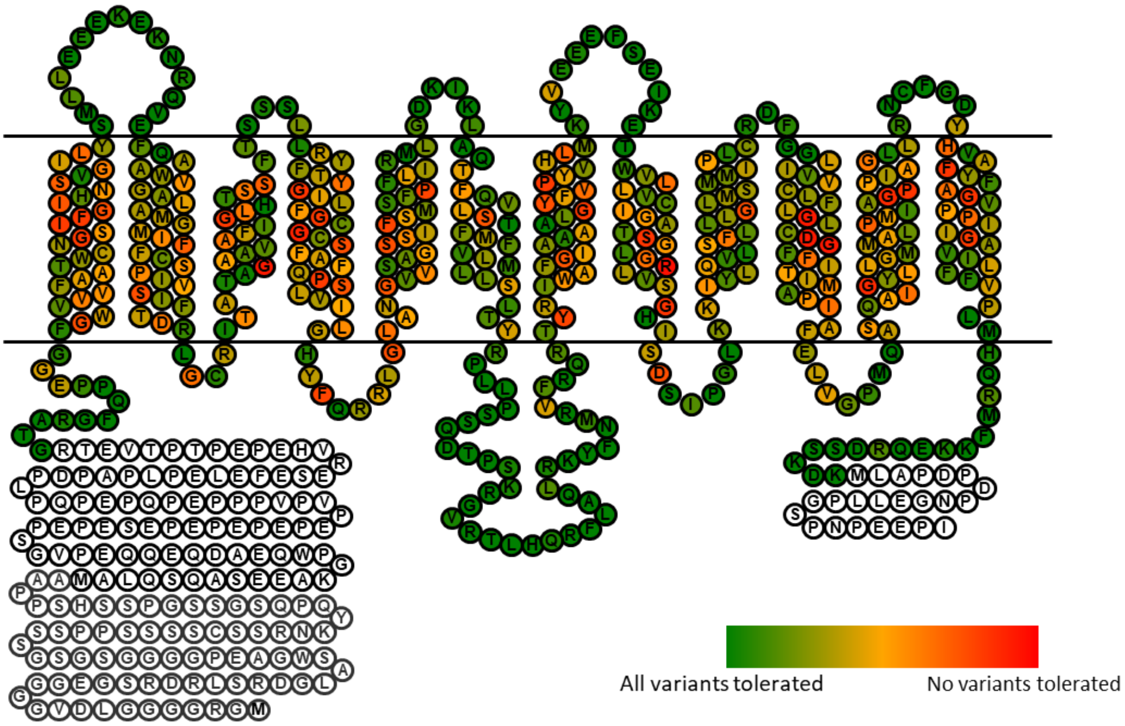

Supplementary Fig. 30. 2D structural model of MCT8 with colors indicating predicted overall tolerability of missense variants from amino acid residue G161 to K590.

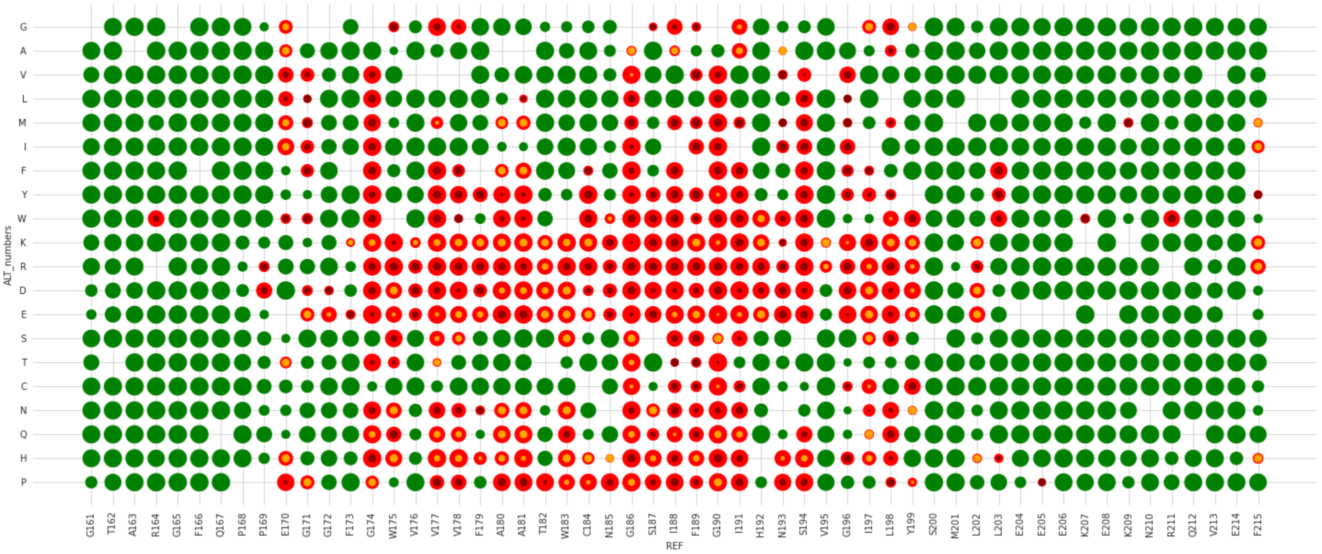
ALT_numbers
G
A
V
L
M
I
F
Y
W
K
R
D
E
S
T
C
N
Q
H
P
REF
G161 T162 A163 R164 G165 F166 Q167 P168 P169 E170 G171 G172 F173 G174 W175 V176 V177 V178 F179 A180 A181 T182 W183 C184 N185 G186 S187 I188 F189 G190 I191 H192 N193 S194 V195 G196 I197 L198 Y199 S200 M201 L202 L203 E204 E205 E206 K207 E208 K209 N210 R211 Q212 V213 E214 F215

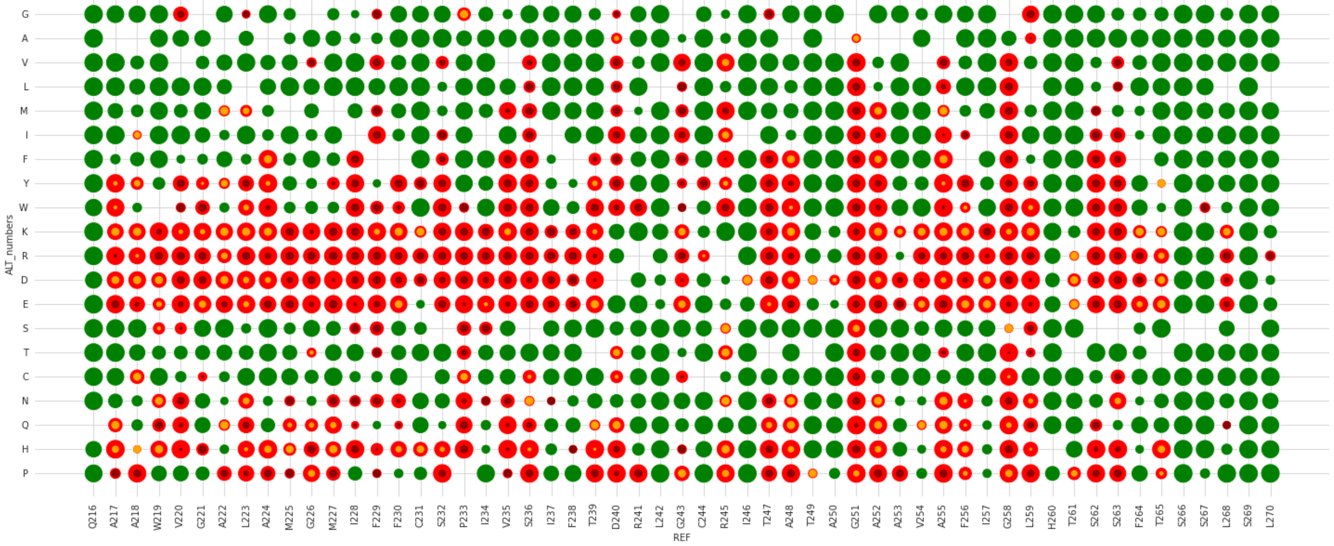
ALT_numbers
G
A
V
L
M
I
F
Y
W
K
R
D
E
S
T
C
N
Q
H
P
REF
Q216 A217 A218 W219 V220 G221 A222 L223 A224 M225 G226 M227 I228 F229 F230 C231 S232 P233 I234 V235 S236 I237 F238 T239 D240 R241 L242 G243 C244 R245 I246 T247 A248 T249 A250 G251 A252 A253 V254 A255 F256 I257 G258 L259 H260 T261 S262 S263 F264 T265 S266 S267 L268 S269 L270

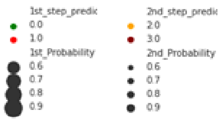
1st_step_predic
0.0
1.0
1st_Probability
0.6
0.7
0.8
0.9
2nd_step_predic
2.0
3.0
2nd_Probability
0.6
0.7
0.8
0.9

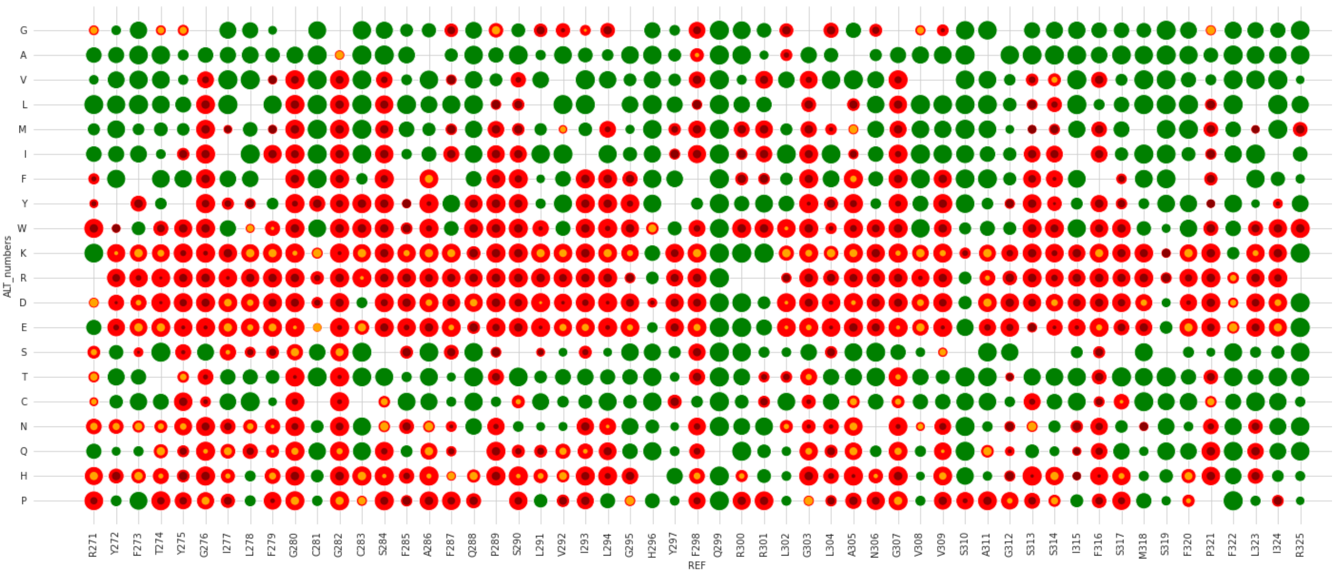
ALT_numbers
G
A
V
L
M
I
F
Y
W
K
R
D
E
S
T
C
N
Q
H
P
R271 Y272 F273 T274 Y275 G276 I277 L278 F279 G280 C281 G282 C283 S284 F285 A286 F287 Q288 P289 S290 L291 V292 I293 L294 G295 H296 Y297 F298 Q299 R300 R301 L302 G303 L304 A305 N306 G307 V308 V309 S310 A311 G312 S313 S314 I315 F316 S317 M318 S319 F320 P321 F322 L323 I324 R325
REF

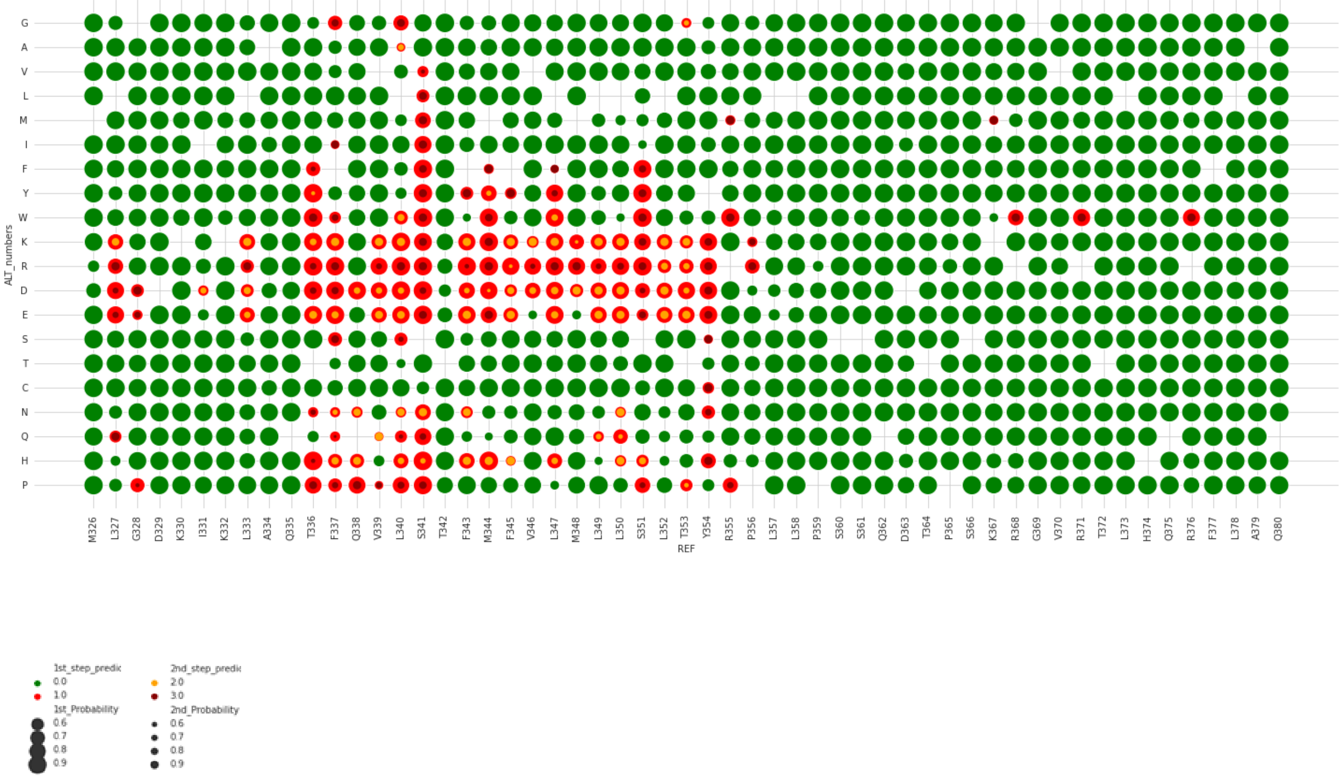
ALT_numbers
G
A
V
L
M
I
F
Y
W
K
R
D
E
S
T
C
N
Q
H
P
M326 L327 G328 D329 K330 I331 K332 L333 A334 Q335 T336 F337 Q338 V339 L340 S341 T342 F343 M344 F345 V346 L347 M348 L349 L350 S351 L352 T353 Y354 R355 P356 L357 L358 P359 S360 S361 Q362 D363 T364 P365 S366 K367 R368 G369 V370 R371 T372 L373 H374 Q375 R376 F377 L378 A379 Q380
REF
1st_step_predic
0.0
1.0
1st_Probability
0.6
0.7
0.8
0.9
2nd_step_predic
2.0
3.0
2nd_Probability
0.6
0.7
0.8
0.9

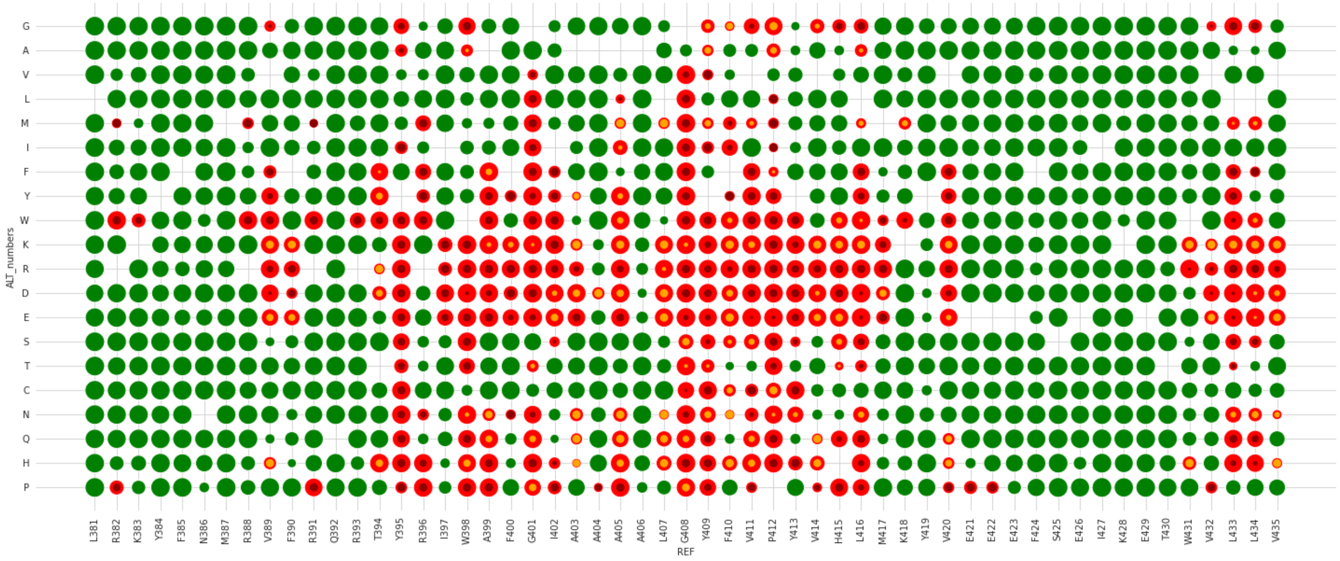
ALT_numbers
REF
G A V L M I F Y W K R D E S T C N Q H P
L381 R382 K383 Y384 F385 N386 M387 R388 V389 F390 R391 Q392 R393 T394 Y395 R396 I397 W398 A399 F400 G401 I402 A403 A404 A405 A406 L407 G408 Y409 F410 V411 P412 Y413 V414 H415 L416 M417 K418 Y419 V420 E421 E422 E423 F424 S425 E426 I427 K428 E429 T430 W431 V432 L433 L434 V435

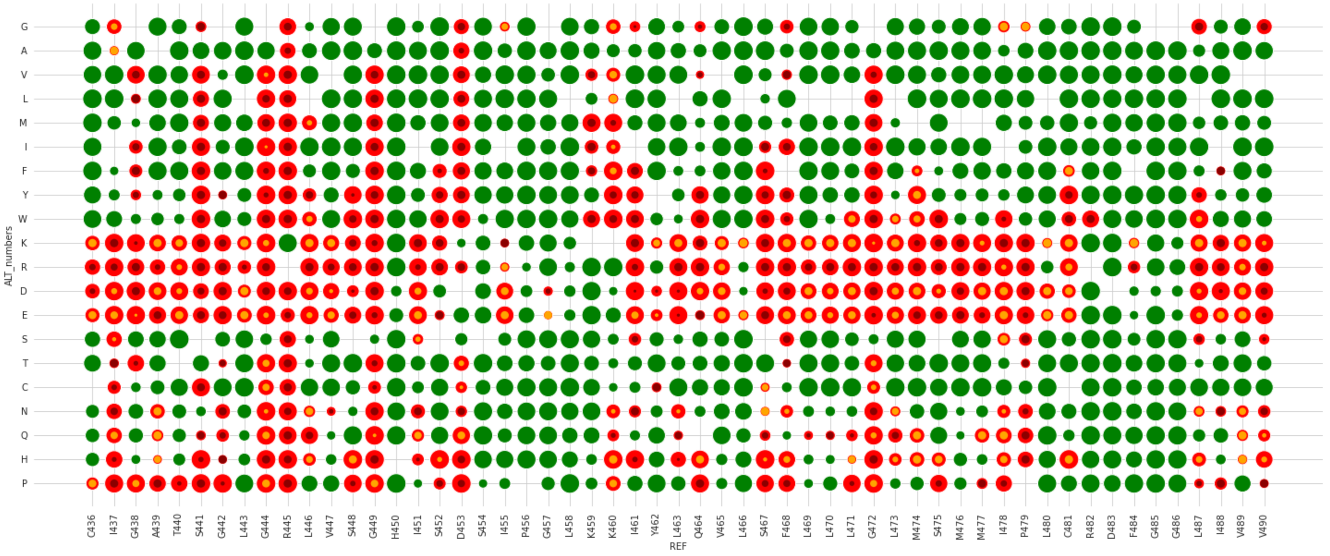
ALT_numbers
REF
G A V L M I F Y W K R D E S T C N Q H P
C436 I437 G438 A439 T440 S441 G442 L443 G444 R445 L446 V447 S448 G449 H450 I451 S452 D453 S454 I455 P456 G457 L458 K459 K460 I461 Y462 L463 Q464 V465 I466 S467 F468 L469 L470 L471 G472 L473 M474 S475 M476 M477 I478 P479 L480 C481 R482 D483 F484 G485 G486 L487 I488 V489 V490

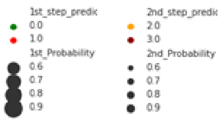
1st_step_predic
0.0
1.0
1st_Probability
0.6
0.7
0.8
0.9
2nd_step_predic
2.0
3.0
2nd_Probability
0.6
0.7
0.8
0.9

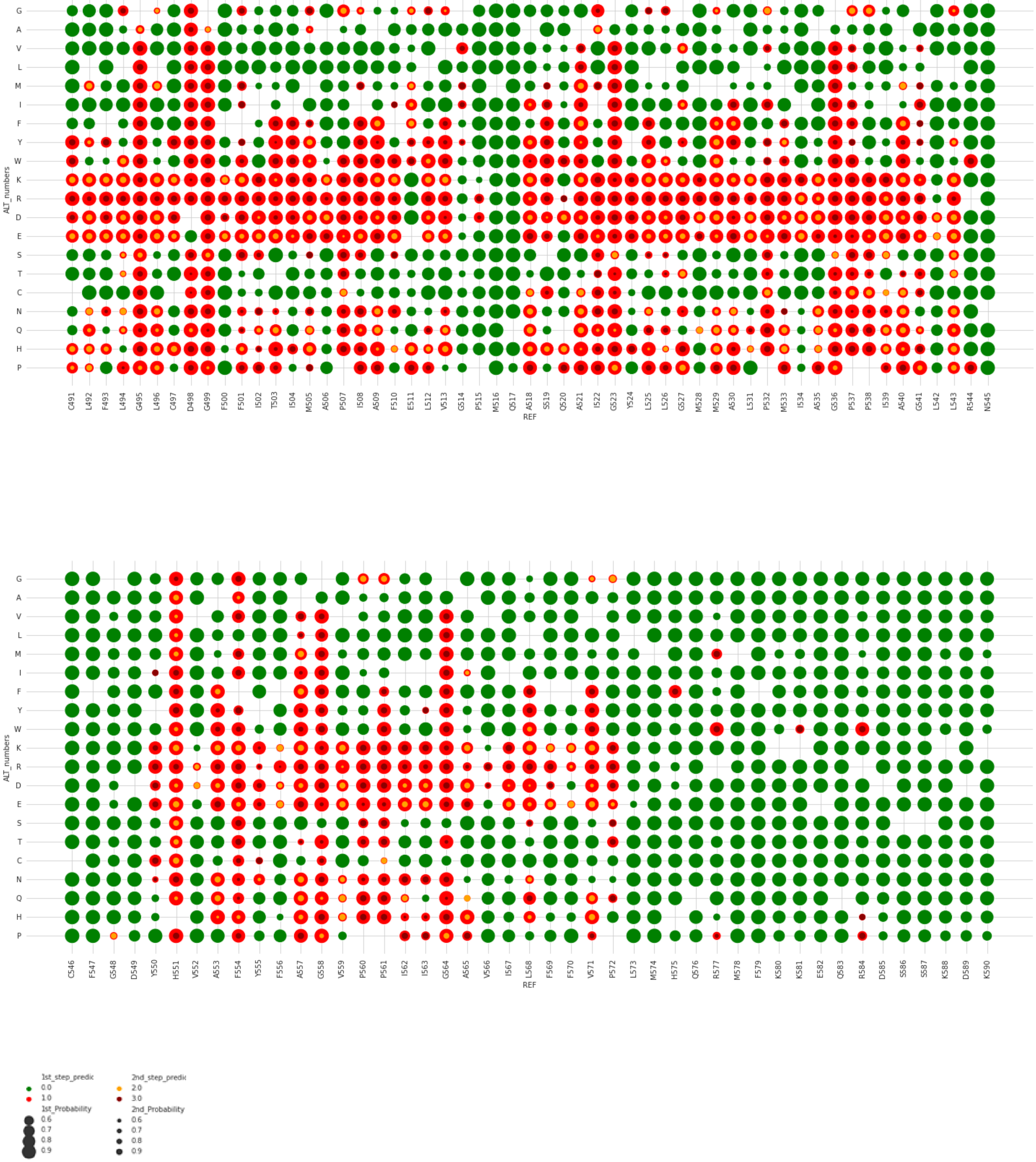

Supplementary Fig. 31. Pathogenicity and disease severity prediction (in case of pathogenic prediction) of all potential missense variants in MCT8 from amino acid residue from G161 to K590. Green: predicted benign; red: predicted pathogenic; yellow in red: predicted mild and moderate LoF;

brown in red: predicted severe LoF; the size of the circle denotes the probability of correct prediction: larger circle, higher probability. Source data are provided in Data Source File.

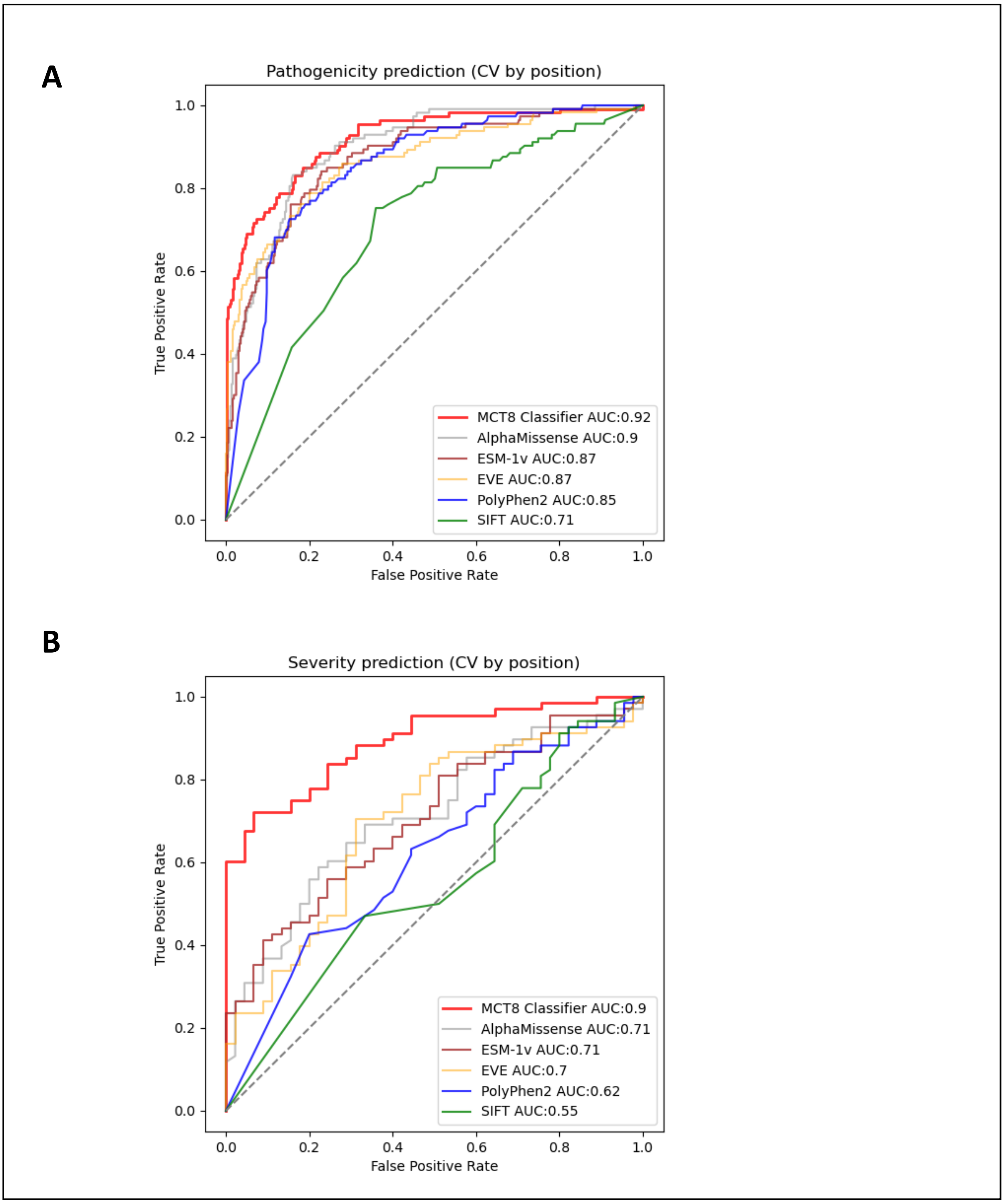

Supplementary Fig. 32. Performance of the MCT8 pathogenicity (A) and severity (B) classifier performance of the classifier by 10-fold cross-validation splitting the dataset by positions. Source data are provided as a Source Data file.

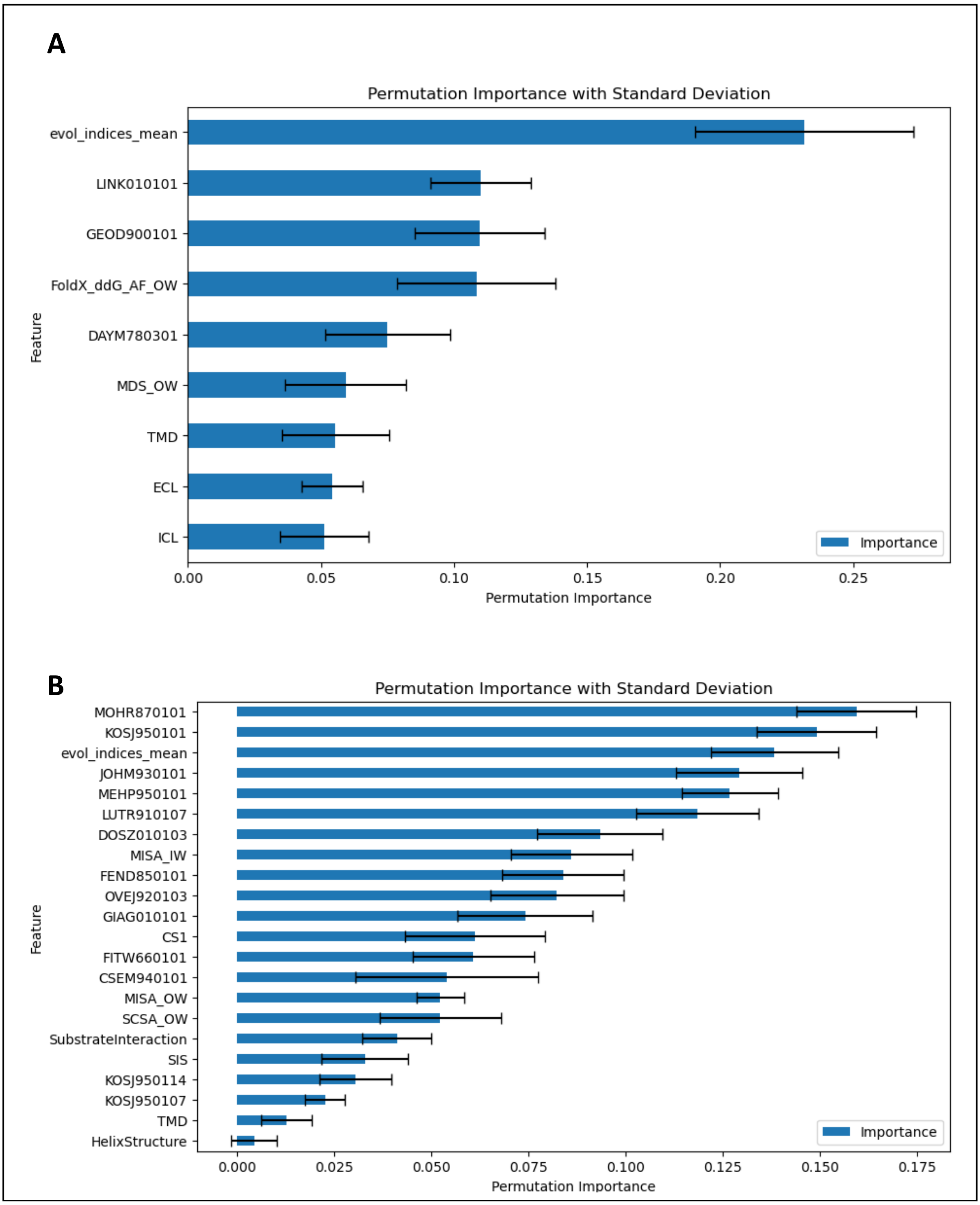

Supplementary Fig. 33. Feature relevance of the MCT8 pathogenicity (A) and severity (B) classifier by performing a feature permutation importance inspection. Source data are provided as a Source Data file. Source data are provided as a Source Data file.

References

1. Li ZL, Buck M. Beyond history and "on a roll": The list of the most well-studied human protein structures and overall trends in the protein data bank. *Protein science : a publication of the Protein Society* **30**, 745-760 (2021).

2. Drew D, North RA, Nagarathinam K, Tanabe M. Structures and General Transport Mechanisms by the Major Facilitator Superfamily (MFS). *Chem Rev* **121**, 5289-5335 (2021).

3. Groeneweg S, Lima de Souza EC, Meima ME, Peeters RP, Visser WE, Visser TJ. Outward-Open Model of Thyroid Hormone Transporter Monocarboxylate Transporter 8 Provides Novel Structural and Functional Insights. *Endocrinology* **158**, 3292-3306 (2017).

4. Groeneweg S, Lima de Souza EC, Visser WE, Peeters RP, Visser TJ. Importance of His192 in the human thyroid hormone transporter MCT8 for substrate recognition. *Endocrinology* **154**, 2525-2532 (2013).

5. Lima de Souza EC, Groeneweg S, Visser WE, Peeters RP, Visser TJ. Importance of cysteine residues in the thyroid hormone transporter MCT8. *Endocrinology* **154**, 1948-1955 (2013).

6. Wilson MC, Meredith D, Bunnun C, Sessions RB, Halestrap AP. Studies on the DIDS-binding site of monocarboxylate transporter 1 suggest a homology model of the open conformation and a plausible translocation cycle. *J Biol Chem* **284**, 20011-20021 (2009).

7. Rahman B, Schneider HP, Broer A, Deitmer JW, Broer S. Helix 8 and helix 10 are involved in substrate recognition in the rat monocarboxylate transporter MCT1. *Biochemistry* **38**, 11577-11584 (1999).

8. Ueda S*, et al.* Differences in response of thyrotropin to 3,5,3'-triiodothyronine and 3,5,3'-triiodothyroacetic acid in patients with resistance to thyroid hormone. *Thyroid* **6**, 563-570 (1996).

9. Wejaphikul K*, et al.* Insight Into Molecular Determinants of T3 vs T4 Recognition From Mutations in Thyroid Hormone Receptor alpha and beta. *J Clin Endocrinol Metab* **104**, 3491-3500 (2019).

10. Wejaphikul K*, et al.* Role of leucine 341 in thyroid hormone receptor beta revealed by a novel mutation causing thyroid hormone resistance. *Thyroid*, (2018).

11. Jumper J*, et al.* Highly accurate protein structure prediction with AlphaFold. *Nature* **596**, 583-589 (2021).

12. van Geest FS*, et al.* Clinical and Functional Consequences of C-Terminal Variants in MCT8: A Case Series. *J Clin Endocrinol Metab* **106**, 539-553 (2021).

13. Groeneweg S, van den Berge A, Lima de Souza EC, Meima ME, Peeters RP, Visser WE. Insights Into the Mechanism of MCT8 Oligomerization. *J Endocr Soc* **4**, bvaa080 (2020).

14. Zhang XC, Zhao Y, Heng J, Jiang D. Energy coupling mechanisms of MFS transporters. *Protein science : a publication of the Protein Society* **24**, 1560-1579 (2015).

15. Wang N*, et al.* Structural basis of human monocarboxylate transporter 1 inhibition by anti-cancer drug candidates. *Cell* **184**, 370-383 e313 (2021).

16. Groeneweg S*, et al.* The role of Arg445 and Asp498 in the human thyroid hormone transporter MCT8. *Endocrinology* **155**, 618-626 (2014).

17. Kinne A*, et al.* Essential molecular determinants for thyroid hormone transport and first structural implications for monocarboxylate transporter 8. *J Biol Chem* **285**, 28054-28063 (2010).

18. Protze J, Braun D, Hinz KM, Bayer-Kusch D, Schweizer U, Krause G. Membrane-traversing mechanism of thyroid hormone transport by monocarboxylate transporter 8. *Cell Mol Life Sci* **74**, 2299-2318 (2017).

19. van Hasselt PM, Ferdinandusse S, van Haaften G. Monocarboxylate transporter 1 deficiency and ketone utilization. *N Engl J Med* **372**, 578-579 (2015).

20. Moradi M, Enkavi G, Tajkhorshid E. Atomic-level characterization of transport cycle thermodynamics in the glycerol-3-phosphate:phosphate antiporter. *Nat Commun* **6**, 8393 (2015).

21. Law CJ*, et al.* Salt-bridge dynamics control substrate-induced conformational change in the membrane transporter GlpT. *Journal of molecular biology* **378**, 828-839 (2008).

22. Dang S*, et al.* Structure of a fucose transporter in an outward-open conformation. *Nature* **467**, 734-738 (2010).

23. Zimmermann MO, Lange A, Zahn S, Exner TE, Boeckler FM. Using Surface Scans for the Evaluation of Halogen Bonds toward the Side Chains of Aspartate, Asparagine, Glutamate, and Glutamine. *Journal of chemical information and modeling* **56**, 1373-1383 (2016).

24. Johannes J, Braun D, Kinne A, Rathmann D, Kohrle J, Schweizer U. Few Amino Acid Exchanges Expand the Substrate Spectrum of Monocarboxylate Transporter 10. *Mol Endocrinol* **30**, 796-808 (2016).

25. Groeneweg S*, et al.* Disease characteristics of MCT8 deficiency: an international, retrospective, multicentre cohort study. *Lancet Diabetes Endocrinol* **8**, 594-605 (2020).

26. Karczewski KJ*, et al.* The mutational constraint spectrum quantified from variation in 141,456 humans. *Nature* **581**, 434-443 (2020).

27. RRID:AB_1079343, https://scicrunch.org/resolver/AB_1079343.

28. RRID:AB_2107445, https://scicrunch.org/resolver/AB_2107445.

29. RRID:AB_2533147, https://scicrunch.org/resolver/AB_2533147.

30. RRID:AB_621843, https://scicrunch.org/resolver/AB_621843.

31. RRID:AB_10706161, https://scicrunch.org/resolver/AB_10706161.

32. RRID:AB_143165, https://scicrunch.org/resolver/AB_143165.

33. RRID:AB_2535718, https://scicrunch.org/resolver/AB_2535718.

34. Groeneweg S*, et al.* Effectiveness and safety of the tri-iodothyronine analogue Triac in children and adults with MCT8 deficiency: an international, single-arm, open-label, phase 2 trial. *Lancet Diabetes Endocrinol* **7**, 695-706 (2019).

35. van Geest FS*, et al.* Long-Term Efficacy of T3 Analogue Triac in Children and Adults With MCT8 Deficiency: A Real-Life Retrospective Cohort Study. *J Clin Endocrinol Metab* **107**, e1136-e1147 (2022).

36. https://www.mapchart.net/.).

37. https://www.prb.org/international/indicator/population/snapshot.).

38. van Hasselt PM*, et al.* Monocarboxylate transporter 1 deficiency and ketone utilization. *N Engl J Med* **371**, 1900-1907 (2014).

39. Zhang B*, et al.* Cooperative transport mechanism of human monocarboxylate transporter 2. *Nat Commun* **11**, 2429 (2020).

40. Sasaki S*, et al.* Crucial residue involved in L-lactate recognition by human monocarboxylate transporter 4 (hMCT4). *PLoS One* **8**, e67690 (2013).

41. Dhayat N*, et al.* Mutation in the Monocarboxylate Transporter 12 Gene Affects Guanidinoacetate Excretion but Does Not Cause Glucosuria. *J Am Soc Nephrol* **27**, 1426-1436 (2016).

42. Staubli A*, et al.* Abnormal creatine transport of mutations in monocarboxylate transporter 12 (MCT12) found in patients with age-related cataract can be partially rescued by exogenous chaperone CD147. *Hum Mol Genet* **26**, 4203-4214 (2017).

43. Nancolas B, Sessions RB, Halestrap AP. Identification of key binding site residues of MCT1 for AR-C155858 reveals the molecular basis of its isoform selectivity. *Biochem J* **466**, 177-188 (2015).

44. Merezhinskaya N, Fishbein WN, Davis JI, Foellmer JW. Mutations in MCT1 cDNA in patients with symptomatic deficiency in lactate transport. *Muscle Nerve* **23**, 90-97 (2000).

45. Krieger E, Vriend G. New ways to boost molecular dynamics simulations. *J Comput Chem* **36**, 996-1007 (2015).

Original full immunoblots

Original full (uncropped) immunoblots

Series 1

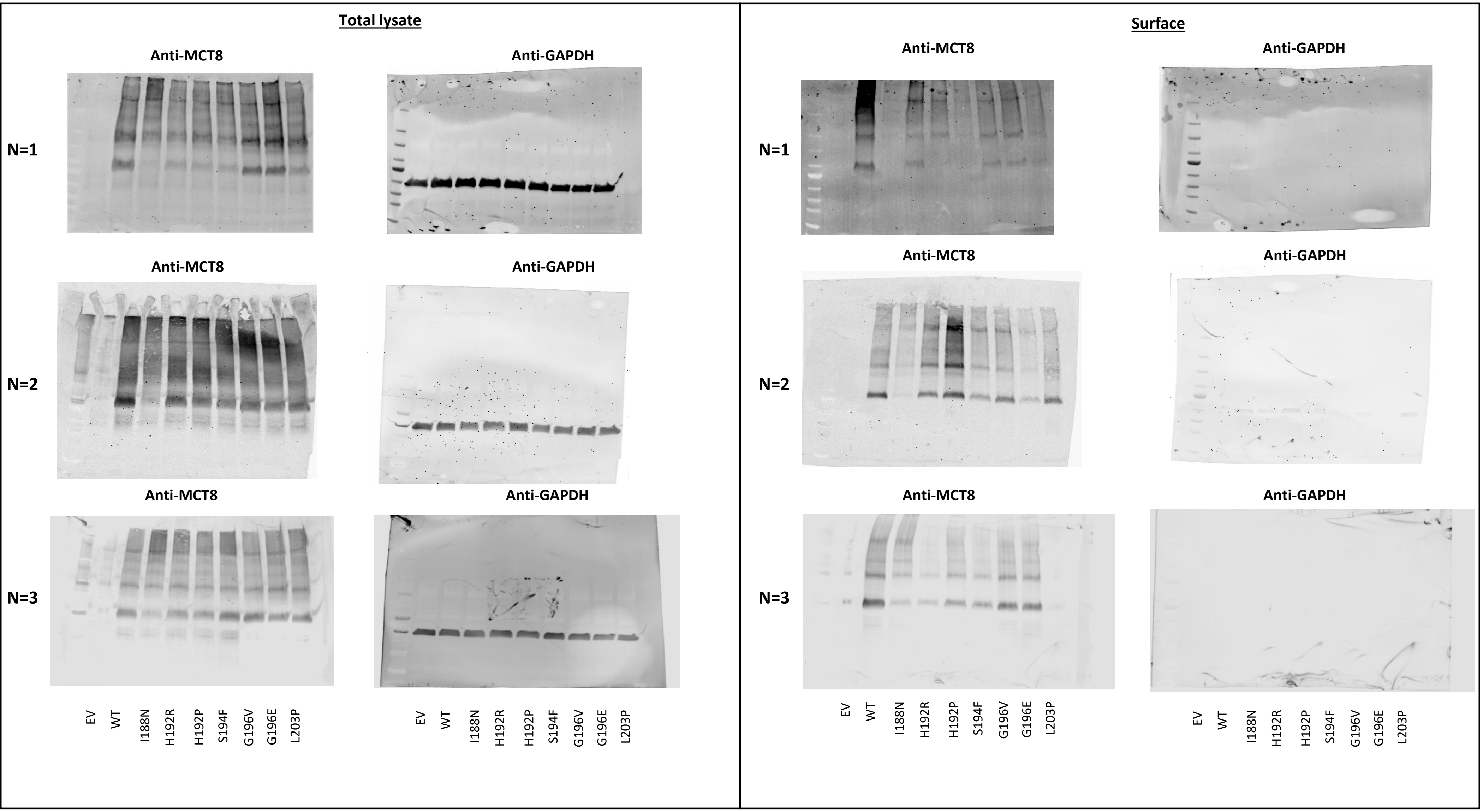
Total lysate
Surface
Anti-MCT8
Anti-GAPDH
Anti-MCT8
Anti-GAPDH
N=1
Anti-MCT8
Anti-GAPDH
Anti-MCT8
Anti-GAPDH
N=2
Anti-MCT8
Anti-GAPDH
Anti-MCT8
Anti-GAPDH
N=3
EV WT I188N H192R H192P S194F G196V G196E L203P
EV WT I188N H192R H192P S194F G196V G196E L203P
EV WT I188N H192R H192P S194F G196V G196E L203P
EV WT I188N H192R H192P S194F G196V G196E L203P

Series 2

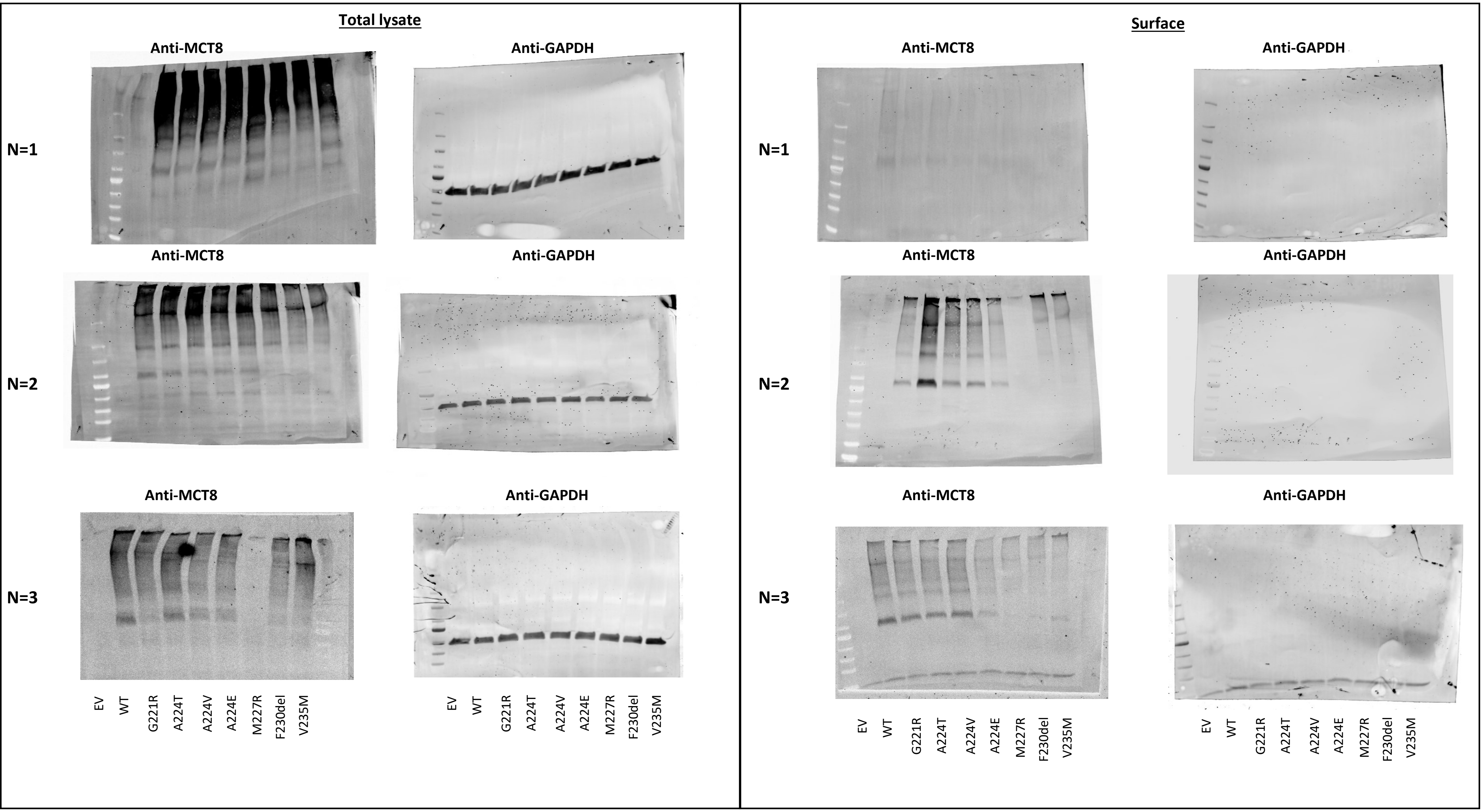

Total lysate
Surface
Anti-MCT8
Anti-GAPDH
N=1
N=2
N=3
EV
WT
G221R
A224T
A224V
A224E
M227R
F230del
V235M

Series 3

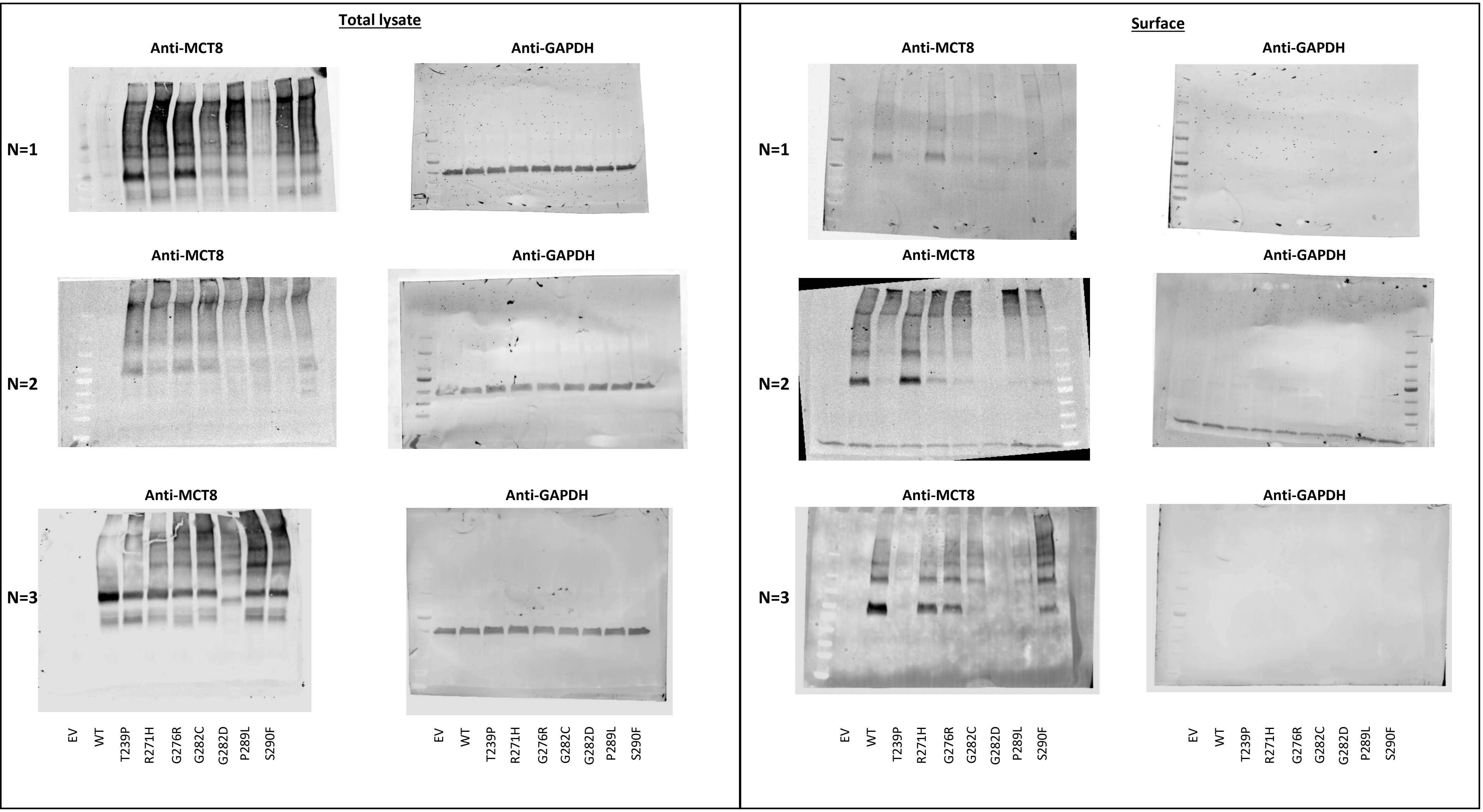

Total lysate
Surface
Anti-MCT8
Anti-GAPDH
Anti-MCT8
Anti-GAPDH
N=1
Anti-MCT8
Anti-GAPDH
Anti-MCT8
Anti-GAPDH
N=2
Anti-MCT8
Anti-GAPDH
Anti-MCT8
Anti-GAPDH
N=3
EV
WT
T239P
R271H
G276R
G282C
G282D
P289L
S290F

Series 4

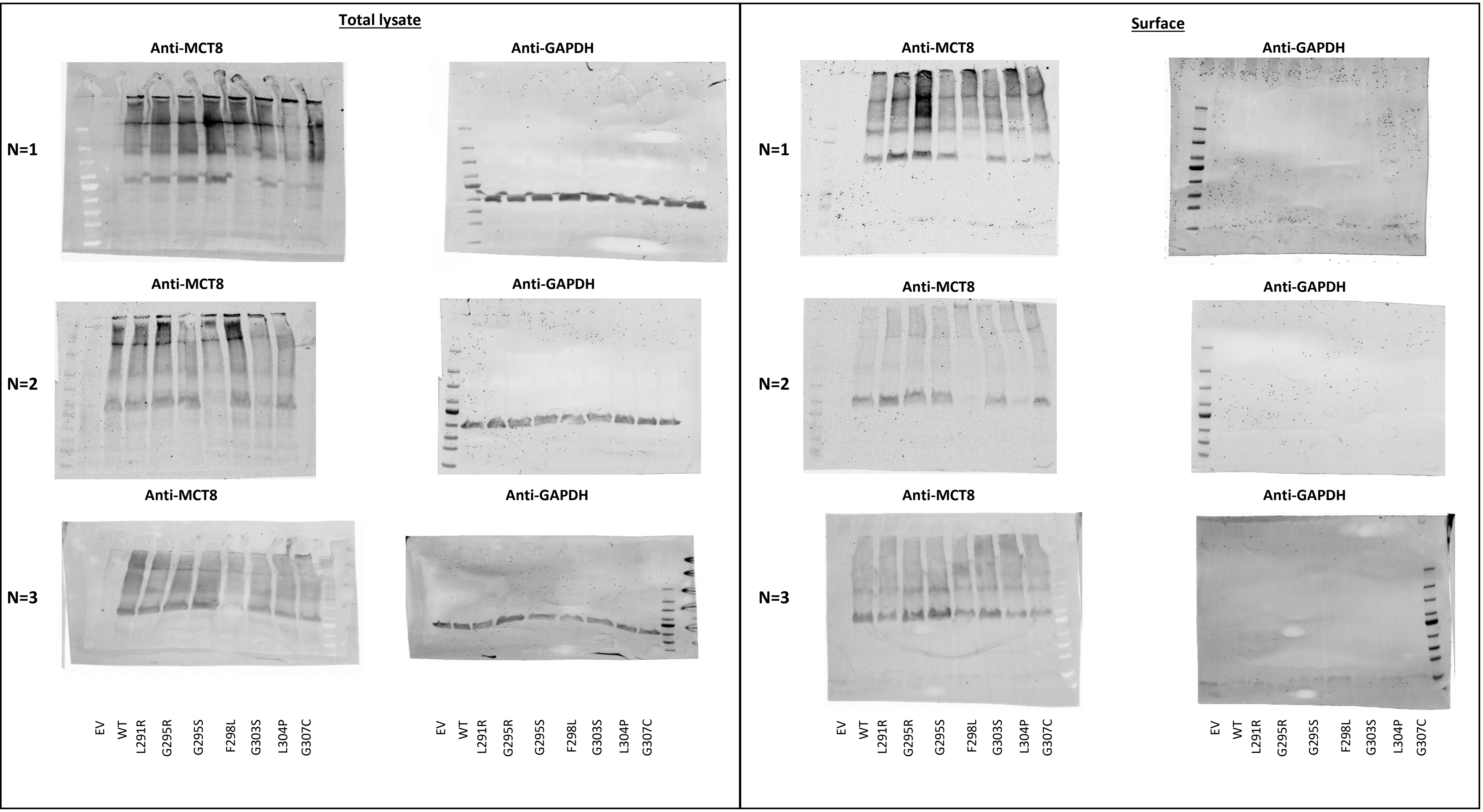
Total lysate
Surface
Anti-MCT8
Anti-GAPDH
N=1
N=2
N=3
EV
WT
L291R
G295R
G295S
F298L
G303S
L304P
G307C

Series 5

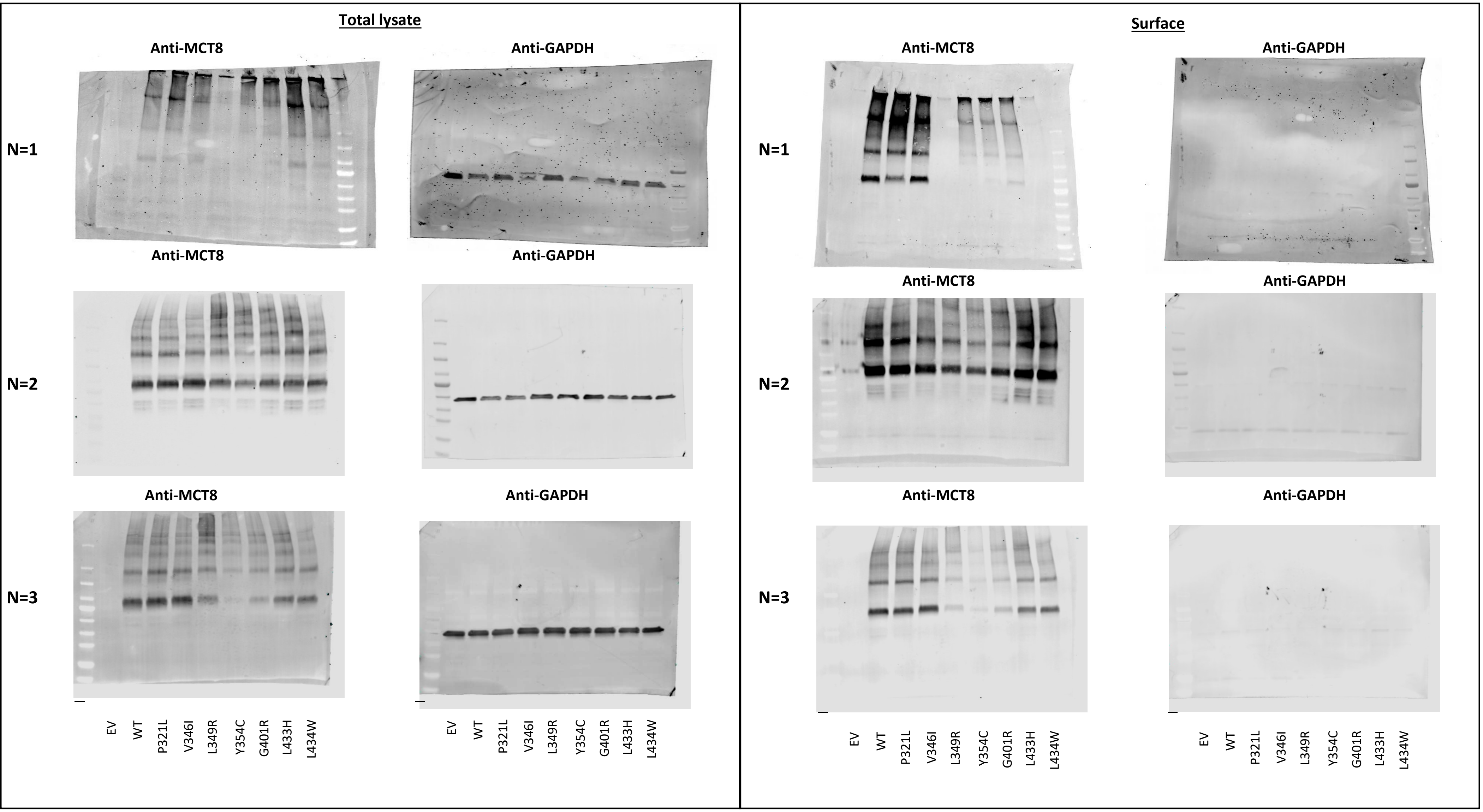

Total lysate
Surface
Anti-MCT8
Anti-GAPDH
Anti-MCT8
Anti-GAPDH
N=1
N=2
N=3
EV
WT
P321L
V346I
L349R
Y354C
G401R
L433H
L434W

Series 6

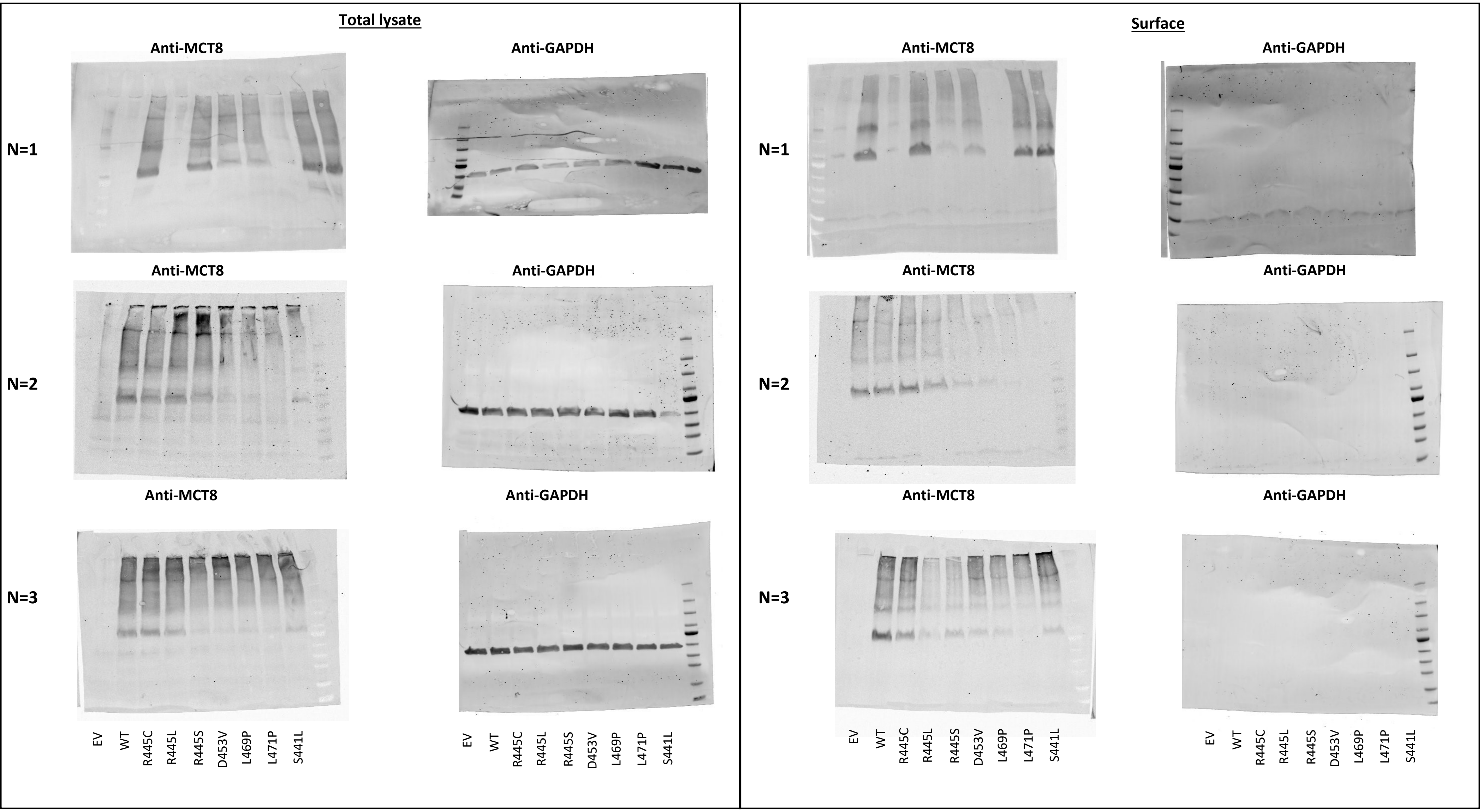
Total lysate
Surface
Anti-MCT8
Anti-GAPDH
Anti-MCT8
Anti-GAPDH
N=1
Anti-MCT8
Anti-GAPDH
Anti-MCT8
Anti-GAPDH
N=2
Anti-MCT8
Anti-GAPDH
Anti-MCT8
Anti-GAPDH
N=3
EV WT R445C R445L R445S D453V L469P L471P S441L
EV WT R445C R445L R445S D453V L469P L471P S441L
EV WT R445C R445L R445S D453V L469P L471P S441L
EV WT R445C R445L R445S D453V L469P L471P S441L

Series 7

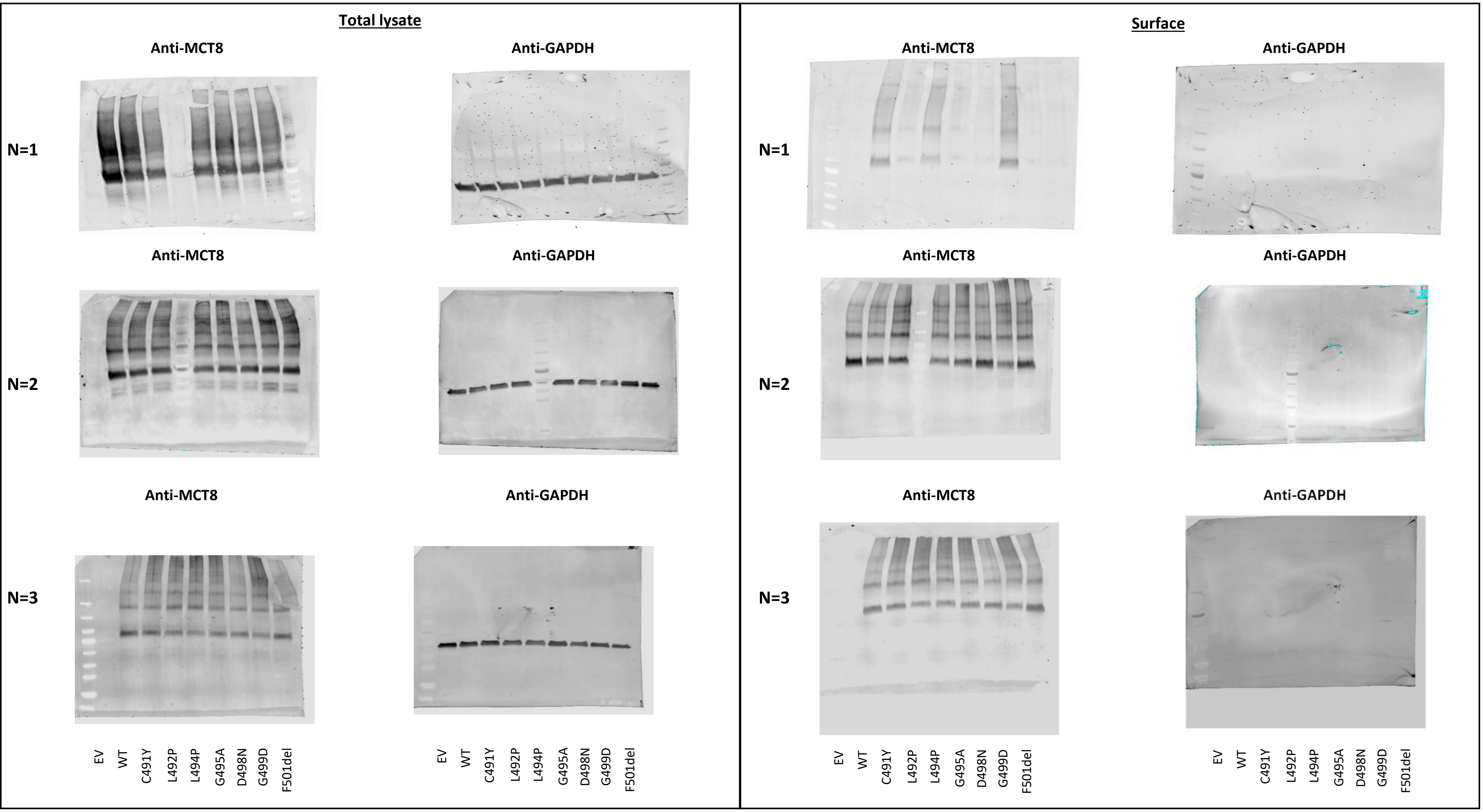

Total lysate
Anti-MCT8
Anti-GAPDH
N=1
Anti-MCT8
Anti-GAPDH
N=2
Anti-MCT8
Anti-GAPDH
N=3
EV WT C491Y L492P L494P G495A D498N G499D F501del
EV WT C491Y L492P L494P G495A D498N G499D F501del
Surface
Anti-MCT8
Anti-GAPDH
N=1
Anti-MCT8
Anti-GAPDH
N=2
Anti-MCT8
Anti-GAPDH
N=3
EV WT C491Y L492P L494P G495A D498N G499D F501del
EV WT C491Y L492P L494P G495A D498N G499D F501del

Series 8

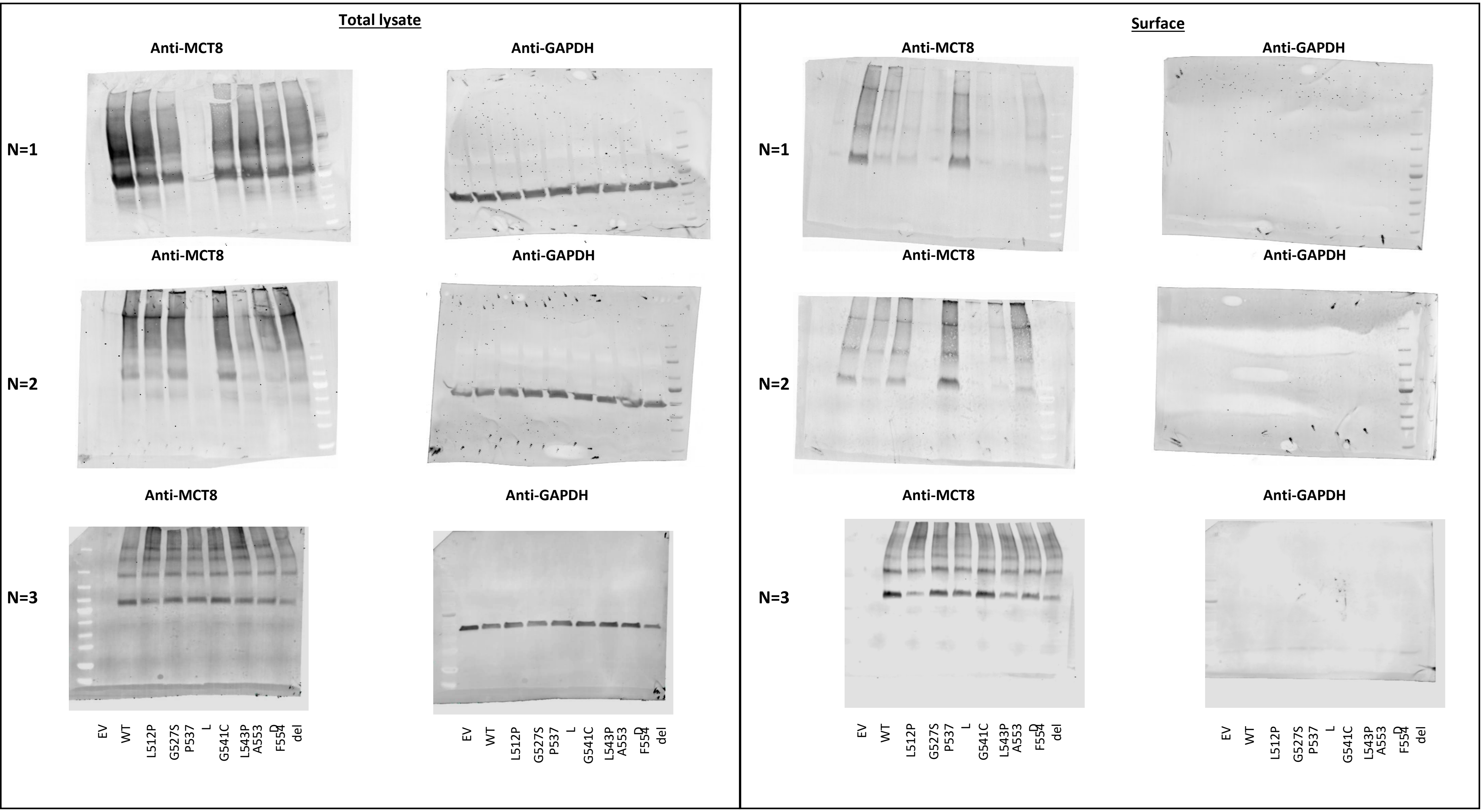
Total lysate
Surface
Anti-MCT8
Anti-GAPDH
N=1
N=2
N=3
EV
WT
L512P
G527S
P537
L
G541C
L543P
A553
D
F554
del

Series 9

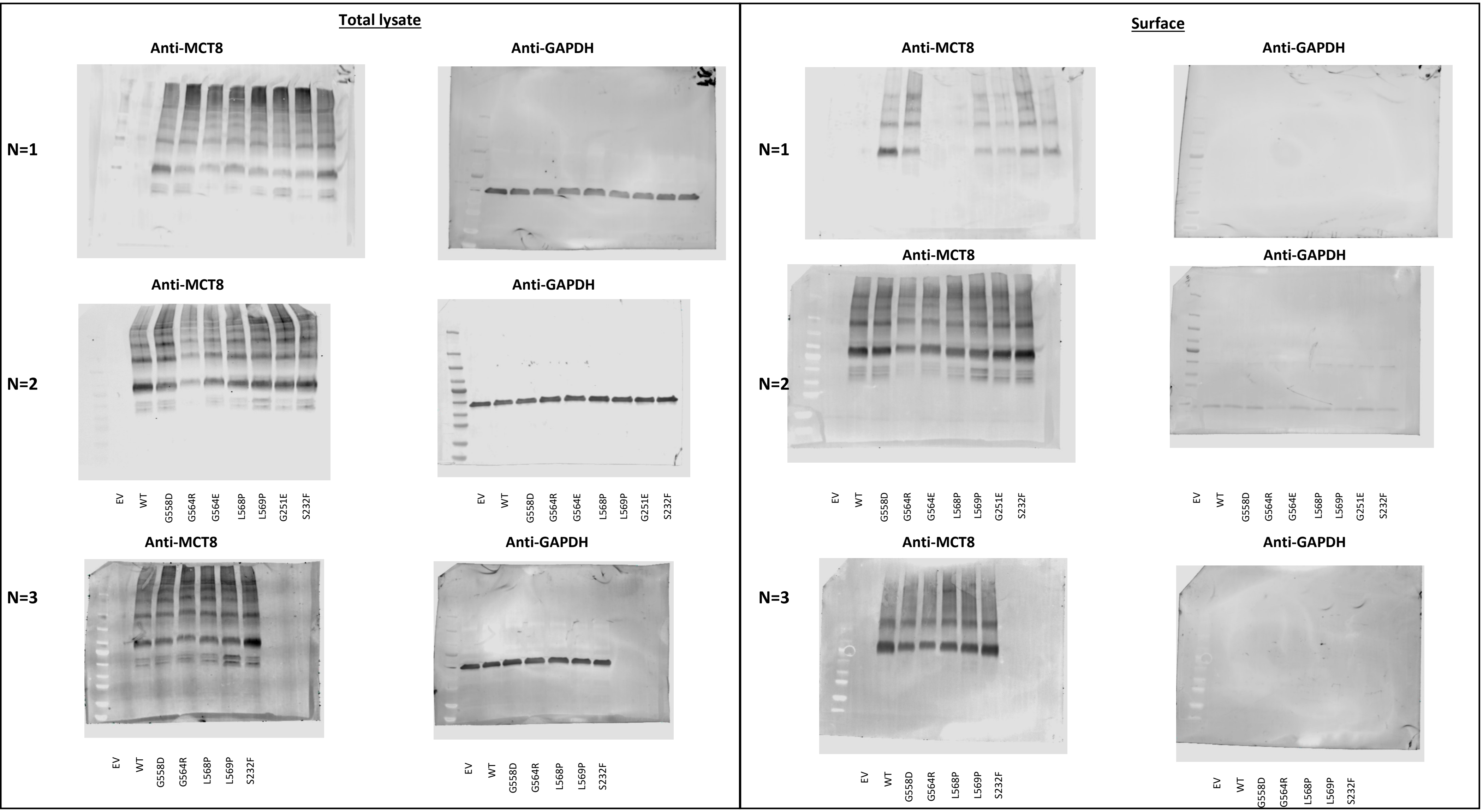

Total lysate
Surface
N=1
N=2
N=3
Anti-MCT8
Anti-GAPDH
Anti-MCT8
Anti-GAPDH
Anti-MCT8
Anti-GAPDH
Anti-MCT8
Anti-GAPDH
Anti-MCT8
Anti-GAPDH
Anti-MCT8
Anti-GAPDH
EV WT G558D G564R G564E L568P L569P G251E S232F
EV WT G558D G564R G564E L568P L569P G251E S232F
EV WT G558D G564R G564E L568P L569P G251E S232F
EV WT G558D G564R G564E L568P L569P G251E S232F
EV WT G558D G564R L568P L569P S232F
EV WT G558D G564R L568P L569P S232F
EV WT G558D G564R L568P L569P S232F
EV WT G558D G564R L568P L569P S232F

Series 10

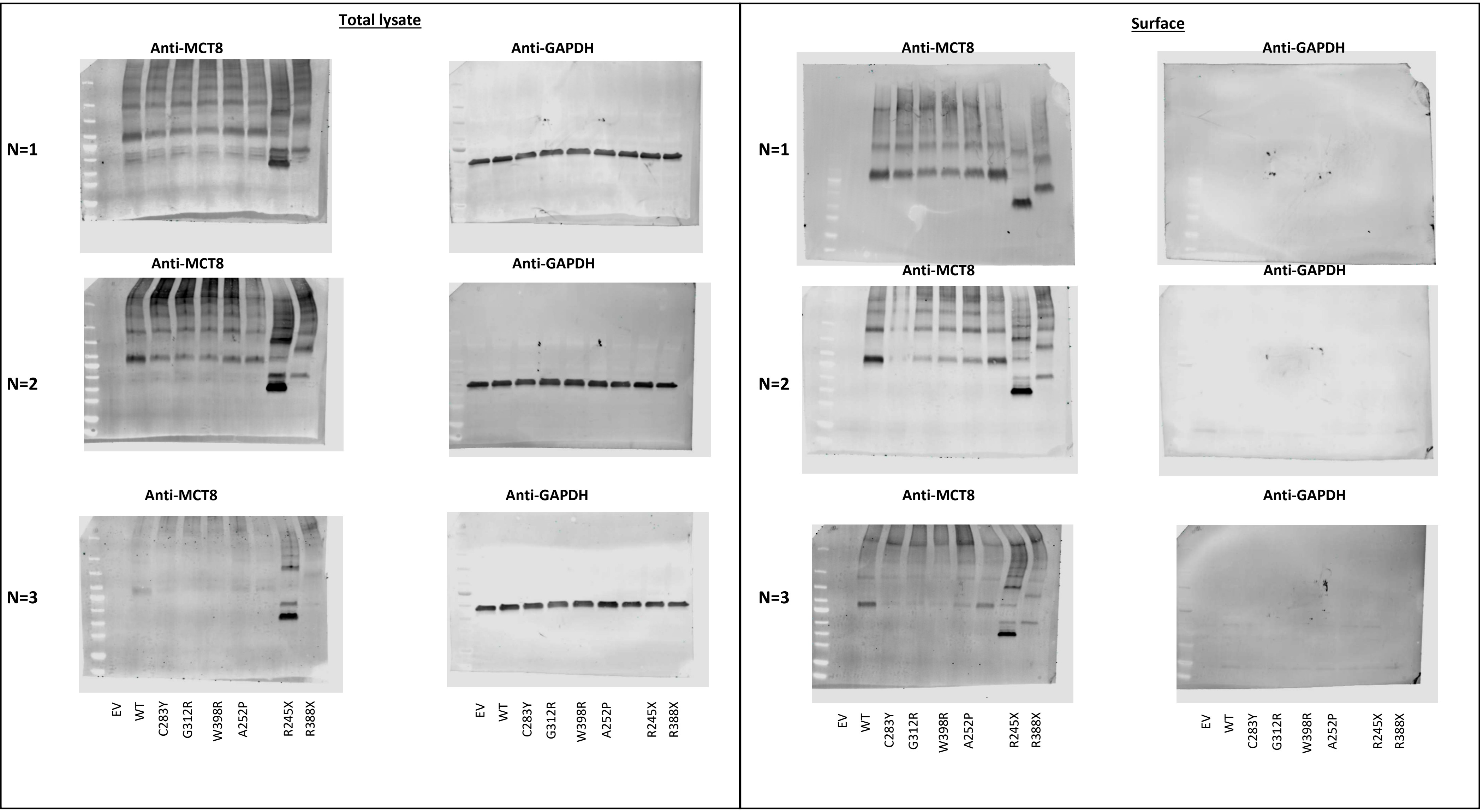
Total lysate
Surface
Anti-MCT8
Anti-GAPDH
N=1
N=2
N=3
EV
WT
C283Y
G312R
W398R
A252P
R245X
R388X

Series 11

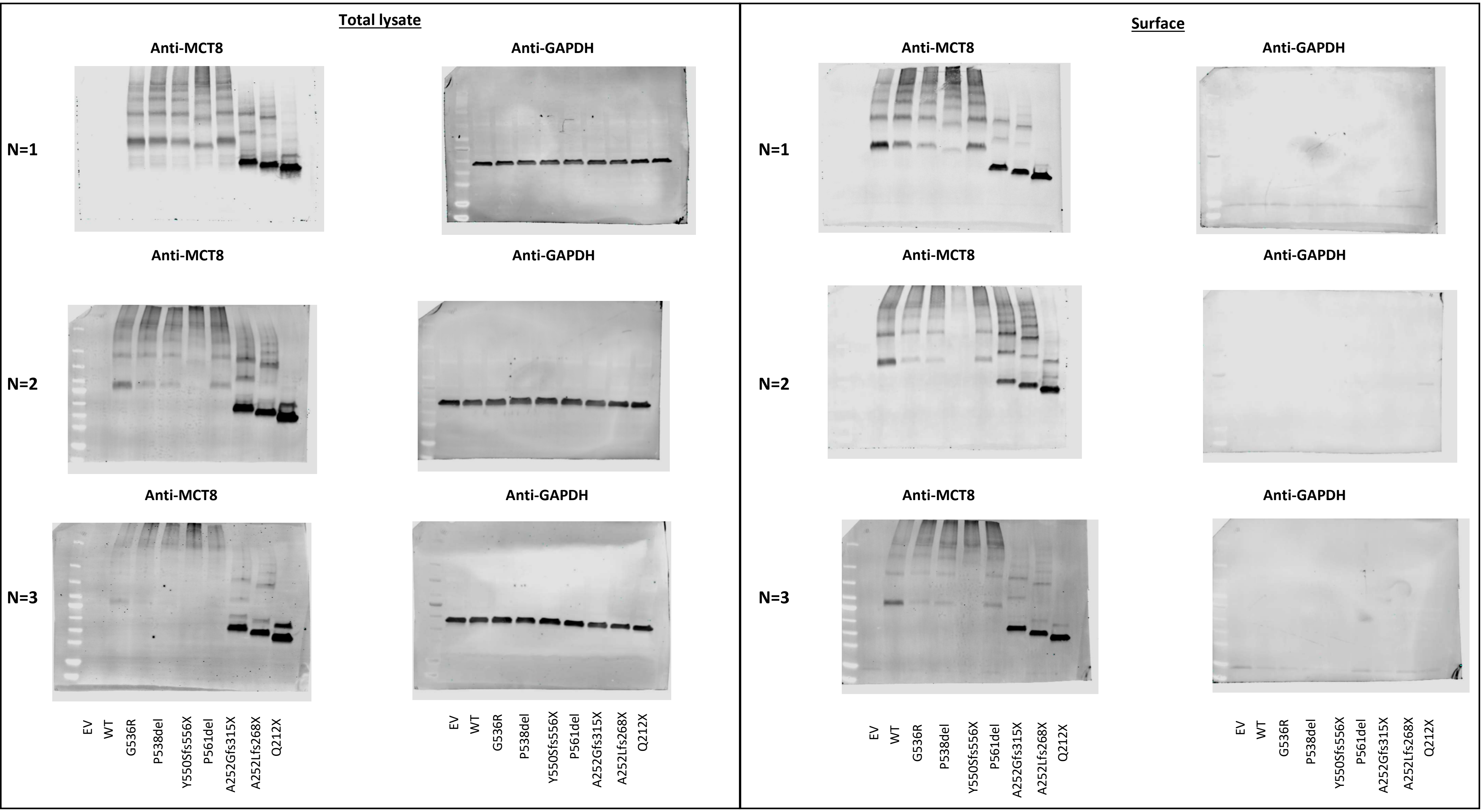
Total lysate
Anti-MCT8
Anti-GAPDH
N=1
N=2
N=3
EV
WT
G536R
P538del
Y550Sfs556X
P561del
A252Gfs315X
A252Lfs268X
Q212X
Surface
Anti-MCT8
Anti-GAPDH
N=1
N=2
N=3
EV
WT
G536R
P538del
Y550Sfs556X
P561del
A252Gfs315X
A252Lfs268X
Q212X

Series 12

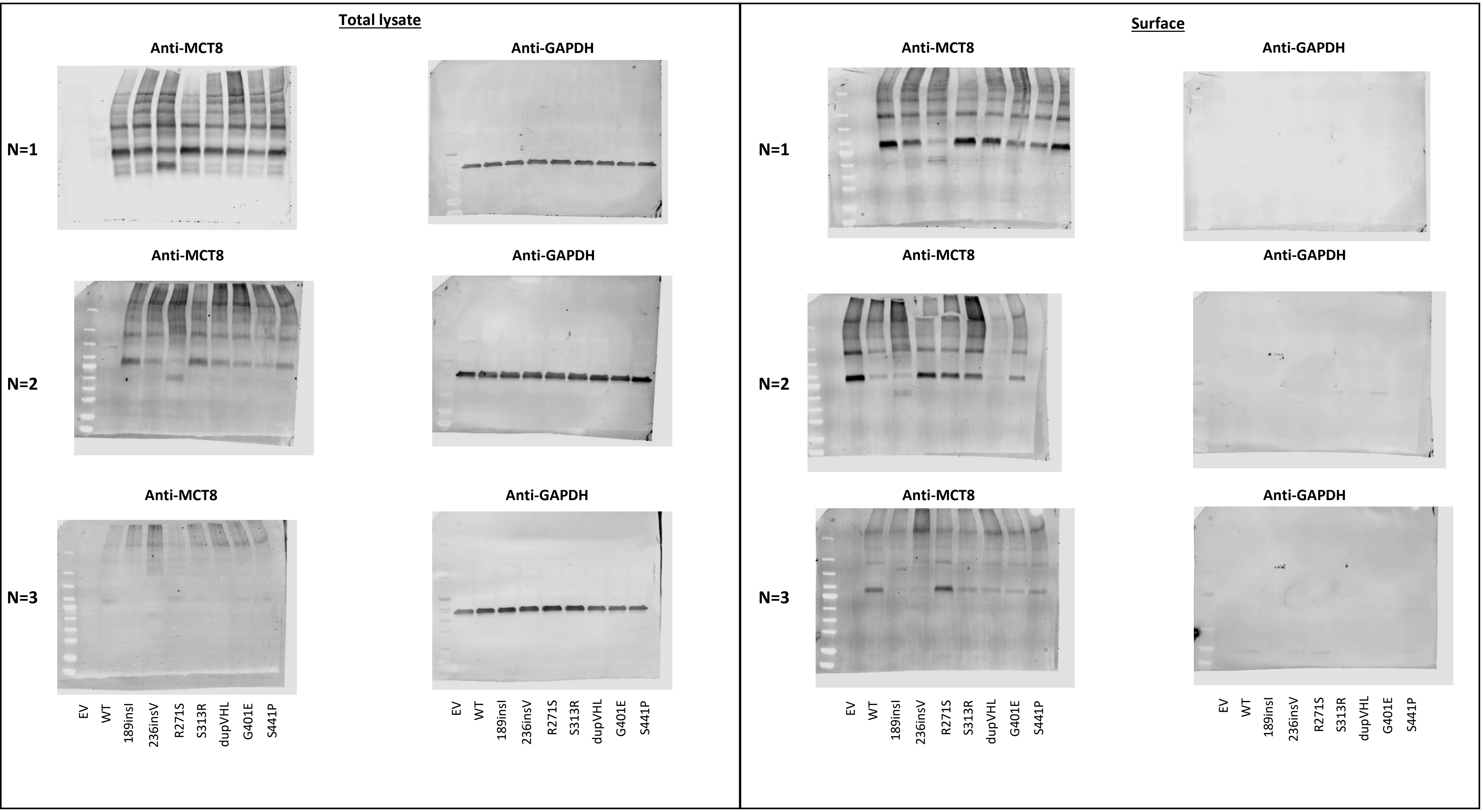

Total lysate
Surface
Anti-MCT8
Anti-GAPDH
N=1
N=2
N=3
EV
WT
189insI
236insV
R271S
S313R
dupVHL
G401E
S441P

Series 13

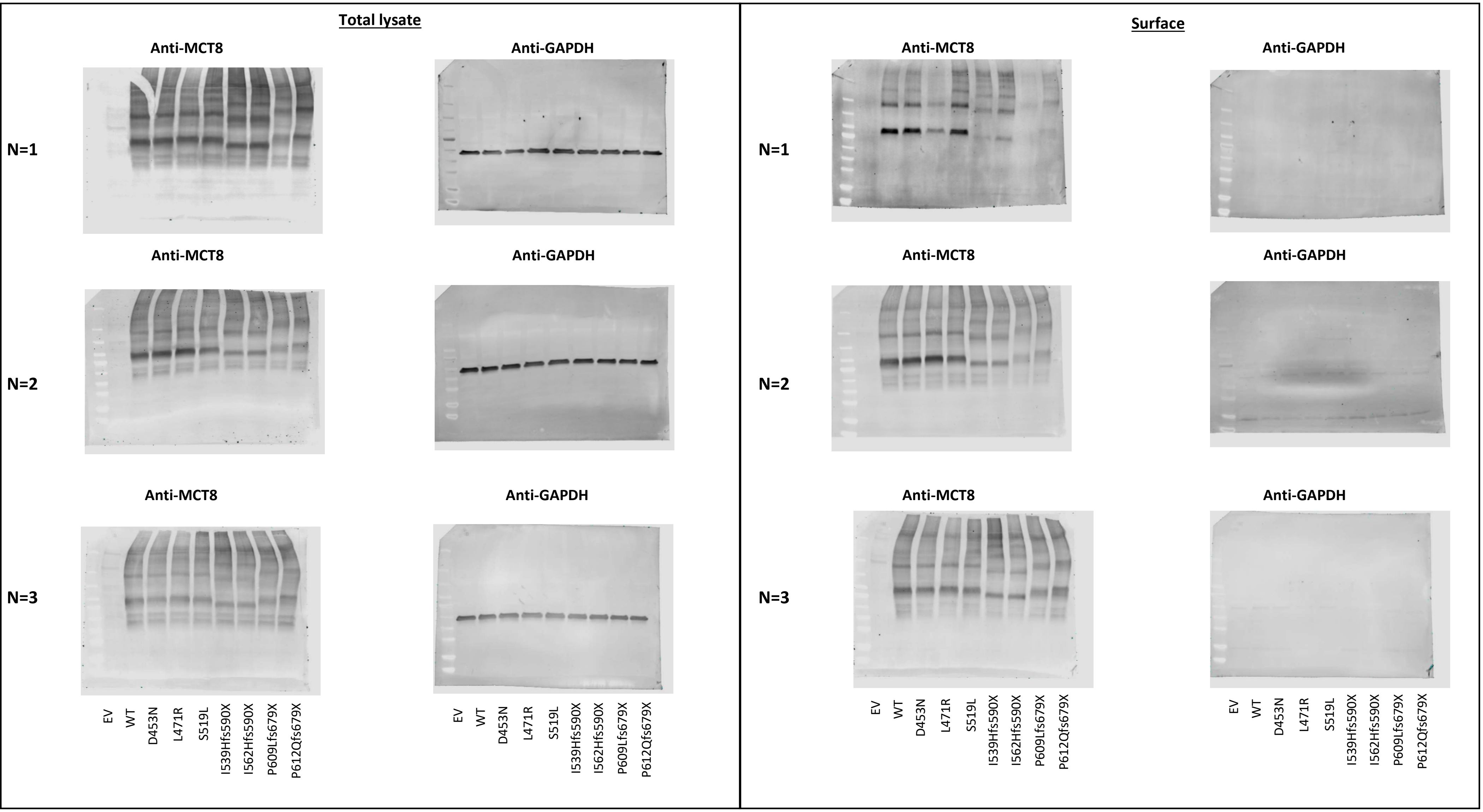

Total lysate
Surface
Anti-MCT8
Anti-GAPDH
Anti-MCT8
Anti-GAPDH
N=1
Anti-MCT8
Anti-GAPDH
Anti-MCT8
Anti-GAPDH
N=2
Anti-MCT8
Anti-GAPDH
Anti-MCT8
Anti-GAPDH
N=3
EV
WT
D453N
L471R
S519L
I539Hfs590X
I562Hfs590X
P609Lfs679X
P612Qfs679X

Series 14

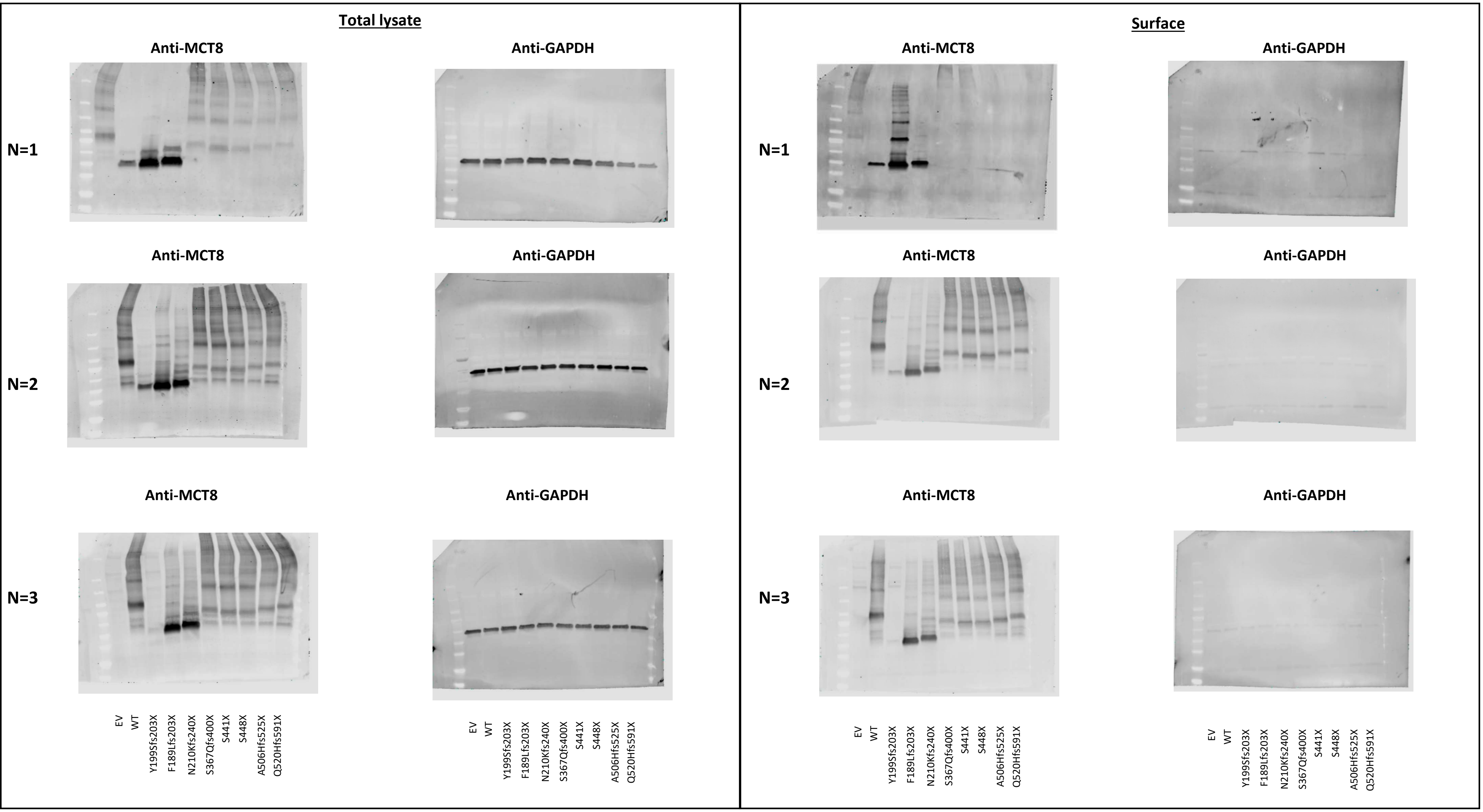
Total lysate
Surface
Anti-MCT8
Anti-GAPDH
N=1
N=2
N=3
EV
WT
Y199Sfs203X
F189Lfs203X
N210Kfs240X
S367Qfs400X
S441X
S448X
A506Hfs525X
Q520Hfs591X

Series 15

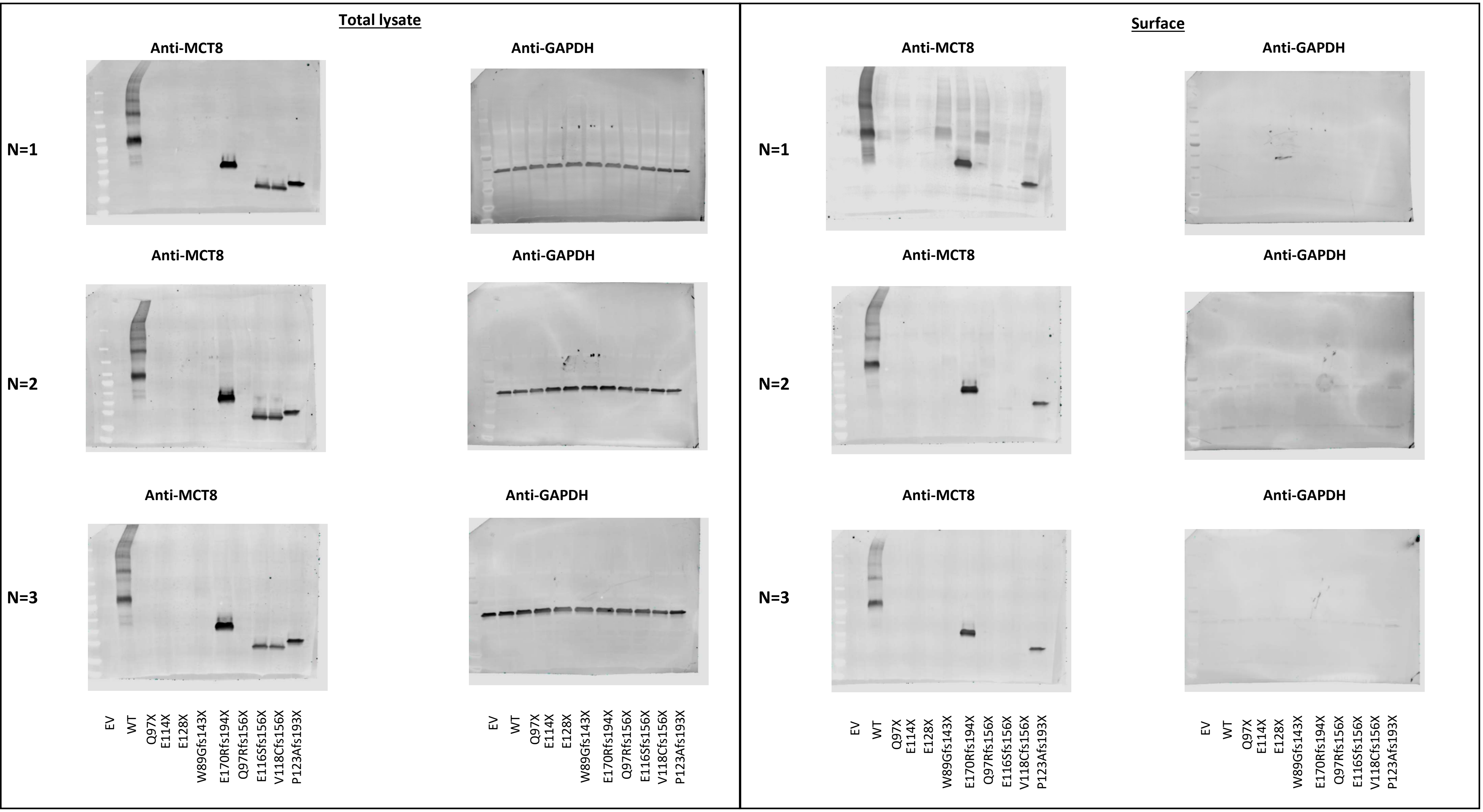

Total lysate
Surface
Anti-MCT8
Anti-GAPDH
N=1
N=2
N=3
EV
WT
Q97X
E114X
E128X
W89Gfs143X
E170Rfs194X
Q97Rfs156X
E116Sfs156X
V118Cfs156X
P123Afs193X

Series 16

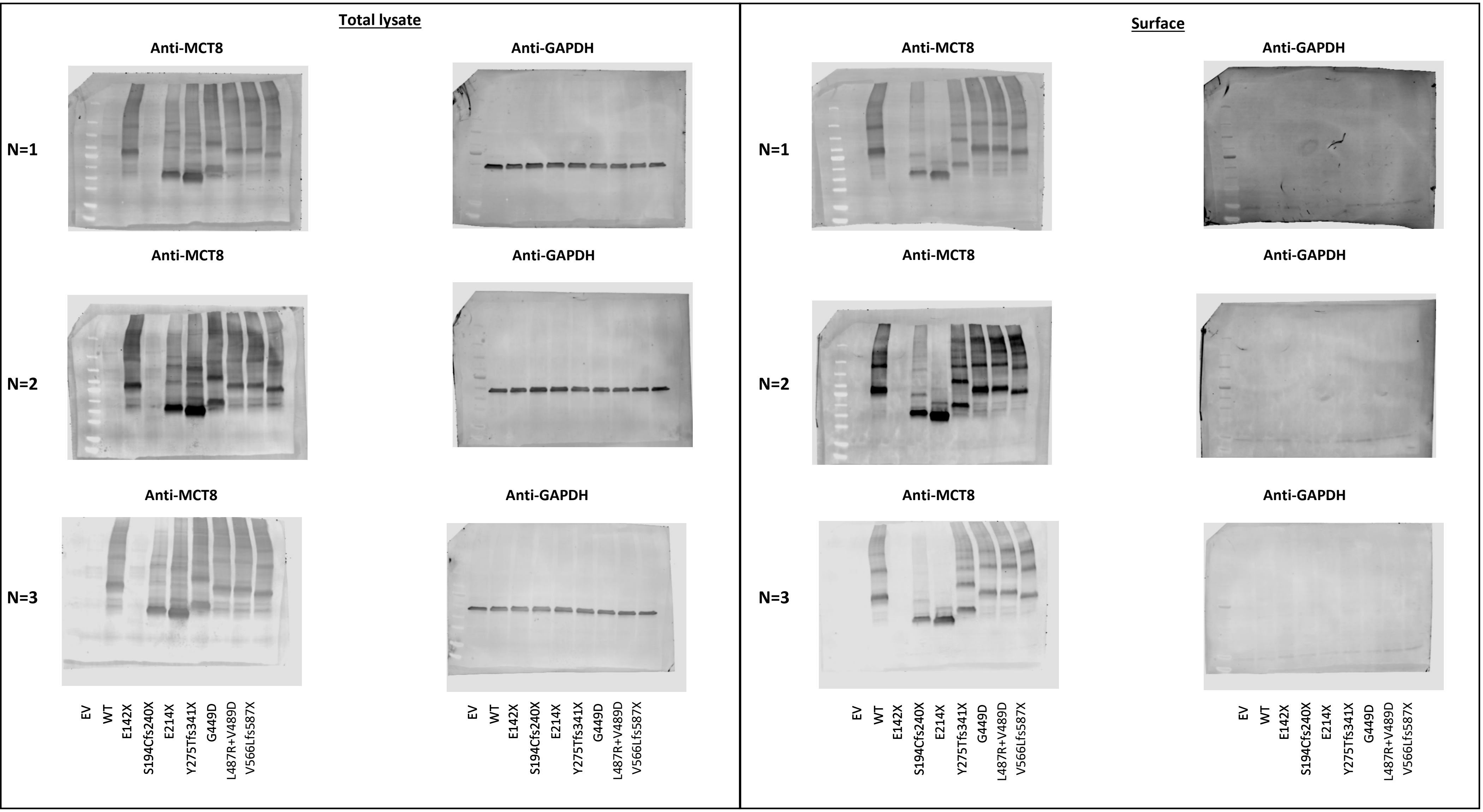

Total lysate
Surface
Anti-MCT8
Anti-GAPDH
N=1
N=2
N=3
EV
WT
E142X
S194Cfs240X
E214X
Y275Tfs341X
G449D
L487R+V489D
V566Lfs587X

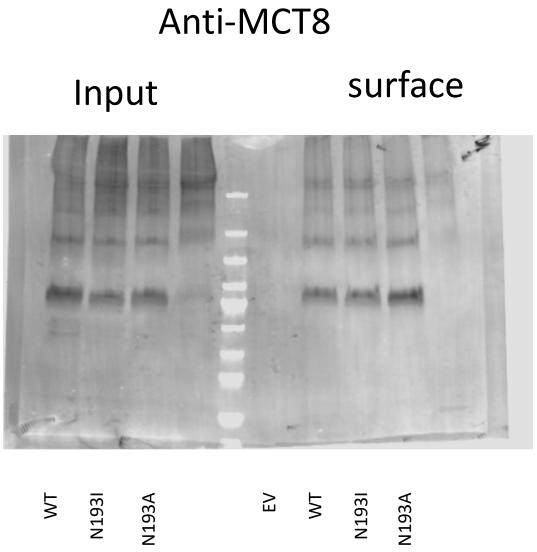
Anti-MCT8
Input
surface
WT
N193I
N193A
EV
WT
N193I
N193A

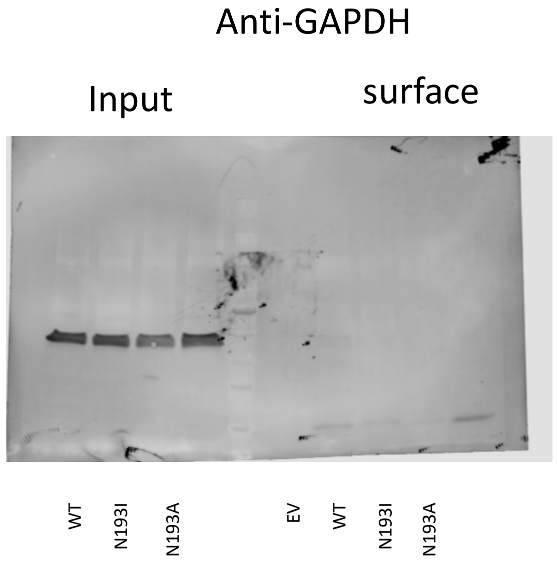
Anti-GAPDH
Input
surface
WT
N193I
N193A
EV
WT
N193I
N193A
