# Supplementary material for: Mapping variants in thyroid hormone transporter MCT8 to disease severity by genomic, phenotypic, functional, structural and deep learning integration
Source: Nat Commun. 2025 Mar 12;16:2479. doi: 10.1038/s41467-025-56628-w (PMC11904026; doi:10.1038/s41467-025-56628-w)
Supplement: Supplementary file 2 — Description of Additional Supplementary Files [file 41467_2025_56628_MOESM2_ESM.pdf]

Description of Additional Supplementary Files

File Name: Supplementary Data 1

Description: This file contains the statistical tests and p values underlying the data shown in all figures.
