# Supplementary material for: Mapping variants in thyroid hormone transporter MCT8 to disease severity by genomic, phenotypic, functional, structural and deep learning integration
Source: Nat Commun. 2025 Mar 12;16:2479. doi: 10.1038/s41467-025-56628-w (PMC11904026; doi:10.1038/s41467-025-56628-w)
Supplement: Supplementary file 4 — Reporting Summary [file 41467_2025_56628_MOESM4_ESM.pdf]

nature portfolio

Corresponding author(s): W. Edward Visser
Last updated by author(s): Sep 25, 2024

Reporting Summary

Nature Portfolio wishes to improve the reproducibility of the work that we publish. This form provides structure for consistency and transparency in reporting. For further information on Nature Portfolio policies, see our Editorial Policies and the Editorial Policy Checklist.

Statistics

For all statistical analyses, confirm that the following items are present in the figure legend, table legend, main text, or Methods section.

n/a	Confirmed	
☐	☒	The exact sample size (*n*) for each experimental group/condition, given as a discrete number and unit of measurement
☐	☒	A statement on whether measurements were taken from distinct samples or whether the same sample was measured repeatedly
☐	☒	The statistical test(s) used AND whether they are one- or two-sided<br>*Only common tests should be described solely by name; describe more complex techniques in the Methods section.*
☐	☒	A description of all covariates tested
☐	☒	A description of any assumptions or corrections, such as tests of normality and adjustment for multiple comparisons
☐	☒	A full description of the statistical parameters including central tendency (e.g. means) or other basic estimates (e.g. regression coefficient) AND variation (e.g. standard deviation) or associated estimates of uncertainty (e.g. confidence intervals)
☐	☒	For null hypothesis testing, the test statistic (e.g. *F*, *t*, *r*) with confidence intervals, effect sizes, degrees of freedom and *P* value noted<br>*Give P values as exact values whenever suitable.*
☒	☐	For Bayesian analysis, information on the choice of priors and Markov chain Monte Carlo settings
☒	☐	For hierarchical and complex designs, identification of the appropriate level for tests and full reporting of outcomes
☐	☒	Estimates of effect sizes (e.g. Cohen's *d*, Pearson's *r*), indicating how they were calculated

Our web collection on statistics for biologists contains articles on many of the points above.

Software and code

Policy information about availability of computer code

Data collection	Patient related data was collected by members of the study team.<br>Radiolabeled T3 and T4 counts for transport assays were acquired by a gamma counter (Perkin Elmer 2470 Wizard2 automatic gamma counter).<br>Confocal microscopy was done by a Zeiss Meta 510 microscope.
Data analysis	Expression levels of MCT8 by surface biotinylation were quantified using IMAGE J version 1.52a.<br>Homology modelling was done using YASARA Structure Software (YASARA Biosciences GmbH, Vienna, Austria). All images were created using YASARA Structure and Pov-Ray v3.6 software (www.povray.org).<br>Genotype-phenotype correlations were performed using GraphPad 9.<br>Confocal images were analysed using Zeiss LSM software (Carl Zeiss).<br>For conservation alignments, Consurf2010 was used through http://consurf.tau.ac.il.<br><br>We screened for non-synonymous variant data from control-only individuals in gnomAD v4.0 (https://gnomad.broadinstitute.org/).<br><br>We used publicly available software for genetic (look-up) analyses included in this study. Details about them including (where applicable) the computer codes are available in the following URLs:<br>SAIGE (https://saigegit.github.io/SAIGE-doc/)<br>GCTA-fastBAT (https://yanglab.westlake.edu.cn/software/gcta/#Overview)<br>GCTA-fastGWA (https://yanglab.westlake.edu.cn/software/gcta/#Overview)<br>GGPLOT2 (https://ggplot2.tidyverse.org/reference/ggplot.html)

PLINK (https://zzz.bwh.harvard.edu/plink/download.shtml)

For generating the multiple sequence alignment used in the classifier, we followed a protocol similar to the Frazer et al (PMID: 34707284), which builds on the profile HMM homology search tool Jackhmmer (PMID: 22039361), where sequences were obtained from the UniRef100 database of non-redundant protein (PMID: 25398609). The codebase for the EVE (PMID: 34707284) model is available at github.com/OATML-Markslab/EVE.

Figures of the mutational landscape Figures were created by using SeaBorn Python Library (Waskom ML 2021 Seaborn: Statistical data visualization. J Open Source Software 6:3021).

The codebase for the MCT8 classifier is available at github: https://github.com/martin-mariano/MCT8classifier

For manuscripts utilizing custom algorithms or software that are central to the research but not yet described in published literature, software must be made available to editors and reviewers. We strongly encourage code deposition in a community repository (e.g. GitHub). See the Nature Portfolio guidelines for submitting code & software for further information.

Data

Policy information about availability of data

All manuscripts must include a data availability statement. This statement should provide the following information, where applicable:

- Accession codes, unique identifiers, or web links for publicly available datasets
- A description of any restrictions on data availability
- For clinical datasets or third party data, please ensure that the statement adheres to our policy

The MCT8 homology model was generated based on the Cryo-EM structures of MCT1 (Protein Data Bank [PDB]# 6LZ0, 7CKR, 6LYY) and MCT2 (PDB# 7BP3), as well as the crystal structure of the major facilitator superfamily protein FucP (PDB# 3O7Q) and bacterial MFS (PDB# 6HCL).

Research involving human participants, their data, or biological material

Policy information about studies with human participants or human data. See also policy information about sex, gender (identity/presentation), and sexual orientation and race, ethnicity and racism.

Reporting on sex and gender	Patients were all male (MCT8 is at the X-chromosome). Genetic look-up was performed in male and female (self-reported data).
Reporting on race, ethnicity, or other socially relevant groupings	Different ancestries were included. Ethnicity was reported by parents or physicians.
Population characteristics	For genetic lookup: population based studies were used. Details are included in PMID: 30367059 and PMID: 35113692.
Recruitment	We utilized available data from patients with MCT8 deficiency.No participants were recruited specifically fort his projected. Further information on recruitment of the population studies are included in PMID: 30367059 and PMID: 35113692.
Ethics oversight	The process of disease outcomes collection was evaluated and approved by the appropriate local institutional review boards or ethics committees. However, for the retrospective analysis of existing datasets of patients in routine clinical care, most centres did not require additional specific institutional review board approval. For other centres, studies were either ethically approved or the ethics committee provided a waiver for approval.

Note that full information on the approval of the study protocol must also be provided in the manuscript.

Field-specific reporting

Please select the one below that is the best fit for your research. If you are not sure, read the appropriate sections before making your selection.

[x] Life sciences [ ] Behavioural & social sciences [ ] Ecological, evolutionary & environmental sciences

For a reference copy of the document with all sections, see nature.com/documents/nr-reporting-summary-flat.pdf

Life sciences study design

All studies must disclose on these points even when the disclosure is negative.

Sample size	Sample size for human participants was not calculated a priori, with all available individuals with a mutation in SLC16A2 being included. For molecular experiments, no sample size was calculated, but results were collected from at least three biological replicates as described in figure legends to ensure consistent phenotypes and to perform statistical analyses.
Data exclusions	For patients with MCT8 deficiency, only males were included. For genetic look-up studies, participants aged <18 years, of non-European ancestry, using thyroid medication, or with a history of thyroid surgery were excluded from all analyses.
Replication	No formal replication was performed as no additional independent samples were available.

Randomization	Randomization for patients with MCT8 deficiency is not applicable. The results on which the genetic look-up was performed were analysed and adjusted for age, sex and relevant study-specific covariates such as principal components for population stratification, study center and family-structure if applicable.
Blinding	As patients with MCT8 deficiency were investigated with most of the data collected being part of the clinical diagnosis and management, blinding of the investigators was not possible.

Reporting for specific materials, systems and methods

We require information from authors about some types of materials, experimental systems and methods used in many studies. Here, indicate whether each material, system or method listed is relevant to your study. If you are not sure if a list item applies to your research, read the appropriate section before selecting a response.

Materials & experimental systems

n/a	Involved in the study	
☐	☒	Antibodies
☐	☒	Eukaryotic cell lines
☒	☐	Palaeontology and archaeology
☒	☐	Animals and other organisms
☐	☒	Clinical data
☒	☐	Dual use research of concern
☒	☐	Plants

Methods

n/a	Involved in the study	
☒	☐	ChIP-seq
☒	☐	Flow cytometry
☒	☐	MRI-based neuroimaging

Antibodies

Antibodies used	We report information on antibodies in the following order: Name of AB; Species raised; (P or M); Manufacturer (and catalogue number); Dilution used for WB; Dilution used for ICH; RRID; Ref<br>MCT8; Rabbit (P); ATLAS (HPA003353); 1:2,000; 1:1,000; AB_1079343; (65) GAPDH; Mouse (M); Millipore (Mab 374); 1:20,000; N/A; AB_2107445; (66) ZO1; Mouse (M); Thermo Fisher (33-9100); N/A; 1:500; AB_2533147; (67) RDye800; Goat; LI-COR (926-32211);1:20,000;N/A; AB_621843;(68) IRDye680;Goat; LI-COR (926-68020); 1:20,000; N/A;AB_10706161;(69) Alexa 488; Goat;Thermo Fisher (A11008); N/A; 1:1,000; AB_143165;(70) Alexa 633; Goat; Thermo Fisher (A21050); N/A;1:1,000; AB_2535718; (71)<br>See Supplementary Table 8 for further details.
Validation	All antibodies were validated by commercial suppliers. Detailed information can be found on the commercial websites.<br>65. RRID:AB_1079343, https://scicrunch.org/resolver/AB_1079343.<br>66. RRID:AB_2107445, https://scicrunch.org/resolver/AB_2107445.<br>67. RRID:AB_2533147, https://scicrunch.org/resolver/AB_2533147.<br>68. RRID:AB_621843, https://scicrunch.org/resolver/AB_621843.<br>69. RRID:AB_10706161, https://scicrunch.org/resolver/AB_10706161.<br>70. RRID:AB_143165, https://scicrunch.org/resolver/AB_143165.<br>71. RRID:AB_2535718, https://scicrunch.org/resolver/AB_2535718.<br>See the following references in the manuscript: (65) (66) (67) (68) (69) (70) (71)

Eukaryotic cell lines

Policy information about cell lines and Sex and Gender in Research

Cell line source(s)	Patient cells were all derived from male patients (MCT8 is at the X-chromosome). COS-1 African green monkey kidney (CVCL_0223) and JEG-3 human choriocarcinoma (CVCL_0363) cells were obtained from ECACC (Sigma-Aldrich).
Authentication	None of the cell lines were authenticated, but all of them were tested for thyroid hormone uptake and proven diminished thyroid hormone transport
Mycoplasma contamination	Mycoplasma was tested negative
Commonly misidentified lines (See ICLAC register)	No commonly misidentified cell lines were used.

Clinical data

Policy information about clinical studies

All manuscripts should comply with the ICMJE guidelines for publication of clinical research and a completed CONSORT checklist must be included with all submissions.

Clinical trial registration	Our cohort consisted of patients who had been enrolled in the Triac Trial I (PMID: 31377265), patients who participated in the named patient program for Triac treatment (PMID: 34679181), and historical cases for whom the Erasmus University Medical Center (Erasmus MC; Rotterdam, Netherlands) fulfilled a consultancy role following the first reports of MCT8 deficiency in 2004. To this cohort we added patients identified through a systematic literature review.
Study protocol	not applicable
Data collection	not applicable
Outcomes	not applicable

Plants

Seed stocks	*Report on the source of all seed stocks or other plant material used. If applicable, state the seed stock centre and catalogue number. If plant specimens were collected from the field, describe the collection location, date and sampling procedures.*
Novel plant genotypes	*Describe the methods by which all novel plant genotypes were produced. This includes those generated by transgenic approaches, gene editing, chemical/radiation-based mutagenesis and hybridization. For transgenic lines, describe the transformation method, the number of independent lines analyzed and the generation upon which experiments were performed. For gene-edited lines, describe the editor used, the endogenous sequence targeted for editing, the targeting guide RNA sequence (if applicable) and how the editor was applied.*
Authentication	*Describe any authentication procedures for each seed stock used or novel genotype generated. Describe any experiments used to assess the effect of a mutation and, where applicable, how potential secondary effects (e.g. second site T-DNA insertions, mosiacism, off-target gene editing) were examined.*
